# Efficacy and safety of KN026, a bispecific anti-HER2 antibody, in combination with KN046, an anti-CTLA4/PD-L1 antibody, in patients with advanced HER2-positive nonbreast cancer: a combined analysis of a phase Ib and a phase II study

**DOI:** 10.1038/s41392-025-02195-x

**Published:** 2025-03-19

**Authors:** Dan Liu, Jifang Gong, Jian Li, Changsong Qi, Zuoxing Niu, Bo Liu, Zhi Peng, Suxia Luo, Xicheng Wang, Yakun Wang, Rusen Zhao, Lilin Chen, Ting Deng, Zhen Li, Lei Chen, Meimei Fang, Hongwei Yang, Linzhi Lu, Yanming Zhang, Fengling Kang, Ting Xu, Xiaotian Zhang, Lin Shen

**Affiliations:** 1 Key Laboratory of Carcinogenesis and Translational Research (Ministry of Education/Beijing), Department of Early Drug Development Center, Peking University Cancer Hospital and Institute, Beijing, China; 2 State Key Laboratory of Holistic Integrative Management of Gastrointestinal Cancers, Beijing Key Laboratory of Carcinogenesis and Translational Research, Department of Gastrointestinal Oncology, Peking University Cancer Hospital and Institute, Beijing, China; 3 State Key Laboratory of Holistic Integrative Management of Gastrointestinal Cancers, Beijing Key Laboratory of Carcinogenesis and Translational Research, Department of Early Drug Development Center, Peking University Cancer Hospital and Institute, Beijing, China; 4 Department of Medical Oncology, Cancer Hospital of Shandong First Medical University (Shandong Cancer Hospital), Jinan, Shandong China; 5 Department of Internal Medicine, Affiliated Tumor Hospital of Zhengzhou University, Henan Cancer Hospital, Zhengzhou, Henan China; 6 Key Laboratory of Carcinogenesis and Translational Research (Ministry of Education/Beijing), Department of Gastrointestinal Oncology, Peking University Cancer Hospital and Institute, Beijing, China; 7 Department of Medical Oncology, Zibo Municipal Hospital, Zibo, Shandong China; 8 Department of Medical Oncology, The First Affiliated Hospital of Xiamen University, Xiamen, Fujian China; 9 Department of GI Oncology, Tianjin Medical University Cancer Institute and Hospital, Tianjin, China; 10 Department of Internal Medicine Ward 5, Linyi Cancer Hospital, Linyi, Shandong China; 11 Department of Medical Oncology, Cancer Hospital of Shantou University Medical College, Shantou, Guangdong China; 12 Department of Breast and Thyroid Surgery, Suining Central Hospital, Suining, Sichuan China; 13 Gastroenterology Department, Gansu Wuwei Tumour Hospital, Wuwei, Gansu China; 14 Oncology Inpatient Area 2/3, Linfen Central Hospital, Linfen, Shanxi China; 15 Jiangsu Alphamab Biopharmaceuticals Co., Ltd., Suzhou, Jiang Su, China

**Keywords:** Drug development, Gastrointestinal cancer

## Abstract

To evaluate the efficacy and safety of KN026, a novel bispecific HER2 (ECD2 and ECD4) antibody, plus KN046, a PD-L1, and CTLA4 bispecific antibody, in patients with advanced HER2-positive solid tumors. We conducted two sequentially designed phase Ib and II studies with similar target populations and evaluation schedules. The primary endpoints included safety, maximum tolerated dose (MTD), the recommended phase II dose (RP2D) for the phase Ib study, and the objective response rate (ORR) and duration of response (DoR) for the phase II study. Hereby, we solely report the results from 113 nonbreast cancer patients. In phase Ib, MTD was not reached. Dose 3 was confirmed to be acceptable for the phase II study. An objective response has been exclusively observed in HER2-positive patients. Any grade treatment-related adverse events (TRAEs) were reported in 108 (95.6%) patients. The most common TRAEs were infusion reactions (38.9%), anemia (37.2%), elevated AST (31.0%), and diarrhea (30.1%). Among the 108 patients evaluated for efficacy, the overall ORR was 55.6% (95%CI, 45.7%, 65.1%). In the HER2-positive GC subgroup, 38 patients received this regimen as the 1st-line treatment and 30 patients achieved an objective response, with an ORR of 78.9% (95%CI, 62.7%, 90.4%). Among 27 pretreated patients, the ORR was 44.4% (95%CI, 25.5%, 64.7%). In the other HER2-positive solid tumor subgroup (n = 34), the ORR was 52.9% (95%CI 35.1%,70.2%). Thus, KN026 plus KN04 exhibits promising efficacy and acceptable safety profiles in HER2-positive nonbreast cancer, as does the 1st-line treatment for GC.

## Introduction

The human epidermal growth factor receptor-2 (HER2) has been established as a pivotal therapeutic target across multiple solid tumor types, particularly in gastrointestinal malignancies (including gastric and colorectal cancers), biliary tract cancers, and non-small cell lung cancer (NSCLC).^1–5^ Trastuzumab has consistently maintained its golden position as the first-line therapeutic standard of care for HER2-positive malignancies.^1,2^ Despite its therapeutic efficacy, a substantial proportion of patients (~30–50%) fail to derive significant benefit from trastuzumab, with disease progression typically occurring within 6–10 months after treatment initiation.^2,6,7^ Current therapeutic strategies, including anti-HER2 antibody-drug conjugates (ADCs), VEGFR2-targeted therapies, and conventional chemotherapy, have shown only limited improvements in survival outcomes.^8–10^ This highlights the urgent need for more efficacious treatment modalities, particularly innovative anti-HER2 therapeutic approaches. It is noteworthy that all currently approved anti-HER2 therapies for nonbreast cancers, including gastric cancer (GC), specially target the HER2 extracellular domain 4 (ECD4). While chemotherapy remains a fundamental component of current treatment regimens. A significant portion of patients are unable to tolerate the side effects of chemotherapy or choose to refuse chemotherapy. Therefore, there is a crucial unmet need for the development of improved combination therapies, with a particular focus on chemotherapy-free treatment regimens. This shift could potentially enhance patient outcomes and quality of life, addressing the limitations of existing therapies and providing more tolerable and effective options for HER2-positive cancer patients.

Our research group has previously demonstrated a distinct tumor immune microenvironment in HER2-positive gastric cancer (GC), revealing a significantly reduced population of exhausted T cells (characterized by CD8 + PD-1 + LAG-3 + TIM-3− and CD8 + PD-1 − TIM-3+ phenotypes) interacting with tumor cells compared to HER2-negative GC, suggesting enhanced immunotherapeutic potential in HER2-positive malignancies.^11^ Stagg et al.’s work demonstrates that PD-1 blockade synergistically enhances trastuzumab’s therapeutic efficacy.^12^ Clinical validation comes from the KEYNOTE-811 trial, which established that first-line treatment with pembrolizumab in combination with trastuzumab and chemotherapy significantly improves objective response rate (ORR) and progression-free survival (PFS) in HER2-positive GC.^7^ The immunotherapeutic landscape is further enriched by the role of cytotoxic T lymphocyte-associated antigen-4 (CTLA4), a critical T-cell co-receptor and one of the most extensively characterized immune checkpoints, which is frequently overexpressed across various tumor types.^13,14^ Preclinical and clinical evidence consistently demonstrates complementary therapeutic effects when combining CTLA4 blockade with PD-1 or PD-L1 checkpoint inhibitor.^15–19^ Additionally, emerging data indicate that dual targeting of HER2 extracellular domains (ECD2 and ECD4) yields superior clinical outcomes, with reported ORR improvements of 8-13% in HER2-positive tumors.^3,20,21^ Based on these previous studies, we propose that a novel therapeutic strategy simultaneously targeting the HER2 (through ECD2/4 dual blockade), PD-1/PD-L1, and CTLA4 pathways could potentially revolutionize treatment paradigms for HER2-positive solid tumors. This combination therapeutic regimen may not only enhance antitumor activity but also pave the way for chemotherapy-free treatment modalities in HER2-positive solid tumors.

KN026 represents a novel bispecific antibody targeting two distinct HER2 epitopes (ECD2 and ECD4), with its structural configuration detailed in Supplementary Fig. 1a. This innovative therapeutic agent maintains potent antibody-dependent cell-mediated cytotoxicity (ADCC) while demonstrating significant antiproliferative effects against HER2-overexpressing tumor cells in both in vitro and in vivo models.^22^ Clinical investigations have revealed that KN026 achieves improved objective response rates (ORR) in gastric cancer (GC) patients across a spectrum of HER2 expression levels, including those with prior trastuzumab treatment.^23^ The phase I dose escalation and expansion study established the recommended phase II dose (RP2D) at two alternative regimens: 20 mg/kg administered every 2 weeks (Q2W) or 30 mg/kg administered every 3 weeks (Q3W).^24^ KN046, a novel human IgG1 Fc-fused bispecific antibody manufactured using Chinese hamster ovary (CHO) cell expression systems, demonstrates dual immune checkpoint blockade through simultaneous targeting of PD-L1/PD-1 and CTLA4/CD80-CD86 interactions.^25^ As illustrated in Supplementary Fig. 1b, the molecular architecture combines two distinct functional domains through advanced protein engineering. Preclinical evaluations have revealed KN046’s enhanced binding affinity for PD-L1 compared to conventional monoclonal antibodies, coupled with its unique capacity to deplete tumor-infiltrating regulatory T cells (Tregs) within the tumor microenvironment.^26^ This dual mechanism of action potentiates antitumor immunity through concurrent enhancement of effector T-cell activity and attenuation of immunosuppressive cellular components. On the basis of the phase I clinical trial data in patients with advanced solid tumors, the RP2D of KN046 was established as 5 mg/kg administered every 2 weeks. Notably, KN046 demonstrated a more favorable safety profile compared to the combination therapy of nivolumab and ipilimumab, with significantly reduced incidence of grade 3–4 treatment-related adverse events.^25,27^

We sequentially conducted a phase Ib (ClinicalTrials.gov identifier: NCT04040699) and a phase II (ClinicalTrials.gov identifier: NCT04521179) clinical study employing almost identical inclusion criteria to evaluate the antitumor activity of KN026 combined with KN046 in advanced HER2-positive solid tumors. To enhance statistical precision in estimating treatment effects and safety profiles, a combined analysis of these two studies was warranted. This report details the integrated findings from both study phases.

## Results

### Study population characteristics

**Fig. 1:**
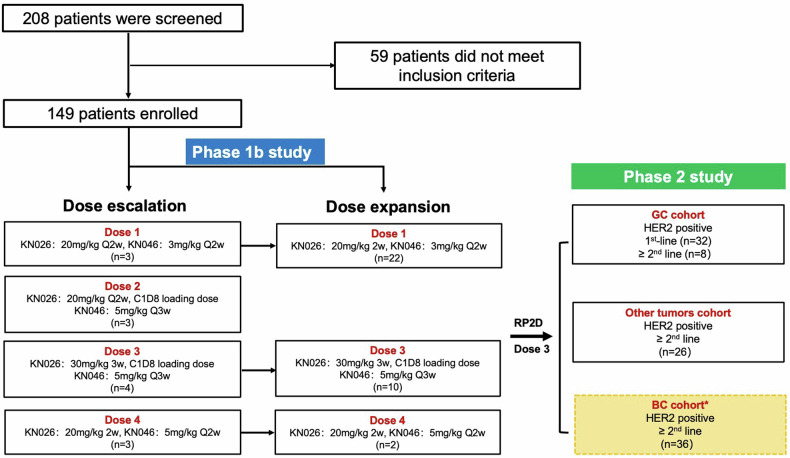
The diagram shows patient numbers and dispositions in the phase Ib and phase II studies. *The results of the BC cohort would not be reported in this article for immature data

From Oct. 2019 to Jan. 2022, 208 patients were screened and 149 patients were ultimately enrolled (phase Ib, n = 47; phase II, n = 102) (Fig. 1). Only the results for nonbreast cancer cohorts (n = 113) are reported in this article. An analysis of pharmacokinetics (PK) and immunogenicity is planned to be reported in an isolated article.

The baseline demographic and clinical characteristics are summarized in Table 1. Among them, 73 patients were males and 40 patients were females, with a median age of 57 years old (range 29.0–82.0 years). The tumor types included GC (n = 74), colorectal cancer (CRC) (n = 26), and other solid tumors (n = 13). All patients were mismatch repair proficient or microsatellite stable (pMMR/MSS). The majority of patients (n = 103, 91.2%) had HER2-positive tumors. PD-L1 status was tested in 43 of 74 (58.1%) GC patients at baseline. Twenty-six patients (60.5%) were PD-L1 positive, and 17 patients (39.5%) were PD-L1 negative. Notably, 39 HER2-positive GC patients received this regimen as the 1st-line treatment. With respect to treatment history, 74 patients were pretreated, with 30 patients (40.5%) receiving prior anti-HER2 therapies, 18 patients (24.3%) receiving prior PD-1/PD-L1 blockade, and 7 patients (9.5%) receiving both.

**Table 1:** The demographic and clinical characteristics

	Phase 1b^a^	Phase 2			Total
		GC/GEJ cancer	Other HER2-positive tumors		
	n = 47	(n = 40)	CRC (n = 15)	Non-CRC^b^ (n = 11)	n = 113
Median age, years (range)	56.0 (29.0, 74.0)	63.0 (34.0, 82.0)	55.0 (37.0, 67.0)	58.0 (41.0, 65.0)	57.0 (29.0, 82.0)
Sex					
Male	32 (61.8%)	29 (72.5%)	8 (53.3%)	4 (36.4%)	73 (64.6%)
Female	15 (31.9%)	11 (27.5%)	7 (46.7%)	7 (63.6%)	40 (35.4%)
ECOG					
0	6 (12.8%)	8 (20.0%)	6 (40.0%)	3 (27.3%)	23 (20.4%)
1	41 (87.2%)	32 (80.0%)	9 (60.0%)	7 (63.6%)	89 (78.8%)
NA	0	0	0	1 (9.1%)	1 (0.9%)
HER2 status^c^					
HER2 positive	38 (80.9%)	40 (100.0%)	15 (80.0%)	10 (90.9%)	103 (91.2%)
HER2 low-expression	7 (14.9%)	0	0	0	7 (6.2%)
HER2 mutation	2 (4.3%)	0	0	1 (9.1%)	3 (2.7%)
Liver metastasis					
Yes	28 (59.6%)	28 (70.0%)	10 (66.7%)	5 (45.5%)	71 (62.8%)
No	19 (40.4%)	12 (30.0%)	5 (33.3%)	6 (54.5%)	42 (37.2%)
Peritoneal metastasis					
Yes	7 (14.9%)	2 (5.0)	0	0	9 (8.0%)
No	40 (85.1%)	38 (95.0)	15 (100.0%)	11 (100.0%)	104 (92.0%)
Lymph node metastasis					
Yes	13 (27.7%)	13 (32.5%)	1 (6.7%)	4 (36.4%)	31 (27.4%)
No	34 (72.3%)	27 (67.5%)	14 (93.3%)	7 (63.6%)	82 (72.6%)
Treatment naïve	7 (14.9%)	32 (80.0%)	0	0	39 (34.5%)
Pretreated patients	40 (85.1%)	8 (20.0%)	15 (100.0%)	11(100.0%)	74 (65.5%)
Prior anti-HER2 therapies^d^	21 (52.5%)	4 (50.0%)	4 (26.7%)	1 (9.1%)	30 (40.5%)
Prior PD-1/PD-L1 blockades	10 (25.0%)	2 (25.0%)	1 (6.7%)	5 (45.5%)	18 (24.3%)
Both anti-HER2 therapies and PD-1/ PD-L1 blockades	5 (12.5%)	2 (25.0%)	0	0	7 (9.5%)

^a^In phase 1b part, 47 patients included GC (n = 34), CRC (n = 11), duodenum cancer (n = 1), abdominal cavity cancer (n = 1)

^b^Non-CRC cancer included renal cancer (n = 1), gallbladder cancer (n = 4), pancreatic cancer (n = 1), non-small cell lung cancer (n = 5)

^c^HER2 positive included HER2 IHC 3+, HER2 IHC 2+ with gene amplification confirmed by ISH method, and NGS HER2 copy number >6

^d^Anti-HER2 therapies included trastuzumab, pertuzumab, pyrotinib, disitamab vedotin (RC48), MBS301(a novel bispecific HER2 ECD2/ECD4 antibody)

### Dose determination for the phase II study

The combination dose of KN026 and KN046 for the phase II study was predefined as 30 mg/kg Q3w/5 mg/kg Q3w (Dose 3) when the study designed, based on the reported results. In the dose exploration part of the phase Ib study, 13 patients were enrolled (Dose 1, n = 3; Dose 2, n = 3; Dose 3, n = 4; Dose 4, n = 3). No DLT was observed and the MTD was not reached. Safety profile was acceptable at Dose 3 (Supplementary Table 1). In the dose expansion part of phase Ib study, one patient who was enrolled in Dose 1 died for treatment-related pulmonary arterial hypertension (PAH) (Supplementary Table 2). Dose 3 therapy showed greater efficacy in patients with HER2-positive and PD-L1-negative GC. Moreover, considering the convenience of clinical practice, Dose 3 was determined as the combination dose for the phase II study after Data Safety Monitoring Committee (DSMC) discussion.

### Antitumor activity and survival

By the cutoff date of May 20th, 2023, the median follow-up duration was 18.7 months (95%CI, 17.5, 19.7). In total, 108 patients completed the efficacy assessment, including 99 patients with HER2-positive tumors. Five patients dropped out before the first efficacy evaluation due to the COVID-19 outbreak. Among the patients assessed, 60 patients achieved an objective response (CR, n = 1; PR, n = 59), and 30 patients experienced SD. The overall ORR was 55.6% (95%CI, 45.7%, 65.1%), the DCR was 83.3% (95%CI, 78.1%, 92.0%) and the clinical benefit rate (CBR) was 61.1% (95%CI 51.3%, 70.3%). Among pretreated HER2-positive patients (n = 61), 27 patients had prior anti-HER2 therapies, and 14 patients (51.9%) achieved a PR. Nine of 15 patients (60.0%) who received prior PD-1/PD-L1 blockade treatment achieved a PR. The objective responses were still observed in 4 of the 6 patients (66.7%) pretreated with both anti-HER2 therapy and PD-1/PD-L1 blockade (Supplementary Table 3).

In the phase Ib study, initially, patients with HER2 positive, low-expression, and mutation status were included to explore safety and the benefit population. Objective responses were solely observed in HER2-positive patients. Additionally, HER2-positive patients had relatively longer median PFS (6.2 vs. 3.6 months, *p* = 0.035) and OS (NE vs. 13.4 months, *p* = 0.067) than those with HER2 low-expression or mutation (Supplementary Fig. 2). Thus, only patients with HER2-positive solid tumors were included in further studies. In the HER2-positive GC subgroup, 7 of the 11 patients (63.5%) who received this regimen as 2nd-line treatment achieved a PR, 5 of them (45.5%) achieved a confirmed PR. Encouraged by the efficacy of 2nd-line treatment, treatment-naïve patients with advanced HER2-positive GC were enrolled after DSMC discussion. Six of the 7 treatment (85.7%) naïve patients achieved a PR (Supplementary Table 4). Thus, the use of this regimen in the 1st-line treatment was further explored in a phase II study.

**Fig. 2:**
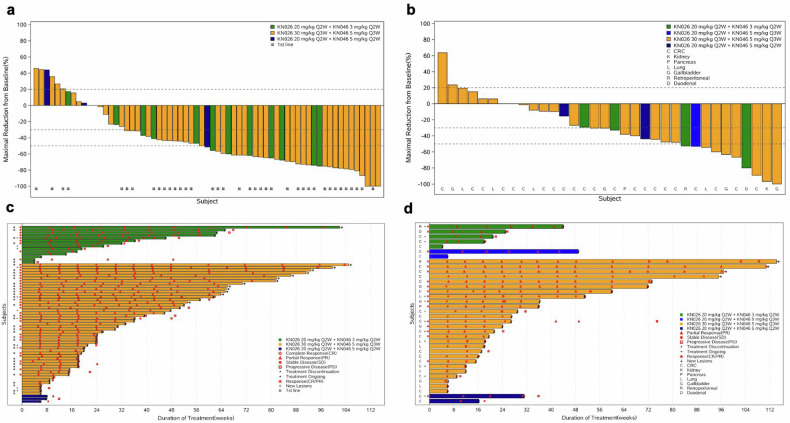
Treatment response and duration of evaluable patients with HER2-positive tumors in the phase Ib and phase II studies. Waterfall plot of the best objective response of patients with HER2-positive GC (**a**), CRC, and other solid tumors (**b**); swimming plot of the treatment duration of patients with HER2-positive GC (**c**), CRC and other solid tumors (**d**)

**Fig. 3:**
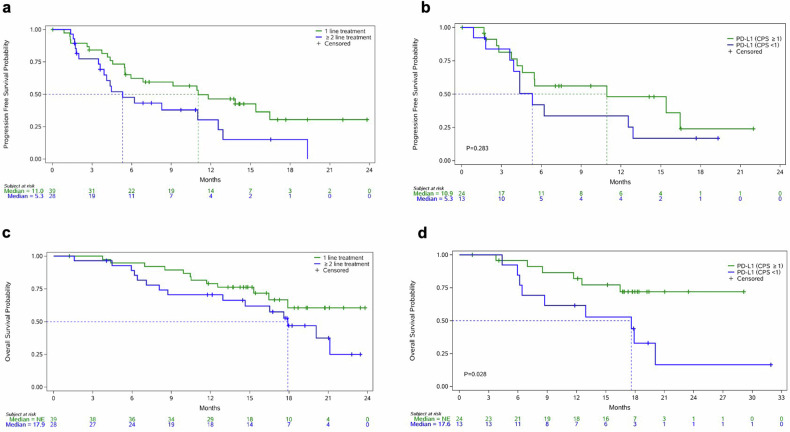
Survival curves of patients with HER2-positive GC evaluated in the phase Ib and phase II studies. Kaplan–Meier plot of PFS for different treatment lines (**a**) and PD-L1 statuses (**b**). Kaplan–Meier plot of OS for different treatment lines (**c**) and PD-L1 statuses (**d**)

In total, 38 HER2-positive GC patients who received this regimen as the 1st-line treatment were ultimately enrolled in the both phase Ib and phase II studies. Thirty patients achieved an objective response (CR, n = 1; PR, n = 29), with an ORR and DCR of 78.9% (95%CI, 62.7%, 90.4%) and 89.5% (95%CI, 75.2%, 97.1%), respectively. Only 3 patients experienced disease progression at the first efficacy assessment (Table 2). The median DoR was 13.4 months (95%CI, 8.3, NE), and median PFS was 11.0 months (95%CI, 5.5, 16.5). The median OS was not reached. In addition, 27 patients HER2-positive GC received KN026 plus KN046 as a ≥2nd-line treatment. Twelve patients achieved a PR, and 11 patients experienced SD, with an ORR of 44.4% (95%CI, 25.5%, 64.7%) and a DCR of 85.2% (95%CI, 66.3%, 95.8%). The median DoR was 10.8 months (95%CI, 4.0, NE) and median PFS was 5.3 months (95%CI, 3.6, 12.6). The best percentage change from baseline in terms of the size of the tumor lesions, duration of response, and survival curves of PFS and OS in the HER2-positive GC cohort are shown in Figs. 2a, c and 3a, c.

**Table 2:** The efficacy and survival among evaluable HER2-positive patients in the phase Ib and phase II studies

	GC		Other tumors		
	1st-line treatment (n = 38)	≥2nd-line treatment (n = 27)	CRC (n = 22)	Non-CRC^a^ (n = 12)	Total (n = 34)
Best overall response					
CR	1 (2.6%)	0	0	0	0
PR	29 (76.3%)	12 (44.4%)	10 (45.5%)	8 (66.7%)	18 (52.9%)
SD	4 (10.5%)	11 (40.7%)	11 (50.0%)	2 (16.7%)	13 (38.2%)
PD	3 (10.5%)	4 (14.8%)	1 (4.5%)	2 (16.7%)	3 (8.8%)
Objective response rate (ORR, 95%CI)	78.9% (62.7%, 90.4%)	44.4% (25.5%, 64.7%)	45.5% (24.4%,67.8%)	66.7% (34.9%, 90.1%)	52.9%(35.1%, 70.2%)
Disease control rate (DCR, 95%CI)	89.5% (75.2%, 97.1%)	85.2% (66.3%, 95.8%)	95.5% (77.2%, 99.9%)	83.3% (51.6%, 97.6%)	91.2% (76.3%, 98.1%)
Clinical benefit rate (CBR, 95%CI)	78.9% (62.7%, 90.4%)	51.9% (31.9%, 71.3%)	54.5 (32.2%, 75.6%)	66.7% (34.9%, 90.1%)	58.8% (40.7%, 75.4%)
Duration of response (DoR, 95%CI)	13.4 (8.3, NE)	10.8 (4.0, NE)	8.1 (3.2, NE)	NE (11.1, NE)	5.0 (4.1, 15.2)
Progression-free survival (PFS, 95%CI)	11.0 (5.5, 16.5)	5.3 (3.6, 12.6)	5.6 (3.7, 16.6)	25.0 (6.0, 50.5)	5.6 (3.9, 8.2)
Overall survival (OS, 95%CI)	NE (16.5, NE)	17.9 (8.7, NE)	NE (19.2, NE)	16.7 (2.7, 41.3)	NE (19.2, NE)
6-month progression-free rate	62.4% (44.8%, 75.7%)	47.6% (27.1%, 65.6%)	45.5% (23.2%, 65.3%)	25.0% (6.0%, 50.5%)	37.2% (20.8%, 53.7%)
12-month progression-free rate	46.4% (29.3%, 62.0%)	30.3% (12.0%, 51.1%)	39.7% (18.6, 60.2%)	16.7% (2.7%, 41.3%)	30.5% (15.5%, 46.9%)

^a^Non-CRC cancer included duodenum cancer (n = 1), abdominal cavity cancer (n = 1), renal cancer (n = 1), gallbladder cancer (n = 4), non-small cell lung cancer (n = 4), pancreatic cancer (n = 1)

The relationships between antitumor activity and PD-L1 status in HER2-positive GC were also analyzed (Supplementary Table 5). Among the patients whose efficacy data were effectively evaluated (n = 65), 36 patients had baseline PD-L1 data. Twenty-three patients were PD-L1 positive, and 16 patients achieved a PR, with an ORR of 69.5%. The median PFS was 10.9 months (95%CI, 4.2, NE) and the median OS was not reached. The DoR was 13.4 months (95%CI, 3.3, NE). Thirteen patients were PD-L1 negative. Seven patients achieved a PR, with an ORR of 53.8%. The median PFS was 5.3 months (95%CI, 1.8, 12.9) and median DoR was 10.8 months (95%CI 3.1, NE). Compared with those in patients with PD-L1-negative tumors, both the median PFS and OS were relatively longer in those with PD-L1-positive tumors (*p* = 0.283 for PFS and *p* = 0.028 for OS) (Fig. 3b, d).

In addition, 34 patients with HER2-positive other solid tumors (CRC, n = 22; non-CRC, n = 12) were evaluated in this study. The efficacy is shown in Table 2. Eighteen patients achieved a PR and 13 patients experienced SD, with an ORR of 52.9% (95%CI, 35.1%,70.2%) and DCR of 91.2% (95%CI, 76.3%, 98.1%). The median DoR was 5.0 months (95%CI, 3.2, NE) and median PFS was 5.6 months (95%CI, 3.9, 8.2). The best percentage change from baseline in the size of tumor lesions and the duration of response in other HER2-positive tumors were shown in Fig. 2b, d.

### Safety analysis

All enrolled patients (n = 113) who received at least 1 cycle of KN026 plus KN046 administration were included in the safety assessment. The median treatment period for KN026 was 8.0 (range 1.0–50.0) cycles and that for KN046 was 6.0 (range 1.0–50.0) cycles. The safety profiles are summarized in Table 3 and Supplementary Table 6.

**Table 3:** Any grade treatment-related adverse events (TRAEs) occurring in ≥5% of patients and all grade ≥3 TRAEs in the phase Ib and phase II studies

	Phase 1b		Phase 2		Total	
	N = 47 (%)		N = 66 (%)		N = 113 (%)	
	Grade ≥ 3	All grades	Grade ≥ 3	All grades	Grade ≥ 3	All grades
All	10 (21.3)	44 (93.6)	25 (37.9)	54 (97.0)	35 (31.0)	108 (95.6)
Infusion reactions	1 (2.1)	17 (36.2)	1 (1.5)	27 (40.9)	2 (1.8)	44 (38.9)
Anemia	2 (4.3)	22 (46.8)	4 (6.1)	20 (30.3)	6 (5.3)	42 (37.2)
Elevated AST	0	15 (31.9)	4 (6.1)	20 (30.3)	4 (3.5)	35 (31.0)
Diarrhea	0	13 (27.7)	2 (3.0)	21 (31.8)	2 (1.8)	34 (30.1)
Elevated ALT	1 (2.1)	12 (25.5)	2 (3.0)	20 (30.3)	3 (2.7)	32 (28.3)
Leukemia	0	9 (19.1)	0	19 (28.8)	0	28 (24.8)
Rash	0	11 (23.4)	1 (1.5)	16 (24.2)	1 (0.9)	27 (23.9)
Elevated TBIL	0	9 (19.1)	0	11 (16.7)	0	20 (17.7)
Neutropenia	1 (2.1)	7 (14.9)	0	13 (19.7)	1 (0.9)	20 (17.7)
Hypothyroidism	0	6	0	11 (16.7)	0	17 (15.0)
Elevated DBIL	0	1 (2.1)	2 (3.0)	15 (18.2)	2 (1.8)	16 (14.2)
Weight loss	0	2	1 (1.5)	12 (18.2)	1 (0.9)	14 (12.4)
Thrombocytopenia	1 (2.1)	8 (17.0)	1 (1.5)	5 (7.6)	2 (1.8)	13 (11.5)
Fever	0	3 (6.4)	1 (1.5)	8 (12.1)	1 (0.9)	11 (9.7)
Fatigue	0	1 (2.1)	1 (1.5)	9 (13.6)	1 (0.9)	10 (8.8)
Hyponatremia	0	3 (6.4)	1 (1.5)	6 (9.1)	1 (0.9)	9 (8.0)
Vomiting	0	2 (4.3)	1 (1.5)	7 (10.6)	1 (0.9)	9 (8.0)
Anorexia	0	0	0	9 (13.6)	0	9 (8.0)
Hypokalemia	0	3 (6.4)	1 (1.5)	5 (7.6)	1 (0.9)	8 (7.1)
Hyperthyroidism	0	3 (6.4)	0	5 (7.6)	0	8 (7.1)
Elevated creatine	0	2 (4.3)	0	6 (9.1)	0	8 (7.1)
Elevated GGT	1 (2.1)	2 (4.3)	0	5 (7.6)	1 (0.9)	7 (6.2)
Proteinuria	0	5 (10.6)	0	2 (3.0)	0	7 (6.2)
Pruritus	1	1 (2.1)	1 (1.5)	6 (9.1)	2 (1.8)	7 (6.2)
Elevated TSH	0	3 (6.4)	0	3 (4.5)	0	6 (5.3)
Elevated cTnI	0	2 (4.3)	1 (1.5)	3 (4.5)	1 (0.9)	5 (4.4)
Interstitial lung disease	0	3 (6.4)	1 (1.5)	2 (3.0)	1 (0.9)	5 (4.4)
Hypopituitarism	0	0	2 (3.0)	2 (3.0)	2 (1.8)	2 (1.8)
Abdominal pain	0	1 (2.1)	1 (1.5)	1 (1.5)	1 (0.9)	2 (1.8)
Abnormal liver function	1 (2.1)	2 (4.3)	0	0	1 (0.9)	2 (1.8)
Decreased RBC	0	1 (2.1)	1 (1.5)	1 (1.5)	1 (0.9)	2 (1.8)
Hyperglycemia	0	1 (2.1)	1 (1.5)	1 (1.5)	1 (0.9)	2 (1.8)
Macula	0	0	1 (1.5)	1 (1.5)	1 (0.9)	1 (0.9)
Pulmonary hypertension	1 (2.1)	1 (2.1)	0	0	1 (0.9)	1 (0.9)
Acute kidney injury	0	0	1 (1.5)	1 (1.5)	1 (0.9)	1 (0.9)
Immune-related enteritis	0	0	1 (1.5)	1 (1.5)	1 (0.9)	1 (0.9)
Immune-related gastritis	0	0	1 (1.5)	1 (1.5)	1 (0.9)	1 (0.9)
Encephalitis	1 (2.1)	1 (2.1)	0	0	1 (0.9)	1 (0.9)
Urinary tract infection	0	0	1 (1.5)	1 (1.5)	1 (0.9)	1 (0.9)
Hydronephrosis	0	0	1 (1.5)	1 (1.5)	1 (0.9)	1 (0.9)
Ureteral stricture	0	0	1 (1.5)	1 (1.5)	1 (0.9)	1 (0.9)
Immune-related hepatitis	0	0	1 (1.5)	1 (1.5)	1 (0.9)	1 (0.9)
Immune-related endocrine abnormalities	1 (2.1)	1 (2.1)	0	0	1 (0.9)	1 (0.9)
Hypoadrenalism	0	0	1 (1.5)	1 (1.5)	1 (0.9)	1 (0.9)

TRAEs of any grade occurred in 108 patients (95.6%), and TRAEs ≥grade 3 were observed in 35 patients (31.0%). The most common TRAEs affecting more than 30% of the patients were infusion reactions (n = 44, 38.9%), anemia (n = 42, 37.2%), elevated AST (n = 35, 31.0%) and diarrhea (n = 34, 30.1%). Anemia (n = 6, 5.3%), elevated AST (n = 4, 3.5%) and elevated alanine aminotransferase (ALT) (n = 3, 2.7%) were commonly observed as ≥grade 3 TRAEs (occurring >2 patients). There were 74 patients (65.5%) who experienced any grade immune-related adverse events (irAEs), and 14 patients (12.4%) who experienced ≥ grade 3 irAEs. The most commonly observed irAEs (≥15%) included diarrhea (n = 18, 15.9%), hypothyroidism (n = 17, 15.0%), and rash (n = 17, 15.0%). Treatment-related serious adverse events (TRSAEs) were observed in 25 patients (22.1%). Eleven (9.7%) and 20 (17.7%) patients permanently discontinued the KN026 and KN046 infusions, respectively, for TRAEs. Two patients (1.8%) died from TRAEs including drug-related PAH (n = 1) and interstitial pneumonia (n = 1).

## Discussion

This article is a combined analysis of a phase Ib and a phase II study aimed at assessing KN026 plus KN046 in HER2-positive solid tumors. A phase Ib study was initiated as a pivotal study to assess the safety and efficacy of this regimen. We observed encouraged efficacy, especially in HER2-positive GC as the 1st-line treatment, with 6 of the 7 patients achieving a PR. After discussion with the DSMC, the phase Ib study was terminated and a phase II study was conducted to further confirm the efficacy. A combined analysis of 2 sequential studies with adaptive designs was needed to improve the estimation precision of both efficacy and safety. We have focused mainly on the exploration of a chemotherapy-free mode for treating HER2-positive solid tumors, even as 1st-line treatment. To the best of our knowledge, only a limited number of similar studies have been reported thus far. The ORR of this regimen in HER2-positive GC patients as the 1st-line treatment was 78.9% (95%CI, 62.7%, 90.4%). The median DoR was 13.4 months (95%CI, 8.3, NE), the median PFS was 11.0 months (95%CI, 5.5, 16.5) and the median OS not reached.

In a phase Ib study, patients with HER2-positive cancer were identified as the target population for further exploration. This study mainly explored the antitumor activity of KN026 plus KN046 in HER2-positive GC. In addition, the efficacy decreased with increasing numbers of prior treatment lines. For sustained HER2-directed therapies, the loss of HER2 expression and the development of enriched genetic aberrations in downstream pathways as a compensatory mechanism for HER2 antibodies could contribute to the reduced efficacy of this regimen in later-line treatment.^28^ Early exposure to KN026 and KN046 might confer more benefits. Hence, this regimen was further explored as a 1st-line treatment for HER2-positive GC. Among the 38 patients evaluated for treatment efficacy, 30 achieved an objective response, including 1 patient who achieved CR and 29 patients who achieved PR. Only 3 patients experienced PD after the first efficacy assessment. This finding indicated the potential of KN026 plus KN046 in a chemotherapy-free model for 1st-line treatment of HER2-positive GC, which compared relatively favorably with the reported AIO INTEGA and MAHOGANY studies that evaluated the combination of HER2, PD-1/PD-L1 and CTLA4 blockade in the treatment of naïve HER2-positive GC.^29,30^ Although, the phase Ib and II studies were single-arm studies, the efficacy and survival rates were comparable to those reported in KEYNOTE-811.^7^ A phase III study is warranted to further investigate this regimen in a treatment-naïve HER2-positive patient population. KN026 plus KN046 also contributed to an ORR of 44.4% in patients receiving ≥ 2nd-line treatment. This efficacy is relatively greater than that reported in the CP-MGAH22-05 study, which evaluated margetuximab plus pembrolizumab in pretreated HER2-positive GC.^31^ For the 2nd-line treatment of HER2-positive GC, the options for further anti-HER2 therapies were limited until several anti-HER2 ADCs developed. Trastuzumab deruxtecan and Disitamab vedotin plus toripalimab (PD-1 blockade) have shown encouraging antitumor activity as 2nd-line treatments for HER2-positive GC.^32,33^ This chemotherapy-free regimen showed comparable efficacy and a lower incidence of hematological toxicity. In this study, over half (55.6%) of the evaluable HER2-positive GC had prior anti-HER2 therapies. This might be attributed to selection bias due to the limited sample size, patients suffering disease progression within 6 months after finishing adjuvant therapy, and the limited economic condition of patients. And HER2-positive GC patients who had received prior anti-HER2 therapies could still benefit from this regimen, achieving an ORR of 53.3%. The relationship between treatment efficacy and PD-L1 status was explored in GC cohorts. On the basis of the study protocol, the expression status of PD-L1 was not mandatory collected at the time of patient enrollment. Therefore, only nearly 60% of the patients completed the PD-L1 expression test. Approximately 60% of patients were PD-L1 positive, which is comparable with reported studies.^27,29,31^ PD-L1 positivity was also identified as a potential predictor of survival benefit with this regimen for patients with GC. These findings align with several reported studies.^7^ The relationship between efficacy and PD-L1 status should be confirmed in further phase III studies. Moreover, KN026 plus KN046 has been explored in other HER2-positive solid tumors, including CRC, lung cancer, gallbladder cancer, and other solid tumors. In the CRC subgroup, KN026 plus KN046 showed relatively greater efficacy than did a latter-line treatment (9.7%–45.3%).^34^ The median PFS of 5.6 months suggests that this regimen is associated with a meaningful improvement in survival. In other non-CRC solid tumors subgroup, KN026 plus KN046 also had a relatively higher ORR than that reported in previous studies.^35,36^ The results highlight the efficacy of HER2-targeted therapies as an effective targeted treatment options for patients with HER2-positive solid tumors.

The spectrum and incidence of AEs were similar to those observed with KN026 or KN046 monotherapy and other HER2 ECD2/4 antibodies or immunotherapies.^23–26,37,38^ Anemia was the most common form of hematological toxicity, with an incidence of 37.2%. Given that anemia is a common symptom of GI tumors, this result might be affected to a certain extent. Other hematological toxicities, such as thrombocytopenia and neutropenia, were infrequently observed. The incidence of diarrhea, which significantly affects the treatment tolerance in patients with GI tumors, was significantly lower than that reported in the reported JACOB study of pertuzumab, trastuzumab, and chemotherapy in HER2-positive GC patients (30.1% vs. 48%).^20^ Although diarrhea, rash, and thyroid function disorders were the most common irAEs, all were manageable and resolved with appropriate treatment. Moreover, the risk of ≥grade 3 TRAEs in KN026 plus KN046 was significantly lower than that reported in the phase Ib/II studies of ZW25 plus tislelizumab and chemotherapy, the KEYNOTE-811 study, and current standard 2nd-line chemotherapies.^7,8,39^ Notably, the risk of infusion-related reactions was substantially increased with the combination of KN026 and KN046, with an incidence of 38.9% compared with 12.7% (KN026) or 13.8% (KN046) alone, necessitating premedication administration. It might also contribute to more common infusion interruptions and discontinuations. Any grade immune-related AEs affecting important organs, such as interstitial lung disease (4.4%), myocarditis (2.7%), and encephalitis (0.9%), were infrequently observed and were mostly relieved after corticosteroid administration and supportive care. One patient with grade 5 immune-related interstitial lung disease died despite receiving high-dose corticosteroid infusion. Additionally, one patient who received Dose 1 died from PAH 1.6 months after the first infusion. The patient developed progressive dyspnea and was subsequently diagnosed with PAH. Acute pulmonary arterial embolism, venous embolism, pulmonary infection, and other cardiac vascular disorders can be excluded by computed tomography (CT), computed tomography pulmonary angiography (CTPA) scan, venous ultrasound, and blood examination. This patient died shortly after completing the treatment. A literature review revealed that several case reports and reviews have associated anti-HER2 therapies with PAH.^40–42^ Anti-HER2 therapies might induce hemorrhagic telangiectasia, disrupt cytoskeletal microtubules, and lead to apoptosis of potentially HER2‐expressing endothelial cells, potentially resulting in PAH. Moreover, immunotherapy-related PAH has rarely been reported.^27,43^ Therefore, following discussion with the DSMC, PAH was prioritized as a concern associated with KN026. However, this was an insolate case, and the definitive cause could not be conclusively determined, as the patient’s family declined an autopsy. As a serious and rare TRAE, PAH should receive increased attention in future studies. Even though some patients were dropped out for serious TRAEs after a short-term drug infusion, they could still have survival benefit. For example, one patient with HER2-positive GC who received this regimen as the first-line treatment suffered grade 4 encephalitis after 2 drugs infusion doses. This patient recovered after high-dose corticosteroid infusion and supportive treatment. Although this patient permanently discontinued this regimen for the intolerable TRAE, this patient achieved a PR for more than 30 months until the last follow-up.

This combined analysis has several potential limitations. In these 2 studies, HER2 status was re-assessed in a central lab, retrospectively. The consistency of HER2 status between the local and the central lab was 84.2%, which might due to differences among labs, the degradation of HER2 protein in the specimen, and the heterogeneity of tumors.^44^ Primarily being phase Ib and II and single-arm studies have limited sample sizes, which can lead to unavoidable bias in patient selection. To further explore the efficacy and survival outcomes of this regimen, especially as a 1st-line treatment for HER2-positive GC, a phase III study is warranted.

In conclusion, the KN026 in combination with KN046 showed acceptable safety profiles and promising efficacy in HER2-positive nonbreast cancer, even as a 1st-line treatment for GC.

## Materials and methods

### Study design and patients

The phase Ib study is an open-label, multicenter, single-arm, investigator-initiated trial aimed to explore the safety, tolerability, and primary efficacy of KN026 plus KN046. The phase Ib study was conducted in 2 centers in China from Sep. 2019 (ClinicalTrials. gov, NCT04040699). This phase II study is an open-label, multicenter, single-arm, industry-sponsored trial aimed to explore the efficacy of KN026 plus KN046 in patients with HER2-positive solid tumors. The phase II study was conducted across 14 centers in China from Nov. 2020 (ClinicalTrials. gov, NCT04521179). Here, we present the combined analysis of a phase Ib and a phase II study of KN026 plus KN046 in patients with HER2-positive solid tumors for almost identical inclusive criteria. Since the data from the breast cancer (BC) cohort of the phase II study are not yet mature, the results of KN026 plus KN046 in HER2-positive breast cancer will be reported separately at a later date.

Patients were eligible for if they were between the ages of 18 and 75 years and had histologically HER2-positive and other HER2 alteration (HER2 low-expression and mutation, only permitted in protocol V1.0-2.0 of the phase Ib study) that were locally advanced, or metastasized. Patients progressed on ≥1 prior systemic therapy or for which no standard therapies were available. Treatment-naïve HER2-positive GC/GEJ were also allowed. Patients had ≥1 measurable lesion according to the Response Evaluation Criteria in Solid Tumors (RECIST) version 1.1, with an Eastern Cooperative Oncology Group performance status of 0 or 1, adequate organ function, and an expected survival time over 3 months. Please refer to the study protocols for additional key inclusion and exclusion criteria.

All eligible patients in both phase Ib and phase II studies provided written informed consent. All versions of protocols were approved by the ethics committee of each center. Both phase Ib and phase II studies were performed in accordance with the Declaration of Helsinki and Good Clinical Practice guidelines.

### Procedures

The phase Ib study included a dose exploration part and dose expansion part. In dose exploration part, a “3 + 3” design was used. Based on the previous studies of KN026 and KN046 monotherapies, the RP2Ds of KN026 monotherapy were determined to be 20 mg/kg Q2w and 30 mg/kg Q3w.^23^ While, KN046 is recommended as 5 mg/kg Q2w and has been shown to have antitumor effects at 3 mg/kg Q2w and 5 mg/kg Q3w as monotherapy.^25^ Thus, four predefined combination doses of KN026 with KN046, including 20 mg/kg Q2w/3 mg/kg Q2w (Dose 1), 20 mg/kg Q2w/5 mg/kg Q3w (Dose 2), 30 mg/kg Q3w/5 mg/kg Q3w (Dose 3), and 20 mg/kg Q2w/5 mg/kg Q2w (Dose 4), were used for dose and safety exploration. Dose expansion can be initiated once safety was confirmed at a specific dose level with the approval of the DSMC. The number of patients in the dose expansion part was planned to include ~20 patients in Dose 1 and 3, while ~12 patients in Dose 4.

In the phase II study, the combined dose of KN026 plus KN046 was selected based on the PK/pharmacodynamics (PD) analyses in phase I studies of KN026 and KN046 monotherapy. In the phase I study of KN026 monotherapy, 30 mg/kg Q3w was chosen as the RP2D.^23^ Based on the PK/PD analysis of KN046 monotherapy, the KN046 concentration under more than 75% dosing interval at 5 mg/kg Q3W is above the target trough concentration at each cycle.^25^ Thus, 5 mg/kg Q3W is suitable for KN046 combination therapy. Finally, the combination dose of KN026 and KN046 was predefined as 30 mg/kg Q3w/5 mg/kg Q3w in the phase 2 study, if the safety could be confirmed in phase Ib study. There were 80-122 patients planned to be enrolled, including 3 HER2-positive tumor cohorts (GC, breast cancer, other tumors). In the GC cohort, 30 to 60 patients were planned to enrolled. In the BC cohort, 30 to 36 patients were planned to enrolled. And in other tumors cohorts, 20 to 26 patients were planned to be included. The results of BC cohort would be reported in an isolated article. The sample size calculation is based on the estimate of the 95% CI for the ORR using the Clopper-Pearson method (Supplementary Materials).

All patients were intravenously infused with both KN026 and KN046. At the doses of 2 and 3, a loading dose of KN026 was added on day 8 of cycle 1 to rapidly reach the peak concentration and achieve optimal antitumor activity.^21^ KN026 was first infused for 90 min. If tolerated, the infusion was shortened to 60 min for all subsequent administrations. KN046 was infused for 90–120 min. Dose delays were allowed for the management of AEs. Dose modifications were not allowed. The treatment could be continued until the clinical or imaging disease progressed, unacceptable toxicity developed, other antitumor treatments were initiated, infusion was refused, withdrawal of consent, or the end of the study, whichever occurred first.

### Endpoints

In the phase Ib study, the primary objective was to determine the MTD and RP2D during the dose exploration part and to assess the preliminary efficacy during the dose expansion part. The secondary endpoints included safety, tolerance, and the effect of HER2 status on efficacy. Exploratory endpoints are aimed to assessing the relationship between biomarkers (PD-L1 status, etc) and efficacy. In the phase II study, the primary endpoint was to assess the ORR and DoR. The secondary endpoints included PFS, OS, 6-month/12-month progression-free rate, clinical benefit (CR + PR + SD ≥ 24 weeks) rate (CBR), safety, tolerance, pharmacokinetics, immunogenicity, the effect of HER2 status and drug exposure on efficacy. Exploratory endpoints included the relationship between biomarkers (PD-L1 status, etc) and antitumor activity (ORR, etc.).

### Safety assessment

Safety assessment was performed across the entire study. All adverse events (AEs) were recorded and categorized on the basis of severity (NCI-CTCAE V.5.0) and their relationship with KN026 or KN046. The TRAEs included all AEs that were probably and definitely related to the study drugs. After completion of treatment, each subject will be followed up for adverse events (AEs) for 30 days and 90 days thereafter.

The DLT evaluation included all patients who received at least 80% of the planned dose during the dose exploration part of phase Ib study. A DLT was defined as a TRAE occurring within the 21 days (Q3w) or 28 days (Q2w) after the administration of the first dose that met the predefined criteria based on grading per NCI-CTCAE V.5.0: grade ≥ 2 central nervous system toxicity and any grade ≥ 3 non-hematological toxicities (except for alopecia, transient grade 3 fatigue, local reactions, headache, nausea, vomiting, infusion reaction, fever, flu-like symptoms, tumor flare and single abnormality in the laboratory test), grade ≥ 3 agranulocytosis (≥7 days), febrile neutropenia, grade ≥ 3 thrombocytopenia with a bleeding tendency or requiring platelet transfusion; other grades ≥ 4 hematologic toxicities. The specific definitions of DLT are provided in Supplementary Materials, Part 2.

### Efficacy assessment

The clinical response was evaluated by investigators based on RECIST version 1.1 at baseline and every 6 (Q3w) or 8(Q2w) weeks (±7 days) during 48 weeks after the first infusion, and then every 12 weeks (±7 days) thereafter until disease progression, the start of new antitumor therapies, withdrawal of consent, loss to follow-up or study completion, whichever occurred first.

### HER2 and PD-L1 statuses

The HER2 status from local laboratories was collected. HER2 immunohistochemistry test was done using an FDA-approved HercepTest (Dako, Denmark). For fluorescence in situ hybridization (FISH), Dako HER2 IQFISH pharmDX was used. A next-generation sequencing was also used in HER2 test. All HER2 status of the tumors was confirmed by certified pathologists. HER2 positivity was defined as HER2 3+ by IHC or 2+ by IHC with FISH amplification (HER2:CEP17 ratio > 2 or HER2 gene copy number > 6) or NGS HER2 copy number > 6 based on the guidelines for HER2 detection^45,46^; HER2 mutations included HER2 exon 20 insertion, HER2 deletion, and other nonsynonymous activating mutations reported in the COSMIC database.

The PD-L1 status from local laboratories was collected, if possible, but was not mandatory. IHC was used to examine PD-L1 expression with a PD-L1 (22C3) assay. PD-L1 status was recorded by the combined positive score (CPS). A CPS = 1 was used as the cutoff value to define PD-L1 positivity (CPS ≥ 1) and negativity (CPS < 1).

### Statistical analysis

All the statistical analyses were performed using SAS version 9.4 or GraphPad Prism software. Analyses on safety and adverse events were performed descriptively. The ORR and CBR were estimated, and the 95% CIs were calculated with the Clopper-Pearson method. The Kaplan–Meier method was used to estimate the median DoR in responders, PFS, OS and TTR, and survival curves. The Brookmeyer-Crowley method was used to calculate the 95%CIs for the medians, and Greenwood’s formula was used to calculate the 95%CIs for the progression-free rates at 6 and 12 months. Post-hoc biomarker analyses were performed to examine potential associations between HER2 and PD-L1 expression.

## Supplementary information

Supplementary Materials

The protocol of the Phase Ib study

The main protocol revision of the phase Ib study from V2.0 to V6.1

The protocol of the Phase II study

The table of sampling volume and ORR

## Data Availability

The protocol and all data supporting the study’s findings are available in the manuscript and Supplementary Materials. All requests for further data generated or analyzed during this study will be considered by the leading clinical center, Department of Gastrointestinal Oncology, Beijing Cancer Hospital, and the study collaborator, Jiangsu Alphamab Biopharmaceuticals Co., Ltd, to verify whether the request is subject to any intellectual property or confidentiality obligations. Data may be requested 24 months after study completion. Qualified researchers should submit a proposal to the corresponding author outlining the reasons for requiring the data. If the proposal is approved the sponsor will provide the data if the requestor signs a data-access agreement. The use of data must comply with the requirements of the Human Genetics Resources Administration of China and other country or region-specific regulations.
