# Supplementary material for: Efficacy and safety of KN026, a bispecific anti-HER2 antibody, in combination with KN046, an anti-CTLA4/PD-L1 antibody, in patients with advanced HER2-positive nonbreast cancer: a combined analysis of a phase Ib and a phase II study
Source: Signal Transduct Target Ther. 2025 Mar 19;10:104. doi: 10.1038/s41392-025-02195-x (PMC11923254; doi:10.1038/s41392-025-02195-x)
Supplement: Supplementary file 1 — Supplementary Materials [file 41392_2025_2195_MOESM1_ESM.docx]

Supplementary Materials for

Efficacy and safety of KN026, a bispecific anti-HER2 antibody, in combination with KN046, an anti-CTLA4/PD-L1 antibody, in patients with advanced HER2-positive nonbreast cancer: a combined analysis of a phase Ib and a phase II Study

Dan Liu, Jifang Gong, Jian Li, Changsong Qi, Zuoxing Niu, Bo Liu, Zhi Peng, Suxia Luo, Xicheng Wang, Yakun Wang, Rusen Zhao, Lilin Chen, Ting Deng, Zhen Li, Lei Chen, Meimei Fang, Hongwei Yang, Linzhi Lu, Yanming Zhang, Fengling Kang, Ting Xu, Xiaotian Zhang, Lin Shen

Correspondence to: zhangxiaotian@bjmu.edu.cn; shenlin@bjmu.edu.cn;

**This PDF file includes:**

Supplementary figures 1 to 2

The definition of DLTs

Supplementary tables 1 to 6

**Other Supplementary Materials for this manuscript include the following:**

The protocol of the phase Ib study [KN046-IST-02 protocol version 2.0. pdf]

The main protocol revision of the phase Ib study from V2.0 to V6.1 [Main protocol revision of KN046-IST-02 from V2.0 to 6.1.pdf]

The protocol of the phase II study [KN026-203 protocol version 4.2. pdf]

The table of sampling volume and ORR [Table of sampling volume and ORR.pdf]

**Part 1. Supplementary figures**

**Figure 1.
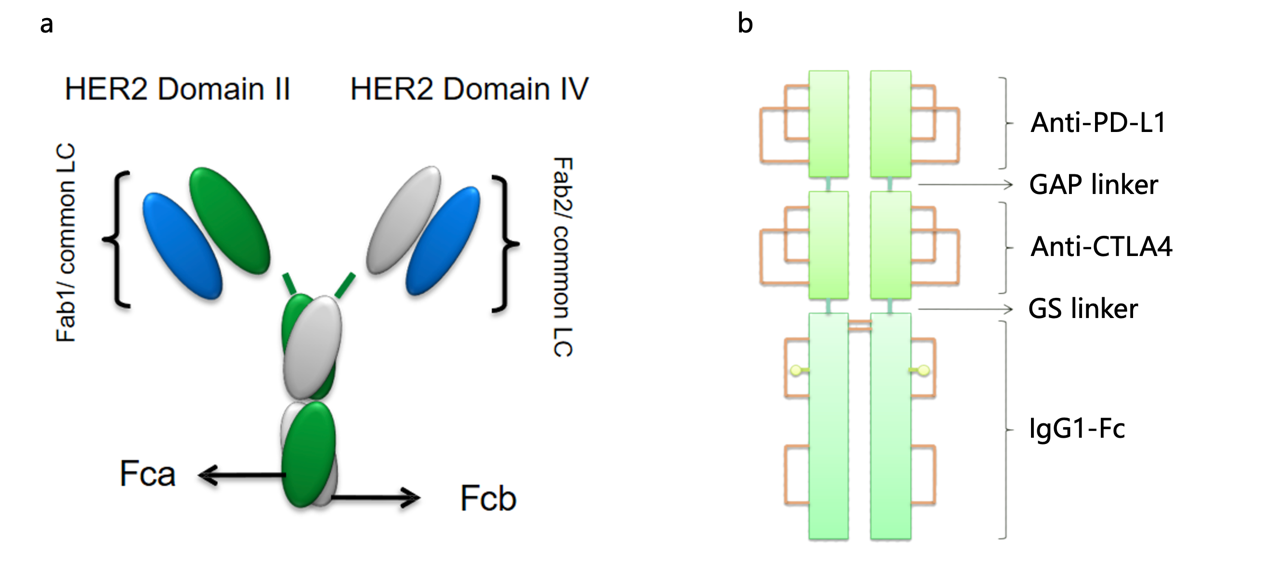
**

**Figure 2.**

a


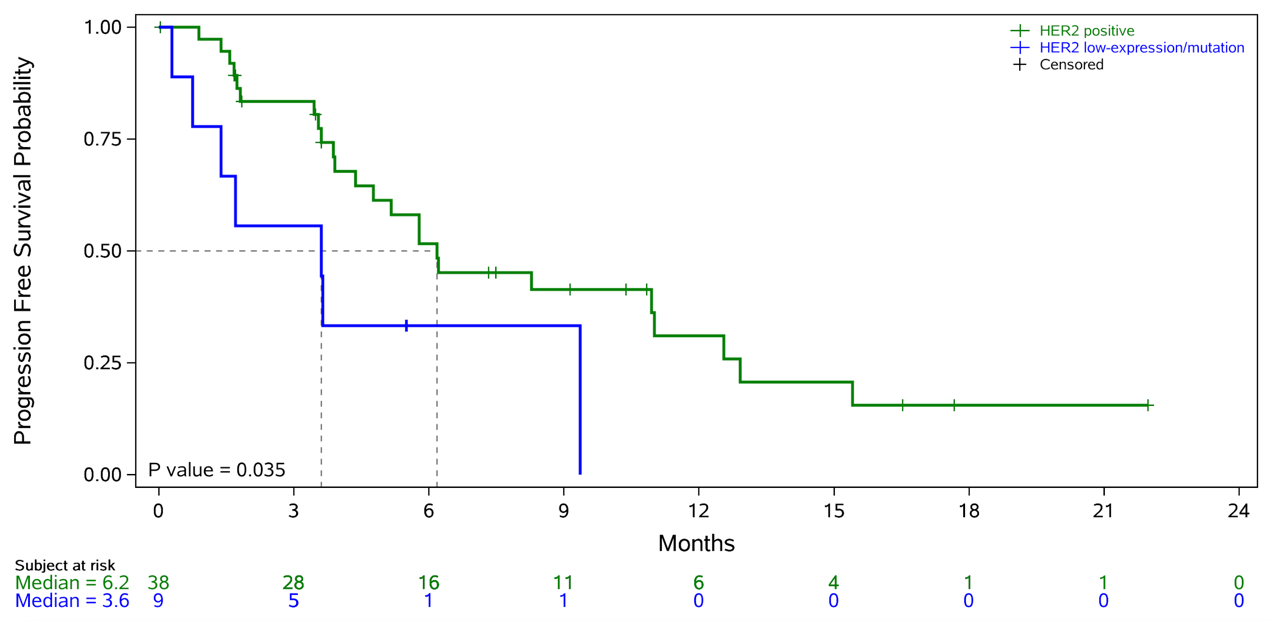


b
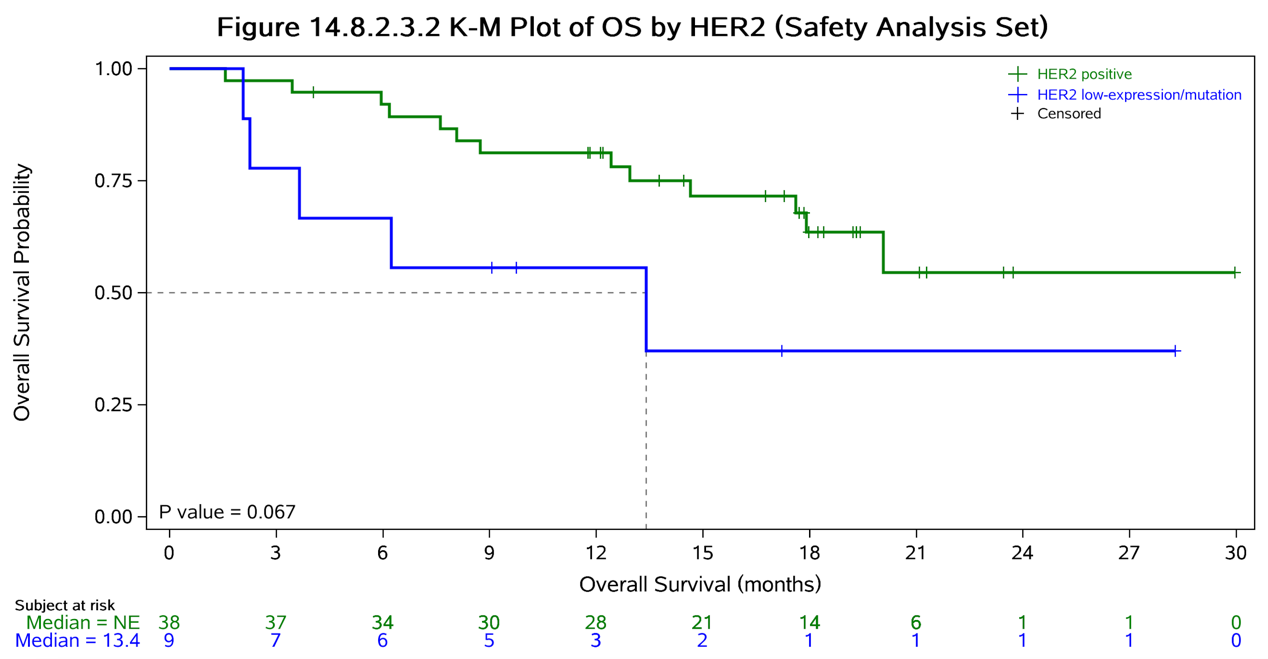


**Figure Legends for supplementary figures:**

**Figure 1. The diagrams for the structure of KN026 (a) and KN046 (b).**

**Figure 2. The survival curves of enrollment patients stratified by HER2 expression in the phase Ib study.**

The PFS (a) and OS (b) of enrollment patients stratified by HER2 expression

**Part 2. Definition of DLTs**

DLT in the phase Ib study is defined as AEs which are related to treatment with KN026 and/or KN046 and meet the following criteria:

(1) Hematological toxicity:

a. ≥ Grade 3 agranulocytosis lasting more than 7 days;

b. Febrile neutrophil decreased;

c. ≥ Grade 3 thrombocytopenia with bleeding tendency or requiring platelet transfusion;

d. ≥ Grade 4 hematologic toxicity, except for Grade 4 lymphopenia (alone).

(2) Non-hematological toxicity:

a. Drug-related symptomatic heart failure (NYHA Grade 3 or higher); or ≥ 15% absolute decrease in LVEF from baseline;

b. ≥ Grade 3 raised serum creatinine;

c. ≥ Grade 3 total bilirubin increased;

d. Grade 3 ALT and/or AST increased lasting more than 7 days;

e. Grade 4 ALT and/or AST increased;

f. ≥ Grade 2 central nervous system toxicity;

g. ≥ Grade 3 cardiotoxicity;

h. Grade ≥ 3 asymptomatic serum pancreatic amylase or lipase increased lasting more than 14 days;

i. Serum pancreatic amylase or lipase increased accompanied by clinical symptoms and signs and requiring medical intervention;

j. Other ≥ Grade 3 nonhematologic toxicities, except:

- - - Any grade of alopecia;
    - Transient (≤ 24 hours) Grade 3 fatigue, local reactions, headache, nausea, and vomiting that resolved to ≤ Grade 1;
    - Grade 3 diarrhea, Grade 3 skin toxicity, or Grade 3 liver function-related parameter increased (ALT, AST, or GGT) that resolve to ≤ Grade 1 within 7 days after medical intervention;
    - Grade 3 infusion-related reactions that resolved within 6 hours of medical intervention;
    - Grade 3 flu-like symptoms or fever that are transient (≤ 6 hours) after medical intervention;
    - A single abnormality in the laboratory test that is not clinically significant and resolves to ≤ Grade 1 within 7 days of appropriate medical intervention;
    - Tumor recurrence, manifested with pain, local irritation, or local rash at the site of a known or suspected tumor.

**Part 3. Supplementary tables**

**Table 1. Any grade treatment-related adverse events (TRAEs) in dose exploration part of the phase Ib study**

|  | Dose 1  N = 3 (%) | | Dose 2  N =3(%) | | | Dose 3  N =4(%) | | | Dose 4  N =3 (%) | | Total  N = 13 (%) | |
| --- | --- | --- | --- | --- | --- | --- | --- | --- | --- | --- | --- | --- |
|  | Grade ≥ 3 | All Grades | Grade ≥ 3 | | All Grades | | Grade ≥ 3 | All Grades | Grade ≥ 3 | All Grades | Grade ≥ 3 | All Grades |
| All TRAEs | 0 | 3 (100.0) | 1 (33.3) | 3 (100.0) | | 0 | | 4 (100.0) | 0 | 3 (100.0) | 1 (7.7) | 13 (100.0) |
| SAE | 0 | 0 | 0 | 1(33.3) | | 0 | | 0 | 0 | 1(33.3) | 0 | 2(15.4) |
| Infusion-related reactions | 0 | 0 | 0 (0.0) | 1 (33.3) | | 0 | | 3 (75.0) | 0 | 0 | 0 | 4 (30.8) |
| Anemia | 0 | 1(33.3) | 1 (33.3) | 2 | | 0 | | 1(25.0) | 0 | 0 | 1 (7.7) | 4 (30.8) |
| Diarrhea | 0 | 2(66.7) | 0 | 0 | | 0 | | 1(25.0) | 0 | 1 (33.3) | 0 | 4 (30.8) |
| Elevated AST | 0 | 1(33.3) | 0 | 1 (33.3) | | 0 | | 2(50.0) | 0 | 0 | 0 | 4 (30.8) |
| Other thyroid dysfunction | 0 | 1(33.3) | 0 | 0 | | 0 | | 0 | 0 | 2 | 0 | 3(23.1) |
| Rash | 0 | 2(66.7) | 0 | 1 (33.3) | | 0 | | 0 | 0 | 0 | 0 | 3(23.1) |
| Elevated bilirubin | 0 | 1(33.3) | 0 | 1 (33.3) | | 0 | | 1(25.0) | 0 | 0 | 0 | 3(23.1) |
| Leukopenia | 0 | 1(33.3) | 0 | 1 (33.3) | | 0 | | 0 | 0 | 0 | 0 | 2(15.4) |
| Elevated ALT | 0 | 0 | 0 | 0 | | 0 | | 1(25.0) | 0 | 1 (33.3) | 0 | 2(15.4) |
| Hyponatremia | 0 | 0 | 0 | 1 (33.3) | | 0 | | 1(25.0) | 0 | 0 | 0 | 2(15.4) |
| Neutropenia | 0 | 1 (33.3) | 0 | 1 (33.3) | | 0 | | 0 | 0 | 0 | 0 | 2(15.4) |
| Hypothyroidism | 0 | 1 (33.3) | 0 | 0 | | 0 | | 1(25.0) | 0 | 0 | 0 | 2(15.4) |
| Interstitial lung disease | 0 | 1 (33.3) | 0 | 1 (33.3) | | 0 | | 0 | 0 | 0 | 0 | 2(15.4) |
| Hypokalemia | 0 | 0 | 0 | 0 | | 0 | | 1(25.0) | 0 | 0 | 0 | 1 (7.7) |
| Myositis | 0 | 0 | 0 | 0 | | 0 | | 0 | 0 | 1 (33.3) | 0 | 1 (7.7) |
| Elevated urinary WBC | 0 | 1 (33.3) | 0 | 0 | | 0 | | 0 | 0 | 0 | 0 | 1 (7.7) |
| Proteinuria | 0 | 1 (33.3) | 0 | 0 | | 0 | | 0 | 0 | 0 | 0 | 1 (7.7) |
| Elevated urinary RBC | 0 | 1 (33.3) | 0 | 0 | | 0 | | 0 | 0 | 0 | 0 | 1 (7.7) |
| Vomiting | 0 | 1 (33.3) | 0 | 0 | | 0 | | 0 | 0 | 0 | 0 | 1 (7.7) |
| Elevated TSH | 0 | 1 (33.3) | 0 | 0 | | 0 | | 0 | 0 | 0 | 0 | 1 (7.7) |
| Elevated Scr | 0 | 0 | 0 | 0 | | 0 | | 0 | 0 | 1 (33.3) | 0 | 1 (7.7) |
| Thrombocytopenia | 0 | 1 (33.3) | 0 | 0 | | 0 | | 0 | 0 | 0 | 0 | 1 (7.7) |

Note: Analysis was performed with the safety analysis population, which included all patients who received ≥1 dose of combined therapy. Patients may have experienced ≥1 event per adverse event, but it was only counted once at the worst Common Terminology Criteria for Adverse Events grade.

**Table 2. Any grade treatment-related adverse events (TRAEs) occurring in ≥5% of patients and all grade ≥ 3 TRAEs in dose expansion part of the phase Ib study**

|  | Dose 1  N = 22 (%) | | | | Dose 2  N =0(%) | | | | | | | Dose 3  N =10 (%) | | | | | | | Dose 4  N =2 (%) | | | | | | Total  N = 34 (%) | | | | |
| --- | --- | --- | --- | --- | --- | --- | --- | --- | --- | --- | --- | --- | --- | --- | --- | --- | --- | --- | --- | --- | --- | --- | --- | --- | --- | --- | --- | --- | --- |
|  | Grade ≥ 3 | | All Grades | | Grade ≥ 3 | | | | All Grades | | | | Grade ≥ 3 | | | | All Grades | | Grade ≥ 3 | | | All Grades | | | Grade ≥ 3 | | | All Grades | |
| All TRAEs | 6(27.2) | | 22 (100.0) | | 0 | | | 0 | | | | 3 (30.0) | | | | | 8 (80.0) | | 0 | | | 1 (50.0) | | | 9 (26.5) | | | 31 (91.2) | |
| SAE | 4(18.2) | | 5(22.7) | | 0 | | | 0 | | | | 0 | | | | | 2(20.0) | | 0 | | | 0 | | | 4(11.8) | | | 7(20.6) | |
| Anemia | 1(4.5) | | 13(59.1) | | 0 | | | 0 | | | | 0 | | | | | 4(40.0) | | 0 | | | 1(50.0) | | | 1(2.9) | | | 18(52.9) | |
| Infusion-related reactions | 1(4.5) | | 9(40.9) | | 0 | | | 0 | | | | 0 | | | | | 4 (40.0) | | 0 | | | 1 (50.0) | | | 1(2.9) | | | 13 (38.2) | |
| Elevated AST | 0 | | 6(27.3) | | 0 | | | 0 | | | | 0 | | | | | 5(50.0) | | 0 | | | 0 | | | 0 | | | 11(32.4) | |
| Elevated ALT | 0 | | 6(27.3) | | 0 | | | 0 | | | | 1 | | | | | 4(40.0) | | 0 | | | 0 | | | 1(2.9) | | | 10(29.4) | |
| Diarrhea | 0 | | 4(18.2) | | 0 | | | 0 | | | | 0 | | | | | 5(50.0) | | 0 | | | 0 | | | 0 | | | 9(26.5) | |
| Rash | 0 | | 6(27.3) | | 0 | | | 0 | | | | 0 | | | | | 2(20.0) | | 0 | | | 0 | | | 0 | | | 8(23.5) | |
| Leukopenia | 0 | | 5(22.7) | | 0 | | | 0 | | | | 0 | | | | | 2(20.0) | | 0 | | | 0 | | | 0 | | | 7(20.6) | |
| Thrombocytopenia | 1(4.5) | | 5(22.7) | | 0 | | | 0 | | | | 0 | | | | | 2(20.0) | | 0 | | | 0 | | | 1(2.9) | | | 7(20.6) | |
| Elevated bilirubin | 0 | | 4(18.2) | | 0 | | | 0 | | | | 0 | | | | | 2(10.0) | | 0 | | | 0 | | | 0 | | | 6(17.6) | |
| Neutropenia | 1(4.5) | | 4(18.2) | | 0 | | | 0 | | | | 0 | | | | | 1(10.0) | | 0 | | | 0 | | | 1(2.9) | | | 5(14.7) | |
| Hypothyroidism | 0 (0.0) | | 3 (13.6) | | 0 | | | 0 | | | | 0 | | | | | 1(10.0) | | 0 | | | 0 | | | 0 | | | 4 (11.8) | |
| Proteinuria | 0 | | 2 (9.1) | | 0 | | | 0 | | | | 0 | | | | | 2(20.0) | | 0 | | | 0 | | | 0 | | | 4(11.8) | |
| Hyperthyroidism | 0 | | 2(9.1) | | 0 | | | 0 | | | | 0 | | | | | 0 | | 0 | | | 1(50.0) | | | 0 | | | 3(8.8) | |
| Elevated GGT | 0 | | 0 | | 0 | | | 0 | | | | 1(10.0) | | | | | 2(20.0) | | 0 | | | 0 | | | 1(2.9) | | | 2(5.9) | |
| Hypokalemia | 0 | | 2 (9.1) | | 0 | | | 0 | | | | 0 | | | | | 0 | | 0 | | | 0 | | | 0 | | | 2(5.9) | |
| Liver dysfunction | 1(4.5) | | 2 (9.1) | | 0 | | | 0 | | | | 0 | | | | | 0 | | 0 | | | 0 | | | 0 | | | 2(5.9) | |
| Elevated cTnI | 0 | | 2 (9.1) | | 0 | | | 0 | | | | 0 | | | | | 0 | | 0 | | | 0 | | | 0 | | | 2(5.9) | |
| Weight loss | 0 | | 0 | | 0 | | | 0 | | | | 0 | | | | | 2(20.0) | | 0 | | | 0 | | | 0 | | | 2(5.9) | |
| Elevated TSH | 0 | | 2 (9.1) | | 0 | | | 0 | | | | 0 | | | | | 0 | | 0 | | | 0 | | | 0 | | | 2(5.9) | |
| Elevated ALP | 0 | | 0 | | 0 | | | 0 | | | | 0 | | | | | 2(20.0) | | 0 | | | 0 | | | 0 | | | 2(5.9) | |
| Elevated LDH | 0 | | 0 | | 0 | | | 0 | | | | 0 | | | | | 2(20.0) | | 0 | | | 0 | | | 0 | | | 2(5.9) | |
| Endocrine abnormal | | 1(4.5) | | 1 (4.5) | | | 0 | | | 0 | | | | 0 | | 0 | | | | | 0 | | | 0 | | | 1(2.9) | | 1(2.9) |
| Pruritus | 0 | | | 0 | 0 | | | 0 | | | | 1(10.0) | | | | | 1(10.0) | | 0 | | | 0 | | | 1(2.9) | | | 1(2.9) | |
| Encephalitis | | 1 (4.5) | | 1 (4.5) | | 0 | | | | | 0 | | | | 0 | | | 0 | | 0 | | | 0 | | | 1(2.9) | | 1(2.9) | |
| Pulmonary arterial hypertension | | 1 (4.5) | | 1 (4.5) | | 0 | | | | | 0 | | | | 0 | | | 0 | | 0 | | | 0 | | | 1(2.9) | | 1(2.9) | |

Note: Analysis was performed with the safety analysis population, which included all patients who received ≥1 dose of combined therapy. Patients may have experienced ≥1 event per adverse event, but it was only counted once at the worst Common Terminology Criteria for Adverse Events grade.

**Table 3. Anti-tumor activity in evaluable HER2 positive patients stratified according to prior anti-HER2 or PD-1/PD-L1 regimens in the phase Ib and II studies**

|  | GC/GEJ (N = 27) | CRC (N = 22) | Non-GC/CRC (N = 12) | Total (N = 61) |
| --- | --- | --- | --- | --- |
| **Prior anti-HER2 regimens** | 15 | 9 | 3 | 27 |
| ORR [n, (%)] | 8 (53.3) | 4 (44.4) | 2 (66.7) | 14 (51.9) |
| DCR [n, (%)] | 14 (93.3) | 9 (100) | 2 (66.7) | 25 (92.6) |
| DoR, months (95% CI) | 6.6 (3.0, NE) | 6.3 (4.2, NE) | 4.1 (4.1, NE) | 4.5 (4.1, 10.8) |
| **Prior PD-1/PD-L1 blockades** | 9 | 1 | 5 | 15 |
| ORR [n, (%)] | 5 (55.6) | 0 | 4 (80.0) | 9 (60.0) |
| DCR [n, (%)] | 8 (88.9) | 1 (100) | 4 (80.0) | 13 (86.7) |
| DoR, months (95% CI) | NE (4.0, NE) | - | 4.8 (2.9, NE) | 6.6 (2.9, NE) |
| **Both prior anti-HER2 and PD-1/PD-L1 regimens** | 6 | 0 | 0 | 6 |
| ORR [n, (%)] | 4 (66.7) | 0 | 0 | 4 (66.7) |
| DCR [n, (%)] | 6 (100) | 0 | 0 | 6 (100) |
| DoR, months (95% CI) | NE (4.0, NE) | - | - | NE (4.0, NE) |
|  | | | | |

**Table 4. Anti-tumor activity of all evaluable patients in the phase 1b study**

|  | GC | | | |  | CRC | Other |
| --- | --- | --- | --- | --- | --- | --- | --- |
|  | 1^st^ line | 2^nd^ line | ≥3^rd^ line | Total |  | ≥2^nd^ line | |
|  | N=7(%) | N=13(%) | N=12(%) | N=32(%) |  | N=9(%) | N=2(%) |
| **Best overall response** |  |  |  |  |  |  |  |
| CR | 0 | 0 | 0 | 0 |  | 0 | 0 |
| PR | 6 (85.7) | 5 (38.5) | 2 (16.7) | 13 (40.6) |  | 2 (22.2) | 2 (100.0) |
| SD | 0 | 6 (46.2) | 4 (33.3) | 10 (31.3) |  | 7 (77.8) | 0 |
| PD | 1 (14.3) | 2 (15.4) | 6 (50.0) | 9 (28.1) |  | 0 | 0 |
| ORR (95%CI) % | 85.7 (42.1,99.6) | 38.5 (13.9, 68.2) | 16.7 (2.1, 48.4) | 40.6 (23.7, 59.4) |  | 22.2 (2.8, 60.0) | 100 (15.8, 100.0) |
| DCR (95%CI) % | 85.7 (42.1,99.6) | 84.6 (54.6, 98.1) | 50.0 (21.1, 78.9) | 71.9 (53.3,86.3) |  | 100(66.4,100.0) | 100 (15.8, 100.0) |
| **HER2 status**  Positive | 7 (100.0) | 11 (84.6) | 8 (66.7). | 26 (81.3) |  | 7 (77.8) | 2 (100.0) |
| ORR (95%CI) % | 85.7 (42.1,99.6) | 45.5 (16.8, 76.6) | 25.0 (3.2, 65.1) | 50.0 (29.9, 70.1) |  | 28.6 (3.7, 71.0) | 100 (15.8, 100) |
| DCR (95%CI) % | 85.7 (42.1,99.6) | 90.9 (58.7, 99.8) | 62.5 (24.5, 91.5) | 80.8 (60.7,93.5) |  | 100 (59.0, 100) | 100 (15.8, 100) |
| Low-expression | 0 | 2 (15.4) | 4 (33.3) | 6 (18.8) |  | 0 | 0 |
| ORR (95%CI) % | 0 | 0 | 0 | 0 |  | 0 | 0 |
| DCR (95%CI) % | 0 | 50 (1.3, 98.7) | 25.0 (0.6, 80.6) | 33.3 (4.3,77.7) |  | 0 | 0 |
| Mutation | 0 | 0 | 0 | 0 |  | 2 (22.2) | 0 |
| ORR (95%CI) % | 0 | 0 | 0 | 0 |  | 0 | 0 |
| DCR (95%CI) % | 0 | 0 | 0 | 0 |  | 100 (15.8,100) | 0 |
| **PD-L1 status**  CPS ≥1 |  |  |  |  |  |  |  |
|  | 4 (57.1) | 3 (23.1) | 5 (41.7) | 12 (37.5) |  | 0 | 2 (100) |
| ORR (95%CI) % | 100 (39.8,100) | 33.3 (3.2, 65.1) | 20.0 (0.5, 71.6) | 50.0 (21.1,78.9) |  | 0 | 100 (15.8, 100) |
| DCR (95%CI) % | 100 (39.8,100) | 66.7 (9.4, 99.2) | 60.0 (14.7, 94.7) | 75.0 (42.8,94.5) |  | 0 | 100 (15.8, 100) |
| CPS < 1 | 2 (28.6) | 8 (61.5) | 4 (33.3) | 14 (43.8) |  | 6 (66.7) | 0 |
| ORR (95%CI) % | 50 (1.3,98.7) | 37.5 (8.5, 75.5) | 0 | 28.6 (8.4,58.1) |  | 16.7 (0.4,64.1) | 0 |
| DCR (95%CI) % | 50 (1.3,98.7) | 87.5 (47.4, 99.7) | 25.0 (0.6, 80.6) | 64.3 (35.1,87.2) |  | 100 (54.1,100) | 0 |

Note: All patients who achieved PR were HER2-positive.

**Table 5. The anti-tumor activity of HER2 positive GC patients stratified by PD-L1 status in the phase Ib and II studies**

|  | **GC** | | |
| --- | --- | --- | --- |
|  | **1^st^ line treatment**  **(n=38)** | **≥2^nd^ line treatment**  **(n=27)** | **Total**  **(n=65)** |
| **PD-L1 status** |  |  |  |
| **CPS ≥1 (n)** | 15 | 8 | 23 |
| ORR | 13 (86.7) | 3 (37.5) | 16 (69.6) |
| DCR | 15 (100) | 6 (75.0) | 21 (91.3) |
| Median DoR (95%CI) | 13.4 (3.3, NE) | NE (NE, NE) | 13.4 (3.3, NE) |
| Median PFS (95%CI) | 10.9 (3.7, 16.5) | NE (1.7, NE) | 10.9 (4.2, NE) |
| Median OS (95%CI) | NE (8.5, NE) | NE (NE, NE) | NE (16.5, NE) |
| **CPS < 1 (n)** | 4 | 9 | 13 |
| ORR | 3 (75.0) | 4 (44.4) | 7 (53.8) |
| DCR | 3 (75.0) | 8 (88.9) | 11 (84.6) |
| Median DoR (95%CI) | NE (3.1, NE) | 7.5 (4.0, NE) | 10.8 (3.1, NE) |
| Median PFS (95%CI) | NE (0.9, NE) | 4.8 (1.8, 12.6) | 5.3 (1.8, 12.9) |
| Median OS (95%CI) | NE (4.4, NE) | 12.9 (5.9, 17.9) | 17.6 (6.2, 20.1) |

**Table 6. Any grade immune-related adverse events (irAEs) occurring in ≥2% of patients and all grade ≥ 3 irAEs in the phase Ib and II studies**

|  | Phase Ib | | | Phase II | | | Total | |
| --- | --- | --- | --- | --- | --- | --- | --- | --- |
|  | N = 47 (%) | | N = 66 (%) | | | | N=113 (%) | |
|  | Grade ≥ 3 | All Grades | | | Grade ≥ 3 | All Grades | Grade ≥ 3 | All Grades |
| All | 3 (6.4) | 31 (66.0) | | | 11 (37.9) | 43 (97.0) | 14 (12.4) | 74 (65.5) |
| Diarrhea | 0 | 11 (23.4) | | | 1 (1.5) | 7 (10.6) | 1 (0.9) | 18 (15.9) |
| Hypothyroidism | 0 | 6 (12.8) | | | 0 | 11 (16.7) | 0 | 17 (15.0) |
| Rash | 0 | 7 (14.9) | | | 0 | 10 (15.2) | 0 | 17 (15.0) |
| hyperthyroidism | 0 | 3 (6.4) | | | 0 | 5 (7.6) | 0 | 8 (7.1) |
| Elevated TSH | 0 | 3 (6.4) | | | 0 | 3 (4.5) | 0 | 6 (5.3) |
| Fatigue | 0 | 1 (2.1) | | | 0 | 4 (6.1) | 0 | 5 (4.4) |
| Pruritus | 1 (2.1) | 1 (2.1) | | | 1 (1.5) | 4 (6.1) | 2 (1.8) | 5 (4.4) |
| Elevated AST | 0 | 0 | | | 2 (3.0) | 5 (7.6) | 2 (1.8) | 5 (4.4) |
| Interstitial lung disease | 0 | 3 (6.4) | | | 1 (1.5) | 2 (3.0) | 1 (0.9) | 5 (4.4) |
| Thyroid disorder | 0 | 4 | | | 0 | 0 | 0 | 4 (6.1) |
| Decreased TT3 | 0 | 0 | | | 0 | 4 (6.1) | 0 | 4 (6.1) |
| Elevated ALT | 0 | 0 | | | 1 (1.5) | 3 (4.5) | 1 (0.9) | 3 (2.7) |
| Myocarditis | 0 | 0 | | | 0 | 3 (4.5) | 0 | 3 (2.7) |
| Decreased fT4 | 0 | 0 | | | 0 | 3 (4.5) | 0 | 3 (2.7) |
| Elevated CK | 0 | 1 (2.1) | | | 0 | 2 (3.0) | 0 | 2 (1.8) |
| Hypopituitarism | 0 | 0 | | | 2 (3.0) | 2 (3.0) | 2 (1.8) | 2 (1.8) |
| Hyponatremia | 0 | 0 | | | 1 (1.5) | 2 (3.0) | 1 (0.9) | 2 (1.8) |
| Elevated cTnI |  |  | | | 1 (1.5) | 1 (1.5) | 1 (0.9) | 2 (1.8) |
| Immune related enteritis | 0 | 0 | | | 1 (1.5) | 1 (1.5) | 1 (0.9) | 1 (0.9) |
| Immune related gastritis | 0 | 0 | | | 1 (1.5) | 1 (1.5) | 1 (0.9) | 1 (0.9) |
| Encephalitis | 1 (2.1) | 1 (2.1) | | | 0 | 0 | 1 (0.9) | 1 (0.9) |
| Hydronephrosis | 0 | 0 | | | 1 (1.5) | 1 (1.5) | 1 (0.9) | 1 (0.9) |
| Immune related hepatitis | 0 | 0 | | | 1 (1.5) | 1 (1.5) | 1 (0.9) | 1 (0.9) |
| Immune related endocrine abnormalities | 1 (2.1) | 1 (2.1) | | | 0 | 0 | 1 (0.9) | 1 (0.9) |
|  |  |  | | |  |  |  |  |
