# Supplementary material for: Efficacy and safety of KN026, a bispecific anti-HER2 antibody, in combination with KN046, an anti-CTLA4/PD-L1 antibody, in patients with advanced HER2-positive nonbreast cancer: a combined analysis of a phase Ib and a phase II study
Source: Signal Transduct Target Ther. 2025 Mar 19;10:104. doi: 10.1038/s41392-025-02195-x (PMC11923254; doi:10.1038/s41392-025-02195-x)
Supplement: Supplementary file 2 — The protocol of the Phase Ib study [file 41392_2025_2195_MOESM2_ESM.pdf]

CLINICAL STUDY PROTOCOL

KN026 / KN046

A Phase Ib Clinical Study to Evaluate the Efficacy, Safety, and Tolerability of KN026 in Combination with KN046 in Patients with HER2-positive Solid Tumor

Protocol No.: KN046-IST-02

Version No.: 2.0 (Final)

Version date: March 23, 2020

Number of pages: 118

DECLARATIONS

Alphamab Copyright Reserved. Confidentiality. The contents of this document may not be disclosed, published or communicated without the authorization of Alphamab.

Signature Page

We have read this clinical trial protocol (protocol No.: KN046-IST-02, version No.: 2.0, version date: March 23, 2020). I agree to perform relevant responsibilities in accordance with Chinese law, the Declaration of Helsinki, CFDA GCP and this protocol. This study will not be conducted unless approved by the Ethics Committee.

During the conduct of the study, I will strictly adhere to this protocol. In case any amendments to the protocol are required, the sponsor will be notified and approval will be obtained from the sponsor as well as from the Ethics Committee (or filing with the committee) before the amendments are implemented, unless immediate measures must be taken to protect the safety, rights and interests of subjects.

I will keep this protocol and related content confidential.

Jiangsu Alphamab Biopharmaceuticals Co., Ltd.

Medical director (printed): Xu Junfang

(Signature): Signature Date: (MM/DD/YYYY)

Signature Page

We have read this clinical trial protocol (protocol No.: KN046-IST-02, version No.: 2.0, version date: March 23, 2020). I agree to perform relevant responsibilities in accordance with Chinese law, the Declaration of Helsinki, CFDA GCP and this protocol. This study will not be conducted unless approved by the Ethics Committee.

During the conduct of the study, I will strictly adhere to this protocol. In case any amendments to the protocol are required, the sponsor will be notified and approval will be obtained from the sponsor as well as from the Ethics Committee (or filing with the committee) before the amendments are implemented, unless immediate measures must be taken to protect the safety, rights and interests of subjects.

I will keep this protocol and related content confidential.

Sponsor: Beijing Cancer Hospital

Principal investigator (printed): Shen Lin

(Signature): Signature Date: (MM/DD/YYYY)

Protocol Synopsis

**Investigational products: KN026 (Recombinant Humanized Anti-HER2 Bispecific Antibody); KN046 (Recombinant Humanized PD-L1/CTLA4 Bispecific Single Domain Fc Fusion Protein Antibody)**<br><br>**Protocol No.: KN046-IST-02**
**Study Title: A Phase Ib Clinical Study to Evaluate the Efficacy, Safety, and Tolerability of KN026 in Combination with KN046 in Patients with HER2-positive Solid Tumor**
**Investigator/site: 5-10 clinical study sites in China**
**Phase: Ib**
**Study objectives:**<br><br>**Primary objectives:**<br>• **Dose escalation period: To evaluate the maximum tolerated dose (MTD) and/or recommended phase II dose (RP2D) of KN026 in combination with KN046;**<br>• **Dose expansion period: To evaluate the anti-tumor activity of KN026 in combination with KN046 in patients with HER2-positive solid tumor.**<br><br>**Secondary objectives:**<br>• To evaluate the safety and tolerability of KN026 in combination with KN046;<br>• To evaluate the effect of HER2 amplification on the efficacy of KN026 in combination with KN046;<br>• To evaluate the effect of HER2 expression levels (IHC1+ vs. IHC2+ vs. IHC3+) on the efficacy of KN026 in combination with KN046;<br>• To evaluate the effect of KN026 and KN046 drug exposure levels on anti-tumor activity;<br>• To evaluate immunogenicity of KN026;<br>• To evaluate immunogenicity of KN046.<br><br>**Exploratory objectives:**<br>• To evaluate the effect of PD-L1 expression, TMB, GEP, and TIL on the efficacy of KN026 in combination with KN046.
**Study design:**<br><br>This study is an open-label, Phase Ib, multi-center clinical study to evaluate the efficacy and safety of KN026 in combination with KN046 in subjects with HER2-positive or HER2-expressing solid tumor.<br><br>The study is divided into 2 phases: a dose escalation phase and a dose expansion phase. The prespecified KN026 dose groups in the dose escalation period are 20 mg/kg Q2W group and 30 mg/kg Q2W group; the prespecified KN046 dose group is 3 mg/kg Q2W group (Figure 1). The adaptive dose finding method "3+3" is used in the dose escalation phase. "3+3" design allows dose increase or decrease based on a calculated decision table during subject enrollment so that MTD can be determined. After completion of the dose escalation phase, the dose limiting toxicity is determined by isotonic regression of "3+3" model. The dose closest to the target DLT is the MTD. 3 subjects will be enrolled in the first dose group (KN026 20 mg/kg Q2W + KN046 3 mg/kg Q2W), if no DLT was observed, the dosage was increased. Also, if a DLT was observed in the first three patients at a given dose level, 3 additional eligible patients were enrolled (no additional patients were required if a DLT was observed in one of six patients), if no DLT was observed, the dosage was increased. Dose escalation continued until two out of six patients in each dose cohort experienced a DLT. And an SMC will be convened after DLT observation. In addition, the first dose group may be expanded to a maximum of 12 subjects. If the second dose group meets the criterion of "escalation to higher dose", the subjects will enter the third dose group (KN026 30 mg/kg Q3W, loading dose + KN046 5 mg/kg Q3W on C1D1 and D8). If the third dose group meets the criterion of "maintain the original dose", subjects may be expanded up to 12 and the DLT observation for the entire dose escalation cohort may be

ended. The recommended Phase II dose (RP2D) will be decided by the SMC after the end of dose escalation and the subjects will enter the dose expansion phase. The SMC will decide the starting dose of KN026 and KN046 in the dose escalation phase, add the intermediate dose group, or add other cohorts at a dose level based on data including safety and pharmacokinetics from previous dose groups, and/or data from other studies of KN026 and KN046.

Subjects with HER2-positive or HER2-expressing solid tumor will be enrolled in the dose escalation period. HER2-positive is defined as follows:

- HER2 IHC3+ (ASCO/CAP 2018; Wolff et al, 2018); **or**
- HER2 gene amplification (ISH+: HER2/CEP17 ratio > 2 or HER2 gene copy number > 6; or NGS: HER2 gene copy number > 6); **or**
- HER2 gene mutation (NGS method);
    - HER2 Exon 20 insertion;
    - HER2 deletion near amino acids 755-759;
    - G309A, G309E, S310F, D769H, D769Y, V777L, P780-Y781insGSP, V842I, R896C；
    - Non-synonymous activating mutations (or insertions and deletions) found in 2 or more samples reported in the COSMIC database

HER2 expression is defined as IHC 1+ or IHC 2+ and ISH-.

In Part 1 of the dose expansion phase, patients with HER2-positive gastric/gastroesophageal junction cancer (GC/GEJ) who have not received systemic therapy (N: about 20-25) will be enrolled. Subjects who have received prior HER2-targeted therapy such as trastuzumab in the dose escalation phase or expansion phase should provide tumor tissue samples collected after failure of HER2-targeted therapy for determination of HER2-positive status.

An SMC meeting will be held after the end of Part 1 of the dose expansion phase to decide whether to conduct Part 2 of the dose expansion phase and further collect efficacy data from patients with HER2-positive or HER2-expressing solid tumor. The definition of HER2-positive in Part 2 of the extension phase will be adjusted (if applicable) based on the results of Part 1.

All subjects will be required to submit slides for central laboratory review of HER2 status at screening. The testing flow of the central laboratory is shown in the following table:

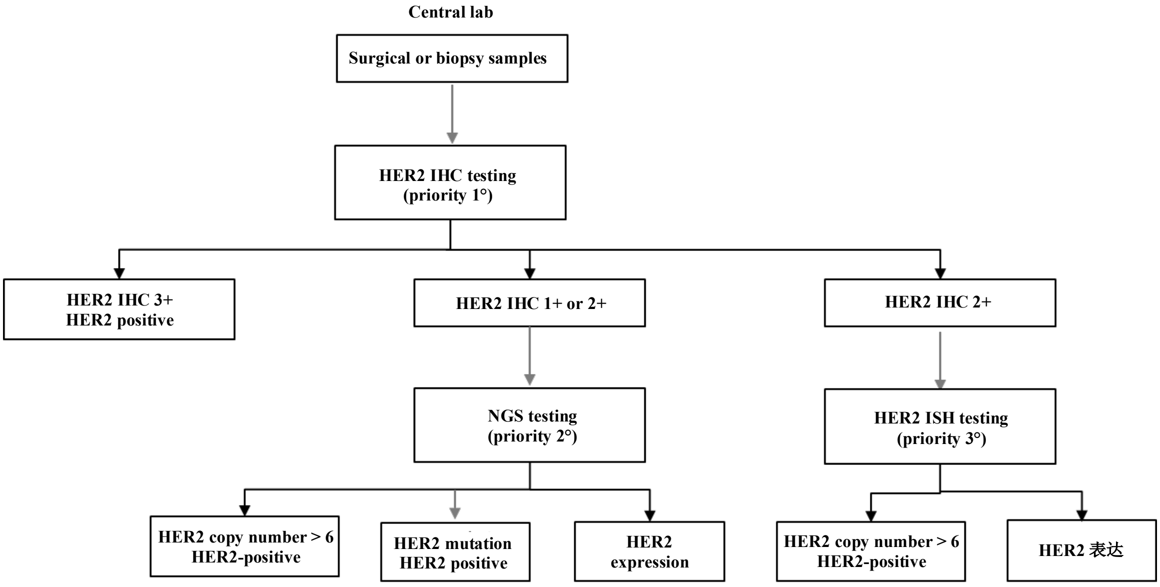

Each subject's study period includes a screening period (-28 to 0 day), a treatment period (up to 2 years of treatment; if the investigator judges that the subject is still benefiting after 2 years, continuation of treatment is allowed with the consent of the sponsor and Alphamab), end of treatment follow-up (within 7 days after the decision to discontinue treatment), 30-day safety follow-up, 90-day safety follow-up, and survival follow-up.

Each subject will receive KN026 in combination with KN046 per protocol until radiographic progression, intolerable toxicity, or withdrawal of consent per RECIST 1.1, whichever occurs first. After the investigator first judges the subject condition as disease progression per RECIST 1.1 criteria, if the subject is clinically stable and did not experience intolerable toxicity during the treatment, the subject is allowed to continue treatment with KN046 until disease progression is confirmed. Clinical stability is defined as: Stable ECOG score, no unacceptable toxicity associated with KN046 treatment, no rapid disease progression requiring other salvage therapy, and no emergencies requiring urgent medical intervention due to disease progression (e.g. central nervous system metastases, dyspnea due to tumor compression of the airway, spinal cord compression, etc.). The clinical, oncology, and laboratory evaluation flow of each study phase is detailed in Table 1 and 2 (Evaluation Flow Chart) as well as Section 7.

Study population, inclusion criteria, exclusion criteria:

Inclusion criteria:

I01. Subjects are able to understand the Informed Consent Form (ICF), voluntarily participate in the study and sign the ICF;

I02. Subjects ≥ 18 and ≤ 75 years of age, male or female, on the day of signing the informed consent form;

I03. Histologically or cytologically confirmed as patients with metastatic or locally advanced unresectable HER2-positive or HER2-expressing solid tumor. HER2-positive is defined as follows:

- HER2 IHC 3+; **or** HER2 gene amplification: In situ molecular hybridization ISH method (fluorescence in-situ hybridization, FISH; dual-color silver-enhanced in situ hybridization, DSISH) is used to confirm HER2 gene amplification (HER2/CEP17 ratio > 2.0), or NGS HER2 copy number > 6; **or** NGS HER2 gene mutation;

  Note: HER2 gene mutations include: HER2 Exon 20 insertion; HER2 deletion near amino acid 755-759; G309A, G309E, S310F, D769H, D769Y, V777L, P780-Y781insGSP, V842I, R896C; non-synonymous activating mutations (or insertions and deletions) found in 2 or more samples reported in the COSMIC database

  Note: HER2 status at enrollment may be confirmed by a local laboratory or a central laboratory and used for eligibility evaluation, and if HER2 status is confirmed by a local laboratory, pathological slides must be provided to the sponsor-designated central laboratory for review; local and central laboratories should not only report HER2 gene amplification, but also indicate HER2/CEP17 values. If the patient has received HER2-targeted therapy in the front line and disease has progressed, a post-progression tumor tissue sample is required to confirm HER2 status;

  Note: For colorectal cancer, **wild-type RAS** is required;

- HER2 expression is defined as IHC 1+; **or** IHC 2+ and no amplification of HER2 gene is confirmed by ISH method of in situ molecular hybridization (fluorescence in situ hybridization, FISH; dual-color silver-enhanced in situ hybridization, DSISH);

I04. Prior anti-tumor therapy requires the following:

- HER2-positive GC/GEJ: Metastatic or locally advanced unresectable, no prior systemic therapy has been received, and relapse and metastasis have existed more than 12 months from the end of neoadjuvant/adjuvant chemotherapy;
- HER2-positive or HER-expressing GC/GEJ: **Having received** ≥ 1 prior systemic therapy for metastatic or locally advanced unresectable GC/GEJ and the disease has progressed, and front-line systemic therapy includes at least platinum- or fluorouracil-based chemotherapy with or without trastuzumab; subjects who have relapsed within 12 months after the end of neoadjuvant/adjuvant chemotherapy are considered as failing first-line therapy;
- HER2-positive or HER-expressing non-GC/GEJ GI: **Having received** ≥ 1 prior systemic therapy for metastatic or locally advanced unresectable tumor and the disease has progressed; front-line systemic therapy for ESCC and mCRC includes at least platinum in combination with fluorouracil or taxane-based chemotherapy; mCRC requires ≥ 2 lines of systemic therapy for metastatic or locally advanced unresectable tumor with disease progression; subjects who have

relapsed within 12 months after the end of neoadjuvant/adjuvant chemotherapy or radical chemoradiotherapy are considered as failing the first-line treatment;

I05. At least 1 measurable lesion at baseline per RECIST 1.1 criteria. If a subject has only 1 measurable lesion at baseline, the lesion area must have not received prior radiotherapy, or there is evidence of significant progression of the lesion after the end of radiotherapy;

I06. ECOG score 0 or 1 (Appendix 5);

I07. LVEF ≥ 50%, determined by echocardiography (ECHO); multiple uptake gated acquisition (MUGA) scan will be used only in the absence of ECHO, and baseline and subsequent follow-up methods will be the same;

I08. NYHA functional class 0-1;

I09. Liver function meets the following criteria within 7 days prior to the first dose:

- Total bilirubin ≤ 1.0 × ULN (Gilbert's syndrome or total bilirubin of subjects with liver metastasis ≤ 1.5 × ULN);
- Transaminase (ALT/AST) ≤ 1.5 × ULN (≤ 3 × ULN for subjects with liver metastases);

I10. Renal function within 7 days prior to the first dose: Serum creatinine ≤ 1.5 × ULN and serum creatinine clearance ≥ 60 mL/min (calculated according to the Cockcroft-Gault formula);

I11. Bone marrow function meets the following criteria within 7 days prior to the first dose:

- Hemoglobin ≥ 9.0 g/dL;
- Absolute neutrophil count ≥ 1.5 × 10^9/L;
- Platelet count ≥ 100 x 10^9/L;
- INR or PT ≤ 1.5 × ULN and aPTT ≤ 1.5 × ULN;

I12. TSH is normal; if TSH is abnormal, total T3 or free T3, and free T4 should be within the normal range;

I13. Life expectancy ≥ 3 months;

I14. Female subjects of childbearing potential or male subjects with a partner of childbearing potential agree to use highly effective contraception (see Appendix 4) from 7 days before the first dose until 24 weeks postdose. Female subjects of childbearing potential must have a negative serum pregnancy test within 7 days before the first dose;

I15. Subjects are able and willing to comply with protocol-scheduled visits, treatment regimens, laboratory tests, and other study-related procedures.

Exclusion criteria:

E01. Subjects with untreated active brain metastases or with meningeal metastases; if the subject's brain metastases are treated and the metastasis condition is stable (brain imaging at least 4 weeks prior to the first dose shows stable disease and there are no new neurological symptoms, or neurological symptoms have returned to baseline), and there is no evidence of new or enlargement of the original brain metastases, enrollment is allowed;

E02. Decrease in LVEF to < 45% or absolute decrease in LVEF > 15% during prior HER2-targeted therapy;

E03. Prior cumulative doses of anthracyclines exceeding doxorubicin, liposomal doxorubicin or other anthracyclines by > 320 mg/m^2;

E04. Having participated in any other interventional study within 28 days before the first dose;

E05. Having received other anti-tumor therapy within 28 days prior to the first dose or within 5 half-lives of the previous anti-tumor drug, whichever is shorter;

E06. Having received major surgical treatment (such as major abdominal, transthoracic surgery; excluding diagnostic aspiration or peripheral vascular access replacement) within 28 days prior to the first dose;

E07. Radical radiotherapy within 3 months prior to the first dose; palliative radiation therapy within 2 weeks prior to the first dose is allowed, the radiation dose meets the diagnostic and treatment criteria for local palliative treatment, and the radiation coverage is less than 30% of the bone marrow area;

E08. Previous treatment with immune checkpoint blockers or T-cell costimulatory drugs, etc., including but not limited to immune checkpoint blockers such as PD-1, PD-L1, CTLA4, LAG3, therapeutic vaccines, etc. (Only for subjects included in DLT observation, subjects enrolled in the expansion phase are allowed to have received prior immune checkpoint blockers);

E09. Subjects who require systemic corticosteroids (≥ 10 mg/day prednisone or equivalent dose of other corticosteroids) or immunosuppressive therapy for 5 consecutive days within 14 days prior to the first dose in this study; except inhaled or topical corticosteroids, or physiologic replacement doses of corticosteroids for adrenal insufficiency; short-term (≤ 7 days) corticosteroids are allowed for prophylaxis (e.g., contrast media allergy) or for the treatment of non-autoimmune disorders (e.g., delayed-type hypersensitivity reactions due to contact allergens);

E10. Having received live vaccines (including live attenuated vaccines) within 28 days before the first dose;

E11. Previous or current interstitial pneumonia/lung disease;

E12. Having a history of or current autoimmune diseases, including, but not limited to, Crohn's disease, ulcerative colitis, systemic lupus erythematosus, sarcoidosis, Wegener's syndrome (granulomatosis with polyangiitis, Graves' disease, rheumatoid arthritis, hypophysitis, uveitis), autoimmune hepatitis, systemic sclerosis (scleroderma, etc.), Hashimoto's thyroiditis (refer to the following exceptions), autoimmune vasculitis, autoimmune neuropathy (Guillain-Barre syndrome), etc. With the following exceptions: Type I diabetes mellitus, hypothyroidism with stable hormone replacement therapy (including hypothyroidism caused by autoimmune thyroid disorder), psoriasis or vitiligo that does not require systemic treatment;

E13. Have other malignancies within 5 years before the first dose, except cured skin squamous cell carcinoma, basal cell carcinoma, non-muscle invasive bladder cancer, localized low-risk prostate cancer (defined as stage ≤ T2a, Gleason score ≤ 6, and PSA ≤ 10 ng/mL (as measured) at diagnosis of prostate cancer, the patients had received curative treatment and no prostate-specific antigen (PSA) biochemical recurrence), and in-situ cervical/breast cancer;

E14. History of uncontrolled complications including but not limited to:

- Active HBV or HCV infection;
- Known history of HIV infection or AIDS;
- Active tuberculosis;
- Active infection, or systemic use of anti-infective drugs within 28 days before the first dose of KN046;
- Uncontrolled hypertension (BP ≥ 150/95 mmHg at rest), symptomatic cardiac insufficiency (NYHA II-IV), unstable angina or myocardial infarction within 6 months, or risk of QTc prolongation or arrhythmia (baseline QTc > 470 msec <Fridericia correction>, refractory hypokalemia, long QT syndrome, atrial fibrillation with heart rate > 100 bpm at rest, or severe valvular disease, etc.);

E15. Toxicities with prior anti-tumor therapy did not recover to CTCAE ≤ Grade 1 (NCI-CTCAE v5.0) or baseline, with the exception of alopecia and skin pigmentation (any grade);

E16. Prior history of allogeneic bone marrow or organ transplantation;

E17. Prior history of allergic reaction, hypersensitivity reaction, and intolerance to antibody drugs; history of significant allergy to drugs (e.g., severe allergic reactions, immune-mediated hepatotoxicity, immune-mediated thrombocytopenia or anemia);

E18. Pregnant or breastfeeding women;

E19. Other conditions that, in the opinion of the investigator, would affect the safety or compliance with the study drug, including but not limited to moderate to large pleural/ascites/pericardial effusion, difficult-to-correct pleural/ascites/pericardial effusion, psychiatric disorders, etc.

Number of subjects:

Dose escalation period: up to approximately 30 persons;

Dose expansion period: 20-25 subjects.

Study treatment:

Investigational product: KN046

Dosage form and strength: Solution for injection, i.v., 40 mg/1.6 mL/vial, or 300 mg/12 mL/vial

Route of administration: Intravenous infusion over no less than 90 min (90-120 min)

Treatment: 3 mg/kg i.v. or higher

Treatment period:

Q2W: 28 days (4 weeks) or other dosing intervals

Investigational product: KN026

Dose and strength: Lyophilized powder, 50 mg/vial; or solution for injection, i.v., 325 mg/13 mL/vial

Route of administration: Intravenous infusion, the first infusion time is 90 min (± 15 min); if no infusion-related adverse event occurs during the first infusion, the subsequent infusion time can be adjusted to approximately 60 min (45-75 min).

Dose regimen: 20 mg/kg i.v.; or 30 mg/kg i.v.

Treatment period:

Q2W: 28 days (4 weeks) or other dosing intervals

Study endpoints:

Primary endpoints:

- **Dose escalation period:** Dose-limiting toxicity (DLT);
- **Dose-expansion phase:**
  - Objective response rate (ORR) as judged by the investigator per RECIST 1.1 criteria; and
  - Duration of response (DOR)

Secondary endpoints:

- Progression-free survival rate (PFSR) and clinical benefit rate at Month 6 and 12 judged by the investigator according to RECIST 1.1 criteria (clinical benefit rate (CBR; defined as CR, PR, or SD > 24 weeks);
- Overall survival rate (OS rate) at Month 6 and 12;
- Frequency and severity of adverse events (AEs) (NCI CTCAE 5.0); changes in vital signs, physical examinations, electrocardiograms, and safety laboratory measures;
- Correlation between biomarkers (HER2 IHC, HER2 amplification) and clinical efficacy parameters (ORR, CBR, PFSR, etc.);
- The correlation of KN026 and KN046 drug exposure (AUC_{ss}, $C_{trough,ss}$, $C_{avg,ss}$, etc.) with efficacy measures (ORR, DOR, CBR, etc.);
- Incidence and titer of anti-KN026 antibody (ADA); incidence of KN026 neutralizing anti-drug antibody (NADA) in subjects with high ADA titer;
- Incidence and titer of anti-KN046 antibody (ADA); incidence of KN046 neutralizing anti-drug antibody (ADA) in subjects with high ADA titer).

Exploratory endpoint:

- Correlation between biomarkers (PD-L1 expression, TMB, TIL) and clinical efficacy parameters (ORR, CBR, PFSR, etc.).

Evaluation procedure: Refer to Table 1 and Table 2

Statistical methods:

Sample size:

The sample size calculation for the dose escalation phase is not based on statistical assumptions, but the number of subjects in each dose group is specified according to the 3+3 method.

In Part 1 of the dose expansion phase, 20 to 25 subjects will be enrolled. The sample size calculation is based on the estimate of the 95% CI for ORR using the Clopper Pearson method:

**Sample size**	**ORR,%**	**ORR 95% CI**
25	40%	(21.1%, 61.3%)
	45%	(24.4%, 65.1%)
	50%	(31.3%, 72.2%)
	55%	(34.9%, 75.6%)
	60%	(38.7%, 78.9%)
	65%	(42.5%, 82.0%)
	70%	(50.6%, 87.9%)
	75%	(54.9%, 90.6%)
**Sample size**	**ORR,%**	**ORR 95% CI**
20	40%	(19.1%, 63.9%)
	45%	(23.1%, 68.5%)
	50%	(27.2%, 72.8%)
	55%	(31.5%, 76.9%)
	60%	(36.1%, 80.9%)

	65%	(40.8%, 84.6%)
	70%	(45.7%, 88.1%)
	75%	(50.9%, 91.3%)

Analysis population:

- The **safety dataset (SS)** includes all subjects who have received at least 1 full or partial dose of KN026 and/or KN046. Subjects will be categorized according to the treatment groups planned in the protocol. All raw data will be counted using SS, which will be the default analysis set used for all analyses unless otherwise noted;
- The **efficacy analysis set (EAS)** includes all subjects who have received at least 1 full or partial dose of KN026 and/or KN046 and have at least 1 postbaseline tumor imaging evaluation. Subjects will be categorized according to the treatment groups planned in the protocol. The ORR and DOR data are statistically analyzed using the EAS;
- The **DLT analysis set** includes all subjects in the dose escalation phase of the study who have received ≥ 80% of KN026 and ≥ 80% of KN046 scheduled dosing, or experienced a DLT during the DLT observation period;
- The **PK analysis set** is defined as subjects who have received at least 1 full or partial dose of KN026 and/or KN046 and have at least 1 post-dose PK sample for analysis;
- The **immunogenicity analysis set** is defined as subjects who have received at least 1 full dose or partial dose of KN026 and/or KN046 and have at least 1 post-dose immunogenicity sample for analysis.

Analysis of primary endpoints:

- **DLT**: The frequency of DLTs will be reported by dose level according to the DLT analysis set;
- **ORR and DOR**: The 95% confidence interval of the exact probability method of ORR calculated by Clopper Pearson will be reported by treatment group based on the EAS analysis set; for time-related events (DOR), parameters calculated by the Kaplan-Meier method will be reported by treatment group and HER2 status (HER2 positive vs. low HER2 expression).

Analysis of secondary endpoints:

- Safety analyses will be performed based on the SS. Descriptive statistical analyses of safety endpoints will be performed by treatment groups. Safety analyses will be based on the incidence of AEs, treatment-emergent AEs (TEAEs), immune-related AEs (irAEs), treatment-related AEs (TRAEs), and changes in vital signs, ECG, weights, ECOG score, and laboratory values (hematology and serum chemistry);
- Efficacy analyses will be based on the EAS. For individual proportions (ORR, CBR), the 95% confidence intervals calculated by Clopper Pearson will be reported by treatment group; for time-related events (DOR, PFS, OS), parameters calculated by the Kaplan-Meier method (including median and 95% confidence interval) will be reported by treatment group and HER2 status (HER2 positive vs. low HER2 expression);
- The correlation between HER2 amplification and clinical efficacy variables will be based on the ROC curve analysis of HER2/CEP17 ratio versus clinical efficacy variables (ORR, CBR, PFS rate, OS rate), and the comparison of HER2 IHC (IHC1+ vs. 2+ vs. 3+) will be based on analysis of variance;
- The correlation of KN026 and KN046 drug exposure (AUC_{ss}, $C_{trough,ss}$, $C_{avg,ss}$, etc.) with clinical response variables (ORR, DOR, CBR, etc.) and the influence of the interaction (additive, synergistic, etc.) between KN026 and KN046 on clinical response variables will be analyzed through Logistic regression model;
- For immunogenicity, the frequency and titer of anti-KN026 and KN046 antibodies will be listed by treatment group and immunogenicity analysis set, and the descriptive statistical analysis will be performed; for subjects with high titers, neutralizing antibody data will also be assessed and presented.

Analysis of exploratory endpoints:

- The PD-L1 expression level and the correlation between TMB and clinical efficacy variables will be analyzed based on the ROC curve of PD-L1 expression level and TMB value against clinical efficacy variables (ORR, CBR, PFS rate, OS rate), and the comparison of TIL (low vs. medium vs. high) will be based on analysis of variance.

STUDY PROCEDURES

1.1 OVERALL STUDY DESIGN

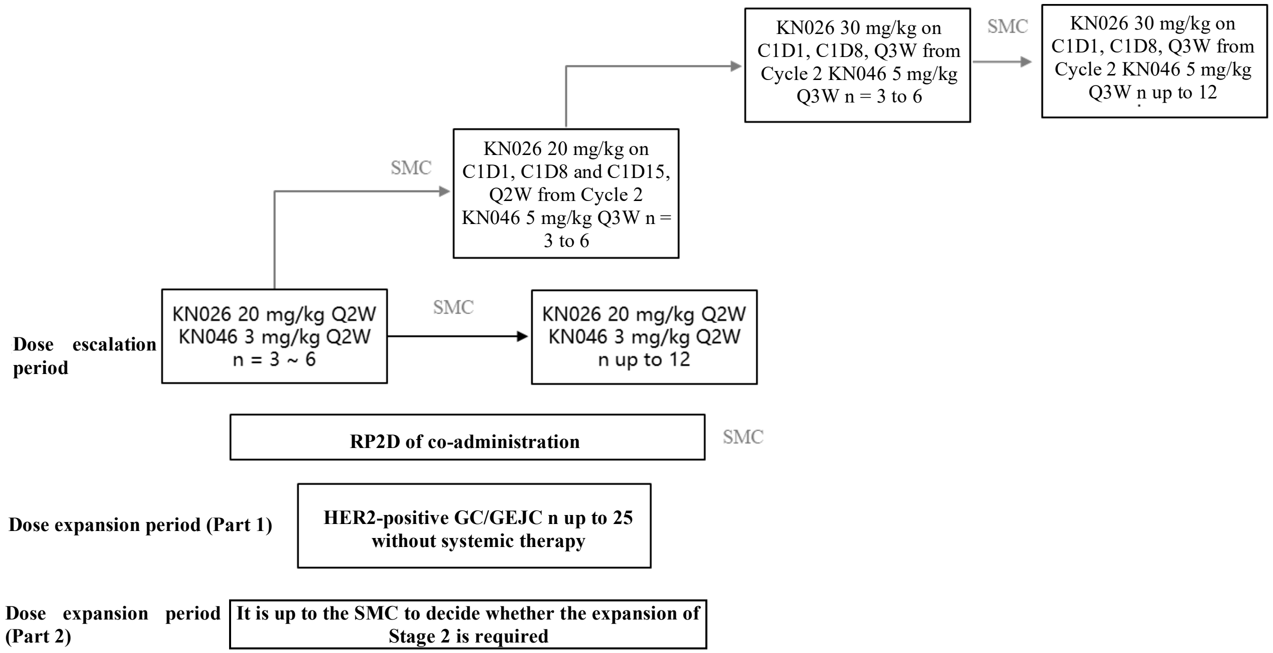

Note: The SMC will decide the starting dose of KN026 and KN046 in the dose escalation period, add the intermediate dose group, or add other cohorts at a dose level based on data including safety and pharmacokinetics from previous dose groups, and/or data from other studies of KN026 and KN046

1.2 STUDY FLOW CHART

Table 1 Evaluation flow chart

Evaluation	Screening	Treatment period							End of treatment	Safety follow-up	Long-term safety follow-up	Survival follow-up
**Visit**	**Screening**	**Week 1**	**Week 2**	**Week 3**	**Week 4**	**Week 5**	**Week 6**	**Repeat Week 1 to 6 every 6 weeks from Week 7 onward**	**EOT**	**30-day follow-up**	**90-day follow-up**	**Every 12 weeks**
**Day**	**(Day -28 to Day 0)**	**1**	**1 (± 3 days)**	**1 (± 3 days)**	**1 (± 3 days)**	**1 (± 3 days)**	**1 (± 3 days)**	**1 (± 3 days)**	**Within 7 days after the decision to discontinue treatment[1]**	**Within 30 days (± 3 days) after last dose**	**Within 90 days (± 7 days) after last dose**	**± 14 days**
**General procedures**												
Informed consent	X											
Inclusion/exclusion criteria	X											
Demographic data	X											
Medical history/comorbidities	X											
Tumor history	X											
Prior medication history/concomitant medications/concomitant procedures[2]	X	X							X	X	（X）	（X）
Subsequent anti-tumor therapy									X	X	X	X
Survival status												X
**Clinical examination/evaluation**												
**AE[3]**	X	X							X	X	X	X
**General physical examination**	X								X			
**Symptom-directed physical examination**		Prior to each dose of KN026 and/or KN046								X	X	
**Height**	X											
**Weight**	X	Prior to each dose of KN026 and/or KN046							X	X	X	

Vital signs	X	Prior to each dose of KN026 and/or KN046							X	X	X	
12-lead ECG	X	Every 6 weeks							X			
ECOG score	X	Prior to each dose of KN026 and/or KN046							X	X	X	
**Local laboratory tests/evaluations**[4]												
Blood count and differential[5]	X[8]	Prior to each dose of KN026 and/or KN046							X	（X）[9]		
Coagulation	X[8]	If clinically indicated										
Serum chemistry[6]	X[8]	Prior to each dose of KN026 and/or KN046							X	（X）[9]		
Urinalysis[7]	X[8]	Every 12 weeks, or as clinically indicated							X	（X）[9]		
Thyroid function	X	Every 6 weeks, or as clinically indicated							X	（X）[9]		
LVEF	X	Every 6 weeks, or as clinically indicated							X		X	Every 3 months till 1 year
Troponin	X	If clinically indicated										
Serum pregnancy test (if applicable)	(X)[10]								(X)			
Urine pregnancy test (if applicable)		(Every 12 weeks)										
Follicle stimulating hormone (only if confirmation of menopausal status is required)	(X)											
HBV, HCV, HIV[10]	X											
CRP	X											
**Central laboratory tests/evaluations**												
HER2 status (HER2 IHC, HER2 amplification) (tumor tissue)[11]	X											
Biomarkers (tumor tissue) (non-mandatory)[11]	X											
Pharmacokinetics		See Table 2										
ADA		See Table 2										
**Tumor imaging evaluation**												

Tumor imaging (thoracic/abdominal/pelvic)[13,14]	X	Every 8 weeks; after 48 weeks: every 12 weeks							(X)	(X)	(X)	(X)
Tumor imaging (brain, if applicable)[13,14]	(X)	(if clinically indicated)										
Bone scan (if applicable)[15]	(X)	(if clinically indicated)										
**Study drug administration**												
KN046 administration (Q2W)[16]		X		X		X		Administration on d1, 15/28				
KN026 administration (Q2W)[16]		X	(X)	X		X		Administration on d1, 15/28				
KN046 administration (Q3W)[16]		X			X			Administration on Day 1/21				
KN026 administration (Q3W)[16]		X	(X)		X			Administration on Day 1/21				

1 The EOT visit may be performed on the day of the KN026 and KN046 discontinuation, in which case the same EOT tests may not be repeated if they are performed on the same day as the last pre-treatment evaluation; if the 30-day safety visit is on the same day as the EOT visit, the same tests may not be repeated;

2 All concomitant medications/procedures within 28 days prior to dosing should be recorded. Concomitant medications/procedures after 30-day safety follow-up period are also to be recorded as part of SAE recording, analysis, and reporting if they are performed for the purpose of treating SAEs related to KN046 and/or KN026;

3 After the subject signs the informed consent form, all adverse events need to be recorded until 30 days after the last dose of KN046 and/or KN026 or initiation of new anti-tumor therapy, whichever occurs first. SAEs and TRAEs will be collected up to 90 days after the last dose of KN046 or 30 days after the last dose of KN026, whichever occurs later. The investigator is required to monitor adverse events until stable or until the outcome is known, unless the subject is recorded as "lost to follow-up". In the event of treatment-related serious adverse events (SAEs) of KN046, they need to be recorded whenever they occur and regardless of the time from discontinuation of KN046, and reported to regulatory authorities in accordance with local regulations; the symptomatic left ventricular systolic dysfunction and asymptomatic left ventricular systolic dysfunction ($\geq$ 10% and < 50% decrease in LVEF from baseline) within 12 months after the last dose of KN026 should be recorded and reported as AEs, and those meeting the criteria for SAEs should be reported to regulatory authorities in accordance with local regulations;

4 Safety laboratory evaluations should be obtained prior to dosing; pre-dose safety laboratory evaluations on Cycle 1 Day 1 should be performed at screening. Safety laboratory evaluations should include: blood count and differential, coagulation tests (PT/INR/aPTT/TT), serum chemistry, urinalysis, thyroid function (TSH/T3/free T3/free T4), troponin, HBV/HCV/HIV, and serum/urine pregnancy test. The blood count and differential, coagulation tests (PT/INR/aPTT/TT), and serum chemistry tests should be obtained within 7 days prior to the first dose and used for evaluation of inclusion/exclusion criteria. Detailed description can be referred to Section 7.3.3;

5 Blood count and differential includes: red blood cell count (RBC), hematocrit, hemoglobin, mean hemoglobin content (MCH), mean corpuscular hemoglobin concentration (MCHC), mean corpuscular volume (MCV), platelet count, white blood cell count (WBC), neutrophil count, lymphocyte count, monocyte count, basophil count, eosinophil count;

6 Serum chemistry tests include: alkaline phosphatase, gamma-glutamyltransferase, alanine aminotransferase (ALT), aspartate aminotransferase (AST), albumin, total protein, total bilirubin, creatinine, serum urea nitrogen/total urea, fasting blood glucose, blood calcium, blood potassium, blood sodium, chloride, phosphate, lactate dehydrogenase (LDH);

7 Urinalysis includes: pH, specific gravity, glucose, protein, ketone, urine blood cells; if urine protein ≥ 2+ (dipstick), the 24-hour urine protein and urine protein/creatinine ratio should be collected;

8 Test results should be obtained within 7 days (including 7 days) before dosing;

9 The items with abnormal EOT examination need to be re-determined at 30-day safety follow-up;

10 The test includes HBV, HCV antibody and HIV antibody. If HBsAg is positive, HBV DNA should be added; if HCV antibody is positive, HCV RNA should be added;

11 Formalin-fixed archival specimen (biopsy or surgery) tissue (block or slide) containing tumor tissue obtained recently (tumor tissue biopsy from a non-irradiated area within 2 years) is required for subjects at screening for HER2 IHC, HER2 amplification, PD-L1 expression, TMB, GEP, and TIL analysis. Endoscopic biopsy, core needle biopsy, excisional biopsy, trephine biopsy, and surgical specimens are acceptable. Fine needle aspiration biopsy specimens are not acceptable. Samples may be provided as tumor-containing FFPE tissue blocks or slides of blocks, slides should be freshly cut (within 1 week), 4 to 6 µm thick, and mounted with SuperFrost Plus microscope slides, it is preferable to provide approximately 15 to 20 slides, if not possible, it is recommended to provide 10 to 15 slides, of which 5 slides are to be used by the central laboratory for HER2 status review. If the subject has received prior HER2-targeted therapy and has disease progression, the tumor tissue samples after failure of HER2-targeted therapy are required for confirmation of HER2 status (for HER2 IHC and HER2 expansion). If there are less than 10 slides, the investigator and the sponsor's medical monitor are required to decide whether to enroll the subject, and if slides are less than 5, the subject is not allowed to be enrolled (screening failure);

12 Subjects will have chest/abdomen/pelvis (specific tumor types need to include other specific areas) CT scan or MRI (chest CT is mandatory if MRI is used). If CT/MRI imaging is not sufficient to evaluate tumor burden, other established evaluation methods may be added. Contrast-enhanced CT scan is recommended, and contrast-enhanced MRI may be considered if the subject is allergic to contrast. The imaging method needs to be consistent with that used for lesion detection at baseline, and the same imaging device is preferred for subsequent tumor evaluation visits. Baseline tumor imaging assessments should be completed within 21 days prior to the first dose, including at least thoracic, abdominal, and pelvic cavities. Subjects who have not undergone imaging evaluation of brain tumors within 42 days prior to the first dose are required to undergo brain enhanced CT or MRI at screening. Tumor imaging follow-up will be performed every 8 weeks (± 7 days), every 12 weeks (± 7 days) after 48 weeks until disease progression (RECIST 1.1), initiation of new anti-tumor therapy, withdrawal of informed consent, loss to follow-up, death, or end of the study, whichever occurs first (Section 7.3.2.3). Brain imaging follow-up is required only if clinically indicated (e.g., new CNS symptoms or worsening of previous symptoms).

The first occurrence of disease response (CR, PR) as judged by RECIST 1.1 criteria requires reexamination of imaging no earlier than 4 weeks and no later than 8 weeks to confirm the disease response per RECIST 1.1 criteria, and the first occurrence of disease progression as judged by RECIST 1.1 criteria requires reexamination of imaging no earlier than 4 weeks and no later than 8 weeks to confirm the disease progression per RECIST 1.1 criteria (Section 7.3.2.3, 6.1.3);

14 Subjects who discontinue treatment not due to imaging tumor progression (e.g., unacceptable toxicity [Section 6.1.4], clinical deterioration) must continue to be followed for tumor imaging after discontinuation until tumor progression per RECIST 1.1 criteria, initiation of new anti-tumor therapy, withdrawal of informed consent, or loss to follow-up, whichever occurs first (Section 7.3.2.3); if a subject experiences disease progression as judged by RECIST 1.1 criteria and is confirmed, but meets the criteria for continued treatment after progression (Section 6.1.3), and continues to have clinical benefit from continued treatment with KN046 as judged by the investigator, continued follow-up imaging and oncology evaluation are allowed with reference to iRECIST criteria[19];

15 Subjects who did not have a bone scan within 90 days prior to the first dose are required to have a bone scan at screening. Subsequently, the bone scan should be performed only if clinically indicated. For new lesions suggested by the bone scan, CT/MRI examination is required to determine whether it is metastatic, and oncology evaluation will be performed according to RECIST 1.1 criteria;

16 The duration of each KN046 infusion should not be less than 90 minutes (90 to 120 minutes); after the end of each dose of KN046, the subject should be observed at the study site for at least 2 hours. The first infusion time of KN026 should be 90 minutes (± 15 minutes). If no infusion-related AEs occurred during the first infusion, the subsequent infusion time could be adjusted to 60 minutes (45 to 75 minutes); after the end of each dose of KN026, the subject should be observed at the study site for at least 2 hours. If KN046 and KN026 are administered on the same day, KN026 should be administered first and then KN046 after 2 hours of the end of infusion.

Table 2 Schedule of evaluation procedures: PK and immunogenicity collection form for KN046 (Q2W) and KN026 (Q2W)

Dose n	Time	KN046 PK samples	KN046 ADA samples	KN026 PK samples	KN026 ADA samples
1	0 h (pre-dose of KN046 or KN026) (-60 min)	3.5 mL	3.5 mL	3.5 mL	3.5 mL
1	End of KN046 or KN026 infusion (+ 30 min)	3.5 mL		3.5 mL	
(*)	0 h (pre-dose of KN026) (-60 min)			3.5 mL	3.5 mL
(*)	End of KN026 infusion (+ 30 min)			3.5 mL	
2	0 h (pre-dose of KN046 or KN026) (-60 min)	3.5 mL	3.5 mL	3.5 mL	3.5 mL
3	0 h (pre-dose of KN046 or KN026) (-60 min)	3.5 mL	3.5 mL	3.5 mL	3.5 mL
3	End of KN046 or KN026 infusion (+ 30 min)	3.5 mL		3.5 mL	
4	0 h (pre-dose of KN046 or KN026) (-60 min)	3.5 mL	3.5 mL	3.5 mL	3.5 mL
5	0 h (pre-dose of KN046 or KN026) (-60 min)	3.5 mL	3.5 mL	3.5 mL	3.5 mL
11	0 h (pre-dose of KN046 or KN026) (-60 min)	3.5 mL	3.5 mL	3.5 mL	3.5 mL
21	0 h (pre-dose of KN046 or KN026) (-60 min)	3.5 mL	3.5 mL	3.5 mL	3.5 mL
33	0 h (pre-dose of KN046 or KN026) (-60 min)	3.5 mL	3.5 mL	3.5 mL	3.5 mL
45	0 h (pre-dose of KN046 or KN026) (-60 min)	3.5 mL	3.5 mL	3.5 mL	3.5 mL
EOT		3.5 mL	3.5 mL	3.5 mL	3.5 mL
30-day safety follow-up		3.5 mL	3.5 mL	3.5 mL	3.5 mL
90-Day safety follow-up		3.5 mL	3.5 mL	3.5 mL	3.5 mL
Total		***49 mL***	***42 mL***	***49 mL***	***42 mL***

(*) Applicable to the dose that is increased in the KN026 loading dose regimen

PROTOCOL TABLE OF CONTENTS

Clinical Study Protocol **1**
Study Procedures **13**
1.1 Overall study design 13
1.2 Study flow chart 14
Protocol: Table of Contents **21**
Abbreviations **26**
1 Study Background **28**
1.1 EGFR/HER 28
1.2 HER2-positive tumors and immunotherapy 28
1.3 KN026 29
1.3.1 Preclinical pharmacokinetic study 30
1.3.2 Preclinical toxicology studies 30
1.3.3 Clinical studies 31
1.4 KN046 32
1.4.1 Preclinical pharmacokinetic study 32
1.4.2 Preclinical toxicology studies 32
1.4.3 Clinical studies 33
2 Study Rationale **36**
2.1 Justification for dose levels 36
2.1.1 Rationale for dose levels of KN026 36
2.1.2 Rationale for dose level of KN046 38
2.2 Rationale for primary study endpoints 38
2.3 Rationale for indication 38
2.4 Benefit - Risk Assessment 39
3 Study Objectives and Endpoints **40**
3.1 Study objectives 40
3.1.1 Primary objective 40
3.1.2 Secondary objectives 40
3.1.3 Exploratory objectives 40
3.2 Study endpoints 40
3.2.1 Primary endpoints 40

3.2.2 Secondary endpoints 40
3.2.3 Exploratory endpoints 41
4 Study Design 42
4.1 Overall study design 42
4.1.1 Definition of end of study 46
4.1.2 Premature discontinuation of study 46
4.2 Interim analysis 46
4.3 Scientific Monitoring Committee (SMC) 46
5 Study Population 48
5.1 Inclusion criteria 48
5.2 Exclusion criteria 49
5.3 Subject withdrawal criteria 51
5.3.1 Discontinuation of study drugs 51
5.3.2 Withdrawal from study 52
6 Study Drugs and Subject Treatment 53
6.1 Investigational product 53
6.1.1 Drug dosage form and strength 53
6.1.2 Drug preparation 53
6.1.2.1 Preparation of KN046 53
6.1.2.2 Preparation of KN026 54
6.1.3 Dose and method of administration of study drugs 54
6.1.4 Dose modifications for study drugs 55
6.1.4.1 Dose modification of KN046 55
6.1.4.2 Dose modification of KN026 57
6.1.5 Treatment assignment 60
6.1.6 Drug packaging and labeling 60
6.1.7 Drug storage, transportation and shelf life 60
6.1.8 Drug quantity management 60
6.1.9 Assessment of compliance 61
6.1.10 Occupational safety 61
6.2 Concomitant medications and non-drug therapies 61
6.2.1 Permitted medications 61
6.2.2 Prohibited medications and procedures 61
6.2.3 Special precautions 62
6.2.3.1 Infusion-related and hypersensitivity reactions 62

6.2.3.2 Tumor lysis syndrome 64
7 Study Procedures and Evaluations 65
7.1 Study visit plan 65
7.1.1 Screening 65
7.1.2 Treatment period 66
7.1.3 End of treatment (EOT) visit 66
7.1.4 30-day and 90-day safety visits 67
7.1.5 Long-term follow-up 67
7.2 Study evaluation 68
7.2.1 Demographic and other baseline characteristics 68
7.2.1.1 Demographic data 68
7.2.1.2 Tumor diagnosis 68
7.2.1.3 Medical history 68
7.2.1.4 Other baseline evaluations 69
7.2.2 Efficacy evaluation 69
7.2.2.1 Computed tomography (CT), MRI, FDG PET/CT, and other evaluations 69
7.2.2.2 Baseline oncology evaluation 70
7.2.2.3 Subsequent oncology evaluations 70
7.2.3 Safety evaluation 71
7.2.3.1 Physical examination 71
7.2.3.2 Vital signs 72
7.2.3.3 Body height and weight 72
7.2.3.4 ECOG score 72
7.2.3.5 Laboratory tests 72
7.2.4 Pharmacokinetic evaluation 74
7.2.5 Immunogenicity evaluation 74
7.2.6 Biomarker evaluation 75
7.2.6.1 Evaluation of tumor tissue biomarkers 75
8 Evaluation and Recording of Adverse Events 76
8.1 Definition of adverse events 76
8.1.1 Adverse event 76
8.1.2 Serious adverse event 76
8.2 Recording and reporting of adverse events 77
8.2.1 Recording and reporting of serious adverse events 78
8.2.1.1 Follow-up of serious adverse events 79

8.2.2 Adverse reactions of special interest 79
8.2.3 Recording and reporting of laboratory test abnormalities and other abnormalities . 80
8.2.3.1 Hepatic function abnormal 80
8.2.4 Pregnancy and intrauterine exposure 81
8.2.5 Overdose, medication error, and drug abuse 81
9 Data Analysis and Statistics 82
9.1 Statistical methods 82
9.2 Sample size calculation 82
9.3 Analysis of data sets 82
9.3.1 Safety set 83
9.3.2 Efficacy analysis set 83
9.3.3 DLT analysis set 83
9.3.4 PK analysis set 83
9.3.5 Immunogenicity analysis set 83
9.4 Demographic and other baseline characteristics 83
9.5 Primary endpoint analysis 83
9.6 Analysis of secondary endpoints 84
9.6.1 Safety endpoints 84
9.6.1.1 Adverse event 84
9.6.1.2 Tolerability 85
9.6.1.3 Laboratory abnormalities 85
9.6.1.4 Other safety data (physical examination, ECG, vital signs) 85
9.6.2 Efficacy endpoints 85
9.6.3 Pharmacokinetic endpoints 86
9.6.4 Correlation between pharmacokinetic and clinical efficacy parameters 86
9.6.5 Immunogenicity endpoints 86
9.7 Exploratory endpoint analysis 86
10 Ethics and Regulatory 87
10.1 Regulations and ethics 87
10.2 Responsibilities of investigators and IEC/IRC 87
10.3 Subject information and consent 87
10.4 Subject privacy 88
10.5 Clinical study insurance and subject compensation 88
10.6 Regulatory authority monitoring, quality assurance and inspection 89
11 Study Management 90

11.1 Closure of study and study center 90
11.2 Storage of study data 90
11.3 Provision of study results and information to investigators 91
11.4 Information disclosure and patents 91
11.5 Publication of study results 92
12 References 93
13 Appendices 95
Appendix 1 Response Evaluation Criteria in Solid Tumors (RECIST 1.1) 96
Appendix 2 Immune Response Evaluation Criteria in Solid Tumors (iRECIST) 104
Appendix 3 Management Principles of Immune-related Adverse Events 106
Management of immune-related pneumonitis 107
Management of immune-related colitis 109
Management of immune-related endocrine adverse events 111
Management of immune-related hepatitis 112
Management of immune-related rash 113
Management of immune nephritis 114
Management of immune-related myocarditis 115
Appendix 4 Contraception Guidance 116
Appendix 5 Eastern Cooperative Oncology Group Performance Status 117
Appendix 6 IHC Interpretation of HER2 Detection in Gastric Cancer 118

ABBREVIATIONS

ADA:	Anti-drug antibody
AE:	Adverse event
ALT:	Alanine transaminase
AIDS:	Acquired immunodeficiency syndrome
aPTT:	Partial thromboplastin time
AST:	Aspartate aminotransferase
CBR:	Clinical benefit rate
CR:	Complete response
CT:	Computerized tomography
DLT:	Dose limiting toxicities
DDR:	DNA damage response
DOR:	Duration of response
EAS:	Efficacy analysis set
ECG:	Electrocardiogram
ECOG:	Eastern cooperative oncology group performance status
HBV:	Hepatitis B virus
HCV:	Hepatitis C virus
HIV:	Human immunodeficiency virus
INR:	International normalized ratio
MRI:	Magnetic resonance imaging
NSCLC:	Non-small cell lung cancer
NCI-CTCAE:	National Cancer Institute-Common Terminology Criteria for Adverse Events
NYHA:	New York Heart Association
ORR:	Objective response rate
OS:	Overall survival
PD-L1:	Programmed death receptor ligand-1
PD:	Pharmacodynamics
PET:	Positron emission tomography
PFS:	Progression-free survival
PK:	Pharmacokinetics
PT:	Prothrombin time
PR:	Partial response
RECIST:	Response Evaluation Criteria in Solid Tumors
SAE:	Serious adverse event
SS:	Safety analysis set
SD:	Stable disease
SMC:	Scientific review committee
TEAE:	Treatment-emergent adverse events
TESADR:	Treatment-emergent serious adverse drug reactions
TIL:	Tumor-infiltrating lymphocyte
TMB:	Tumor mutation burden
TRAE:	Treatment-related adverse events

TRSADR：	Treatment-related serious adverse drug reactions
WOCBP:	Women of childbearing potential

1 STUDY BACKGROUND

1.1 EGFR/HER

The human epidermal growth factor receptor (EGFR or HER) family consists of four distinct types: HER1 (EGFR or c-erbB-1), HER2 (neu, p185 or cerbB-2), HER3 (c-erbB-3), and HER4(c-erbB-4). These four molecules compile a family of 20 receptor tyrosine kinases (RTKs) of type I[1] [2] [3]. This family of RTKs plays an important role in cell proliferation, differentiation, adhesion, survival, and migration. The 20 RTK families share some structural relationships, including extracellular and intracellular segments linked by transmembrane segments, showing high functional and structural identity. In the HER family, similarity of these molecules ranges from 53% (EGFR and HER3) to 64% (EGFR and HER2). The most conserved sequence is located on the tyrosine kinase domain with 59 to 81% identity, and the C-terminal sequence shows a poor similarity between 12 to 30%[4].

HER proteins consist of highly conserved extracellular domains (ECDs) or extracellular binding regions in which ligand fragments interact and result in molecular structural changes that promote receptor dimerization and enhance the kinase activity. The ECD includes domains I, II, III, and IV. Domains I and III interact with corresponding ligands. II and IV are domains common to the HER group and consist of two highly reactive cysteine-rich domains that interact with each other and with the cell membrane. HER3 does not have intramembrane kinase activity whereas HER2 lacks ligand binding sites. However, these receptors can cooperate with each other to form active heterodimers and activate downstream pathways, leading to cell proliferation [5] [6] [7].

HER2 is a transmembrane 185 kDa protein, the gene of which is located on chromosome 17q2145. HER2 protein in mammary epithelial cells is between 20,000 and 50,000 per cell, but cancer cells have more than 2,000,000 receptors per cell [8] [9]. Overexpression of HER2 is also associated with poor prognosis in tumors such as ovarian cancer, gastric cancer, and uterine papillary serous carcinoma [10] [11] [12]. Mutations at codon 655 of the HER2 gene can alter the structure of the receptor transmembrane domain, resulting in an active form that promotes the formation of HER2 homodimers. The mutations of HER2 gene or overexpression of HER2 protein can promote the formation of HER2 homodimers, resulting in continuous uncontrolled growth and division, and apoptosis decrease. Amplification of HER2 results in overexpression of protein, formation of ligand-independent dimers, or formation of dimers with any other receptor in the same family. HER2 has no extracellular domain to which ligands can be ligated, but has high affinity for receptors in the same family, thereby forming heterodimers [1] [13]. Among type I RTKs, HER2 interacts most favorably with other molecules, prolonging ligand/heterodimer ligation, and prolonging activation of the mitogen-activated protein kinase (MAPK) pathway. In addition, HER2 does not interact with any ligand and its conformational arm is readily present in a competent form and linked to any other monomer of the same family [1] [14] [15].

1.2 HER2-positive tumors and immunotherapy

Synergy of HER2-targeted agents with immunotherapy was observed in both HER2-positive breast and gastric cancers. Trastuzumab can promote immunogenic cell death, leading to uptake of breast carcinoma-associated antigens (BCAAs) by dendritic cells (DCs), presenting and eliciting a $CD8^+$-specific immune response against breast cancer (BC) cells bearing BCAAs. In the NeoPHOEBE trial, neoadjuvant trastuzumab was combined with the pan-PI3K inhibitor Buparlisib or placebo for 6 weeks followed by the addition of paclitaxel. TIL infiltration was higher in tumor samples after 15 days of treatment compared with TIL score in baseline pretreatment biopsy, which was highly related to pCR [16]. Lapatinib blocks HER2 signaling by stabilizing HER2 protein expression on BC cell membranes, increasing the HER2 protein of FcR on mononuclear immune cells. When HER2 is blocked by lapatinib, the potential effect of IgG1 Fc on ADCC may explain the anti-tumor activity superimposed by trastuzumab. Trastuzumab in combination with lapatinib was superior to lapatinib in PFS (HR: 0.74; 95% CI: 0.58-0.94, p = 0.011) and OS (HR: 0.74; 95% CI: 0.57-0.97, p = 0.026) in the EGF104900 clinical trial [17]. These preclinical and clinical studies have suggested the potential of anti-HER2 therapy in combination with immunotherapy.

PD1 can strongly induce lymphocytes around BC cells. The combination of T-DM1 with checkpoint inhibitors (anti-PD-1 and anti-CTLA-4) has produced a curative effect in animal models, and combinations of PD-1 and CTLA4 inhibitors with anti-$HER2^+$ therapies are currently being developed. In the PANACEA Phase Ib/II study, pembrolizumab in combination with trastuzumab in patients with HER2 + BC had an objective response rate (ORR) of 15.2% in PD-L1-positive patients, 0% in PD-L1-negative patients, 39% in patients with more than 5% TIL in tumor samples, and 5% in patients with low TIL (< 5%) [18]. Margetuximab is an Fc-optimized mAb that targets HER2, which can enhance Fc ADCC function. The ORR of margetuximab in combination with pembrolizumab was 35.7% in patients with HER2 IHC3+ GC/GEJ and 63.6% in patients with PD-L1+/HER2 IHC3+ GC/GEJ, indicating synergistic efficacy of anti-HER2 therapy with immune checkpoint blockers [19].

Therefore, patients with HER2-positive tumors will be enrolled in this study to explore the efficacy of KN026 combined with KN046.

1.3 KN026

KN026 is a novel recombinant humanized bispecific antibody that binds two distinct extracellular domains II (pertuzumab recognition site) and IV (trastuzumab recognition site) of HER2 protein simultaneously. The intact KN026 antibody protein is a heterodimeric antibody molecule consisting of two different IgG1 isoform heavy chains (KN026 heavy chain I, KN026 heavy chain II) and two kappa isoform light chains with identical sequence (common light chain). Both ligand-dependent and ligand-independent signaling of HER2 can be blocked, and thus a better therapeutic effect is expected. The preclinical pharmacodynamic results showed that the anti-tumor effect of KN026 in multiple tumor models at the same dose is not only significantly superior to that of trastuzumab or pertuzumab alone, but also significantly superior to that of trastuzumab combined with pertuzumab in vivo for some tumor types. Preclinical studies have shown that KN026 can be enriched at high concentrations on the surface of tumor cells, and can well target trastuzumab-resistant HER2-overexpressing tumors, as well as tumors with low expression of HER2. ZW25 is a drug with the same mechanism of action as KN026 under investigation abroad, and has achieved some efficacy in solid tumors with low expression of HER2. Therefore, the preliminary efficacy of KN026 in combination with KN046 in HER2-expressing solid tumors will be explored in this study.

1.3.1 Preclinical pharmacokinetic study

After a single intravenous injection of KN026 to cynomolgus monkeys in the range of 1-25 mg/kg, half-life ($T_{1/2}$), mean residence time (MRT) and volume of distribution (Vz) tended to be prolonged, and clearance (CL) tended to be decreased with the increase of dose. Single doses of KN026 at 1, 5, and 25 mg/kg resulted in half-lives of 23.4, 49.6, and 91.4 h, respectively. There were no significant differences in the main pharmacokinetic parameters between male and female animals after dosing in each dose group. Maximum concentration (C_{max}) and serum exposure ($AUC_{(0-t)}$) increased with the increase of dose in cynomolgus monkeys.

After four consecutive subcutaneous injections of KN026 at 15, 40, and 120 mg/kg, the mean C_{max} and $AUC_{(0-t)}$ increased with the increase of dose in cynomolgus monkeys. There was no significant accumulation at the low, medium and high doses.

1.3.2 Preclinical toxicology studies

Single-dose and multiple-dose toxicity studies of KN026 were conducted in cynomolgus monkeys.

The maximum tolerated single dose was greater than 200 mg/kg in cynomolgus monkeys.

When KN026 was administered at 15, 40, 120 mg/kg once weekly for 5 consecutive doses, no effects were observed on the cardiovascular system, respiratory system and central nervous system of cynomolgus monkeys.

The results of a 6-week study with a 4-week recovery period in cynomolgus monkeys showed that no KN026-related changes were observed in food consumption, local irritation reaction at the administration site, ophthalmological examination, blood pressure, respiratory rate, body temperature, ECG, neurobehavioral examination, clinical pathology parameters, organ weights, gross anatomy and relevant pathology. The no observed adverse effect level (NOAEL) was 120 mg/kg/dose. At this dose, mean C_{max} and $AUC_{(0-t)}$ values of KN026 on Day 22 of the dosing period were 3641.06 μg/mL and 268432.64 hr*μg/mL in males and 3939.65 μg/mL and 325175.98 hr*μg/mL in females, respectively.

Immunogenicity: In the single intravenous dose PK study in cynomolgus monkeys, all 3 dose groups (1 mg/kg, 5 mg/kg, or 25 mg/kg) of animals were positive for anti-drug antibodies (ADAs) 42 days postdose, with the number of positive animals of 5/6, 3/6, and 4/6. In the multiple intravenous dose study in cynomolgus monkeys (15, 40, and 120 mg/kg weekly), the number of ADA-positive animals was 3/10, 4/10, and 3/10 on Day 29 postdose, and was 3/4, 3/4, and 2/4 on Day 57 postdose, respectively.

Hemolysis assay showed that KN026 (25 mg/mL) do not cause hemolytic reactions in red blood cells of rabbits.

In conclusion, the available preclinical toxicology studies support Phase I clinical studies in humans. The results of toxicology study are detailed in the IB.

1.3.3 Clinical studies

The first-in-human study of KN026 (KN026-CHN-I-001) was conducted in Fudan University Shanghai Cancer Center and enrolled subjects with HER2-positive advanced breast and gastric cancers who had failed prior standard of care using a traditional "3+3" dose escalation design. As of January 22, 2020, 63 patients with HER2-positive advanced breast cancer have been treated with KN026, including 5 mg/kg QW (n = 3), 10 mg/kg QW (n = 3), 20 mg/kg Q2W (n = 28), and 30 mg/kg Q3W (n = 29). All of these subjects had experienced prior second and later lines of anti-HER2 therapy (e.g., trastuzumab, lapatinib, T-DM1), and had completed DLT evaluation in all four dose groups, which showed the subjects are well tolerated and no DLT events occurred. Subjects received KN026 for a median exposure time of 12 weeks (range: 4 to 60 weeks). Of those, 33 (52.4%) subjects had an exposure time of more than 12 weeks, 8 (12.7%) subjects had more than 24 weeks, 2 (3.2%) subjects had more than 36 weeks, and 1 (1.6%) subject had more than 60 weeks. All 51 subjects had ≥ 1 postbaseline tumor assessment: for the 20 mg/kg Q2W group, 1 subject had complete disappearance of target lesions at two postbaseline evaluations, and the overall response was assessed as confirmed partial response (PR), 5 subjects were assessed as confirmed PRs, and 3 subjects were assessed as unconfirmed PRs; for the 30 mg/kg Q3W group, 2 subjects were assessed as confirmed PRs and 3 subjects were assessed as unconfirmed PRs. A total of 23 subjects had stable disease (SD), most of whom had varying degrees of shrinkage of target lesion. The objective response rate was 27.5%, and the disease control rate was 72.5%. At recommended Phase II doses (20 mg/kg Q2W and 30 mg/kg Q3W), the objective response rate was 31.1%, and the disease control rate was 75.6%. KN026 Treatment-related AEs were reported in 48 (76.2%) subjects, in which AEs with an incidence of ≥5% were: fever (n = 15, 23.8%), diarrhea (n = 12, 19.0%), aspartate aminotransferase increased (n = 9, 14.3%), hypokalemia (n = 6, 9.5%), infusion-related reaction (n = 6, 9.5%), ECG T wave abnormal (n = 6, 9.5%), white blood cell count decreased (n = 5, 7.9%), alanine aminotransferase increased (n = 5, 7.9%), blood creatinine increased (n = 5, 7.9%), proteinuria (n = 4, 6.3%), asthenia (n = 4, 6.3%), hypertriglyceridemia (n = 4, 6.3%), hyperglycemia (n = 4, 6.3%), rash (n = 4, 6.3%), anemia (n = 4, 6.3%), first degree atrioventricular block (n = 4, 6.3%), and neutrophil count decreased (n = 4, 6.3%). There were 4 ≥ Grade 3 treatment-related adverse events: in the 20 mg/kg Q2W group, 1 subject had a Grade 3 transaminase increased without bilirubin increased (leading to hospitalization; recovered after symptomatic treatment) and 1 subject had a Grade 3 ventricular arrhythmia (leading to hospitalization; discontinuation of KN026; recovered at a 30-day safety follow-up after symptomatic treatment); in the 30 mg/kg Q3W group, 1 subject had a Grade 3 infusion-related reaction (fever and chills on the first dose; recovered after treatment, but was hospitalized the next day due to fever) and 1 subject had a Grade 3 hypertension (the patient had a history of hypertension; the blood pressure transiently increased to Grade 3 2 hours after the first dose, and resolved on the same day). No KN026 treatment-related AEs led to death. No decrease in LVEF of ≥ 15% or below the lower limit of normal was observed. There were 5 KN026 treatment-related SAEs, including 1 Grade 3 transaminase increased, 1 Grade 3 ventricular arrhythmia, 1 Grade 2 interstitial pneumonia, 1 grade 3 cardiac myxoma and 1 infusion-related reaction. The occurrence of safety events was consistent with that reported with other anti-HER2 agents, and no new safety signals were noted. As of September 2019, pharmacokinetic data after the first dose have been available for 12 patients in Phase I KN026 study in China. KN026 showed a linear pharmacokinetic profile with the increase of dose for C_{max} and $AUC_{0-\infty}$ from 5 to 30 mg/kg. Mean predose KN026 plasma concentrations in the 10 mg/kg QW group had reached steady-state trough levels (79 μg/mL) at the approved dose of trastuzumab.

1.4 KN046

KN046 is a novel recombinant humanized bispecific antibody that binds both PD-L1 and CTLA-4, thereby blocking the binding of PD-L1 to PD1 and CTLA-4 to CD80/CD86. KN046 is expressed and produced by CHO cells and the wild-type IgG1 Fc in the molecule maintains ADCC and CDC functions. CTLA-4 inhibitors act on naive T cells in secondary lymphoid organs and also mediate the depletion of regulatory T cells (Tregs), resulting in anti-tumor effects. PD-1/PD-L1 inhibitors can relieve inhibitory conduction pathways in the tumor microenvironment, thereby activating tumor-infiltrating CTLs. The binding of KN046 molecule to PD-L1 is stronger than that of CTLA-4, and has a stronger inhibitory effect on tumors with high expression of PD-L1, so KN046 can strongly activate the immune system in the tumor microenvironment. In clinical toxicology studies in cynomolgus monkeys, KN046 also showed good tolerability.

1.4.1 Preclinical pharmacokinetic study

A preclinical PK study of KN046 was conducted in cynomolgus monkeys. After intravenous administration of 1-100 mg/kg KN046 in cynomolgus monkeys, C_{max} and AUC increased proportionally with the dose, with $T_{1/2}$ of 51-88.3 h and CL of 0.701-0.747 mL/hr/kg; the mean accumulation rate after multiple doses was less than 2.0 (0.72-1.38).

PK information for KN046 is detailed in the IB.

1.4.2 Preclinical toxicology studies

Single-dose and multiple-dose toxicity studies of KN046 were conducted in non-human primates.

Single-dose toxicity: Single-dose toxicity was also observed in a 4-week multiple-dose toxicity study in cynomolgus monkeys. Its results showed the MTD of a single dose of KN046 is approximately greater than 100 mg/kg.

Multiple-dose toxicity: In the 4-week multiple-dose toxicology study in cynomolgus monkeys, study drugs were administered intravenously at 0 mg/kg (control group), 10 mg/kg, 30 mg/kg, and 100 mg/kg weekly for a total of 5 doses, with a 6-week recovery period. In the 30 mg/kg group, 1 male died during the 5th dosing period. Pathological observations were performed on Day 18, 20, and/or 29, presumably due to immunogenic challenge with KN046, which had the highest ADA titer on Day 29, rather than a direct effect of KN046. KN046-related major changes were noted in the ≥ 30 mg/kg group, including enlarged groin nodes and decreased body weight and food consumption in 1 animal (at 100 mg/kg); pathological changes were mainly indicative of inflammatory responses, including slightly increased large unstained cell counts, mild to moderate increases in fibrinogen concentrations, and slight to mild increases in globulin concentrations; histopathologically noted adverse reactions included multiorgan vascular inflammation, mesangioproliferative glomerulonephropathy and/or concomitant tubular degeneration/necrosis/regeneration, hypertrophy/hyperplasia of Kupffer cells, neutrophil/mononuclear cell infiltration in liver sinusoids and/or portal areas, and cardiomyocyte degeneration/necrosis (at ≥ 30 mg/kg). The NOAEL for KN046 was determined to be 10 mg/kg.

Immunogenicity: In a single intravenous dose PK study in cynomolgus monkeys, all 3 groups of animals (1.0 mg/kg, 3.0 mg/kg, or 10.0 mg/kg) were positive for anti-drug antibodies (ADA) 42 days postdose, with the number of positive aninals of 17/18. Only 1 animal in the 3.0 mg/kg group was ADA-negative. In multiple intravenous dose study in cynomolgus monkeys (10, 30, and 100 mg/kg weekly), 4/10 animals were ADA-positive on Day 29, and 4/10, 0/10, and 1/10 animals were ADA-positive on Day 43 in three dose groups, respectively.

Hemolysis assay showed that KN046 (26.3 mg/mL) do not cause hemolytic reactions in red blood cells of rabbits.

In conclusion, the available preclinical toxicology studies support Phase I clinical studies in humans. Toxicology study results are detailed in the IB.

1.4.3 Clinical studies

There are two KN046 studies, including KN046-AUS-001 (first-in-human study of KN046) and KN046-CHN-001 (first-in-China study of KN046).

1) The first-in-human study of KN046 (KN046-AUS-001) was conducted at 3 study sites in Australia using a traditional "3 + 3" dose escalation design. As of January 20, 2020, a total of 54 subjects were enrolled in this study, including 0.3 mg/kg Q2W (n = 1), 1 mg/kg Q2W (n = 3), 3 mg/kg Q2W (n = 17), 5 mg/kg Q2W (n = 30), and 10 mg/kg Q2W (n = 3). Subjects received KN046 for a median exposure time of 11 weeks (range: 2 to 67 weeks). Of these, 45 (51.1%) subjects had an exposure time of more than 12 weeks, 20 (22.7%) subjects had more than 24 weeks, 12 (13.6%) subjects had more than 36 weeks, and 2 (2.3%) subjects had more than 48 weeks. KN046 was generally well tolerated by the subjects. Four DLT events were observed in 3 subjects, including 1 subject in the 5 mg/kg group who experienced Grade 3 treatment-related hepatic function abnormal without increased bilirubin, 1 subject in the 10 mg/kg group who experienced Grade 3 pruritic erythematous rash, and 1 subject in the 10 mg/kg group who experienced Grade 3 aspartate transferase increased and Grade 3 arthritis. These events were resolved within 3 weeks. Therefore, the maximum tolerated dose was considered to be 5 mg/kg Q2W.

   Treatment-related adverse events (TRAEs) of KN046 were reported in 41 (75.9%) subjects and KN046 TRAEs of Grade 3 or above were reported in 20 (37.0%) subjects. Treatment-related SAEs of KN046 were reported in 14 (25.9%) subjects and immune-related adverse events (irAEs) were reported in 26 (48.1%) subjects, of which 13 (24.1%) subjects experienced irAEs of Grade 3 or above. The most common TRAEs included arthralgia, fatigue, and infusion-related reactions. The most common irAEs included arthralgia (musculoskeletal and connective tissue disorders) and pruritus (skin and subcutaneous tissue disorders). No dose-related relationship was found for TRAEs and irAEs, and no increase in the number and severity of TRAEs or irAEs was found with escalation to the RP2D level as compared with the lower dose level.

   As of January 20, 2020, 35 subjects received at least one postbaseline response evaluation and were included in the efficacy analysis. The results showed 1 confirmed CR, 2 confirmed PR, 4 unconfirmed PR, 15 SD, and 20 PD. The objective response rate (CR + PR) was 16.7% and the disease control rate (CR + PR + SD) was 52.4%. As of the cut-off date, 8 evaluable subjects were still receiving KN046 treatment.

After administration of 3 mg/kg (n = 3), KN046 showed linear elimination in humans with half-life ranging from 4.4 to 7.3 days (mean 6.2 days) and trough concentrations ranging from 4.6 to 6.8 µg/L (mean 5.4 µg/L) on Day 15. After administration of 5 mg/kg (n = 6), KN046 showed linear elimination in humans with half-life ranging from 5.2 to 11.3 days (mean 8.0 days) and trough concentrations ranging from 5.9 to 16.2 µg/L (mean 12.8 µg/L) on Day 15

1) KN046-CHN-001 is a Phase 1a/1b study in Chinese subjects conducted at the Sun Yat-sen University Cancer Center. As of January 20, 2020, 88 subjects were enrolled in the study, including 1 mg/kg Q2W (n = 1), 3 mg/kg Q2W (n = 30), 5 mg/kg Q2W (n = 45), 5 mg/kg Q3W (n = 6), and fixed dose of 300 mg Q3W (n = 6). Subjects received KN046 for a median exposure time of 11 weeks (range: 2 to 54 weeks). Of these, 45 (51.1%) subjects had an exposure time of more than 12 weeks, 20 (22.7%) subjects had more than 24 weeks, 12 (13.6%) subjects had more than 36 weeks, and 2 (2.3%) subjects had more than 48 weeks. Among the 88 subjects, 33 subjects are still receiving study treatment, and 55 subjects have discontinued study treatment, including: 1) 36 subjects with disease progression; 2) 5 subjects with AEs; 3) 3 deaths; 4) 2 subjects lost to follow-up; and 5) 9 subjects with other reasons. The subjects enrolled in the Phase 1 study in China were mainly patients with advanced solid tumors. No dose-limiting toxicity (DLT) events were observed during the dose escalation phase, and the maximum tolerated dose was not reached at 5 mg/kg Q2W, therefore 5 mg/kg Q2W was considered as the RP2D. As of the cut-off date, 74 (84.1%) of 88 subjects experienced TRAEs, 9 (10.2%) experienced TRAEs of Grade 3 or above, and 4 (4.5%) experienced treatment-related SAEs. Forty (45.5%) subjects experienced irAEs, of which 4 (4.5%) were Grade 3. Similar to the results of the KN046-AUS-001 study, TRAEs and irAEs showed no dose dependence during the dose escalation phase. The most common TRAEs included rash (34.1%), pruritus (31.8%), alanine aminotransferase increased (19.3%), infusion-related reaction (17.0%), asthenia (17.0%), aspartate aminotransferase increased (13.6%), pyrexia (12.5%), and arthralgia (10.2%). The most common irAEs included skin and subcutaneous tissue disorders and general disorders and administration site conditions. As of January 20, 2020, 75 subjects were evaluable for response, including 8 confirmed PR and 27 SD. A total of 29 evaluable subjects are still receiving KN046. The objective response rate was 12.0% and the disease control rate was 48.0%. The PK was similar between the Chinese and Australian populations.

As of June 24, 2019, in vitro releasing data of IL-2 were obtained from 16 subjects, and a PK/PD model was constructed using R, with the model equation as:

$$E(C) = E0 - \frac{Imax \cdot (E0 - 1) \times C}{IC50 + C}$$

The model showed adequate goodness-of-fit plots. Due to the high residual standard error (RSE) of 115.6% for the model parameter IC_{50}, the range of 95%CI (180-457 ng/mL) of the interval estimates was too large for a precise estimation of the IC_{50}. Therefore, the corresponding Cmin targets were estimated from the point estimate of IC_{50} (138 ng/mL) and the upper limit of 95%CI (457 ng/mL), respectively. The corresponding Cmin targets were 2629 ng/mL and 8683 ng/mL, respectively. Based on the preliminary exposure-response relationship analysis of the efficacy data, 5 mg/kg Q2W was recommended for further exploration of KN046 monotherapy, and 5 mg/kg Q3W was recommended for further exploration of KN046 co-medication.

2 STUDY RATIONALE

2.1 JUSTIFICATION FOR DOSE LEVELS

2.1.1 Rationale for dose levels of KN026

The starting dose of KN026 in this study was 20 mg/kg Q2W, which was mainly selected based on safety and preliminary efficacy data from KN026-CHN-I-001 study (first-in-human study of KN026). The safety evaluation of KN026-CHN-I-001 at 20 mg/kg dose level has been completed. KN026 was generally well tolerated by the subjects without DLT events. There were 5 subjects with confirmed PR and 3 subjects with unconfirmed PR, and the subjects with SD had varying degrees of reductions in the target lesions. The dose selection also referred to the approved doses of the same class of drugs trastuzumab and pertuzumab, as well as the clinical study data and recommended Phase 2 dose of the investigational drug ZW25 with the same action mechanism.

The action mechanism of KN026 was similar to trastuzumab in combination with pertuzumab. As described in the package inserts of trastuzumab[10], trastuzumab is used for metastatic breast cancer with an initial loading dose of 4 mg/kg and a weekly maintenance dose of 2 mg/kg, resulting in a steady-state trough level of 79 μg/mL; trastuzumab is used for adjuvant treatment of breast cancer with an initial loading dose of 4 mg/kg and a maintenance dose of 6 mg/kg every 3 weeks, resulting in a steady-state trough level of 63 μg/mL. As described in the package inserts for pertuzumab[10], pertuzumab is used for adjuvant treatment of breast cancer with an initial loading dose of 840 mg and a maintenance dose of 420 mg every 3 weeks, resulting in a steady-state trough level of 60-70 μg/mL. Therefore, we expected that the mean trough concentration levels of the effective dose of KN026 would also be within this range. In the Phase 1 dose escalation study of KN026, the dose levels with trough concentrations within the range of 60-80 μg/mL will be selected as the dose in Phase 2 clinical study. At present, we have obtained PK data of KN0265 mg/kg and 10 mg/kg QW dose levels up to March 10, 2019, which showed that the pre-dose plasma concentration of KN026 in the 10 mg/kg QW dose group had reached the steady-state trough levels at the approved trastuzumab dose. Whereas the dose intensities of 20 mg/kg Q2W and 10 mg/kg QW were comparable.

ZW25 is a bispecific antibody against HER2 under investigation abroad, with the same target and action mechanism as KN026. The Phase 1 clinical trial of ZW25 was a dose escalation study conducted in solid tumors with HER2 overexpression and with HER2 moderate and low expression, with four dose groups of 5 mg/kg (QW), 10 mg/kg (QW), 15 mg/kg (QW), and 20 mg/kg (Q2W). All four dose levels showed good safety without DLTs, and most of the most common AEs were Grade 1-2, with no drug-related SAEs occurred. Cohort expansion of ZW25 was conducted at both 10 mg/kg QW and 20 mg/kg Q2W dose levels to further explore safety and efficacy, and subsequent studies were conducted with 20 mg/kg Q2W as the recommended Phase 2 dose. PK data from Phase 1 clinical study of ZW25 indicated the comparable dose intensities at 10 mg/kg QW and 20 mg/kg Q2W. Cross-comparison results showed a good coherence between the PK data of KN026 and ZW25 at 5 mg/kg QW dose level. Therefore, the dosing schedule of ZW25 in the expansion study can be used as one of the rationales for dose selection in this study.

Besides, the translational medicine researches of KN026 suggested that the effective concentration of KN026 was 20 µg/mL, and 60-80 µg/mL was the optimal tumor killing concentration range. A population PK model was constructed based on human PK data, and 1000 simulations of the target population were performed. The recommended Phase 2 dose was defined as that 90% or more of the simulated population reach the target concentration range of 60-80 µg/mL. In combination of the clinical safety data, 20 mg/kg Q2W or 30 mg/kg Q3W was used as the recommended Phase 2 dose of KN026, and steady state would be reached at Week 8 (Q2W) or Week 9 (Q3W) after multiple doses (Figure 1 and 2).

A human PK-tumor growth model was constructed based on human PK and efficacy data to analyze the correlation between PK and anti-tumor activity in humans. The analysis suggested that rapid maximal effective concentrations were associated with the optimal antitumor activity, so a loading dose was designed. The steady-state concentrations would be reached in the first cycle of administration after the loading dose in Weeks 1 and 8 at 20 mg/kg Q2W (Figure 3), which was expected to further improve the efficacy.

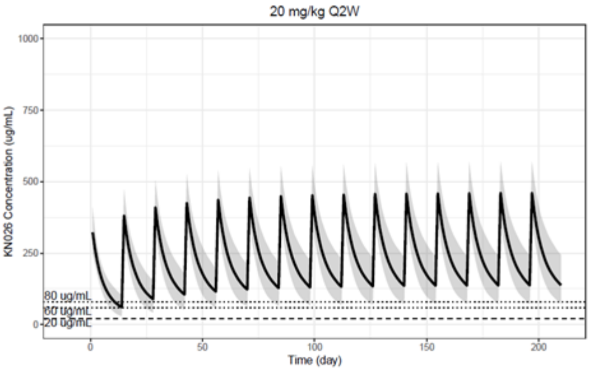

Figure 1

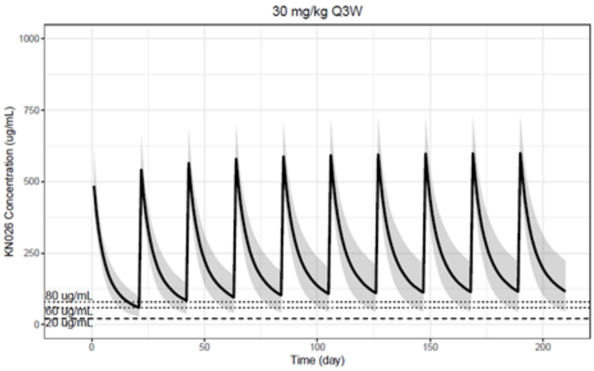

Figure 2

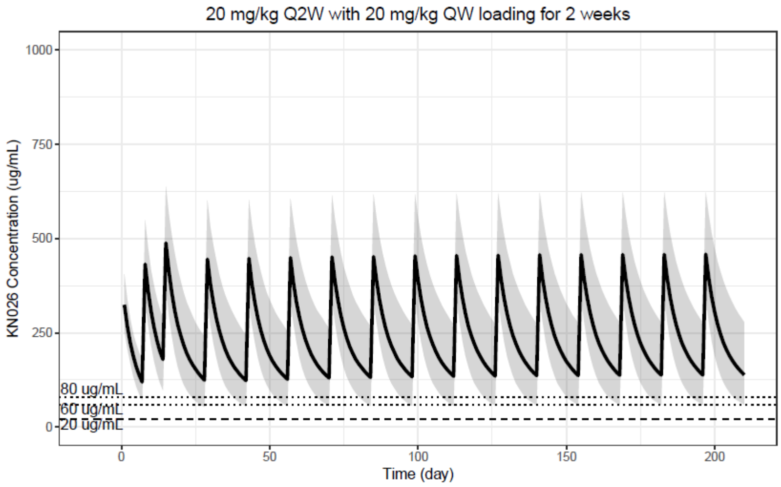

Figure 3

2.1.2 Rationale for dose level of KN046

The predetermined dose of KN046 for this study is 3 mg/kg Q2W. The rationale for dose selection was mainly based on the safety and preliminary efficacy data from the KN046-AUS-001 study (first-in-human study of KN046) and KN046-CHN-001 study (first-in-China study of KN046). KN046-AUS-001 has completed the safety evaluation at 5 mg/kg Q2W dose group and KN046-CHN-001 has completed the safety evaluation at 3 mg/kg dose group. KN046 was generally well tolerated by the subjects. One subject with NSCLC in the 3 mg/kg Q2W group was observed to be CR and 1 subject with ovarian cancer experienced 56% reduction in target lesions. At 3 mg/kg Q2W dosing, the steady-state trough concentration exceeded approximately 40 folds of the corresponding drug concentration for the maximum *in vitro* IL-2 releasing effect, which also suggested the 3 mg/kg dose as an effective pharmacodynamic dose.

2.2 RATIONALE FOR PRIMARY STUDY ENDPOINTS

The study will be conducted in 2 parts: dose escalation phase and dose expansion phase.

DLTs were regarded as the primary endpoint in the dose escalation phase. As a typical Phase 1 study, the occurrence of DLTs is an appropriate endpoint to evaluate safety and tolerability in the dose escalation phase.

The primary endpoints of the dose expansion phase were ORR and DOR as assessed by the IRC per RECIST 1.1 criteria. For early exploratory study of efficacy, objective response and response duration are acceptable surrogate endpoints for measuring clinical benefits and can reflect the antitumor activity of drugs, especially persistent antitumor response can be observed.

2.3 RATIONALE FOR INDICATION

The dose expansion phase will be conducted in HER2-positive gastric/gastroesophageal junction (GC/GEJ) tumors. The rationale for selection of HER2-positive tumors is provided in Section 1.2.

One female subject (HER2 IHC 3+) with moderately differentiated adenocarcinoma of GC previously treated with trastuzumab in combination with XELOX regimen was enrolled in the first dose group of this study (KN026 20 mg/kg Q2W + KN046 3 mg/kg Q2W), with a treatment assessment of SD at Week 8, whose target lesion reduction was approximately 25%. Besides, 1 male subject (HER2 IHC 3+) with moderately differentiated adenocarcinoma of GEJ who failed previous chemotherapy was enrolled, with a treatment assessment of PD at Week 8, whose target lesion reduction was approximately 45%. The main AEs of these two subjects were rash and diarrhea at Grade 1 or 2. Therefore, the subjects with HER2-positive GC/GEJ tumors who have not been systematically treated will be enrolled to explore the antitumor activity of the first-line therapy of KN026 in combination with KN046.

2.4 BENEFIT - RISK ASSESSMENT

The occurrence of AEs will be closely observed in the clinical trial, including vital signs, ECG, laboratory blood, and urine tests. Based on the preclinical studies of KN026 and clinical results of currently marketed anti-HER2 monoclonal antibody drugs with the same target, the most common adverse reactions included pyrexia, nausea, vomiting, infusion-related reactions, diarrhea, infection, cough aggravated, headache, asthenia, dyspnea, rash, neutropenia, anemia, and myalgia. Most were mild to moderate in severity. Adverse reactions leading to interruption or discontinuation of anti-HER2 monoclonal antibody therapy included congestive heart failure, significant decrease in left ventricular function, severe infusion reactions, and pulmonary toxicity. Careful monitoring and special therapeutic measures are required. Based on relevant ESMO and NCCN guidelines and the principles of toxicity management in expert consensus, this study will reduce the potential risks of subjects with measures such as exclusion criteria, safety monitoring, initial dose design, treatment interruption provisions, discontinuation criteria, and management of toxicity related to anti-HER2 monoclonal antibody drugs (Section 6.1.4). The management of potential infusion reactions and hypersensitivity reactions during use of KN026 as a biological product is described in Section 6.2.3.1.

Based on preclinical studies of KN046 and clinical results of marked anti-PD-L1 and anti-CTLA-4 monoclonal antibody drugs with the same target, the most common adverse reactions included tiredness, infusion reaction, diarrhea, arthralgia, rash, nausea, pruritus, and headache, most of which were mild to moderate in severity. Autoimmune-related toxicities may occur during drug treatment with anti-PD-L1 and anti-CTLA-4 monoclonal antibodies, so careful monitoring and special therapeutic measures are required. These drug-related AEs included rash and pruritus in the skin system, diarrhea and colitis in the gastrointestinal system, hypophysitis, hepatitis, endocrine disorders, pneumonia, and renal insufficiency. In this study, principles for the management of immune-related toxicities will be developed with reference to relevant ESMO and NCCN guidelines. The potential risks of subjects will be reduced with measures such as exclusion criteria, safety monitoring, initial dose design, treatment interruption provisions, discontinuation criteria, and management of immune-related toxicities (Section 6.1.4, Appendix 3). The management of potential infusion reactions and hypersensitivity reactions during use of KN046 as a biological product is described in Section 6.3.3.1. As ADCC function is retained in the structure of KN046, the potential mangement of tumor lysis syndrome is described in Section 6.3.3.2.

The Scientific Monitoring Committee (SMC) will be established to monitor the benefits and risks of the study on an ongoing basis (Section 4.4), and the study will be suspended or terminated in the event of unexpected and unacceptable adverse reactions (Section 4.2).

The study will be conducted in compliance with the protocol, GCP, the Declaration of Helsinki and other relevant regulations.

3 STUDY OBJECTIVES AND ENDPOINTS

3.1 STUDY OBJECTIVES

3.1.1 Primary objective

- **Dose escalation phase: To evaluate the MTD and/or RP2D of KN026 in combination with KN046**
- **Dose expansion phase: To evaluate the anti-tumor activity of KN026 in combination with KN046 in patients with HER2-positive solid tumors**

3.1.2 Secondary objectives

- **To evaluate the safety and tolerability of KN026 in combination with KN046;**
- **To evaluate the effect of HER2 amplification on the efficacy of KN026 in combination with KN046;**
- **To evaluate the effect of HER2 expression levels (IHC1+ vs. IHC2+ vs. IHC3+) on the efficacy of KN026 in combination with KN046;**
- **To evaluate the effect of KN026 and KN046 drug exposure levels on anti-tumor activity;**
- **To evaluate immunogenicity of KN026;**
- **To evaluate immunogenicity of KN046.**

3.1.3 Exploratory objectives

- **To evaluate the effect of PD-L1 expression, TMB, GEP, and TIL on the efficacy of KN026 in combination with KN046.**

3.2 STUDY ENDPOINTS

3.2.1 Primary endpoints

- **Dose escalation phase: Dose-limiting toxicity (DLT)**
- **Dose/cohort expansion phase: Objective response rate (ORR) and duration of response (DOR) as judged by the investigator per RECIST 1.1 criteria**

3.2.2 Secondary endpoints

- **Progression-free survival rate (PFSR) and clinical benefit rate at Month 6 and 12 judged by the investigator according to RECIST 1.1 criteria (clinical benefit rate (CBR; defined as CR, PR, or SD > 24 weeks);**
- **Overall survival rate (OS rate) at Month 6 and 12;**
- **Frequency and severity of adverse events (AEs) (NCI CTCAE 5.0); changes in vital signs, physical examinations, electrocardiograms, and safety laboratory measures;**

- **Correlation between biomarkers (HER2 IHC, HER2 amplification) and clinical efficacy parameters (ORR, CBR, PFSR, etc.);**
- **The correlation of KN026 and KN046 drug exposure (AUC$_{ss}$**, $C_{trough,ss}$, $C_{avg,ss}$, etc.) with efficacy measures (ORR, DOR, CBR, etc.);
- **Incidence and titer of anti-KN026 antibody (ADA); incidence of KN026 neutralizing anti-drug antibody (NADA) in subjects with high ADA titer;**
- **Incidence and titer of anti-KN046 antibody (ADA); incidence of KN046 neutralizing anti-drug antibody (ADA) in subjects with high ADA titer).**

3.2.3 Exploratory endpoints

- **Correlation between biomarkers (PD-L1 expression, TMB, TIL) and clinical efficacy parameters (ORR, CBR, PFSR, etc.).**

4 STUDY DESIGN

4.1 OVERALL STUDY DESIGN

This is a Phase Ib study in subjects with advanced solid tumors to evaluate the safety and tolerability, pharmacokinetics/pharmacodynamics, preliminary anti-tumor activity, immunogenicity, and the predictive effect of biomarkers of KN026 in combination with KN046.

The study will be divided into a dose escalation phase and a dose/cohort expansion phase.

The rationale for selecting the initial dose in the dose escalation phase is detailed in Section 2.1.

This study is an open-label, Phase Ib, multi-center clinical study to evaluate the efficacy and safety of KN026 in combination with KN046 in subjects with HER2-positive or HER2-expressing solid tumor.

The study is divided into 2 phases: a dose escalation phase and a dose expansion phase. The prespecified KN026 dose groups in the dose escalation period are 20 mg/kg Q2W group and 30 mg/kg Q2W group; the prespecified KN046 dose group is 3 mg/kg Q2W group (Figure 1). The adaptive dose finding method "3+3" is used in the dose escalation phase. "3+3" design allows dose increase or decrease based on a calculated decision table during subject enrollment so that MTD can be determined. After completion of the dose escalation phase, the dose limiting toxicity is determined by isotonic regression of "3+3" model. The dose closest to the target DLT is the MTD. 3 subjects will be enrolled in the first dose group (KN026 20 mg/kg Q2W + KN046 3 mg/kg Q2W), if no DLT was observed, the dosage was increased. Also, if a DLT was observed in the first three patients at a given dose level, 3 additional eligible patients were enrolled (no additional patients were required if a DLT was observed in one of six patients), if no DLT was observed, the dosage was increased. Dose escalation continued until two out of six patients in each dose cohort experienced a DLT. And an SMC will be convened after DLT observation. In addition, the first dose group may be expanded to a maximum of 12 subjects. If the second dose group meets the criterion of "escalation to higher dose", the subjects will enter the third dose group (KN026 30 mg/kg Q3W, loading dose + KN046 5 mg/kg Q3W on C1D1 and D8). If the third dose group meets the criterion of "maintain the original dose", subjects may be expanded up to 12 and the DLT observation for the entire dose escalation cohort may be ended. The recommended Phase II dose (RP2D) will be decided by the SMC after the end of dose escalation and the subjects will enter the dose expansion phase. The SMC will decide the starting dose of KN026 and KN046 in the dose escalation phase, add the intermediate dose group, or add other cohorts at a dose level based on data including safety and pharmacokinetics from previous dose groups, and/or data from other studies of KN026 and KN046.

Subjects with HER2-positive or HER2-expressing solid tumor will be enrolled in the dose escalation period. HER2-positive is defined as follows:

- HER2 IHC3+ (ASCO/CAP 2018; Wolff et al, 2018); **or**
- HER2 gene amplification (ISH+: HER2/CEP17 ratio > 2 or HER2 gene copy number > 6; or NGS: HER2 gene copy number > 6); **or**
- HER2 gene mutation (NGS method);
    - HER2 Exon 20 insertion;
    - HER2 deletion near amino acids 755-759;

- G309A, G309E, S310F, D769H, D769Y, V777L, P780-Y781insGSP, V842I, R896C;
- Non-synonymous activating mutations (or insertions and deletions) found in 2 or more samples reported in the COSMIC database

HER2 expression is defined as IHC 1+ or IHC 2+ and ISH-.

- In Part 1 of the dose expansion phase, patients with HER2-positive gastric/gastroesophageal junction cancer (GC/GEJ) who have not received systemic therapy (N: about 20-25) will be enrolled.

Subjects who have received prior HER2-targeted therapy such as trastuzumab in the dose escalation phase or expansion phase should provide tumor tissue samples collected after failure of HER2-targeted therapy for determination of HER2-positive status.

An SMC meeting will be held after the end of Part 1 of the dose expansion phase to decide whether to conduct Part 2 of the dose expansion phase and further collect efficacy data from patients with HER2-positive solid tumor. The definition of HER2-positive in Part 2 of the extension phase will be adjusted (if applicable) based on the results of Part 1.

All subjects will be required to submit slides for central laboratory review of HER2 status at screening. The testing flow of the central laboratory is shown in the following table:

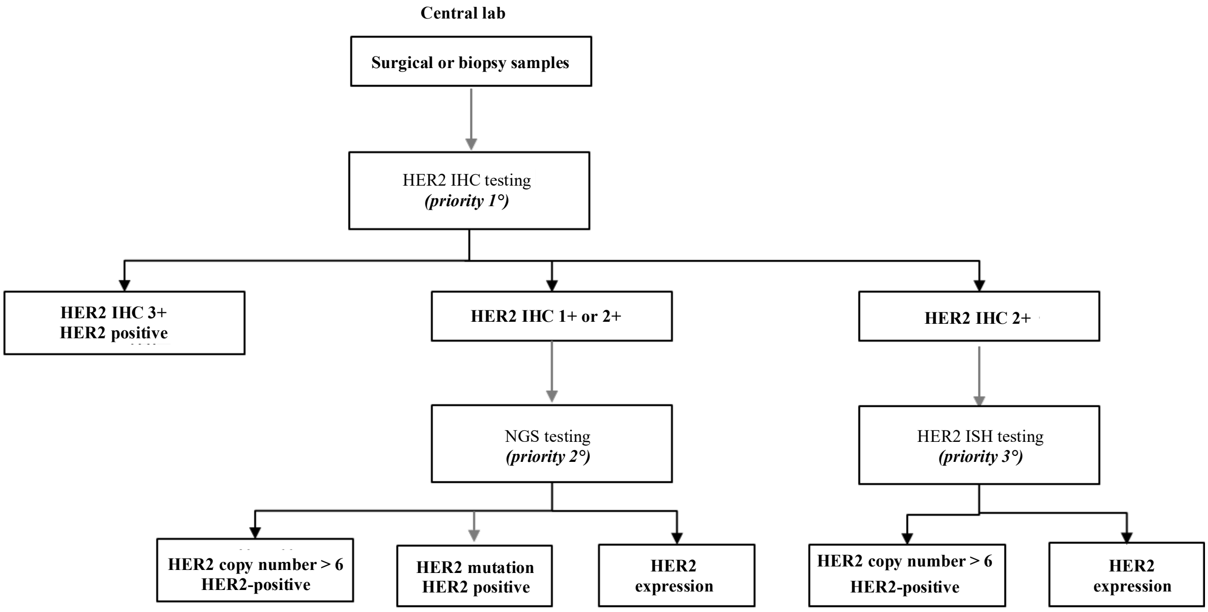

Each subject's study period includes a screening period (-28 to 0 day), a treatment period (up to 2 years of treatment; if the investigator judges that the subject is still benefiting after 2 years, continuation of treatment is allowed with the consent of the sponsor), end of treatment follow-up (within 7 days after the decision to discontinue treatment), 30-day safety follow-up, 90-day safety follow-up, and survival follow-up.

Each subject will receive KN026 in combination with KN046 per protocol until radiographic progression, intolerable toxicity, or withdrawal of consent per RECIST 1.1, whichever occurs first. After the investigator first judges the subject condition as disease progression per RECIST 1.1 criteria, if the subject is clinically stable and did not experience intolerable toxicity during the treatment, the subject is allowed to continue treatment until disease progression is confirmed. Clinical stability is defined as: Stable ECOG score, no unacceptable toxicity associated with KN046 treatment, no rapid disease progression requiring other salvage therapy, and no

emergencies requiring urgent medical intervention due to disease progression (e.g., central nervous system metastases, dyspnea due to tumor compression of the airway, spinal cord compression, etc.). The clinical, oncology, and laboratory evaluation flow of each study phase is detailed in Table 1, 2 and 3 (Evaluation Flow Chart) as well as Section 7.

During the DLT observation period (for Q2W, the DLT observation period is 28 days; for Q3W or QW, the DLT observation period is 21 days), new subjects will be allowed to be re-enrolled for replacement due to non-DLT reasons or when subjects who received the study drugs < 80% of planned dose are withdrawn.

DLT in this study is defined as AEs which are related to treatment with KN026 and/or KN046 and meet the following criteria:

- Hematological toxicity:
    - ≥ Grade 3 agranulocytosis lasting more than 7 days;
    - Febrile neutrophil decreased;
    - ≥ Grade 3 thrombocytopenia with bleeding tendency or requiring platelet transfusion;
    - ≥ Grade 4 hematologic toxicity, except for Grade 4 lymphopenia (alone).
- Non-hematological toxicity:
    - Drug-related symptomatic heart failure (NYHA Grade 3 or higher); or ≥ 15% absolute decrease in LVEF from baseline;
    - ≥ Grade 3 raised serum creatinine;
    - ≥ Grade 3 total bilirubin increased;
    - Grade 3 ALT and/or AST increased lasting more than 7 days;
    - Grade 4 ALT and/or AST increased;
    - ≥ Grade 2 central nervous system toxicity;
    - ≥ Grade 3 cardiotoxicity;
    - Grade ≥ 3 asymptomatic serum pancreatic amylase or lipase increased lasting more than 14 days;
    - Serum pancreatic amylase or lipase increased accompanied by clinical symptoms and signs and requiring medical intervention;
    - Other ≥ Grade 3 nonhematologic toxicities, except:
        - Any grade of alopecia;

- Transient (≤ 24 hours) Grade 3 fatigue, local reactions, headache, nausea, and vomiting that resolved to ≤ Grade 1;
- Grade 3 diarrhea, Grade 3 skin toxicity, or Grade 3 liver function-related parameter increased (ALT, AST, or GGT) that resolve to ≤ Grade 1 within 7 days after medical intervention;
- Grade 3 infusion-related reactions that resolved within 6 hours of medical intervention;
- Grade 3 flu-like symptoms or fever that are transient (≤ 6 hours) after medical intervention;
- A single abnormality in the laboratory test that is not clinically significant and resolves to ≤ Grade 1 within 7 days of appropriate medical intervention;
- Tumor recurrence, manifested with pain, local irritation, or local rash at the site of a known or suspected tumor.

4.1.1 Definition of end of study

The end of the study is defined as 1 year after the last dose for all subjects, or all subjects complete all visits, whichever occurs first.

4.1.2 Premature discontinuation of study

The study will be prematurely discontinued if:

- New information suggests an unacceptable risk-benefit ratio for the study drugs;
- The sponsor considers to terminate the study in the opinion that it is no longer appropriate to continue the study from a medical and ethical point of view;
- It is not possible to complete the study within an acceptable time period due to poor enrollment of subjects; If it is not possible to complete the study within an acceptable time period due to poor enrollment in the dose expansion phase, the sponsor and Alphamab may decide to discontinue the study;
- The sponsor decides to discontinue the development of the study drugs.

If the study is terminated prematurely, the subject should receive a visit as soon as possible and complete the visit as required by Section 7.1.3. For the sake of protecting the subjects, the investigator may be instructed to conduct additional visits.

After termination of the study, the regulatory authorities and the Independent Ethics Committee (IEC)/Institutional Review Board (IRB) will be notified in accordance with applicable regulations.

Regulatory authorities may also request suspension or termination of the whole study.

4.2 INTERIM ANALYSIS

The interim analysis will be performed at the end of the dose escalation phase (defined as the completion of enrollment of all subjects in the dose escalation phase and the completion of DLT observation during the DLT observation phase), and at the end of the dose expansion phase (defined as "the completion of enrollment and the completion of at least 2 post-baseline oncology evaluations").

The SMC may request additional interim analyses to analyze safety and/or efficacy data.

4.3 SCIENTIFIC MONITORING COMMITTEE (SMC)

To ensure the safety and risk-benefit ratio of subjects throughout the study, a Scientific Monitoring Committee (SMC) will be established to periodically review safety data and clinical efficacy. The SMC includes fixed members from the sponsor and Alphamab (including, but not limited to, Head of Early Development, Medical Director, and Biostatistics Expert), participating investigators (if applicable), and external experts (if applicable).

The SMC will evaluate safety data, make a decision on treatment-related dose-limiting toxicities (DLTs), and make an advice on dose escalation or discontinuation of enrollment, but the sponsor has the final decision priority. The SMC will continuously monitor all safety information of subjects (frequency of monitoring will be defined in the SMC charter), make a decision on continuation, modification, or discontinue the entire study or a treatment cohort, and make an advice on the addition of subsequent cohorts. The SMC may adjust the frequency of meetings as appropriate during the course of the study.

Specific working procedures are described in the SMC charter, which will be established prior to study enrollment.

5 STUDY POPULATION

Subjects who meet all the inclusion criteria and none of the exclusion criteria are eligible to be enrolled in this study. Before any study evaluation beyond the subject's routine medical care, the investigators should ensure that the subject or subject's legal representative has provided the written informed consent.

5.1 INCLUSION CRITERIA

I01. Subjects are able to understand the Informed Consent Form (ICF), voluntarily participate in the study and sign the ICF;

I02. Subjects ≥ 18 and ≤ 75 years of age, male or female, on the day of signing the informed consent form;

I03. Histologically or cytologically confirmed as patients with metastatic or locally advanced unresectable HER2-positive or HER2-expressing solid tumor. HER2-positive is defined as follows:

- HER2 IHC 3+; **or** HER2 gene amplification: In situ molecular hybridization ISH method (fluorescence in-situ hybridization, FISH; dual-color silver-enhanced in situ hybridization, DSISH) is used to confirm HER2 gene amplification (HER2/CEP17 ratio > 2.0), or NGS HER2 copy number > 6; **or** NGS HER2 gene mutation;

  Note: HER2 gene mutations include: HER2 Exon 20 insertion; HER2 deletion near amino acid 755-759; G309A, G309E, S310F, D769H, D769Y, V777L, P780-Y781insGSP, V842I, R896C; non-synonymous activating mutations (or insertions and deletions) found in 2 or more samples reported in the COSMIC database

  Note: HER2 status at enrollment may be confirmed by a local laboratory or a central laboratory and used for eligibility evaluation, and if HER2 status is confirmed by a local laboratory, pathological slides must be provided to the sponsor-designated central laboratory for review; local and central laboratories should not only report HER2 gene amplification, but also indicate HER2/CEP17 values. If the patient has received HER2-targeted therapy in the front line and disease has progressed, a post-progression tumor tissue sample is required to confirm HER2 status;

  Note: For colorectal cancer, **wild-type RAS** is required;

- HER2 expression is defined as IHC 1+; or IHC 2+ and no amplification of HER2 gene is confirmed by ISH method of in situ molecular hybridization (fluorescence in situ hybridization, FISH; dual-color silver-enhanced in situ hybridization, DSISH);

I04. Prior anti-tumor therapy requires the following:

- HER2-positive GC/GEJ: Metastatic or locally advanced unresectable, **no** prior systemic therapy has been **received**, and relapse and metastasis have existed more than 12 months from the end of neoadjuvant/adjuvant chemotherapy;
- HER2-positive or HER2-expressing GC/GEJ: **Having received** ≥ 1 prior systemic therapy for metastatic or locally advanced unresectable GC/GEJ and the disease has progressed, and front-line systemic therapy includes at least platinum- or fluorouracil-based chemotherapy with or without trastuzumab; subjects who have relapsed within 12 months after the end of neoadjuvant/adjuvant chemotherapy are considered as failing first-line therapy;

- HER2-positive or HER2-expressing non-GC/GEJ GI: **Having received** ≥ 1 prior systemic therapy for metastatic or locally advanced unresectable tumor and the disease has progressed; front-line systemic therapy for ESCC and mCRC includes at least platinum in combination with fluorouracil or taxane-based chemotherapy; mCRC requires ≥ 2 lines of systemic therapy for metastatic or locally advanced unresectable tumor with disease progression; subjects who have relapsed within 12 months after the end of neoadjuvant/adjuvant chemotherapy or radical chemoradiotherapy are considered as failing the first-line treatment;

I05. At least 1 measurable lesion at baseline per RECIST 1.1 criteria. If a subject has only 1 measurable lesion at baseline, the lesion area must have not received prior radiotherapy, or there is evidence of significant progression of the lesion after the end of radiotherapy;

I06. ECOG score 0 or 1 (Appendix 5);

I07. LVEF ≥ 50%, determined by echocardiography (ECHO); multiple uptake gated acquisition (MUGA) scan will be used only in the absence of ECHO, and baseline and subsequent follow-up methods will be the same;

I08. NYHA functional class 0-1;

I09. Liver function meets the following criteria within 7 days prior to the first dose:

- Total bilirubin ≤ 1.0 × ULN (Gilbert's syndrome or total bilirubin of subjects with liver metastasis ≤ 1.5 × ULN);
- Transaminase (ALT/AST) ≤ 1.5 × ULN (≤ 3 × ULN for subjects with liver metastases);

I10. Renal function within 7 days prior to the first dose: Serum creatinine ≤ 1.5 × ULN and serum creatinine clearance ≥ 60 mL/min (calculated according to the Cockcroft-Gault formula);

I11. Bone marrow function meets the following criteria within 7 days prior to the first dose:

- Hemoglobin ≥ 9.0 g/dL;
- Absolute neutrophil count ≥ 1.5 × 10^9/L;
- Platelet count ≥ 100 x 10^9/L;
- INR or PT ≤ 1.5 × ULN and aPTT ≤ 1.5 × ULN;

I12. TSH is normal; if TSH is abnormal, total T3 or free T3, and free T4 should be within the normal range;

I13. Life expectancy ≥ 3 months;

I14. Female subjects of childbearing potential or male subjects with a partner of childbearing potential agree to use highly effective contraception (see Appendix 4) from 7 days before the first dose until 24 weeks postdose. Female subjects of childbearing potential must have a negative serum pregnancy test within 7 days before the first dose;

I15. Subjects are able and willing to comply with protocol-scheduled visits, treatment regimens, laboratory tests, and other study-related procedures.

5.2 EXCLUSION CRITERIA

E01. Subjects with untreated active brain metastases or with meningeal metastases; if the subject's brain metastases are treated and the metastasis condition is stable (brain

imaging at least 4 weeks prior to the first dose shows stable disease and there are no new neurological symptoms, or neurological symptoms have returned to baseline), and there is no evidence of new or enlargement of the original brain metastases, enrollment is allowed;

E02. Decrease in LVEF to < 45% or absolute decrease in LVEF > 15% during prior HER2-targeted therapy;

E03. Prior cumulative doses of anthracyclines exceeding doxorubicin, liposomal doxorubicin or other anthracyclines by > 320 mg/m^2;

E04. Having participated in any other interventional study within 28 days before the first dose;

E05. Having received other anti-tumor therapy within 28 days prior to the first dose;

E06. Having received major surgical treatment (such as major abdominal, transthoracic surgery; excluding diagnostic aspiration or peripheral vascular access replacement) within 28 days prior to the first dose;

E07. Radical radiotherapy within 3 months prior to the first dose; palliative radiation therapy within 2 weeks prior to the first dose is allowed, the radiation dose meets the diagnostic and treatment criteria for local palliative treatment, and the radiation coverage is less than 30% of the bone marrow area;

E08. Previous treatment with immune checkpoint blockers or T-cell costimulatory drugs, etc., including but not limited to immune checkpoint blockers such as PD-1, PD-L1, CTLA4, LAG3, therapeutic vaccines, etc. (Only for subjects included in DLT observation, subjects enrolled in the expansion phase are allowed to have received prior immune checkpoint blockers);

E09. Having received systemic corticosteroids (≥ 10 mg/day prednisone or equivalent dose of other corticosteroids) or immunosuppressive therapy within 14 days prior to the first dose in this study; except inhaled or topical corticosteroids, or physiologic replacement doses of corticosteroids for adrenal insufficiency;

E10. Having received live vaccines (including live attenuated vaccines) within 28 days before the first dose;

E11. Previous or current interstitial pneumonia/lung disease;

E12. Having a history of or current autoimmune diseases, including, but not limited to, Crohn's disease, ulcerative colitis, systemic lupus erythematosus, sarcoidosis, Wegener's syndrome (granulomatosis with polyangiitis, Graves' disease, rheumatoid arthritis, hypophysitis, uveitis), autoimmune hepatitis, systemic sclerosis (scleroderma, etc.), Hashimoto's thyroiditis (refer to the following exceptions), autoimmune vasculitis, autoimmune neuropathy (Guillain-Barre syndrome), etc. With the following exceptions: Type I diabetes mellitus, hypothyroidism with stable hormone replacement therapy (including hypothyroidism caused by autoimmune thyroid disorder), psoriasis or vitiligo that does not require systemic treatment;

E13. Having other malignancies within 5 years before the first dose, except cured skin squamous cell carcinoma, basal cell carcinoma, non-muscle invasive bladder cancer, localized low-risk prostate cancer (defined as stage ≤ T2a, Gleason score ≤ 6, and PSA ≤ 10 ng/mL (as measured) at diagnosis of prostate cancer, the patients had received curative treatment and no prostate-specific antigen (PSA) biochemical recurrence), and in-situ cervical/breast cancer;

E14. Having a history of uncontrolled complications including but not limited to:

- Active HBV or HCV infection;
- Known history of HIV infection or AIDS;
- Active tuberculosis;
- Active infection, or systemic use of anti-infective drugs within 28 days before the first dose of KN046;
- Uncontrolled hypertension (BP ≥ 150/95 mmHg at rest), symptomatic cardiac insufficiency (NYHA II-IV), unstable angina or myocardial infarction within 6 months, or risk of QTc prolongation or arrhythmia (baseline QTc > 470 msec <Fridericia correction>, refractory hypokalemia, long QT syndrome, atrial fibrillation with heart rate > 100 bpm at rest, or severe valvular disease, etc.);

E15. Toxicities with prior anti-tumor therapy did not recover to CTCAE ≤ Grade 1 (NCI-CTCAE v5.0) or baseline, with the exception of alopecia and skin pigmentation (any grade);

E16. Prior history of allogeneic bone marrow or organ transplantation;

E17. Prior history of allergic reaction, hypersensitivity reaction, and intolerance to antibody drugs; history of significant allergy to drugs (e.g., severe allergic reactions, immune-mediated hepatotoxicity, immune-mediated thrombocytopenia or anemia);

E18. Pregnant or breastfeeding women;

E19. Other conditions that, in the opinion of the investigator, would affect the safety or compliance with the study drug, including but not limited to moderate to large pleural/ascites/pericardial effusion, difficult-to-correct pleural/ascites/pericardial effusion, psychiatric disorders, etc.

5.3 SUBJECT WITHDRAWAL CRITERIA

5.3.1 Discontinuation of study drugs

Subjects will be required to discontinue treatment with KN046 and/or KN026 if:

- Disease progressione judged as per RECIST v1.1 criteria (Note: If the subject's ECOG performance status remains stable and the investigator determines that the subject will benefit from continuous treatment with KN046 and KN026, treatment with KN046 and KN026 will be allowed after first disease progression judged per RECIST v1.1 criteria, as detailed in Section 6.1.3);
- Significant clinical deterioration (clinical progression), defined as new or significant worsening of existing symptoms that are considered clinically significant by the investigator (if disease progression is not met per RECIST 1.1 criteria, the subject needs to continue oncology evaluation);
- Treatment failure requiring urgent use of other anti-tumor drugs (if applicable);
- Unacceptable toxicity (Section 6.1.4) (if study drug is discontinued due to unacceptable toxicity, the subject needs to continue oncology evaluation);

- Pregnancy;
- Concomitant use of prohibited concomitant medications and procedures as specified in Section 6.2.2, and its consequence as discontinuation of KN046 and KN026;
- Withdrawal of consent to continue treatment with KN046 and KN026 (if the subject withdraws consent, and the disease progression is not met per RECIST 1.1 criteria, the subject will be asked and required to continue oncology evaluation);
- Noncompliance.

5.3.2 Withdrawal from study

Subjects are free to discontinue the study at any time without giving reasons. If the subject withdraws from the study, the evaluation plan required at the last visit (EOT visit) should be implemented whenever possible (see Section 7.1.3), with emphasis on the most relevant evaluations. In all cases, the eCRF page records for the EOT visit must be completed whenever possible. In event of withdrawal from the study, subjects will be asked whether to continue safety and long-term follow-up, The long-term follow-up includes collection of survival and subsequent anti-tumor therapy data.

Subjects must be withdrawn from the study if:

- Withdrawal of consent and refusal to continue the study (if the subject withdraws consent, it must be clearly stated whether the subject also refuses to continue post-treatment follow-up);
- Participation in other interventional clinical studies during the study; in this case, the subject will continue to be followed for survival;
- Lost to follow-up.

If the data are not further collected because the subject completely withdraws from the study or does not return to the visit, the investigator must determine the primary reason for the subject's withdrawal as completely and accurately as possible and record this information on the eCRF page. For subjects who are lost to follow-up, the investigator should document in the original document the steps taken to contact the subject to confirm that "he/she has performed his/her duty", such as telephone date and registered letter. For subjects who are lost to follow-up, public records may be consulted for survival status information (alive/dead) if permitted by local laws.

6 STUDY DRUGS AND SUBJECT TREATMENT

The use of study drugs in the dose escalation and dose/cohort expansion phases of the study is summarized in Table 5. A detailed description of medications is provided in Section 6.1.3.

Table3 Dosing plans of KN046 and KN026 in study phases

	Study drug	Preset dose group (mg/kg)	Route of administration	Preset dosing regimen[2]
**Dose escalation phase**	KN046	3	IV	Day 1/14-day cycle
	KN046	5	IV	Day 1/21-day cycle
	KN026	20	IV	Day 1/14-day cycle
	KN026	20	IV	Day 1 and 8 (subsequent)<br>Day 1/14-day cycle
	KN026	30	IV	Day 1 and 8 (subsequent)<br>Day 1/21-day cycle
**Dose expansion phase**	KN046	RP2D	IV	Based on the results of the dose escalation phase
	KN026	RP2D	IV	Based on the results of the dose escalation phase

Note: [1]The dose administered in the dose expansion phase will be determined with reference to the analysis results of the dose escalation phase and/or the results of other KN046 and KN026 studies;

6.1 INVESTIGATIONAL PRODUCT

KN046 and KN026 are study drugs in this study.

6.1.1 Drug dosage form and strength

The dosage form of KN046 is IV injection for single use, with a strength of 40 mg/1.6 mL/vial or 300 mg/12 mL/vial.

The dosage form KN026 is lyophilized powder for injection for single use, with a strength of 50 mg/vial, or IV injection, with a strength of 325 mg/13 mL/vial.

6.1.2 Drug preparation

6.1.2.1 Preparation of KN046

KN046 is predetermined to be administered once every 2 weeks (Q2W) by intravenous infusion.

The dose volume of each group will be calculated based on body weight. For example, for a subject with a weight of 70 kg enrolled in the 1.0 mg/kg group at a dose of 70 mg, the dose volume will be 70 mg/40 mg * 1.6 mL = 2.8 mL. Aseptically withdraw 2.8 mL from two vials of KN046 drug solution and then inject into a 200 mL sterile **5% dextrose injection** bag that has previously withdrawn with a volume of 2.8 mL. The drug solution should be mixed gently and thoroughly before infusion. The infusion time should be controlled within 90-120 minutes. An appropriate amount of sterile **5% dextrose injection** should be rinsed at the end of infusion.

Visible foreign matters and color should be examined macroscopically before drug infusion. KN046 cannot be administered as an intravenous bolus and short infusion. After drug preparation, a maximum error of not more than ± 10% of the theoretical dose is allowed for the actual dose.

6.1.2.2Preparation of KN026

KN026 will be administered every 2 weeks (Q2W) by intravenous infusion.

The dose volume will be calculated based on body weight. The preparation of the drug solution must strictly follow the principle of sterility. For example, for a subject with a weight of 70 kg enrolled in the 20 mg/kg group, the dose will be 1400 mg. For lyophilized powder, 28 vials are required; for IV injection (325 mg/13 mL/vial), 56 mL of solution is required to be withdrawn from 5 vials of drug product. For lyophilized powder, add 2 mL of water for injection to each vial of KN026 and allow to stand at room temperature until complete dissolution (reconstituted drug product must be diluted within 4 hours); then dilute with **normal saline** as needed to prepare a 250 mL solution; finally, invert to mix the solution and then intravenously infuse with an infusion filter (the diluted drug product in the infusion bag must be used within 24 hours). For IV injection, the solution for injection will be diluted with **normal saline** and prepared into a 250 mL solution.

Visible foreign matters and color should be examined macroscopically before drug infusion. KN026 cannot be administered as an intravenous bolus and short infusion. After drug preparation, a maximum error of not more than ± 10% of the theoretical dose is allowed for the actual dose.

6.1.3 Dose and method of administration of study drugs

It is predetermined that subjects in this study will receive KN046 intravenously every 2 weeks for 90-120 minutes.

It is predetermined that subjects in this study will receive KN026 intravenously every 2 weeks, with the infusion time for the first dose of 90 (± 15) minutes. If no infusion-related AEs develop during the first infusion, the subsequent infusion time can be adjusted to 60 minutes (45-75 minutes).

The predefined doses administered for the dose escalation phase and dose expansion phase are presented in Table 3. The rationale for dose selection in the dose escalation phase is presented in Section 2.1.

Body weights of subjects will be measured prior to each KN026 and/or KN046 dose, and the administered doses of KN046 and KN026 will be calculated. Each subject will receive the planned dose of KN046 and KN026 according to the protocol until radiographic disease progression, significant clinical deterioration (clinical progression), unacceptable toxicity (Section 6.1.4), 2 years of treatment (only for KN046), or other conditions meeting discontinuation criteria of KN046 and KN026 or study withdrawal criteria (Sections 5.3.1 and 5.3.2). Refer to Section 6.1.4 and Appendix 3 for the conditions with a need to adjust the mode of administration of KN046 and KN026 (e.g., change in infusion rate, dose delay, resumption of dosing). After 2 years of treatment with KN046, if the subject is judged to be still benefiting from the treatment by the investigator, the treatment will be allowed to continue with the consent of the sponsor and Alphamab.

Subsequent imaging evaluations should be performed approximately 6 weeks (no earlier than 4 weeks and no later than 8 weeks) after the initial determination of PD after the first occurrence of progressive disease (PD), to determine whether the tumor has shrunk or PD is ongoing (confirmed PD). Refer to Section 7.2.2.3 for procedures for confirming PD. If the subject is clinically stable until radiographic disease progression is confirmed, the subject will be allowed to continue treatment with KN046 after discussion with the Medical Monitor and documentation.

Clinical stability is defined as follows:

- Stable ECOG score;
- Absence of unacceptable KN046-related toxicities (Section 6.1.4);
- Absence of fast disease progression that needs an anti-tumor salvage therapy;
- No acute symptoms with disease progression leading to emergencies requiring urgent medical intervention (e.g., metastases to central nervous system, dyspnea due to tumor compression of the airways, or spinal cord compression).

Once radiographic progression is confirmed, subjects need to discontinue KN046 treatment in principle. if the investigator determines that the subject still has clinical benefit, after discussion and documentation with the Medical Monitor, it is allowed to determine the tumor progresses according to the iRECIST criteria (see Appendix 2), and the doctor will manage subjects according to the overall benefit. In this case, subjects should be fully informed and be aware of other possible treatment options.

6.1.4 Dose modifications for study drugs

6.1.4.1Dose modification of KN046

Whenever a subject experiences protocol-specified dose-limiting toxicities (Section 4.1.1) and ≥ Grade 3 toxicities related to KN046 treatment (except for laboratory abnormalities that are not clinically significant or do not meet the criteria for AEs listed below), KN046 is required to be interrupted until treatment-related toxicity resolves to ≤ Grade 1. Guidelines for dose modification of KN046 are listed in Table 4.

Table4 Guidelines for dose modification of KN046

Toxicity	Grade	Dose interruption	Criteria for resumption of dosing	Dose after resumption of dosing	Drug discontinuation criteria
**Hematological toxicities**	1、2	No	Not applicable	Not applicable	Not applicable
	3<br>*Exclude Grade 3 agranulocytosis alone (Grade 3 agranulocytosis alone does not require dose interruption)*	Yes	Toxicity resolves to ≤ Grade 1 within 12 weeks of last dose	Original dose	Toxicity does not resolve to ≤ Grade 1 within 12 weeks after last dose<br>*Permanent discontinuation may be considered if toxicity meets the criteria for SAEs.*
	4	Yes	Not applicable	Not applicable	Permanent discontinuation
**Non-hematological toxicities**<br>Note: The following toxicities will be treated as Grade 1 toxicities.<br>• Any grade alopecia<br>• Grade 2 asthenia<br>• Laboratory abnormalities that are not clinically significant or do not meet the criteria for AEs	1	No	Not applicable	Not applicable	Not applicable
	2	Consider dose interruption if toxicity persists	Toxicity resolves to ≤ Grade 1 within 12 weeks of last dose	Original dose	Toxicity does not resolve to ≤ Grade 1 within 12 weeks after last dose
	3	Yes	Toxicity resolves to ≤ Grade 1 within 12 weeks of last dose	Original dose	Toxicity does not resolve to ≤ Grade 1 within 12 weeks after last dose<br>*Permanent discontinuation may be considered if toxicity meets the criteria for SAEs.*
	4	Yes	Not applicable	Not applicable	Permanent discontinuation

If KN046 treatment-related toxicity does not recover to Grade 0-1 within 12 weeks of the last dose, KN046 will be discontinued after discussion with the Medical Monitor.

Rarely, Grade 2 treatment-related toxicities are allowed to continue to resume KN046 administration, e.g., treatment-related hypothyroidism, type I diabetes mellitus, and other medical conditions with appropriate alternative therapies, in which case the subject will restart the KN046 treatment after the symptoms are controlled with an alternative therapy and after the discussion between the investigator and the Medical Monitor.

For treatment-related toxicities requiring hormonal intervention therapy, KN046 treatment may be restarted when the dose of the hormone needs to be tapered to ≤ 10 mg/day prednisone (or equivalent of other corticosteroids). If the dose of hormone cannot be reduced to ≤ 10 mg/day prednisone (or equivalent of other corticosteroids) within 12 weeks after the last dose, the investigator and the Medical Monitor need to discuss and decide whether to restart the KN046 treatment.

After resumption of KN046 treatment, permanent discontinuation of KN046 treatment is required if the same type of treatment-related toxicity reappears.

Permanent discontinuation of KN046 treatment may also be considered for the safety of subjects in some cases of KN046 treatment-related toxicities, including but not limited to:

- Grade 3 immune-related pneumonia;
- Recurrence of Grade 2 immune-related pneumonia lasting more than 4 weeks after active treatment;
- Grade 2 immune-related central nervous system toxicity lasting more than 4 weeks after active treatment;
- Grade 3 immune-related colitis;
- Grade 3 immune-related uveitis;
- Grade 3 immune-related hepatitis with ALT/AST ≥ 5 x ULN and/or total bilirubin ≥ 3 x ULN;
- Grade 3 immune-related nephritis, and renal insufficiency;
- Grade 1 immune-related myocarditis.

The rules for the management of KN046-related irAEs are provided in Appendix 3.

As a biological product, KN046 retains ADCC activity. Refer to Section 6.2.3.2 for the management of potential tumor lysis syndrome.

6.1.4.2 Dose modification of KN026

Whenever a subject experiences a protocol-specified ≥ Grade 3 toxicities related to KN026 treatment (except for laboratory abnormalities that are not clinically significant or do not meet the criteria for AEs listed below), KN026 is required to be interrupted until treatment-related toxicity resolves to ≤ Grade 1. Guidelines for dose modification of KN026 are listed in Table 7. The investigator may appropriately manage it with reference to the recommendations in the table.

Table 5 Guidelines for dose modification of KN026

Toxicity related to study drug	Severity	Management recommendations
Cardiotoxicity	≥ 15% absolute decrease in LVEF from baseline	Interrupt and confirm again before the next theoretical dose time;<br>Discontinue the treatment if the absolute decrease is still ≥ 10% from baseline;
	≥ 10% absolute decrease in LVEF from baseline, and <50% LVEF	
	LVEF recovers to > 50% and the absolute decrease from baseline is < 15% before the next theoretical dose time	Restart the treatment
	Decrease in LVEF lasting more than 8 weeks or > 3 times of cardiomyopathy at this dose	Permanent discontinuation
Drug-related laboratory abnormalities	Grade 3 leukopenia or neutropenia	Continue the treatment
	Grade 2 ALT, AST, or TBIL increased in patients with normal baseline ALT, AST, or TBIL;<br>Grade 3 or 4 ALT, AST, or TBIL increased lasting < 7 days in patients with baseline ALT, AST, or TBIL > ULN	Dose interruption<br>Resume the treatment if ALT, AST, TBIL decreased to ≤ Grade I.
	Grade 3 or 4 ALT, AST, or TBIL increased in patients with normal baseline ALT, AST, or TBIL; Grade 3 or 4 ALT, AST, or TBIL increased lasting ≥ 7 days in patients with baseline ALT, AST, or TBIL > ULN	Permanent discontinuation
	Any other ≥ Grade 4 drug-related AE or laboratory abnormality	Permanently discontinue the treatment with the following exceptions:<br>1. Individual ≥ Grade 4 amylase or lipase abnormality, which is not related to symptoms or clinical manifestations of pancreatitis, and can be resolved to < Grade 4 within 1 week;<br>2. Individual ≥ Grade 4 electrolyte imbalance/abnormality without clinical sequelae, which can be recovered by supplementation/or appropriate treatment within 72 hours;<br>3. Alkaline phosphatase increased to ≥ Grade 4 in patients with bone metastases, and no other relevant organ function changes judged by the investigator.
Infusion reactions	Grade I infusion reaction<br>Mild transient reaction; infusion interruption not required; intervention not required.	Continue the treatment and monitor closely;<br>50 mg diphenhydramine or 650 mg paracetamol may be administered 30 min predose;
	Grade II infusion reaction<br>Treatment interruption or infusion required, and prompt symptomatic treatment (e.g., antihistamines, NSAIDs, anesthetics, IV fluids).	1. Treatment interruption or infusion is required (intravenous injection of diphenhydramine 50 mg or 650 mg paracetamol); according to symptoms, steroids or bronchodilator treatment are given;<br>2. Continue the treatment, but decrease the infusion rate by 50% and closely monitor;

		if no more severe complications occur immediately after 30 min, the infusion rate can be increased to 100%;<br>4. Discontinue the treatment if symptoms reappear, intravenously inject with diphenhydramine 50 mg, and closely monitor until resolved.
	Grade III or IV infusion reaction	1. Discontinue the treatment, if required, bronchodilator drugs can be used: epinephrine 0.2-1 mg (1:1000), subcutaneous injection; or 0.1-0.25 mg (1:10,000), slow intravenous injection; or diphenhydramine 50 mg and methylprednisolone 100 mg intravenous injection;<br>2. Closely monitor until it is confirmed no recurrence. The investigators should follow their institutional guidelines for the treatment of allergic reactions and may give symptomatic treatment (e.g., oral antihistamines, or hormones) in the event of late onset of allergic symptoms (e.g., local or systemic skin pruritus 1 week after treatment).
Other AEs	First occurrence of other Grade 3 AEs	Interrupt the treatment, and resume the treatment if reduced to ≤ Grade I.
	Second occurrence of the same Grade 3 AE	Permanent discontinuation
	Grade 3 AEs that cannot recover to ≤ Grade 2 within 7 days or ≤ Grade 1 within 14 days	Permanent discontinuation
	Grade 4 AEs	Permanent discontinuation

Once the infusion rate of the study drug has decreased by 50% due to an infusion-related reaction, this rate must be maintained for all subsequent infusions. In the event of Grade 2 or 3 infusion reactions, 50 mg diphenhydramine or 650 mg paracetamol will be administered 30 min prior to subsequent doses for pretreatment.

For treatment-related toxicities requiring hormonal intervention therapy, KN026 treatment may be restarted when the dose of the hormone needs to be tapered to ≤ 10 mg/day prednisone (or equivalent of other corticosteroids). If the dose of hormone cannot be reduced to ≤ 10 mg/day prednisone (or equivalent of other corticosteroids) within 12 weeks after the last dose, the investigator and the Medical Monitor need to discuss and decide whether to restart the KN026 treatment.

After resumption of KN026 treatment, permanent discontinuation of KN026 treatment is required if the same type of treatment-related toxicity reappears.

Permanent discontinuation of KN026 treatment may also be considered for the safety of subjects in some cases of KN026 treatment-related toxicities, including but not limited to:

- Congestive cardiac failure;
- Left ventricular function significantly decreased;

- Serious infusion reactions, including serious allergic reactions, angioedema, interstitial pneumonia or acute respiratory distress syndrome;
- Pulmonary toxicity, including dyspnea, pneumonitis, noninfectious pneumonitis, pleural effusion, acute pulmonary edema, and insufficiency;
- Cumulatively, medication delay for more than 8 weeks or interruption for more than 3 times.

6.1.5 Treatment assignment

The subject will be given a subject number after signing the ICF and be given a treatment assignment number when receiving KN046 and KN026. Subject numbers and treatment assignment numbers will be developed from small to large in order of dates. Each of subject numbers and treatment assignment numbers could not be reassigned to other subjects once it is determined.

6.1.6 Drug packaging and labeling

KN046 is aseptically filled in a neutral borosilicate colorless glass vial, and sealed with a halobutyl rubber stopper and an aluminum-plastic cap. Each 2 mL vial contains 1.6 mL of KN046 drug solution. Each vial is packed in cardboard boxes.

KN026 is aseptically filled in a neutral borosilicate colorless glass vial, and sealed with a halobutyl rubber stopper and an aluminum-plastic cap. Each 10 mL vial contains ili50 mg of KN026 lyophzed powder. Each vial is packed in cardboard boxes.

The bottle label mainly contains the following information: protocol number, drug content and strength, batch number, expiry date, storage conditions, and dosage and administration. The contents of the label will meet the requirements of current regulations.

6.1.7 Drug storage, transportation and shelf life

Study drugs should be dispensed to the study site only after receipt of the requisition form in accordance with relevant regulations and the provisions of the sponsor. The drugs should be used in accordance with the following procedures. KN046 and KN026 may not be given until subjects have been enrolled in this trial. Only authorized site personnel can supply or manage the study drugs. In order to ensure the safety of the subjects and to infuse the drugs according to the regimens, the subjects must return to the study site for each dose, and should not carry and use the drugs themselves. The study drugs must be stored in a secure area that is accessible only by the investigator and authorized study site personnel and meets the storage requirements of the study drugs at 2-8°C.

6.1.8 Drug quantity management

The investigator or authorized drug administrator is responsible for the quantity verification, dispensing and record maintenance of the study drugs. In accordance with relevant regulatory requirements, the investigator or site designee must maintain a drug accountability record throughout the study. It includes the quantity of study drugs received from the sponsor and the

quantity supplied to the subjects. After completion of the study, all remaining vials and unused KN046 and KN026 drugs will be registered and destroyed on site or at an independent center with written consent from the sponsor.

6.1.9 Assessment of compliance

Clinical research associates verify compliance against body weight, group, and other medical records.

6.1.10 Occupational safety

KN046 and KN026 do not pose an occupational safety risk to site staff under normal compounding and dosing conditions.

6.2 CONCOMITANT MEDICATIONS AND NON-DRUG THERAPIES

6.2.1 Permitted medications

The investigator may administer concomitant medications or treatments according to medical standards from the perspective of the subject's disease treatment needs. All concomitant medications will be recorded on the eCRF form, including drug treatment (e.g., prescriptions, over-the-counter drugs, herbal supplements, intravenous injection medications, and liquid drugs), and important non-drug therapies (including physical and blood transfusion therapies). Concomitant medications from 28 days to 90 days prior to first dose, medication modifications during the study, and treatments received due to KN046- and KN026-related SAEs after the 90-day safety follow-up visit should be recorded in the eCRF form, including dose level, dose regimen, route, indications, and start and end time of concomitant medications.

Palliative radiotherapy to bone (e.g., local radiotherapy to relieve bone pain, or to prevent the risk of fracture due to lytic lesions) is allowed in this study, but lesions in the area where palliative radiotherapy to bone is required are not selected as target lesions and palliative radiotherapy is not intended to treat tumors. Disease progression should be judged based on RECIST 1.1 criteria rather than the need for palliative radiotherapy to bone.

6.2.2 Prohibited medications and procedures

Medications not to be used concomitantly during the course of the study include:

- Other anti-tumor drugs (e.g., cytotoxic drugs, radical radiotherapy or radiotherapy for the treatment of tumors, immunotherapy, cytokine therapy [except erythropoietin]);
- Systemic hormones (except for the treatment of immune-related adverse reactions, prophylaxis requiring prior to contrast-enhanced CT angiography due to contrast allergy, alternative therapy at physiological doses, topical hormones [topical, nasal, ocular, inhaled]);
- Immunosuppressants;

- Traditional Chinese medicine with anti-tumor indications approved by China Food and Drug Administration (CFDA);
- Other investigational drugs;
- Major surgery (except for diagnostic puncture and peripheral venous catheterization, surgical treatment is the medical routine after the tumor is down-staged);
- RANKL inhibitors (e.g., denosumab) for treatment of bone metastases;
- Botanical drugs that stimulate the immune system (e.g., mistletoe extracts).

If prohibited concomitant medications or procedures are required during the course of the study, subjects should discontinue treatment with KN046 and KN026 (see Section 5.3.1). The investigator may also contact the sponsor to discuss whether the study drug treatment must be discontinued.

6.2.3 Special precautions

Subjects should receive KN046 and KN026 infusion in a medical institution equipped with resuscitation equipment and emergency medicine, and stay in the hospital for observation for at least 2 hours after the end of each infusion. Whenever a subject receives KN046 and KN026 infusion, it must be ensured that the subject's infusion-related reactions or severe hypersensitivity reactions can be urgently handled according to local treatment guidelines. In order to ensure timely treatment of possible allergic reactions, the study site must always have appropriate rescue medications, such as dexamethasone 10 mg, epinephrine 1:1000 or alternative drugs, and assisted ventilation equipment.

KN046 and KN026 infusion should be stopped immediately in the event of ≥ Grade 2 hypersensitivity, inflammatory, or allergic reactions.

Refer to Sections 6.2.3.1 and 6.2.3.2 for NCI recommendations for the treatment of infusion-related reactions, severe hypersensitivity reactions, and oncolytic syndrome, respectively.

6.2.3.1 Infusion-related and hypersensitivity reactions

Symptoms of infusion-related reactions may be fever, cold intolerance, chills, sweating, and headache. Infusion-related reactions can be handled as described in Table 6.

Table 6 Handling of infusion-related reactions caused by KN046 and KN026

NCI-CTCAE grade	Dose modification of KN046 or KN026
**Grade 1 — mild**<br><br>Mild transient reaction; infusion interruption not required; intervention not required.	• Decrease the infusion rate of KN046 or KN026 by 50% and closely monitor for any signs of worsening<br>• The total infusion time of KN046 or KN026 should not exceed 240 minutes
**Grade 2 — moderate**<br><br>Treatment interruption or infusion required, and prompt symptomatic treatment (e.g., antihistamines, NSAIDs, anesthetics, IV fluids)	• Discontinue infusion with KN046 or KN026<br>• Resume infusion after resolution of infusion-related reaction or decrease in severity to at least 1 grade, with the infusion rate of 50% of the original rate<br>• Closely monitor for signs of worsening
**Grade 3 or 4 — severe or life-threatening**<br><br>**Grade 3:** Prolonged (e.g., not rapidly responsive to symptomatic medication and/or interruption of infusion); recurrence of symptoms following improvement; hospitalization due to AEs<br><br>**Grade 4: Life-threatening; urgent intervention required**	• Immediately discontinue infusion with KN046 or KN026 and disconnect the subject's infusion line<br>• Subjects must immediately discontinue treatment with KN046 or KN026 and must not receive any KN046 or KN026 treatment again

If a subject experiences an infusion-related reaction ≥ Grade 2 for the first time leading to treatment interruption, slow down the infusion rate and resume dosing. If an infusion-related reaction ≥ Grade 2 occurs again, stop the infusion and permanently discontinue KN046.

NCI-CTCAE = National Cancer Institute-Common Terminology Criteria for Adverse Events

Once the infusion rate of KN046 or KN026 has decreased by 50% due to an infusion-related reaction, this rate must be maintained for all subsequent infusions. In case of infusion reactions, drug preparation and infusion must be detailed.

Severe hypersensitivity reactions could manifest as airway injury, decreased oxygen saturation (less than 90%), confusion, lethargy, hypotension, pale skin/clamminess, and cyanosis.

If severe hypersensitivity reactions occur, subjects should be monitored immediately and injected with epinephrine and dexamethasone, and the ICU should be notified and transferred if required. Specific management principles can be found in the full version of the Emergency treatment of anaphylactic reactions: Guidelines for healthcare providers (UK) at https://www.resus.org.uk/pages/reaction.pdf (Reference: Emergency Treatment of Anaphylactic Reactions: Guidelines for Healthcare Providers, 2008).

If a subject experiences an infusion-related reaction or hypersensitivity reaction, the investigator is advised to collect plasma histamine, IL-6, and C-reactive protein (CRP) within 30 minutes of symptom onset, and collect urine methylhistamine again within 24 hours after symptom onset, as well as unplanned ADA and PK samples. For subjects with chest pain, ECG, myocardial enzymogram (CPK, CK, Tn1, TnT), and BNP should be done within 30 minutes of symptom onset. Serum complements (C3, C4, CH50) are required for subjects with typical or atypical manifestations of "triad", such as arthralgia, rash, and/or gastrointestinal symptoms.

If the investigator judged that the benefit of continuation outweighs the risk to the subject, continuation of KN046 and KN026 will be allowed, and prophylaxis will be given 30-60 minutes prior to dosing in all cycles thereafter. Examples of prophylactic dosing regimens are as follows: cetirizine 10 mg PO, famotidine 20-40 mg PO (or equivalent dose IV), acetaminophen 650 mg PO (or equivalent dose IV). If the subject's hypersensitivity reaction manifests as symptom of tracheospasm, montelukast 10 mg PO should be used as part of the prophylactic dosing regimen. The investigator may determine the need for addition of hormones and/or opiates to the prophylactic regimen based on medical judgment. Discussions with immunologists to determine treatment options are encouraged.

6.2.3.2 Tumor lysis syndrome

KN046 has the potential to initiate ADCC effects and therefore is at risk for tumor lysis syndrome. Once this occurs, processing may be performed according to Figure 6.

Figure 1 Evaluation and initial treatment of tumor lysis syndrome

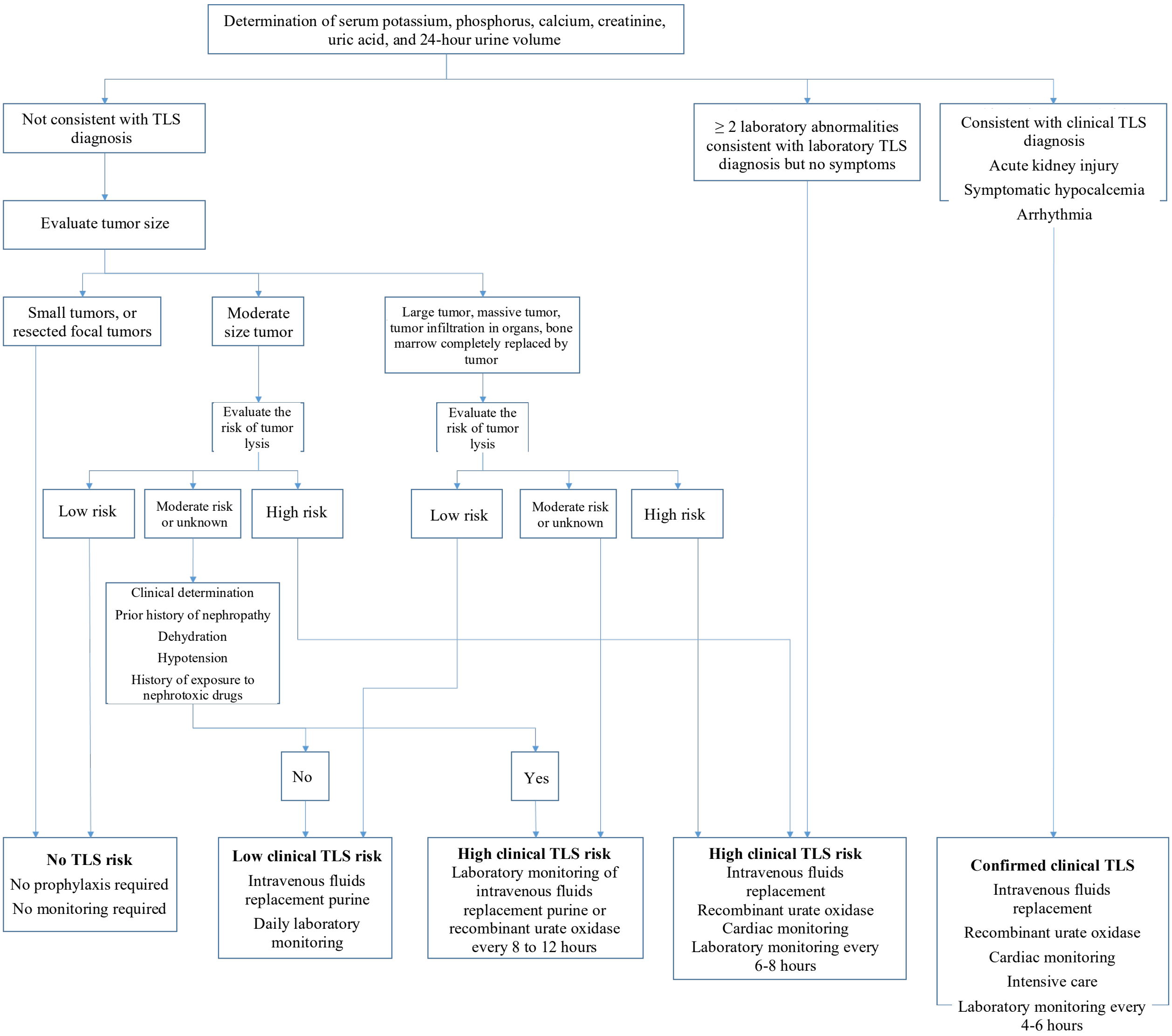

Comments: Howard et al, 2011; TLS: tumor lysis syndrome

7 STUDY PROCEDURES AND EVALUATIONS

7.1 STUDY VISIT PLAN

The evaluation plan for this study is detailed in Table 1.

7.1.1 Screening

Subjects should sign the ICF before screening and before the start of study evaluation. The screening period of this study is within 28 days prior to study drug administration.

Subject information, including demographic information (date of birth, sex, and race) and complete medical history (including tumor history, prior and concomitant medications, prior surgery and radiotherapy, and baseline medical condition, etc.) will be recorded at screening.

Subjects will undergo a full physical examination at screening, including recording of weight, height, vital signs, 12-lead ECG, echocardiography/MUGA, and determination of ECOG performance status.

Safety laboratory tests, including hematology, coagulation, serum chemistry, and urinalysis, must be completed within 7 days prior to study drug administration. Total triiodothyronine (TT3), free T3 (FT3), free thyroxine (FT4), and thyroid stimulating hormone (TSH) will also be evaluated at screening. All subjects should be tested for hepatitis B virus, hepatitis C virus, and human immunodeficiency virus (HIV).

Women of childbearing potential at screening should have a serum β-chorionic gonadotropin test within 7 days prior to study drug administration unless the age-related spontaneous menopause for ≥ 12 consecutive months and follicular estrogen (FSH) increase > 40 mIU/ml, surgical sterilization, or inactive. Pregnancy test is not required for women of non-childbearing potential. If confirmation of menopausal status is required, FSH may be measured at screening.

Oncology (Section 7.2.2), HER2 status, and biomarker evaluation (non-mandatory) will also be performed at screening (Section 7.2.4).

In the event of screening failure due to abnormal laboratory tests, a repeat laboratory test is allowed upon agreement between the investigator and the medical monitor. Failure of a subject to reach the eligibility criteria at screening will result in screening failure and inability to receive study medication. For subjects who fail screening, the following information needs to be recorded on the eCRF:

- Informed consent page;
- Reason for screening failure;
- Demographic information;
- AEs;
- Inclusion/exclusion criteria page.

7.1.2 Treatment period

The treatment period begins from Cycle 1 Day 1 of KN046 and KN026 administration and continues until disease progression (see Sections 6.1.3 and 7.2.2.3), significant clinical deterioration (clinical progression), unacceptable toxicity, or the occurrence of a subject requiring withdrawal from the study or study drug (see Sections 5.3.1 and 5.3.2).

KN046 and KN026 should be discontinued in principle after disease progression is confirmed per RECIST 1.1 (solid tumors) criteria and confirmed by subsequent imaging studies. If a subject is clinically stable and can benefit from continuing KN046 treatment as judged by the investigator, the subject will be allowed to continue treatment with KN046 (see Section 6.1.3). In this case, subjects may be treated with reference to the iRECIST (solid tumors) criteria (Appendix 2). If treatment with KN046 and KN026 is continued after the progression is confirmed per RECIST 1.1 criteria, KN046 and KN026 should be discontinued immediately once the subject is intolerant of the study drug or experiences treatment failure (see Sections 6.1.3, 5.3.1, and 5.3.2).

During the treatment period, subjects will have a visit every 2 weeks except for specific visits specified in the protocol (e.g., PK data collection). The window for the visit period is up to 3 days (± 3 days) before or after the scheduled visit day (except for the PK, ADA sampling visits required in Tables 1 and 2).

The evaluations performed during the treatment period are detailed in and should follow the provisions of Tables 1 and 2.

7.1.3 End of treatment (EOT) visit

Subjects who discontinue treatment with KN046 and KN026 for any reason must have an EOT visit. EOT visit needs to be performed within 7 days of the decision to discontinue treatment, but should be performed before starting a new anti-tumor therapy (if applicable), whichever occurs first. The EOT visit may also be performed on the day of the decision to discontinue treatment with KN046 and KN026, i.e., on the same day as the last pre-treatment evaluation, in which case the tests same as those of EOT need not be repeated.

The eCRF page for EOT visit must include the date of the decision to discontinue KN046 and KN026 treatment, the date of the last KN046 and KN026 treatment, and any of the following reasons:

- AEs;
- Abnormal laboratory value(s);
- Abnormal operation results;
- Deviation from protocol;
- Withdrawal of ICF;
- Lost to follow-up;
- Death (must be noted if it is caused by "study indication" or "other" reasons);
- Disease progression as judged by RECIST 1.1 criteria;

- End of treatment as required by the protocol;
- Other causes, including clinical progression, administrative problems.

If no disease progression as defined by RECIST 1.1 occurs at the EOT visit, subjects will be asked to continue tumor evaluations. If the subject withdraws the informed consent, it must be clearly stated that the subject is willing to continue the follow-up after the end of treatment (see Sections 5.3.2, 7.1.4, 7.1.5).

See Table 1 for the specific assessments in the EOT visit.

7.1.4 30-day and 90-day safety visits

A safety follow-up visit is required for all subjects 30 days (± 3 days) (for KN026 and KN046) and 90 days (± 7 days) (for KN046 only) after the last dose, but should be performed before starting a new anti-tumor therapy (if applicable), whichever occurs first. If the 30-day safety visit is performed on the same day as the EOT visit, the tests same as those of EOT are not required to be repeated in this case.

After the EOT visit, AEs need to be recorded until the 30-day safety follow-up visit. Thereafter, all SAEs and treatment-related non-serious AEs (including immune-related AEs) will be recorded until the 90-day safety follow-up visit. SAEs that persist after the 30-day safety follow-up visit are required to be monitored by the investigator until stabilization or until the outcome is known.

Subsequent anti-tumor therapies, tumor imaging evaluations (see Section 7.2.2, if applicable), etc. will also be collected during the safety follow-up period. Please refer to Table 1 for visits during the safety follow-up period.

7.1.5 Long-term follow-up

SAEs that continue beyond the 90-day safety follow-up visit will need to be monitored in the long-term follow-up until stabilization or until the outcome is known, unless the subject is documented as "lost to follow-up".

Survival status and subsequent anti-tumor therapy of subjects will be collected every 12 weeks (± 14 days) after the EOT visit. Survival follow-up will continue until 1 year after the last subject ends treatment with KN046, or until the last subject dies, whichever occurs first (Section 4.1.4).

Each subject will be followed up until death, loss to follow-up, or the end of the study as a whole (Section 4.1.4). Subjects who do not experience disease progression as defined by RECIST 1.1 after the EOT visit will be asked to continue tumor evaluations (Section 7.2.2.3).

The evaluations to be completed during long-term follow-up are detailed in Table 1.

7.2 STUDY EVALUATION

7.2.1 Demographic and other baseline characteristics

The study evaluations described in this section must be completed at screening.

7.2.1.1 Demographic data

The following data are collected during the screening period:

- Date of birth;
- Sex;
- Race;
- Ethnicity.

7.2.1.2 Tumor diagnosis

Tumor disease information for each subject needs to be confirmed and documented at screening, including:

- At the time of diagnosis, a detailed tumor history, including histopathological type, grade, and stage, determined according to the criteria for cancers, lymph nodes, and metastasis classification from the Union for International Cancer Control (UICC);
- All prior anti-tumor therapies (including surgery, radiotherapy and chemotherapy, immunotherapy);
- Any other diseases receiving chemotherapy, radiotherapy, or immunotherapy;
- Current tumor symptoms and signs, and adverse reactions to current and/or prior anti-tumor therapies;
- Current tumor status.

7.2.1.3 Medical history

The complete medical history of each subject will be collected and documented at screening to confirm whether the subject is eligible for inclusion. The complete medical history should contain at least the following information:

- Prior non-neoplastic diseases and concomitant therapies;
- Prior neoplastic diseases and concomitant therapies;
- Medications (including botanicals) and procedures received within 28 days prior to screening;
- Smoking history;

- Family history of tumor.

7.2.1.4 Other baseline evaluations

Additional baseline evaluations include oncology evaluations, vital signs, full physical examinations, ECOG score (Appendix 5), laboratory tests (Section 7.2.3.5), 12-lead ECG, ECHO/MUGA, biomarker tests, and evaluation of inclusion/exclusion criteria (Table 1).

Refer to Section 7.2.2.2 for baseline oncology evaluation; Section 7.2.3 for baseline safety evaluation; Section 7.2.7 for biomarker tests; Section 7.2.5 for pharmacodynamic evaluation; and Sections 5.1 and 5.2 for evaluation of inclusion/exclusion criteria.

7.2.2 Efficacy evaluation

In this study, the investigator will evaluate tumors according to RECIST 1.1 criteria (Appendices 1 and 2). Results assessed by the investigator will be used to guide clinical decisions (e.g., discontinuation of KN046 and KN026 treatment) and for the analysis of primary and secondary efficacy endpoints. Refer to the imaging manual for detailed oncology evaluations.

7.2.2.1 Computed tomography (CT), MRI, FDG PET/CT, and other evaluations

All subjects will have chest/abdomen/pelvis (specific tumor types need to include other specific areas) CT scan or MRI (chest CT is mandatory if MRI is used). If CT/MRI imaging is not sufficient to evaluate tumor burden, other established evaluation methods may be added. Contrast-enhanced CT scan is recommended, and contrast-enhanced MRI may be considered if the subject is allergic to contrast.

Preferably, CT scan or MRI are performed using a 5 mm tomography thickness with a continuous reconstruction algorithm. The tomography thickness in the CT/MRI scan should not exceed 8 mm using a continuous reconstruction algorithm. All scans performed at baseline and other imaging performed as clinically required (other supportive imaging) need to be repeated at subsequent visits. In conclusion, the imaging method needs to be consistent with that used for lesion detection at baseline, and the same imaging device is preferred for subsequent tumor evaluation visits.

If a brain CT/MRI scan is not performed within 42 days prior to screening, it is required at screening (either method, enhancement is recommended), and a brain CT/MRI scan will be added as clinically indicated at subsequent follow-up if new central nervous system symptoms occur. If no bone scan is performed within 3 months prior to screening, it should be performed at screening, and additional bone scan may be considered at subsequent follow-up if clinically indicated.

For each subject, the investigator will assign 1 or more of the following tumor evaluation methods to determine response to treatment or disease progression: CT or MRI, physical examination, and other evaluation results of primary and/or metastatic tumor lesions. During the study period, imaging methods that are most appropriate for the subject, as well as methods that are most appropriate for evaluating the subject's tumor status should be considered. The evaluation method of tumor status of subjects during the study should be consistent with that used at the time of enrollment.

7.2.2.2 Baseline oncology evaluation

For subjects with solid tumors, target and non-target lesions will be recorded for each subject at screening according to RECIST 1.1 criteria for baseline oncology evaluation.

Subjects must have measurable lesions as judged by the investigator per RECIST 1.1 criteria at baseline (Section 5.1). Subjects with only non-measurable lesions are not eligible for the study.

Measurable lesions is defined as at least 1 measurable non-nodal or nodal lesion by the RECIST 1.1:

- Measurable non-nodal lesions: lesions that can be accurately measured with at least 1 dimension and have the diameter not be less than 2 times the thickness of the tomography scan (e.g., if the thickness on spiral CT scan or MRI is 5 mm, the diameter of the lesion must be ≥ 10 mm);
- Measurable nodal lesions: lymph nodes ≥ 15 mm in short axis;
- Bone lesions selected as target lesions must be osteolytic or mixed osteolytic-osteoblastic lesions that can be identified by CT/MRI and contain soft tissue components.

Non-measurable lesions refer to all other non-measurable lesions, including small lesions (e.g., if the tomography thickness is 5 mm, the long diameter of the CT or MRI scan is < 10 mm, or the short diameter of the pathological lymph node is ≥ 10 mm and < 15 mm). Examples of non-measurable lesions include osteoblastic lesions, leptomeningeal disease, ascites, pleural/pericardial effusion, inflammatory breast disease, cutaneous lymphangitis/pneumonia, abdominal masses not confirmed and followed by imaging techniques, and cystic lesions.

Lesions at sites of prior radiotherapy should be considered non-measurable unless disease progression is demonstrated by unequivocal imaging and the lesion is measurable.

Up to 5 measurable lesions (nodal and non-nodal; up to 2 lesions per organ) will be selected as target lesions and represent, whenever possible, all organs involved. Target lesions should be selected based on size (lesions with the maximum diameter) and suitability for accurate repeated measurements. Each target lesion must be measured at baseline and uniquely numbered sequentially on the eCRF (even if located in the same organ).

Non-target lesions include all other lesions, i.e., lesions that do not meet the criteria for target lesions at baseline. Multiple non-target lesions involved in the same organ may be evaluated as a group and recorded as a single item. Measurement of these lesions is not required. Any non-target lesion identified at baseline must be recorded on the eCRF.

7.2.2.3 Subsequent oncology evaluations

Subjects will undergo subsequent oncology evaluations every 8 weeks and every 12 weeks after 48 weeks until radiographic disease progression, start of new anti-tumor therapy, withdrawal of consent, loss to follow-up, and end of study, whichever occurs first. Subjects who discontinue treatment with KN046 due to unacceptable toxicity or clinical progression should continue to be followed up for imaging and oncological evaluation until radiographic disease progression occurs.

All target or non-target lesions identified at baseline need to be evaluated at subsequent follow-up visits and documented on the appropriate eCRF page, and the evaluation method needs to be consistent with that at baseline. Whenever possible, the same radiologist should perform all oncology evaluations of the subject at baseline and subsequent follow-up visits. New lesions are those that are not identified at baseline but subsequently appear, either measurable or non-measurable, and should be evaluated/measured and recorded on the eCRF page for new lesions.

If a subject experiences a CR or PR as defined by RECIST 1.1 criteria, a CT or MRI scan should be performed 6 weeks later (but not earlier than 4 weeks) as planned to confirm the CR or PR (confirm response).

If a subject experiences PD as defined by RECIST 1.1 criteria for the first time and is clinically stable, the disease progression may be confirmed 6 weeks later (no earlier than 4 weeks and no later than 8 weeks) for clinical treatment decision making (Section 6.1.3).

7.2.3 Safety evaluation

Each subject will be monitored for safety in this study, including hematology, blood chemistry and electrolytes, coagulation, urinalysis, 12-lead ECG, ECHO/MUGA, physical examination, vital signs, height, weight, ECOG score, endocrine test, and immunogenicity test. Detailed tests and frequencies are presented in Table 1.

7.2.3.1 Physical examination

General physical examination will include: general appearance, skin, neck (including thyroid), eyes, ears, nose, throat, lungs, heart, abdomen, back, lymph nodes, extremities, blood vessels, nervous system, and vital signs (Section 7.2.3.2).

Systemic physical examination will be performed at the following visits:

- Screening phase;
- EOT visit.

Symptom-directed physical examinations will include: general condition, vital signs (Section 7.2.3.2), and abnormal findings from physical examination.

Symptom-directed physical examinations will be performed at the following visits:

- Prior to each dose of KN026 and/or KN046;
- 30- and 90-day safety follow-up visits.

Physical examination needs to be documented in the source documents of the study sites. Physical examination abnormalities prior to signing of ICF should be recorded on the appropriate eCRF page for the past/current medical history. New or worsening physical examination abnormalities after signing of informed consent should be recorded as AEs on the appropriate eCRF page.

7.2.3.2 Vital signs

Vital signs will include respiratory rate, pulse rate, blood pressure, and temperature, and will be performed at the following visits:

- Screening phase;
- Prior to each dose of KN026 and/or KN046;
- EOT visit;
- 30- and 90-day safety follow-up visits.

If vital sign measurements coincide with the safety laboratory or PK blood sampling, vital sign measurements should be performed first.

7.2.3.3 Body height and weight

Height will be measured at screening.

Body weight will be measured at the following visits:

- Screening phase;
- Prior to each dose of KN026 and/or KN046;
- EOT visit;
- 30- and 90-day safety follow-up visits.

Body weights measured prior to each dose of KN026 and/or KN046 will be used to calculate the administered doses of KN046 and KN026 (Section 6.1.2).

7.2.3.4 ECOG score

ECOG scoring will be performed at the following visits:

- Screening phase;
- Prior to each dose of KN026 and/or KN046;
- EOT visit;
- 30- and 90-day safety follow-up visits.

ECOG scoring methods are presented in Appendix 5.

7.2.3.5 Laboratory tests

Laboratory tests for safety evaluation will be performed at the local laboratory, and the normal range of the local laboratory will be adopted, including: hematology, serum chemistry

electrolytes, coagulation, urinalysis, endocrine tests, and urine pregnancy/serum pregnancy test (Table 8).

Prior to shipment of study drug, the study site must provide the sponsor with a list of normal ranges for the study site laboratory. Changes in the normal ranges of the laboratory during the course of the study are required to be provided to the sponsor-designated CRO.

Subjects will be fasted for at least 8 hours prior to blood sampling, and samples will be collected prior to the dose of KN046 and KN026. All routine laboratory tests will be analyzed in the local laboratory of the study site, and test results that are clinically significant for the subject's treatment decision (e.g., hematology, blood chemistry and electrolytes, endocrine tests) must be known and evaluated prior to the dose of KN046 and KN026. Reporting of results must be maintained as part of the subject's medical records or source documents, and documented in the eCRF.

Table 7 Laboratory test contents

**Blood chemistry**	**Hematology**	**Coagulation**	**Urinalysis**
Alkaline phosphatase	Red blood cell count	Prothrombin time (PT)	pH
Alanine aminotransferase	Hematocrit	Partial thromboplastin time	Specific gravity
Aspartate aminotransferase	Hemoglobin	International Normalized Ratio	Glucose
Albumin	Mean hemoglobin content (MCH)	Thrombin time (TT)	Protein
Total bilirubin	Mean corpuscular hemoglobin concentration (MCHC)	**Hormone levels**	Ketones
	Mean corpuscular volume (MCV)	Follicle-stimulating hormone (if applicable)	Urine blood cells
Blood urea nitrogen/total urea	Platelet counts	Thyroid stimulating hormone (TSH)	24-hour urine protein 1
Blood calcium	WBC count with differential	Free thyroxine (FT4)	Urine protein/creatinine<br>Random urine protein to creatinine ratio 1
Chloride	Neutrophil count	Total triiodothyronine (TT3)	
Creatinine	Lymphocyte count<br>Lymphocyte count	Free T3 (FT3)	**Viral test**
Glucose	Monocyte count		Five tests for hepatitis B (If HBsAg is positive, HBV DNA should be added)
Lactate dehydrogenase	Basophil count		HCV antibody (If HCV antibody is positive, HCV

			RNA should be added)
Phosphate	Eosinophil count	**Pregnancy test (if applicable)**	HIV 1/2 antibody
Total protein		Serum β-human chorionic gonadotropin (HCG)	
Blood potassium		Urine HCG	
Blood sodium			

1. For urinalysis, if urine protein is ≥ 2+ (dipstick), a 24-hour urine sample will be collected for determination of total protein and a random urine sample will be collected for determination of protein/creatinine ratio

7.2.4 Pharmacokinetic evaluation

The PK evaluation plans for the dose escalation phase and dose expansion phase are presented in Table 1 and Table 2, rerspectively. The PK of the dose escalation phase requires intensive blood sampling, which will be analyzed using compartment-free method (Section 9.6.3). The PK collection in the dose expansion phase is sparse sampling and will be analyzed by a non-linear mixed effects model, which will be described in detail in a separate PK/pharmacodynamic analysis plan.

The PK blood sample collection should be recorded on the appropriate eCRF page, including the exact date and clock time of administration and blood sample collection of KN046 and KN026.

Whole blood samples are drawn by direct venipuncture or by inserting a venous catheter or indwelling trocar into the forearm vein. Samples will be processed, labeled, stored, and shipped as detailed in the laboratory manual. After sampling, all samples should be processed within 60 minutes and stored frozen at ≤ -70°C until transport. PK blood samples are tested by a central laboratory.

If the PK samples and immunogenicity (anti-KN046 and anti-KN026 antibodies) samples are scheduled to be collected at the same time, all of these samples should be collected at the same time with the exact collection time of each sample recorded.

7.2.5 Immunogenicity evaluation

The immunogenicity of KN046 and KN026 is evaluated by detecting anti-drug antibodies (ADAs) and neutralizing antibodies (NADAs). The detection time for ADA is presented in Table 4. Subjects who are positive for the ADAs will be further tested for antibody titers.

Please collect, process, standardize, store, and ship the immunogenicity samples as specified in the laboratory manual. Immunogenicity testing will be performed at the central laboratory.

7.2.6 Biomarker evaluation

7.2.6.1 Evaluation of tumor tissue biomarkers

All subjects will be required to submit slides for central laboratory review of HER2 status at screening. Subjects who have received prior HER2-targeted therapy such as trastuzumab in the dose escalation phase or dose expansion phase should provide tumor tissue samples collected after failure of HER2-targeted therapy for determination of HER2-positive status (Section 4.1).

Subjects who agree to participate in non-mandatory biomarker testing can submit formalin-fixed, paraffin-embedded tissue blocks containing recently obtained tumor tissues (biopsy specimens from a non-irradiated area within 2 years) for TMB/GEP, PD-L1 expression, and TIL cell analysis or at least 8-10 unstained tumor slides (prepared within 1 week) at screening. These analyses will be performed at a central laboratory with the order of priority as TMB/GEP (Section 4.1.2) →PD-L1 expression→TIL. The evaluation plan for biomarkers is presented in Table 1. Biomarker samples should be collected, processed, standardized, stored and shipped in accordance with the laboratory manual and materials provided by the sponsor and Alphamab or its designated central laboratory.

TMB and GEP will be respectively measured by NGS method and Nanostring method, while PD-L1 positive (tumor and stromal cells) will be determined using an immunohistochemical concomitant diagnostic method developed by the central laboratory. Tumor cell analysis includes the percentage of tumor cells at each staining intensity level (0, 1+, 2+, and 3+). PD-L1 expression in tumor microenvironment cells (e.g., immune cells/infiltrating lymphocytes) may also be determined. The number and location of tumor-infiltrating lymphocytes, include but not limited: immunohistochemistry for detection of ICOS+CD4 T cells, natural killer cells, B cells, macrophages, neutrophils, bone marrow-derived suppressor cells, fibroblasts and vascular structures, CD8 and Foxp3 regulatory T cells.

Tissue collection: Specimens should be collected at screening, and tissues (blocks or slides) from archival specimens should be obtained within 1 year prior to screening. Specimens obtained by endoscopic biopsy, aspiration biopsy, excisional biopsy, trephine biopsy, and surgery are acceptable, but those obtained by fine needle aspiration biopsy are not accepted.

Provision of samples: Priority 1: tumor-containing FFPE tissue block; priority 2: if the entire tumor-containing FFPE tissue block cannot be provided, a section of this block should be provided: it should be freshly cut (within 1 week), be 4-6 μm thick, and be mounted with SuperFrost Plus microscope slides.

Tissue processing: Tumor tissues should be fixed in 10% neutral formalin, embedded in paraffin, and routinely processed for histological evaluation. Formalin substitutes are not suitable as a fixative.

Specimens and tissue bank: Biomarker samples may be stored until the end of the study and analyzed together with samples from other studies to analyze the relationship of biomarkers to the efficacy of the study drug or disease, and develop possible diagnostic kits.

8 EVALUATION AND RECORDING OF ADVERSE EVENTS

8.1 DEFINITION OF ADVERSE EVENTS

8.1.1 Adverse event

An adverse event (AE) is an untoward medical occurrence in a subject while receiving treatment with a medicinal product that does not necessarily have a causal relationship with the treatment. An AE may therefore be a discomfort or unexpected sign (including abnormal laboratory finding), symptom, or disease temporally related to the use of a medicinal product, regardless of the causality to the medicinal product.

For a surgical or diagnostic procedure, the condition/disease that led to the procedure, rather than the procedure itself, should be considered an AE. Conditions such as scheduled examinations and surgeries (including endoscopy, appendectomy), hospitalization for convenience or social reasons, and pre-existing symptoms or daily fluctuations rather than worsening of the disease prior to the start of the study are not considered AEs.

8.1.2 Serious adverse event

A serious adverse event (SAE) is an untoward medical occurrence at any dose that results in:

- Death;
- Life-threatening;

  Note: The term "life-threatening" in the definition refers to a subject who is at risk of death at the time of the event; it does not refer to an event that hypothetically might cause death if it is more severe.

- Hospitalisation or an extension of existing hospitalisation required;
  - Hospitalization due to social reasons or convenience reasons (e.g., insurance, inconvenience of accommodation of out-of-town patients), rather than due to the AE itself, is not required to be reported as an SAE;
- Resulting in permanent or severe disability/incapacity;
- Resulting in congenital anomaly/birth defect;
- Other medically significant events.

  Note: Important medical events that do not result in death, are not life-threatening, or do not result in hospitalization may be assessed as SAEs based on the investigator's medical judgment, damages that may brought to the subject, or need of medical or surgical intervention to prevent any of the above SAEs. Examples of such events include allergic bronchospasm requiring intensive treatment in an emergency room or at home, hematologic disease or convulsions that do not result in hospitalization, or development of drug dependence or drug abuse.

8.2 RECORDING AND REPORTING OF ADVERSE EVENTS

This study requires documentation of any untoward medical occurrence during the study after signing the ICF until the end of the 30-day safety follow-up or the initiation of new anti-tumor therapy, whichever occurs first. SAEs and TRAEs require to be collected until the end of the 90-day safety follow-up. SAEs suspected to be related to KN046 needs to be recorded and reported at any time, regardless of the time from discontinuation of KN046. The symptomatic left ventricular systolic dysfunction and asymptomatic left ventricular systolic dysfunction (≥ 10% and < 50% decrease in LVEF from baseline) within 12 months after the last dose of KN026 should be recorded and reported as AEs, and those meeting the criteria for SAEs should be reported to regulatory authorities in accordance with local regulations.

The investigator will grade each AE according to NCI-CTCAE 5.0. If the severity/intensity of a particular AE is not specifically graded by the guideline, the investigator will grade in accordance with the following general definitions of Grade 1 through Grade 5, with his/her best medical judgment:

- Grade 1: Mild; asymptomatic or mild symptoms; clinical or diagnostic observations only; intervention not indicated.
- Grade 2: Moderate; minimal, local or noninvasive intervention indicated; limiting age-appropriate instrumental activities of daily living (ADL) (e.g., preparing meals, shopping for groceries, using the telephone, managing money, etc.);
- Grade 3: Severe; serious or clinically significant but not immediately life threatening; hospitalization or prolongation of hospitalization indicated; disabling; limiting self-care ADL (e.g., bathing, dressing and undressing, feeding self, using the toilet, taking medications), without bedridden.
- Grade 4: life-threatening;urgent intervention indicated; disability;
- Grade 5: death related to AEs.

Each AE report should include a description of the event, duration (date/time of onset and resolution: if it is necessary to evaluate the time of onset of the AE relative to dosing, "Time of administration" should be also recorded), severity, relationship with the study drug, other potential causes leading to the AE, any treatment administered or other action taken (including delay or discontinuation of the study drug), and outcome. In addition, SAEs will be identified, with the corresponding serious criteria recorded.

In the case of death, the primary cause of death (the event leading to death) should be recorded and reported as an SAE. "Fatal" will be recorded as the outcome of the event; death itself could not be recorded as an SAE separately. Only if no cause of death can be reported (e.g., sudden death, unexplained death), the death per se can be reported as an SAE.

Worsening of symptoms and signs related to the study indication is required to be recorded as AEs in the CRF. Disease progression and its associated symptoms/signs (as per RECIST 1.1 criteria) confirmed by imaging evaluation or other means are used as efficacy endpoints and are not recorded as AEs, which are also not required to be reported as SAEs even if they lead to serious outcomes.

The diagnosis should be recorded on the eCRF rather than the individual symptoms and signs (e.g., liver failure should be recorded, rather than jaundice, elevated transaminase with flapping

tremor). However, if the symptoms and signs cannot be classified as an individual diagnosis at the time of the report, each individual event should be recorded on the eCRF as an AE or an SAE. If a subsequent diagnosis is established, it should be reported as follow-up information.

The investigator should clinically evaluate the causality of each AE or SAE to KN046:

- **Not related:** no reasonable relationship to study drug is suspected. The occurrence of the event is more likely to be explained by other factors than the study drug;
- **Related:** a reasonable relationship to study drug is suspected . The AE could be medically (pharmaceutical/clinical) attributed to study drug.

AE records should ensure the completeness, accuracy and consistency of the data, as detailed in the "eCRF Completion Guidelines".

8.2.1 Recording and reporting of serious adverse events

In the event of any new SAE (of any grade) occurring during the reporting period, the investigator must immediately (within 24 hours of awareness of the event) notify Alphamab or its designee by email, and the telephone notification cannot replace the paper report. In the event of any new conditions for previously reported SAEs occurring, the reporting procedures and time limits are the same as for the previously reported SAEs . Alphamab's email address for safety-related reporting is: PV-alphamabonc@mobilemd.cn.

For names, addresses, telephone, and fax numbers for SAE reporting, see details in the SAE Report Form. All written reports should be sent using the SAE Report Form, which must be completed by the investigator in accordance with the specific completion instructions. In addition, the AE section of the eCRF must be completed. Relevant pages of the eCRF (e.g., medical history, concomitant medications) may be simultaneously provided. In all cases, the information provided in the SAE Report Form must be consistent with the event data recorded in the corresponding sections of the eCRF. If the Alphamab or its designee requests SAE follow-up information (e.g., other information, outcome and final evaluation, specific records (if required)) or questions about the SAE report, the investigator should respond immediately, with the same time limit as for the initial SAE report. This allows Alphamab or its designee to evaluate the event promptly, enabling the company to meet stringent time requirements of the regulatory authorities on the obligation of expedite reporting of safety events.

In addition to reporting all SAEs to the sponsor and Alphamab, the investigator also must report to the Ethics Committee in accordance with regulatory requirements.

The sponsor has an obligation to report safety to regulatory authorities. The sponsor and Alphamab or its designee will submit safety reports to the regulatory authorities, investigators, or ethics committees (if applicable), in accordance with regulatory requirements in China. The sponsor and Alphamab will prepare suspected unexpected serious adverse reaction (SUSAR) reports or SAE reports of the study drug and send them to the relevant investigators as required by regulations.

8.2.1.1 Follow-up of serious adverse events

In the event that a subject experiences an SAE, the investigator is required to follow up the SAE (clinical symptoms/signs, laboratory test abnormalities, or other) until it returns to normal or baseline level, or the clinical outcomes are stable. Therefore, the SAE follow-up may continue beyond the end of the study, and the sponsor may also request the SAE follow-up results after the end of the study.

8.2.2 Adverse reactions of special interest

"Adverse events of special interest" occurring during the study requires to be reported immediately to the sponsor and Alphamab, Safety department, or designee, and should be reported to the Sponsor and Alphamab by the investigator within 24 hours of awareness of the event and recorded on the Adverse Event Form of the eCRF, whether it is an SAE or not.

Adverse reactions of special interest include:

- ≥ Grade 3 infusion-related adverse reactions or hypersensitivity reactions;
- KN046
    - ≥ Grade 3 immune-related adverse reactions resulting in dose interruption of KN046 that cannot recover to ≤ Grade 1 or baseline within 12 weeks;
    - ≥ Grade 3 sensory and motor neuropathy (including Guillain-Barre syndrome, myasthenia syndrome) resulting in discontinuation of KN046;
    - Any suspicious KN046 treatment-emergent serious adverse drug reactions (TESADRs) during nonhematologic treatment; rare TESADRs, including but are not limited to, serum sickness, myocarditis, hemolytic anemia, Lambert-Eaton syndrome, myasthenia gravis, rhabdomyolysis syndrome, some epileptic seizure, vasculitis, and pemphigus; any treatment-related serious adverse drug reactions (TRSADRs) leading to discontinuation of KN046;
    - ≥ Grade 4 immune-related hematologic toxicity; ≥ Grade 4 skin toxicity;
    - ≥ Grade 3 immune-related pneumonia;
    - Recurrence of Grade 2 immune-related pneumonia lasting more than 4 weeks after active treatment;
    - Grade 2 immune-related central nervous system toxicity lasting more than 4 weeks after active treatment;
    - ≥ Grade 3 immune-related colitis;
    - ≥ Grade 3 immune-related uveitis or optic neuritis;

    - ≥ Grade 3 immune-related hepatitis with ALT/AST ≥ 10 x ULN; or ALT/AST ≥ 5 x ULN and lasting more than 2 weeks; or ALT/AST ≥ 5 x ULN with total bilirubin ≥ 3 x ULN; or ALT/AST ≥ 5 x ULN with gastrointestinal symptoms (e.g., nausea, vomiting, tenderness of right upper abdomen);
    - ≥ Grade 3 immune-related nephritis, and renal insufficiency;
    - ≥ Grade 1 immune-related myocarditis.
- All cardiac AEs (whether related to KN026 or not) within 1 year after the last dose of KN026 are required to be reported, including the following categories. In addition, all symptomatic left ventricular systolic dysfunction occurring within 3 years after the last dose of KN026 are required to be reported.
    - Symptomatic left ventricular systolic dysfunction;
    - Asymptomatic left ventricular systolic dysfunction with the following:
        - ≥ 10% absolute decrease in LVEF from baseline and < 50% absolute LVEF;
        - ≥ 15% absolute decrease in LVEF from baseline.

8.2.3 Recording and reporting of laboratory test abnormalities and other abnormalities

Laboratory test abnormalities and other abnormal study findings (e.g., ECGs) that are judged to be clinically significant, such as related clinical symptoms and signs, leading to treatment interruption, need for medical intervention, or changes in concomitant therapy, should be recorded as AEs; the laboratory test abnormalities that are judged to be not clinically significant are not required to be recorded as AEs. If a clinically significant laboratory test abnormality or vital sign abnormality is a sign of a disease or syndrome (e.g., alkaline phosphatase and bilirubin increased exceeding 5 times the upper limit of normal (ULN) caused by cholecystitis), only the diagnosis (i.e., cholecystitis) is recorded on the AE Form of the eCRF. Medical issues identified must be reported as AEs, with values above or below the normal range indicated (e.g., "blood potassium increased" should be recorded rather than "blood potassium abnormal"); if laboratory test abnormalities or vital sign abnormalities have standard clinical terms corresponding thereto, clinical terms should be recorded in the eCRF, e.g., blood potassium increased to 7.0 mEg/L should be recorded as "hyperkalemia".

8.2.3.1 Hepatic function abnormal

The investigator must report an AE if: the most appropriate diagnosis or laboratory abnormality (when the diagnosis cannot be established) should be recorded on the AE Form of the eCRF and reported to the sponsor and Alphamab within 24 hours of awareness of the event, whether it is an SAE or not:

- ALT/AST≥10 x ULN;

- ALT/AST ≥ 5 x ULN and lasting more than 2 weeks;
- Or ALT/AST ≥ 5 x ULN with total bilirubin ≥ 3 x ULN; or
- ALT/AST ≥ 5 x ULN with gastrointestinal symptoms (e.g., nausea, vomiting, tenderness of right upper abdomen) and treatment ALT/AST > 10 x ULN.
- Subjects with treatment-emergent ALT or AST elevations greater than 3 times the baseline value, or with increased transaminases at baseline are found to have ALT or AST elevations more than 2 times the baseline value combined with total bilirubin elevations exceeding 2 times the ULN (≥ 35% of which are direct bilirubin);
- The treatment-emergent ALT or AST increases to more than 3 times the baseline value with clinical jaundice.

8.2.4 Pregnancy and intrauterine exposure

To ensure the safety of the subject, pregnancy events occurring in the subject or the subjects' partner during treatment with the study drug or within 6 months after discontinuation of the study drug should be reported to the sponsor and Alphamab within 24 hours of awareness. Pregnant patients should be followed up to determine the pregnancy outcomes, including spontaneous abortion or voluntary termination of the pregnancy, details related to the birth of the infant, and the presence of birth defects, congenital abnormalities, or maternal genetic and/or neonatal complications.

Pregnancy events should be recorded on the clinical study pregnancy form, and reported by the investigator to the sponsor and Alphamab or its designee. Follow-up information of the pregnancy should be recorded on the same form, which should include an evaluation on the relationship between the study drug and the pregnancy outcomes. All SAEs occurring during pregnancy must be recorded and reported on the SAE report form. Pregnancy outcomes must be collected for the female partners of the male subjects administrated with study drug in this study. Informed consent regarding these pregnancy outcomes should be obtained from the mother when reporting.

Adverse pregnancy outcomes such as spontaneous abortion, fetal death, fetal malformation, stillbirth, birth defect, or congenital abnormality should be reported as SAEs.

8.2.5 Overdose, medication error, and drug abuse

An overdose is defined as more than 20% of the dose administered as specified in the protocol.

If a subject experiences an overdose, medication error, abuse, or misuse in the study, regardless of whether there is a relevant adverse event, the investigator should report the situations to the sponsor and Alphamab within 24 hours of awareness. If the above situations lead to SAEs, the investigator should report the SAEs.

9 DATA ANALYSIS AND STATISTICS

9.1 Statistical methods

All data for all subjects in the study will be presented by safety analysis set. Unless otherwise noted, all data will be evaluated based on actual observations and missing data will be not estimated.

Study results will be summarized using descriptive statistics, i.e., statistics for continuous variables may include mean, median, range, and standard deviation/variability. Qualitative variables will be summarized by count and percentage. The uncertainty of estimates will be assessed by confidence intervals (CIs). The specific analytical methods will be described in the Statistical Analysis Plan (SAP).

9.2 Sample size calculation

The sample size calculation for the dose escalation phase is not based on statistical assumptions, but the number of subjects in each dose group is specified according to the 3+3 method.

In Part 1 of the dose expansion phase, 20 to 25 subjects will be enrolled. The sample size calculation is based on the estimate of the 95% CI for ORR using the Clopper Pearson method:

**Sample size**	**ORR (%)**	**ORR 95% CI**
25	40%	(21.1%, 61.3%)
	45%	(24.4%, 65.1%)
	50%	(31.3%, 72.2%)
	55%	(34.9%, 75.6%)
	60%	(38.7%, 78.9%)
	65%	(42.5%, 82.0%)
	70%	(50.6%, 87.9%)
	75%	(54.9%, 90.6%)
**Sample size**	**ORR (%)**	**ORR 95% CI**
20	40%	(19.1%, 63.9%)
	45%	(23.1%, 68.5%)
	50%	(27.2%, 72.8%)
	55%	(31.5%, 76.9%)
	60%	(36.1%, 80.9%)
	65%	(40.8%, 84.6%)
	70%	(45.7%, 88.1%)
	75%	(50.9%, 91.3%)

9.3 Analysis of data sets

The following analysis data sets will be used for statistical analysis and data reporting.

9.3.1 Safety set

The safety set (SS) includes all subjects who have received at least 1 full or partial dose of KN026 and/or KN046. Subjects will be categorized by protocol-planned treatment group and HER2 status (HER2 positive vs low HER2 expression). All raw data will be counted using SS, which will be the default analysis set used for all analyses unless otherwise noted.

Subjects who failed screening will be listed only, without summary analysis.

9.3.2 Efficacy analysis set

The efficacy analysis set (EAS) includes all subjects who have received at least 1 full or partial dose of KN026 and/or KN046 and have at least 1 postbaseline tumor imaging evaluation. Subjects will be categorized by protocol-planned treatment group and HER2 status (HER2 positive vs low HER2 expression). The ORR and DOR data are statistically analyzed using the EAS.

9.3.3 DLT analysis set

The DLT analysis set includes all subjects in the dose escalation phase of the study who have received ≥ 80% of KN026 and KN046 scheduled dosing, or experienced a DLT during the DLT observation period.

9.3.4 PK analysis set

The PK analysis set is defined as subjects who have received at least 1 full or partial dose of KN026 and/or KN046 and have at least 1 post-dose PK sample for analysis.

9.3.5 Immunogenicity analysis set

The immunogenicity analysis set is defined as subjects who have received at least 1 full dose or partial dose of KN026 and/or KN046 and have at least 1 post-dose immunogenicity sample for analysis.

9.4 Demographic and other baseline characteristics

Demographic and other baseline data, including age, gender, height, weight, and ECOG score, will be presented for each subject in the study report, and be summarized by dose level using descriptive statistics (continuous data) or frequency tables (categorical data).

9.5 Primary endpoint analysis

DLT: The frequency of DLTs will be reported by dose level according to the DLT analysis set;

ORR and DOR: 95% confidence interval calculated by Clopper Pearson reported by dose group and/or tumor type in the EAS.

9.6 Analysis of secondary endpoints

9.6.1 Safety endpoints

Actual cumulative dose and duration of exposure (days), dose intensity (calculated ratio of actual dose to actual treatment duration), relative dose intensity (calculated ratio of dose intensity to planned dose/planned treatment duration), number of dose delays, and numbers treatment discontinuations will be listed and summarized for KN046 and KN026.

Safety analysis will be performed based on the SS. Descriptive statistical analyses of safety endpoints will be performed by dose level. Safety analyses will be based on the incidence of AEs, treatment-emergent AEs (TEAEs), adverse reactions of special interests, immune-related AEs (irAEs), treatment-related AEs (TRAEs), and changes in vital signs, ECG, weights, ECOG score, and laboratory values (hematology and serochemistry). The treatment period is defined as a period from the first dose of KN046 to 90 days after the last dose of KN046, or until 1 day before the initiation of a new anti-tumor therapy, whichever occurs first.

9.6.1.1 Adverse events

All AEs will be coded using the Medical Dictionary for Regulatory Activities (MedDRA). The severity of AEs will be graded according to the NCI-CTCAE Version 5.0 Toxicity Rating Scale. AEs will be summarized by preferred term (PT) and system organ class (SOC) with severity and relationship to study medication described.

Analysis of AEs will include, but is not limited to:

- All adverse events (AEs);
- Serious adverse events (SAEs);
- Treatment-emergent adverse events (TEAEs);
- Treatment-related adverse events (TRAEs);
- Immune-related adverse events (irAEs);
- AEs leading to treatment discontinuation;
- AEs leading to death;
- Cardiac AEs.

9.6.1.2 Tolerability

The tolerability of subjects to the study drug will be evaluated by the number of subjects with dose interruption, delay, and discontinuations in each dose group. Reasons for dose interruption, delay, and discontinuation will be listed by dose group, and descriptive statistical analysis will be performed on the frequency of dose interruption, delay and discontinuation by treatment group.

9.6.1.3 Laboratory abnormalities

Laboratory results will be graded according to the NCI-CTCAE Version 5.0. Parameters that cannot be graded will be classified as decreased/normal/increased according to the normal range of laboratory tests.

Test values for each laboratory test (e.g., hematology, serum chemistry) will be listed by laboratory parameter, subject, and dose level. Then, the frequency of obvious laboratory abnormalities (laboratory test abnormalities judged as Grade 3 or 4 according to the NCI-CTCAE Version 5.0) will be described by parameters, number of treatment cycles and dose level; and the frequency of all laboratory abnormalities will be described by parameter, worst grade (according to the NCI-CTCAE Version 5.0), and dose level.

The obvious laboratory abnormalities (laboratory test abnormalities judged as Grade 3 or 4 according to the NCI-CTCAE Version 5.0) will be listed. For the parameters that can be categorized using the CTCAE Version 5.0, laboratory data can be summarized by grade shifts listed in the tables. The parameters that could not be categorized according to the CTCAE Version 5.0 are divided into 3 groups (decreased/normal/increased) according to the normal range, and the data will be listed to statistically analyze the frequency of occurrence.

9.6.1.4 Other safety data (physical examination, ECG, vital signs)

All ECG parameters, including the QT interval corrected according to the Fridericia method (QTc interval) for each subject, will be listed, and descriptive statistical analysis will be performed by dose level and evaluation time, and descriptive statistical analysis will be performed on the change from baseline for each parameter. Dose groups as well as correlations of changes in pharmacokinetic parameters (e.g., C_{max}, AUC_{0-t}) and QTc will be presented graphically.

Blood pressure, pulse, respiratory rate, body temperature, and body weight will be listed for all subjects, and changes from baseline for each value will be presented and statistically described.

All abnormal results of physical examination will be listed.

9.6.2 Efficacy endpoints

Efficacy analyses will be based on the EAS. For individual proportions (CBR), 95% confidence intervals calculated by Clopper Pearson will be reported by dose group; for time-related events (PFS, OS), parameters (including median and 95% confidence interval) will be calculated by the Kaplan-Meier method reported by dose group.

The specific analytical contents and analytical methods will be detailed in the SAP.

9.6.3 Pharmacokinetic endpoints

The concentration-time curves will be plotted for subjects by measured plasma concentrations of KN046 and KN026 and the actual blood collection time points, and PK parameters will be calculated using a non-compartmental model; the mean (arithmetic mean and geometric mean) concentration-time curves will be plotted by measured plasma concentrations of KN046 and KN026 and the scheduled blood collection time points. Concentrations below the LLOQ will be recorded as zero and included in the calculation of PK parameters. Missing sample information will be recorded, but missing samples will not be included in the calculation of PK parameters.

Pharmacokinetic parameters including AUC_{0-t}, AUC_{inf}, AUC_{last}, T_{max}, C_{max}, C_{trough}, CL, V, and $T_{1/2}$ will be calculated using WinNonlin Professional Version 6.4 or higher (Certara, Princeton, NJ, USA) or SAS® Version 9.3 or higher (SAS Institute, Inc., Cary, North Carolina). Descriptive statistical analysis will be performed on pharmacokinetic parameters, including minimum, maximum, median, arithmetic mean, geometric mean, and coefficient of variation (CV)%; T_{max} will be listed by median, 25 and 75 percentiles, minimum, and maximum. An analysis of variance will be performed on each C_{trough} to determine whether steady state is reached. Dose proportionality of KN046 will be assessed using scatter plot regression analysis for AUC (AUC_{0-t}, AUC_{inf}, AUC_{last}) and C_{max}, Hummel Power model or other statistical methods.

The specific analytical contents and analytical methods will be detailed in the SAP.

9.6.4 Correlation between pharmacokinetic and clinical efficacy parameters

Logistic regression analysis will be performed on clinical efficacy parameters (ORR, CBR, PFS rate, OS rate) by AUC (AUC_{0-t}, AUC_{inf}, AUC_{last}), C_{trough}, and C_{max}.

Data of AUC, C_{trough}, and C_{max} can be derived from the results of population pharmacokinetic analysis. It will be described in detail and reported in a separate pharmacokinetic/pharmacodynamic analysis plan.

9.6.5 Immunogenicity endpoints

The frequency of anti-KN046 and -KN026 antibodies and neutralizing antibodies will be listed by dose group, and descriptive statistical analysis will be performed; for ADA-positive subjects, ADA titers will also be presented.

9.7 Exploratory endpoint analysis

The PD-L1 expression level and the correlation between TMB and clinical efficacy variables will be analyzed based on the ROC curve of PD-L1 expression level and TMB value against clinical efficacy variables (ORR, CBR, PFS rate, OS rate), and the comparison of TIL (low vs. medium vs. high) will be based on analysis of variance.

10 ETHICS AND REGULATORY

10.1 Regulations and ethics

This clinical study will be conducted and reported in compliance with the ICH GCP, China GCP, and current laws and regulations of China and foreign countries (including European Directive 2001/20/EC, United States Code of Federal Code Volume 21), and the ethical principles derived from the Declaration of Helsinki.

10.2 Responsibilities of investigators and iec/irc

Recommendations for the protocol and ICF must be reviewed and approved by an Independent Ethics Committee/Institutional Review Board (IEC/IRB) before the study initiation. An approval letter signed and dated by the IEC/IRB must be submitted to the sponsor and Alphamab prior to study initiation. Any amendment to the protocol must be formally approved or filed by the IEC/IRB.

The investigator is responsible for the conduct of the study at the site and ensure that the study is conducted in accordance with the protocol, ethical principles outlined in the Declaration of Helsinki, ICH GCP, China GCP, and any other applicable regulations. The investigator must ensure that the subject provides informed consent before being included in the study.

This study may be submitted to the United States Food and Drug Administration (US FDA) for marketing approval. As specified in Section 54.2 (e) of the United States Code of Federal Regulations (US CFR), for the study making important contribution to the validation of the efficacy and safety of the investigational product (clinical studies covered by the US FDA), the investigators and subinvestigators are obligated to disclose any financial interests that they, their spouses, or their minor children may have in the sponsor or sponsor's investigational product. Disclosure of information during the study and 12 months after the end of the study is required.

The investigator is required to report SAEs to the IEC/IRC, periodically provide updated safety reports, and notify the IEC/IRC at the time of study closure.

10.3 Subject information and consent

Written informed consent is a prerequisite for subject participation in the study. Written informed consent for the subject's participation in the study must be provided prior to the conduct of all study-related procedures.

The investigator must provide adequate information to the subject prior to obtaining the informed consent. The language used in the ICFs is well understood and easy to understand even for lay persons. The subject should be given sufficient time to read this information and the opportunity to ask questions and to request additional information and clarification. Subjects and investigators must sign their names and dates on the ICFs. For subjects who are unable to sign written informed consent, they may be signed by a legal representative. If the subject's legal representative has been fully informed and consented, the subject should be informed of the study content as far as possible, depending on the subject's understanding. Signed and dated ICFs will be retained at the study site and archived by the investigator so that it can be obtained at the time of monitoring, audit, and inspection. A copy of the signed and dated ICF should be provided to the subject and the actual signing date should be recorded in the eCRF.

When new important information becomes available that may be relevant to the subject's informed consent, the sponsor or designee will revise the subject's ICF, which needs to be resubmitted to the IEC/IRB for review and approval. New informed consent and other written information need to be re-signed by the subject, and the investigator needs to explain changes that differ from the previous version, provide adequate reading time for the subject, and provide the subject with the opportunity to ask questions and request additional information and clarify the changes.

Considering the potential teratogenic effects of PD (L) -1 immune checkpoint blockers, female subjects of childbearing potential or fertile male subjects should be informed of the possible uncertain risk to the fetus of the study drug and the need for strict adherence to contraception during the study.

10.4 Subject privacy

A unique number will be assigned immediately to each subject after obtaining informed consent. This number will be used as subject identification in the study and database. All subject data collected in the trial will be retained according to the subject number. The investigator can link trial data to an individual subject via identification information kept at the site. It should be ensured that the original medical data of each subject can be obtained during monitoring, audit and regulatory inspection by regulatory authorities, and the subject confidentiality should be strictly protected.

Data protection and privacy regulations should be observed in capturing, forwarding, processing, and storing subject data. Biomarker samples will be retained for a maximum of 5 years after the end of the study. During this period, samples may be reanalyzed for newly identified markers or using improved techniques. The samples can be destroyed 5 years later, and IEC/IRB approval and subject informed consent will be requested to keep the samples for an additional period.

10.5 Clinical study insurance and subject compensation

This study will provide insurance for all subjects participating in this study. The insurance conditions shall meet local standards (if applicable).

10.6 Regulatory authority monitoring, quality assurance and inspection

The study will be monitored in accordance with ICH GCP and all other applicable regulations. The monitor will visit the study site on a regular basis.

Representatives of the sponsor's Quality Assurance Unit or designated organizations and regulatory authorities must be allowed to inspect the study-related documents and other materials at the site, including investigator site documents and completed eCRFs, study drug, and subject's original medical records/archives.

The protocol, each step of the data collection procedure, and data processing (including the final clinical study report) will be subject to independent quality assurance monitoring. Audits may be performed during the study or at any time thereafter to ensure the correctness and integrity of the study data.

11 STUDY MANAGEMENT

11.1 Closure of study and study center

At study completion, the monitor must complete the following tasks with the investigator or site study personnel, as appropriate:

- Return of all study data to the sponsor and Alphamab
- Respond to all data queries
- Drug accountability, return, or destruction.
- Review the integrity of study records
- Send PK samples to central laboratory

In addition, the sponsor and Alphamab reserve the right to suspend or permanently discontinue the study at the study site for reasons including, but not limited to, safety, ethical, or serious compliance issues. If the sponsor and Alphamab deem it necessary to suspend or permanently discontinue the study at the study site, the investigator will be consulted at that time for discussion of issues including the reasons for the suspension or discontinuation. The sponsor and Alphamab will notify the investigator prior to the suspension or discontinuation takes effect.

If the study is suspended or terminated for safety reasons, the sponsor and Alphamab will promptly notify the investigator and/or the facility, as well as regulatory authorities, of the suspension or termination of the study and the reasons. If required by local regulations, the investigator must promptly notify the Ethics Committee and provide reasons for the suspension or termination of the study.

If the study is permanently discontinued, all study data must be returned to the sponsor and Alphamab. In addition, unused study drug should be treated in accordance with procedures required by the sponsor and Alphamab.

Financial compensation will be made to the investigator and/or facility in accordance with the agreement established between the investigator and the sponsor and Alphamab.

11.2 Storage of study data

The investigator must document the study process adequately and accurately so that the study data can be verified. The documents generally fall into two categories: (1) the investigator's study documents, and (2) the subjects' source data.

The investigator file includes the study protocol and its amendments, case report forms and data query forms, approval certificates and correspondence with ethics committees and regulatory authorities, samples of informed consent forms, drug records, resumes and authorization forms of investigators, and other necessary documents and correspondence.

The subject's source documents (usually defined in advance to record key efficacy/safety parameters) include subject hospital/clinic records, physician's and nurse's notes, appointment book, original laboratory reports, ECG, pathology and special evaluation reports, signed ICF, consultation records, screening and enrollment log, etc.

Investigators should keep these documents intact for 5 years after the drug is approved for marketing. Five years after the drug is approved for marketing, these documents may be destroyed in accordance with relevant regulations, and the sponsor must be informed in advance, and written consent of the sponsor and Alphamab must be obtained prior to destruction.

The sponsor and Alphamab should be notified in advance when the investigator needs to transfer these study documents to any third party or to another location.

If the investigator cannot guarantee this archiving requirement at the site for any or all of the documents, special arrangements must be made between the investigator and the sponsor to store these in sealed containers outside of the site so that they can be returned sealed to the investigator in case of a regulatory audit. If these documents are still in use, copies can be kept elsewhere.

11.3 Provision of study results and information to investigators

When the final study results are obtained, the sponsor and Alphamab will summarize the study results and the summary must be provided for the investigator. In addition, details of treatment administered to the enrolled subjects will be provided for the investigator for data review and confirmation of study results.

Generally, the sponsor and Alphamab will not inform the investigator or the subjects regularly of the study results, since only preliminary data is available early in the study, and these results have yet to be assessed for scientific validity and significance.

11.4 Information disclosure and patents

All information provided by the sponsor and Alphamab and all data or information generated by the study sites (except subject medical records) are the sole property of the sponsor and Alphamab.

All rights, title, and interests in any invention, technology, intellectual property, or industrial property conceived or practiced by the study site personnel during or at the end of the study are all property of the sponsor and Alphamab.

If the ownership clauses contained in the study agreement signed between the study site and the sponsor and Alphamab are inconsistent with this statement, the ownership clauses in the study agreement will prevail.

All information provided by the sponsor and Alphamab and all data or information generated by the study sites (except subject medical records) will be managed by the investigator or other study personnel, and may not be used for any purpose other than guiding the study. These restrictions do not apply to the following:

- Disclosure of information not due to the fault of the investigator or study personnel;
- Information that is necessary to be disclosed to the Ethics Committee for assessment of this study;
- Information that is necessary to be disclosed in order to provide subjects with appropriate medical treatment;
- Study results that may be published, as described in Section 13.5.

If the clauses contained in the study agreement signed between the study site and the sponsor and Alphamab are inconsistent with this statement, the clauses in the study agreement will prevail.

11.5 Publication of study results

The first publication or disclosure of study results must be complete, after which all secondary publications will refer to the first published literature or results. Before submitting any publication, and presenting (for guidance purposes) or disclosing study results generated from the study site (collectively referred to as a "publication"), the investigator must send the planned publication to the sponsor and Alphamab in advance and allow 30 days (full text publication) or 5 working days (abstract) of review. The planned publication should contain only the study results but not the sponsor and Alphamab information or the subject's personal information (including names and initials).

At the sponsor and Alphamab request, the submission or disclosure may be deferred when the sponsor is applying for a patent or seeking similar protection for an invention, technology, intellectual property or industrial property.

If the publication clauses contained in the study agreement signed between the study site and the sponsor & Alphamab are inconsistent with the above statements, the publication clauses in the study agreement will prevail.

12 REFERENCES

[1]. Caroline J Witton, J. R. R., James J Going, Timothy G Cooke and John MS Bartlett. Expression of the HER1-4 family of receptor tyrosine kinases in breast cancer. J Pathol 2003; 200: 290-297.

[2]. Gschwind, A., Fischer, O. M. & Ullrich, A. The discovery of receptor tyrosine kinases: targets for cancer therapy. Nature Reviews Cancer 2004; 4: 361-370.

[3]. Vandergeer, P., Hunter, T. & Lindberg, R. A. Receptor protein-tyrosine kinases and their signal-transduction pathways. Annual Review of Cell Biology 1994; 10: 251-337.

[4]. Jorissen, R. N. et al. Epidermal growth factor receptor: mechanisms of activation and signalling. Experimental Cell Research 2003; 284: 31-53.

[5]. Greenfield, C. et al. Epidermal growth-factor binding induces a conformational change in the external domain of its receptor. Embo Journal 1989; 8: 4115-4123.

[6]. Margolis, B. L. et al. All autophosphorylation sites of epidermal growth factor (EGF) receptor and HER2/neu are located in their carboxylterminal tails. Identification of a novel site in EGF receptor. Journal of Biological Chemistry 1989; 264: 10667-10671.

[7]. Garrett, T. P. J. et al. Crystal Structure of a Truncated Epidermal Growth Factor Receptor Extracellular Domain Bound to Transforming Growth Factor Alpha. Cell Press 2002; 110: 763-773.

[8]. Yamamoto, T. et al. Similarity of protein encoded by the human c-erbb-2 gene to epidermal growth-factor receptor. Nature 1986; 319: 230-234.

[9]. Cersosimo, R. J. Monoclonal antibodies in the treatment of cancer, part 1. American Journal of Health-System Pharmacy 2003; 60: 1531-1548.

[10]. Steffensen, K. D. et al. The prognostic importance of cyclooxygenase 2 and HER2 expression in epithelial ovarian cancer. International Journal of Gynecological Cancer 2007; 17: 798-807.

[11]. Weissinger, F., Reymond, M., Dumke, K. & Krueger, M. Successful Treatment of a Patient with HER2-Positive Metastatic Gastric Cancer with Third-Line Combination Therapy with Irinotecan, 5-Fluorouracil, Leucovorin and Trastuzumab (FOLFIRI-T). Onkologie 2011; 34: 548-551.

[12]. Diaz-Montes, T. P. et al. Clinical significance of Her-2/neu overexpression in uterine serous carcinoma. Gynecologic Oncology 2006; 100: 139-144.

[13]. Papewalis, J., Nikitin, A. Y. & Rajewsky, M. F. G-polymorphism to apolymorphism at amino-acid codon 655 of the human erbb-2/her2 gene. Nucleic Acids Research 1991; 19: 5452-5452.

[14]. Diana Graus-Porta, R. R. B., John M.Daly and Nancy E.Hynes. ErbB-2, the preferred heterodimerization partner of all ErbB receptors, is a mediator of lateral signaling. The EMBO Journal 1997; 16: 1647-1655.

[15]. Prenzel, N., Fischer, O. M., Streit, S., Hart, S. & Ullrich, A. The epidermal growth factor receptor family as a central element for cellular signal transduction and diversification. Endocrine-Related Cancer 2001; 8: 11-31.

[16]. Sibylle Loibl, Lorena de la Pena, Valentina Nekljudova, et al. Neoadjuvant buparlisib plus trastuzumab and paclitaxel for women with HER2+ primary breast cancer: A randomized, double-blind, placebo-controlled phase II trial (NeoPHOEBE). European Journal of Cancer, 2017, 85: 133-145.

[17]. Blackwell KL, Burstein HJ, Storniolo AM, et al. Randomized study of Lapatinib alone or in combination with trastuzumab in women with ErbB2-positive, trastuzumab-refractory metastatic breast cancer. J Clin Oncol, 2010, 28(7): 1124-1130.

[18]. S Loi, F Andre, R Maibach, et al. Abstract OT3-01-05: PANACEA (IBCSG 45-13/BIG 4-13): A phase Ib/II trial evaluating the efficacy of pembrolizumab and trastuzumab in patients with trastuzumab-resistant HER2-positive, metastatic breast cancer. DOI: 10.1158/1538-7445, SABCS15-OT3-01-05.

[19]. Daniel V.T. Catenacci, Haeseong Park, Hope Elizabeth Uronis, et al. Antitumor activity of Margetuximab plus Pembrolizumab in patients with advanced HER2+ (IHC3+) gastric carcinoma. Abstract #65, presented at 2019 ASCO Gastrointestinal Cancers Symposium.

13 APPENDICES

Appendix 1 Response Evaluation Criteria in Solid Tumors (RECIST 1.1)

Definition

At baseline, tumor lesions/lymph nodes will be categorized measurable or non-measurable as follows:

Measurable lesions

- Measurable non-nodal lesions: lesions that can be accurately measured with at least 1 dimension and have the diameter not be less than 2 times the thickness of the tomography scan (e.g., if the thickness on spiral CT scan or MRI is 5 mm, the diameter of the lesion must be ≥ 10 mm);

- Measurable nodal lesions: lymph nodes ≥ 15 mm in short diameter;

- Bone lesions selected as target lesions must be osteolytic or mixed osteolytic-osteoblastic lesions that can be assessed by CT/MRI and contain identifiable soft tissue components.

Non-measurable lesions

Non-measurable lesions refer to all other non-measurable lesions, including small lesions (e.g., if the tomography thickness is 5 mm, the long diameter of the CT or MRI scan is < 10 mm, or the short diameter of the pathological lymph node is ≥ 10 mm and < 15 mm). Examples of non-measurable lesions include osteoblastic lesions, leptomeningeal disease, ascites, pleural/pericardial effusion, inflammatory breast disease, cutaneous lymphangitis/pneumonia, abdominal masses not confirmed and followed by imaging techniques, and cystic lesions.

Special considerations regarding lesion measurability

Bone lesions, cystic lesions and lesions previously treated with local therapy require particular comments:

Bone lesions:

- Bone scans, PET scans, or plain films are not considered adequate imaging techniques to measure bone lesions, but can be used to confirm the presence or disappearance of bone lesions;

- Lytic bone lesions or mixed lytic-blastic lesions, with identifiable soft tissue components, that can be evaluated by cross sectional imaging techniques (e.g. CT or MRI) can be considered as measurable lesions if the soft tissue component meets the definition of measurability described above;

- Blastic bone lesions are non-measurable.

Cystic lesions:

Lesions that meet the criteria of radiographically defined simple cysts should not be considered as malignant lesions (neither measurable nor non-measurable) since they are, by definition, simple cysts;

Cystic lesions thought to represent cystic metastases can be considered as measurable lesions, if they meet the definition of measurability described above. However, if non-cystic lesions are present in the same patient, these are preferred for selection as target lesions.

Lesions previously treated with local therapy:

Tumor lesions situated in a previously irradiated area, or in an area subjected to other loco-regional therapy, are usually not considered measurable unless there has been demonstrated progression in the lesion. Study protocols should detailed describe the conditions under which such lesions will be considered measurable.

Specifications for measurement methods

Measurement of lesions

All measurements should be recorded in metric notation, using calipers in clinical assessment. All baseline evaluations of tumor lesion size should be measured at a time as close as possible to treatment initiation and should be completed within 28 days (4 weeks) prior to the start of the treatment.

Methods of evaluation

- All measurements should be recorded in metric symbols (mm) using a ruler or caliper. All baseline evaluations will be completed at the start of treatment whenever possible and not earlier than 4 weeks prior to treatment start.

- Imaging-based evaluation is preferred when evaluating the effect of anti-tumor therapy based on imaging and clinical findings.

- In order to best evaluate the subjects, the same evaluation methods and techniques should be used to characterize lesions identified and reported at baseline and during follow-up. Contrast-enhanced CT of the chest, abdomen, and pelvis will be performed preferentially using 5 mm slice serial scanning. When serial slice scanning is used, the slice thickness of CT/MRI scan should not exceed 8 mm. If the patient is known to be allergic to CT contrast at baseline or has an allergic reaction during the trial, the following imaging method acceptable to the subject may be used instead: chest non-contrast CT (MRI is not recommended due to respiratory artifacts) plus contrast-enhanced MRI imaging of the abdomen and pelvis.

- A change in methodology can be defined as a change in contrast (e.g., regardless of the reason for the changes, the same technique will be used, e.g., CT, but from the use of contrast to the absence of contrast, and vice versa), and it can also be defined as a change in technical methods (e.g., from CT to MRI, and vice versa), or any other imaging methods. As a result of the methodological change, the overall lesion response will be evaluated as UNK by default.

- FDG-PET: It can be used as a complement to CT scans when evaluating disease progression, especially for possible "new" lesions. New lesions can be identified on the basis of FDG-PET imaging according to the following rules:

  - A negative FDG-PET at baseline and a positive FDG-PET at follow-up can be considered PD signs based on new lesions.
  - No FDG-PET at baseline and a positive FDG-PET at follow-up:
  - If a positive FDG-PET at follow-up is confirmed by CT to be disease at a new site, it is regarded as PD.
  - If a positive FDG-PET at follow-up is not disease at a new site as confirmed by CT, additional CT follow-up is required to determine if progression does occur at that site (if any, the date of PD is the date of the initial CT scan abnormality).
  - If disease at a prior site corresponding to a positive FDG-PET at follow-up does not belong to disease progression on the basis of anatomical imaging as confirmed by CT, it is not regarded as PD.

- Chest X-ray examination: lesions found by chest X-rays can be considered measurable when lesions are clearly circumscribed and surrounded by inflated lungs. However, CT is preferred. Chest X-rays will not be performed in this study to evaluate measurable lesions.

- Ultrasound (US): US should not be used to measure tumor lesions when the primary endpoint of the study is an objective response evaluation. However, it may be an alternative to clinical measurements of superficial palpable lymph nodes, subcutaneous lesions, and thyroid nodules. US can also be used to identify superficial lesions that have disappeared completely, usually evaluated by clinical examination.

- Endoscopy and laparoscopy: the use of endoscopy and laparoscopy for objective tumor evaluation has not been fully and extensively validated. They need to be applied under specific conditions, i.e. advanced equipment and a high level of expertise, which can only be provided by certain central institutions. Therefore, the use of such techniques for objective tumor response evaluation is limited to dedicated sites for confirmation purposes. However, when biopsy results are available, such techniques can play a role in confirming a pathologic complete response.

- Tumor markers: tumor markers alone cannot be used to evaluate response. However, certain specific diseases and a variety of confirmed tumor markers (e.g., CA-125 for ovarian cancer, PSA for prostate cancer, α-FP, LDH, and β-hCG for testicular cancer) can be combined to be referred to as non-target disease. If markers are above the ULN at the start of treatment, these markers must return to normal levels when all lesions of subjects have disappeared and are clinically considered complete response.

- Cytology and histology: cytology and histology can be used to differentiate between patients with PR and CR in rare cases (e.g.,to differentiate whether post-treatment residuals are benign or malignant in tumor types such as germ cell tumors). When a measurable tumor meets criteria for response or stable disease, cytology of the effusion is required during treatment to determine the nature of the tumor or worsening. In this case, response can be distinguished from stable disease (effusion may be a side effect of treatment) or progressive disease (if proven to be tumor-derived) by cytology of the collected liquid samples.

- Clinical examination: only superficial clinical lesions (ie, skin nodules and palpable lymph nodes) are considered measurable. It is recommended to record the skin lesions with color photography, including the use of a ruler to estimate the size of the lesion.

Tumor response assessment

Assessment of overall tumor burden and measurable lesion

To assess objective response or potential subsequent progression, it is necessary to assess the overall tumor burden of all tumor lesions at baseline and use this as a reference for subsequent measurements. Only patients with measurable disease at baseline should be included in protocols where objective tumor response is the primary endpoint. Measurable disease is defined by the presence of at least one measurable lesion. In studies where the primary endpoint is disease progression (either time to progression or proportion with progression at a fixed date), the protocol must specify if enrollment is restricted to those with measurable disease or whether patients having non-measurable disease only are also eligible.

Baseline documentation of "target" and "non-target" lesions

When more than one measurable lesion is present at baseline, all lesions up to a maximum of five lesions total (and a maximum of two lesions per organ) representative of all involved organs should be identified as target lesions and will be recorded and measured at baseline (this means in instances where subjects have only one or two organ sites involved a maximum of two and four lesions respectively will be recorded).

Target lesions should be selected on the size basis (lesions with the longest diameter), be representative of all involved organs, and have good reproducibility for measurement. At times when the largest lesion cannot be used for repeated measurement, the next largest lesion that can be measured reproducibly should be selected.

Lymph nodes are normal tissue and can be detected radiographically even if there is no tumor metastasis. Pathological nodes which are defined as measurable nodes and even target lesions must meet the criterion of a short diameter of ≥ 15 mm by CT scan. Only short diameters need to be detected at the baseline. The short diameter of the node is the diameter normally used by radiologists to judge if a node is involved by solid tumor. Nodal size is generally reported in terms of two-dimensional data from image detection (for CT scan this is always the axial plane; for MRI, a plane will be chosen from the axial, sagittal, or coronal plane). The minimum value of these measurements is the short diameters. For example, an abdominal node which is reported as being 20 mm × 30 mm, has a short diameter of 20 mm, and qualifies as a malignant, measurable node. In this example, 20 mm should be recorded as the node measurement. All other pathological nodes those with short diameter ≥ 10 mm but < 15 mm should be considered as non-target lesions. Nodes that have diameters < 10 mm are considered non-pathological and should not be recorded or followed.

A sum of the diameters (SOD) (longest for non-nodal lesions and shortest for nodal lesions) for all target lesions will be reported as the baseline sum of diameters. If lymph nodes are to be included in the sum, then as noted above, only the short diameter is added into the sum. The baseline SOD will be used as reference for the baseline level of the disease.

All other lesions (or sites of disease) including pathological lymph nodes should be identified as non-target lesions and should also be recorded at baseline. For example, recorded as 'present',

'missing' or in rare cases 'unequivocal progression'. In addition, it is possible to record multiple non-target lesions involving the same organ as a single item on the CRF (e.g., "multiple enlarged pelvic lymph nodes" or "multiple liver metastases").

Response Criteria

The evaluation criteria for target lesions are presented in the table below

Evaluation of target lesions	
Complete Response (CR):	Disappearance of all target lesions. Any pathological lymph nodes (whether target or non-target) must have reduction in short axis to < 10 mm.
Partial Response (PR):	At least a 30% decrease in the SOD of target lesions, taking as reference the baseline sum diameters.
Progressive Disease (PD):	At least a 20% increase in the SOD of target lesions, taking as reference the smallest sum on study (this includes the baseline sum if that is the smallest on study). In addition, the sum must also demonstrate an absolute increase of at least 5 mm (the appearance of one or more new lesions is also considered progression)
Stable Disease (SD):	Neither sufficient shrinkage to qualify for PR nor sufficient increase to qualify for PD, taking as reference the smallest sum diameters while on study.

Special notes on the assessment of target lesions:

Lymph nodes: Lymph nodes identified as target lesions should always have the actual short diameter measurement recorded (measured in the same anatomical plane as the baseline examination), even if the nodes regress to below 10 mm on study. This means that when lymph nodes are included as target lesions, the ‘sum’ of lesions may not be zero even if complete response criteria are met, since a normal lymph node is defined as having a short diameter of < 10 mm. CRFs or other data collection methods may therefore be designed to have target nodal lesions recorded in a separate section where, in order to qualify for CR, each node must achieve a short diameter < 10 mm. For PR, SD and PD, the actual short diameter measurement of the nodes is to be included in the sum of target lesions.

Target lesions that become "too small to measure": While on study, all lesions (nodal and non-nodal) recorded at baseline should have their actual measurements recorded at each subsequent evaluation, even when very small (e.g., 2 mm). However, sometimes CT images of lesions or lymph nodes are so faint that even the radiologist may feel it challenging to assigning an exact measure and may report them as being "too small to measure". When this occurs, it is important that a value is recorded on the eCRF. If it is the opinion of the radiologist that the lesion has likely disappeared, the measurement should be recorded as 0 mm. If the lesion is believed to be present and is faintly seen but too small to measure, a default value of 5 mm should be assigned. (Note: It is less likely that this rule will be used for lymph nodes since they usually have a definable size when normal and are frequently surrounded by fat such as in the retroperitoneum; however, if a lymph node is believed to be present and is faintly seen but too small to measure, a default value of 5 mm should be assigned in this circumstance as well). This default value is derived from the CT slice thickness of 5 mm, which should not be changed with varying CT slice thickness. The measurement of these lesions is potentially non-reproducible, therefore providing this default value will reduce the risks of incorrect evaluation. To reiterate, however, if the radiologist is able to provide an actual measure, that should be recorded, even if it is below 5 mm.

Lesions that split or coalesce: When non-nodal lesions are split into "fragments", the longest diameters of the fragmented portions should be added together to calculate the SOD of the target lesion. Similarly, as lesions coalesce, a plane between them may be maintained that the maximal diameter of each individual lesion can be calculated. If the lesions have truly coalesced such that they are no longer separable, the vector of the longest diameter in this instance should be the maximal longest diameter for the coalesced lesion.

Evaluation criteria for non-target lesions are presented in the table below

Evaluation of non-target lesions	
Complete Response (CR):	Disappearance of all non-target lesions and normalisation of tumor marker level. All lymph nodes must be non-pathological in size (short diameter <10 mm).
Non-CR/Non-PD:	Persistence of one or more non-target lesion(s) and/or maintenance of tumor marker level above the normal limits.
Progressive Disease (PD):	Unequivocal progression of existing non-target lesions. Note: the appearance of one or more new lesions is also considered progression.

Special notes on assessment of progression of non-target disease:

The concept of progression of non-target disease requires additional explanation as follows: When the patient also has measurable disease. In this setting, to achieve "unequivocal progression" on the basis of the non-target disease, there must be an overall level of substantial worsening in non-target disease such that, even in presence of SD or PR in target disease, the overall tumor burden has increased sufficiently to merit discontinuation of therapy. A modest ‘increase’ in the size of one or more non-target lesions is usually not sufficient to quality for unequivocal progression status. The designation of overall progression solely on the basis of change in non-target disease in the face of SD or PR of target disease will therefore be extremely rare.

Evaluation of best overall response

The best overall response (BOR) is the best response recorded from the start of the study treatment until the end of treatment taking into account any requirement for confirmation. On occasion a response may not be documented until after the end of therapy, so protocols should clarify if post-treatment assessments are to be considered in determination of BOR. Protocols must specify how any new therapy introduced before progression will affect best response designation. The assignment of a patient's BOR will depend on the findings of both target and non-target disease and will also take into consideration the appearance of new lesions. Furthermore, it also depends on the trial's nature, protocol requirements and outcome measurement criteria. Specifically, in non-randomized trials where response is the primary endpoint, confirmation of PR or CR is needed to deem either one the BOR.

The timepoint assessments for subjects with tumor response with target lesions and for subjects with only non-target lesions (no target lesions) are provided in the table below, respectively.

Time point response – patients with target (+/- non-target) disease

Target lesions	Non-target lesions	New lesions	Overall response
CR	CR	None	CR
CR	Non-CR/non-PD	None	PR
CR	NE	None	PR
PR	Non-PD or not fully evaluable	None	PR
SD	Non-PD or not fully evaluable	None	SD
Not fully evaluable	Non-PD	None	NE
PD	Any condition	Yes or no	PD
Any condition	PD	Yes or no	PD
Any condition	Any condition	Yes	PD

CR = complete response, PR = partial response, SD = stable disease, PD = progressive disease, and NE = not evaluable

Timepoint tumor response-subjects with non-target lesions only

Non-target lesions	New lesions	Overall response
CR	None	CR
Non-CR or non-PD	None	Non-CR or non-PD
Not fully evaluable	None	NE
Equivocal PD	Yes or no	PD
Any condition	Yes	PD

Note: "Non-CR/non-PD" refers to efficacy that is superior over SD for non-target lesions. Since SD is increasingly used as an endpoint for assessment of efficacy, non-CR/non-PD efficacy has been developed so to assign this category when no lesions can be measured is not advised. For equivocal findings of progression (e.g. very small and undefined new lesions; cystic changes or necrosis in existing lesions), treatment may continue until the next scheduled assessment. If disease progression is confirmed during the next scheduled assessment, the date of progression should be the earlier date when progression is suspected.

Missing assessments and inevaluable designation

When no lesion imaging or measurement is performed at a particular time point, the subject is not evaluable (NE) at that time point. If only a subset of lesion measurements is made at an assessment, usually the case is also considered NE at that time point, unless a convincing argument can be made that the contribution of the missing lesion (s) would not change the response evaluation at the assigned time point. This would be most likely to happen in the case of progressive disease. For example, if a subject has a baseline SOD of 50 mm with three measured lesions, but only two lesions with a SOD of 80 mm are evaluable at follow-up, then the subject will be assessed as PD, regardless of the contribution of the missing lesion.

Best overall response

Best overall response (BOR) is defined as the best response from the start of study drug treatment until disease progression. In some cases, the determination of BOR needs to be confirmed, e.g., confirmation of the first occurrence of complete response and partial response is required in this study. Tumor response evaluated after the start of a new anti-tumor therapy should not be included in the evaluation of BOR:

- Confirmed complete response (confirmed CR): complete response will be assessed at least 2 times before disease progression and the interval between the two assessments will be at least 4 weeks;
- Confirmed partial response (confirmed PR): partial response will be assessed at least 2 times before disease progression which do not meet the CR, and the interval between the two assessments will be at least 4 weeks;
- Stable disease (SD): stable disease will be assessed at least once after > 6 weeks of treatment with the study drug which do not meet the CR or PR;
- Progressive disease (PD): disease progression will be assessed within 12 weeks (including 12 weeks) of study drug treatment which do not meet the CR, PR, and SD. If a subject experiences rapid disease progression without corresponding radiographic evaluation, disease progression can only be assessed if there is clear evidence of clinical deterioration and/or the subject dies from the underlying tumor disease;
- Unknown (UNK): other conditions (e.g., not meet the confirmed CR or PR, no SD after 6 weeks of treatment, or no early disease progression within 12 weeks).

Appendix 2 Immune Response Evaluation Criteria in Solid Tumors (iRECIST)

This new classification is based on the results of recent clinical studies of tumor immunotherapy, after which tumor regression or stabilization may still occur even if some new lesions appear at the time of treatment is initiated, or there is no significant increase in total tumor burden. This study will introduce the concept of iRECIST based on RECIST 1.1 using one-dimensional measurement of tumor lesions (Lesley Seymour et al 2017).

For iRECIST criteria, new target lesions (NL-T) (recorded separately on the eCRF and not included in the baseline target lesion tumor burden evaluation) and new non-target lesions (NL-NT) (recorded separately on the eCRF) will be considered. When PD as defined by RECIST 1.1 first occurs, the status iUPD (immune unconfirmed disease progression) will be reset according to iRECIST criteria, and iUPD needs to be confirmed after 4 to 8 weeks. The clinical relevance of iRECIST criteria remains to be verified, therefore, discontinuation of study drug treatment is not mandatory in the event of immune-related disease progression (as judged by iRECIST), even if the disease progression is confirmed on subsequent 4 to 8 weeks of imaging.

Interpretation of time point tumor response is presented in the following table:

Target lesions	Non-target lesions	New lesions	Time point tumor response (Non-iUPD in previous tumor evaluation)	Time point tumor response (iUPD in previous tumor assessment)
iCR	iCR	None	iCR	iCR
iCR	Non-iCR/non-iUPD	None	iPR	iPR
iPR	Non-iCR/non-iUPD	None	iPR	iPR
iSD	Non-iCR/non-iUPD	None	iSD	iSD
iUPD, no change or shrinkage	iUPD, no change or shrinkage	Yes	N/A	iCPD if the previous new lesions enlarge (≥ 5 mm increase in length and diameter of NL-T; or progression of NL-NT), and iUPD if the previous new lesions do not meet the above criteria for enlargement
iSD、iPR、iCR	iUPD	None	iUPD	iUPD unless NL-NT progresses
iUPD	Non-iCR/non-iUPD; or iCR	None	iUPD	iUPD unless NL-T increases by ≥ 5 mm in length and diameter
iUPD	iUPD	None	iUPD	iCPD if the length and diameter increase ≥ 5 mm in previous target lesion; or progression of non-

				target lesion; otherwise, iUPD
iUPD	iUPD	Yes	iUPD	iCPD if the length and diameter increase ≥ 5 mm in previous target lesions; or progression of non-target lesions; iCPD if the tumor burden or number of previous new lesions increase; otherwise, iUPD
Non-iUPD or progression	Non-iUPD or progression	Yes	iUPD	iCPD if the previous new lesions enlarge (≥ 5 mm increase in length and diameter of NL-T; or progression of NL-NT), and iUPD if the previous new lesions do not meet the above criteria for enlargement

The following table lists how the overall tumor response will be judged based on the time point tumor response:

Time point 1	Time point 2	Time point 3	Time point 4	Time point 5	iBOR
iCR	iCR, iPR, iUPD, or NE	iCR, iPR, iUPD, or NE	iUPD	iCPD	iCR
iUPD	iPR, iSD, or NE	iCR	iCR, iUPD, or NE	iCR, iPR, iSD, iUPD, iCPD, or NE	iCR
iUPD	iPR	iCR, iPR, iUPD, or NE	iPR, iSD, iUPD, NE, or iCPD	iPR, iSD, iUPD, NE, or iCPD	iPR
iUPD	iSD	iSD, iUPD, or NE	iSD, iUPD, iCPD, or NE	iSD, iUPD, iCPD, or NE	iSD
iUPD	iCPD	Either	Either	Either	iCPD
iUPD	iUPD (no iCPD)	iCPD	Either	Either	iCPD
iUPD	NE	NE	NE	NE	iUPD

Reference: Lesley Seymour, Jan Bogaerts, Andrea Perrone, et al. iRECIST: guidelines for response criteria for use in trials testing immunotherapeutics. Lancet Oncol 2017; 18: e143-152.

Appendix 3 Management Principles of Immune-related Adverse Events

Only general management principles for relatively common irAEs are listed below. The treatment of other immune-related toxicity, including rare but serious immune-related toxicity (such as immune-related eye toxicity and CNS toxicity) can refer to the latest guidelines from ESMO (*J. B. A. G. Haanen, F. Carbonnel, C. Robert et al. Management of toxicities from immunotherapy: ESMO clinical practice guidelines for diagnosis, treatment and follow-up. Annals of Oncol 2017; 28(S4): 119-142*) and NCCN (*Julie R Brahmer, Christina Lacchetti, Bryan J Schneider et al. Management of immune-related adverse events in patients treated with immune checkpoint inhibitor therapy: American Society of Clincial Oncology Clinical Practice Guideline. JCO 2018; 36: 1714-1768*). The addition of drugs such as sulfamethoxazole/trimethoprim to prevent opportunistic infections caused by pneumocystis jirovecii and other sources is recommended for subjects who have received hormonal therapy for more than 3 weeks.

Management of immune-related pneumonitis

CTCAE v5.0 Grade	Dose modification of KN046	Management principles	Diagnosis and differential diagnosis
**Grade 1**<br>Asymptomatic, radiographic changes only, manifested as ground-glass degeneration or nonspecific interstitial lung disease	• Interrupt KN046 as appropriate	• Monitor the symptoms and signs every 2-3 days<br>• Pulmonary imaging follow-up every 3 weeks<br>• Respiratory consultation. Bronchoscopy if indicated	Tumor metastasis, bacterial (e.g., chlamydial, listerial) or viral infections should be ruled out
**Grade 2**<br>Mild/moderate symptomatic dyspnea, cough and chest pain	• Interrupt KN046<br>• Resumption of KN046 is allowed if recovered to ≤ Grade 1 within 12 weeks and the hormone dose has been reduced to methylprednisolone 10 mg/day PO (or equivalent of other hormones)<br>• **Permanently discontinue KN046 if ≥ Grade 2 pneumonia recurs after resuming KN046 administration**<br>• **Permanently discontinue KN046 if ≥ Grade 2 pneumonia lasting more than 4 weeks after active treatment**	• Exclude infectious diseases<br>• Pulmonary function test<br>• Respiratory consultation. Bronchoscopy/bronchoalveolar lavage if indicated<br>• Empiric antibiotic therapy if infection is suspected<br>• Monitor symptoms and signs daily, and consider hospitalization<br>• If symptoms and signs do not resolve after 48 hours of anti-infective therapy, it is recommended to start prednisolone 1 mg/kg/day (or equivalent of other hormones); taper the dose of corticosteroids after symptoms resolve for no less than 4 weeks	
**Grade 3-4**<br>Severe new symptoms; new or worsening hypoxia; life-threatening; dyspnea; acute respiratory distress syndrome	• Permanently discontinue KN046	• Pulmonary function test<br>• Respiratory consultation; bronchoscopy/bronchoalveolar lavage is recommended<br>• •Administer methylprednisolone 2-4 mg/kg/day IV	

		• If symptoms do not resolve 48-72 hours after high-dose hormone pulse, infliximab 5 mg/kg every 2 weeks is used; for subjects with concomitant hepatic impairment, mycophenolate mofetil may be considered in place of infliximab<br>• Tapering of hormone should last not less than 6 weeks<br>• Adding prophylactic antibiotics to prevent opportunistic infections in the course of high-dose corticosteroids	

Management of immune-related colitis

CTCAE v5.0 Grade	Dose modification of KN046	Management principles	Diagnosis and differential diagnosis
**Grade 1**<br>Increased diarrhoea < 4 times/day from baseline	• Continue KN046	• Treatment of diarrhea symptoms (loperamide, oral fluid replacement, avoidance of a high-fiber and high-lastose diet)<br>• Add diphenoxylate, atropine sulfate, and budesonide if Grade 1 diarrhea persists for > 1 week<br>• Add prednisolone 0.5-1 mg/kg if Grade 1 diarrhea persists for > 2 weeks<br>• Consider endoscopy if the symptom persists	Need to rule out tumor metastasis, bacterial, parasitic or viral gastroenteritis, or inflammatory bowel disease
**Grade 2**<br>Increased diarrhoea from baseline, 4-6 times/day, or with abdominal pain/bloody stools/nausea	• Interrupt KN046 until recovery to ≤ Grade 1	• • Consider endoscopy to rule out/determine colitis if Grade 2 diarrhea persists for > 1 week or Grade 1-2 diarrhea with bloody stools<br>• • Diphenoxylate, atropine sulfate, and budesonide may be added<br>• If symptoms persist for > 1 week, add oral prednisolone 0.5 to 1 mg/kg (or equivalent of other hormones). Hormone tapering is not less than 4 weeks after symptoms improve; if diffuse ulcers are seen endoscopically, the risk of perforation should be noted, and the rate of hormone reduction should be further slowed down.	
**Grade 3-4**<br>Watery diarrhea ≥ 7 times/day or life-threatening	• Permanent discontinue KN046 for Grade 3-4 colitis<br>• Interrupt KN046 for Grade 3-4 diarrhea<br>• Permanently discontinue KN046 if hormone dose cannot be reduced to ≤ 10 mg/day within 12 weeks	• Hospital admission<br>• Grade 3-4 colitis<br>• Initiate 1-2 mg/kg/day prednisolone (or equivalent of other hormones). Tapering of hormone lasts no less than 4 weeks after symptom relief<br>• Grade 3-4 diarrhea<br>• Perforation other than abdominal plain scan or CT	

		• Gastroenterology consultation, consider endoscopic biopsy<br>• Initiate treatment with intravenous methylprednisolone 125 mg, and subsequently modify to oral prednisolone 1-2 mg/kg/day. If symptoms do not resolve to ≤ Grade 1, corticosteroid tapering is no less than 1 month; for subjects with diffuse or severe ulcers or with bleeding, hormone tapering is no less than 6-8 weeks<br>• If symptoms do not resolve 48-72 hours after hormonal therapy, treat with infliximab 5 mg/kg every 2 weeks. Discontinue Infliximab after symptom relief and tapering of hormone lasts for 6-8 weeks. If symptoms worsen during tapering of hormone, restart high-dose corticosteroid ± infliximab treatment, followed by a longer tapering of hormone. **Warning:** Infliximab is contraindicated in patients with intestinal perforation or accompanying with septicemia<br>• Consider surgical intervention in patients who do not respond to these treatments	

Management of immune-related endocrine adverse events

<table>
<tr><th>CTCAE v5.0 Grade</th><th>Dose modification of KN046</th><th>Management principles</th><th>Diagnosis and differential diagnosis</th></tr>
<tr><td>Grade 1-2
• Hyperthyroidism
• Hypothyroidism
• Thyroiditis</td><td>• Continue KN046</td><td>• More frequent monitoring of thyroid function and serum electrolytes (e.g., every 3-6 weeks) until recovery to baseline
• Consider alternative therapy or thyroid suppression treatment as indicated</td><td rowspan="3">Need to rule out brain tumor metastases, septicaemia, and/or severe infections</td></tr>
<tr><td>Grade 3-4
• Hyperthyroidism
• Hypothyroidism
• Thyroiditis</td><td>• Interrupt KN046 until alternative therapy is stable and symptoms resolve</td><td>• Endocrinology consultation
• Consider alternative therapy or thyroid suppression treatment as indicated</td></tr>
<tr><td>Grade 1-4
• Adrenal insufficiency
• Hypophysitis
• Panhypopituitarism</td><td>• Interrupt KN046 until alternative therapy is stable and symptoms resolve</td><td>• Endocrinology consultation
• Thyroxine and/or hormone replacement therapy for adrenal insufficiency
• Consider pituitary imaging (gadolinium-containing pituitary MRI) if Grade 1-2 hypophysitis is suspected
• Initiate IV methylprednisolone in the event of manifestations of pituitary crisis (grade 3-4 hypophysitis with adrenal insufficiency, dehydration, and electrolyte disturbance such as hyponatremia and hyperkalemia)</td></tr>
</table>

Management of immune-related hepatitis

<table>
<tr><th>CTCAE v5.0 Grade</th><th>Dose modification of KN046</th><th>Management principles</th><th>Diagnosis and differential diagnosis</th></tr>
<tr><td>Grade 1
• ALT or AST > 1-3 × ULN
• Patients with ALT or AST increased from baseline and ≤ Grade 1 toxicity increased from baseline</td><td>• Continue KN046</td><td>• Monitor liver function weekly</td><td rowspan="3">Exclude drug factors (e.g., statins, antibiotics), and history of alcohol use; exclusion of viral factors (e.g., anti-HAV/HBV/HCV antibodies, HEV PCR), other liver diseases (anti-ANA/SMA/LKM/SLA/LP/LCI/iron test), tumor metastasis</td></tr>
<tr><td>Grade 2
• ALT or AST > 3-5 × ULN
• Patients with ALT or AST increased from baseline and ≤ Grade 1 toxicity increased from baseline, and ≤ 5 × ULN</td><td>• Interrupt KN046</td><td>• Reexamination of liver function every 3 days
  • Initiate oral hormonal therapy (prednisolone 1 mg/kg/day) if liver function does not relieve in reexamination</td></tr>
<tr><td>Grade 3-4
• ALT or AST > 5 × ULN
• and/or total bilirubin > 3 × ULN</td><td>• Permanently discontinue KN046</td><td>• Etiological diagnosis by liver biopsy if indicated
• IV methylprednisolone 2 mg/kg/day, and convert to oral prednisolone 1-2 mg/kg/day (or other equivalent hormones) for the subjects with improved liver function within 24-48 hours, and start slowly tapering over no less than 4 weeks
• Add mycophenolate mofetil 500 mg bid therapy to hormonal pulse therapy if no improvement of liver function within 48 hours
• The course of immune hepatitis usually recurs, and attention should be paid to the patients during hormone tapering; if necessary, a new round of high-dose hormonal therapy followed by dose reduction should be restarted
• Infliximab has hepatotoxicity and is not recommended for the treatment of immune hepatitis</td></tr>
</table>

Management of immune-related rash

CTCAE v5.0 Grade	Dose modification of KN046	Management principles	Diagnosis and differential diagnosis
**Grade 1-2**<br>• Skin rash with/without symptoms, skin involvement < 30% BSA	• Continue KN046	• Symptomatic management<br>• Topical hormones, e.g., betamethasone 0.1% ointment, hydrocortisone 1% ointment<br>• Urea ointment in combination with oral antipruritics (e.g., diphenhydramine hydrochloride)<br>• Add oral hormones as appropriate (e.g., Grade 2 dermatitis)<br>• Grade 2 skin toxicity with significant clinical symptoms requires treatment as Grade 3 skin toxicity	Skin metastases, infections, or allergic dermatitis should be excluded
**Grade 3**<br>• Skin involvement ≥ 30% BSA	• Interrupt KN046	• Dermatology consultation; consider skin biopsy for confirmation of diagnosis<br>• Initiate oral hormonal therapy, prednisolone 1 mg/kg/day (or other equivalent hormones) until recovery to ≤ Grade 1, and start hormone tapering over no less than 4 weeks	
**Grade 4**<br>• Skin involvement ≥ 30% BSA, with significant symptoms	• Permanently discontinue KN046	• Dermatology consultation<br>• IV methylprednisolone 1-2 mg/kg/day until recovery to ≤ Grade 1, and start hormone tapering over no less than 4 weeks	

Management of immune nephritis

CTCAE v5.0 Grade	Dose modification of KN046	Management principles	Diagnosis and differential diagnosis
**Grade 1**<br>• Nephritis<br>• Autoimmune nephropathy	• Continue KN046<br>• Interrupt KN046 without improvement in symptomatic management	• Symptomatic management<br>• Monitor renal function weekly until return to baseline	Obstructive nephropathy, tumor metastasis, and drug-induced renal injury need to be excluded
**Grade 2**<br>• Renal insufficiency	• Interrupt KN046<br>• **≥ Grade 2 renal toxicity lasting > 7 days or worsening after active treatment requires permanent discontinuation of KN046**	• Nephrology consultation, renal B-ultrasound, consider renal biopsy<br>• Initiate oral hormonal therapy, prednisolone 1-2 mg/kg/day (or other equivalent hormones) until recovery to ≤ Grade 1, and start hormone tapering over no less than 4 weeks<br>• Persistent for > 7 days or worsening after hormonal therapy requires treatment as Grade 3-4	
**Grade 3-4**<br>• Renal insufficiency	• Permanently discontinue KN046	• Nephrology consultation, renal B-ultrasound, consider renal biopsy<br>• Monitor creatinine levels daily<br>• Initiate oral hormonal therapy, prednisolone 1-2 mg/kg/day (or other equivalent hormones) until recovery to ≤ Grade 1, and start hormone tapering over no less than 4 weeks	

Management of immune-related myocarditis

<table>
<tr><th>CTCAE v5.0 Grade</th><th>Dose modification of KN046</th><th>Management principles</th><th>Diagnosis and differential diagnosis</th></tr>
<tr><td>Grade 1
• Cardiac abnormalities (e.g., myocardial zymogram, ECG)</td><td rowspan="3">• Discontinue KN046</td><td rowspan="3">• Cardiology consultation
• ECG, troponin, BNP, echocardiography, and CXR
• Immediately initiate high-dose hormonal therapy
  • 1-2 mg/kg prednisone, IV or oral based on severity of symptoms
• Cardiovascular symptoms are treated per ACC/AHA guidelines
• Subjects with troponin or conduction abnormalities requiring admission to a cardiac ICU
• Consider increasing the dose of hormones (methylprednisolone 1 g/day) and adding mycophenolate mofetil, infliximab, or antithymocyte globulin for subjects who do not respond to high-dose hormones</td><td rowspan="3">Myocardial infarction, viral myocarditis, infective valvular disease, etc., need to be excluded</td></tr>
<tr><td>Grade 2
• Cardiac abnormality with mild symptoms</td></tr>
<tr><td>Grade 3-4
• Moderate to severe cardiac insufficiency requiring intravenous administration or being life-threatening</td></tr>
</table>

Appendix 4 Contraception Guidance

Contraception must be highly effective.

According to the Clinical Research Collaboration Group (CTFG) Guideline on Recommendations Related to Contraception and Pregnancy Testing in Clinical Trials, highly effective methods of contraception are defined as an annual contraceptive failure rate of less than 1% with proper continuous use, e.g.:

- Hormones (containing estrogen and progesterone) combined with inhibition of ovulation[1] (oral, transvaginal, transdermal) contraception
- Progesterone combined with inhibition of ovulation[1] (oral, injectable, implantable[2])
- Intrauterine device (IUD)[2]
- Intrauterine hormone-releasing system (IUS)[2]
- Bilateral tubal ligation[2]
- Vasectomy for partner[2,3]
- Abstinence[4]

[1] Hormonal contraception may interact with the study drug to reduce the effect of contraception

[2] Such contraceptive methods are less affected by human factors

[3] For women of childbearing potential, this method is a highly effective method of birth control if the only male sexual partner has undergone vasectomy and the procedure is successful

[4] If heterosexual contact is refrained throughout the study, the abstinence can be considered a highly effective method of contraception. The reliability of abstinence contraception requires consideration of the duration of the entire study and the lifestyle of the subject

Appendix 5 Eastern Cooperative Oncology Group Performance Status

**Eastern cooperative oncology group (ECOG) performance status[1]**	
**Grade**	**ECOG**
0	Fully active, fully able to carry on all pre-disease performance without restriction
1	Heavy physical activities are limited, but completely able to walk and can do light or sitting work, such as light house work and office work
2	Able to walk and take care of himself/herself, but unable to work. Up and about > 50% of waking hours
3	Capable of only limited self-care, confined to bed or chair > 50% of waking hours
4	Completely disabled. Unable to take care of oneself. Bedridden
5	Death

[1]Oken MM, Creech RH, Tormey DC et al. Toxicity and Response Criteria of the Eastern Cooperative Oncology Group. Am J Clin Oncol. 1982; 5:649-55.

Appendix 6 IHC Interpretation of HER2 Detection in Gastric Cancer

HER2 IHC scoring criteria for gastric cancer (2018 ASCO/CAP update)

Score and assessment	Surgery specimen-staining morphology	Biopsy specimen-staining morphology
IHC 0 (Negative)	No response or < 10% of tumor cells with a membrane response	All cells are unresponsive.
IHC 1+ (Negative)	≥ 10% of tumor cells with weak/undetectable membrane response; some membranes of cells with response	Tumor cell clusters[a] with weak/undetectable membrane response, regardless of staining percentage of tumor cells
IHC 2+ (Uncertain[b])	≥ 10% of tumor cells with weak to moderate intact basolateral or lateral membrane responses	Tumor cell clusters[a] with weak to moderate intact basolateral or lateral membrane responses, regardless of staining percentage of tumor cells
IHC 3+ (Positive)	≥ 10% of tumor cells with strong intact basolateral or lateral membrane responses	Tumor cell clusters[a] with strong intact basolateral or lateral membrane responses, regardless of staining percentage of tumor cells

[a]Tumor cell clusters are defined as clusters of at least five positive tumor cells

[b]ISH test must be performed on the same sample
