# Supplementary material for: Efficacy and safety of KN026, a bispecific anti-HER2 antibody, in combination with KN046, an anti-CTLA4/PD-L1 antibody, in patients with advanced HER2-positive nonbreast cancer: a combined analysis of a phase Ib and a phase II study
Source: Signal Transduct Target Ther. 2025 Mar 19;10:104. doi: 10.1038/s41392-025-02195-x (PMC11923254; doi:10.1038/s41392-025-02195-x)
Supplement: Supplementary file 3 — The main protocol revision of the phase Ib study from V2.0 to V6.1 [file 41392_2025_2195_MOESM3_ESM.pdf]

KN046-IST-02 study major revisions from v2.0 to v6.1

From v2.0 to 3.0

1) Drug pharmacokinetics and immunogenicity were deleted.
2) The sample size of dose 1 was increased to approximately 20 patients.
3) The definition of HER2-positive is limited to IHC3+ or HER2 gene amplification and patients with HER2 mutations or low expression were no longer enrolled. *(Considering the efficacy for these patients)*
4) The dose expansion phase increased the cohort of KN026 combined with XELOX (2 cycles) followed by KN026 combined with KN046 for 1st line HER2 positive gastric/gastroesophageal cancer (GC/GEJ).

From v3.0 to 4.0

1) Two dose levels were added, which were dose 4 (KN026 20 mg/kg Q2W and KN046 5 mg/kg Q2W) and dose 5 (KN026 30 mg/kg Q2W and KN046 5 mg/kg Q2W).

From v4.0 to 5.1

1) Drug dose was no longer escalated to dose 5. *(Considering the dose level is high enough, because KN026 is used as a monotherapy at a dose of 30 mg/kg Q3W, even though no DLT was reported at previous dose level.)*
2) The dose expansion phase increased the cohorts of KN026 and KN046 combined with lenvatinib for 1st line and late line HER2 positive GC/GEJ.

From v5.1 to 6.1

1) The cohorts of KN026 and KN046 combined with lenvatinib for 1st line and late line HER2 positive GC/GEJ were deleted. *(Considering that KN026 combined with KN046 had showed good efficacy for 1st line HER2 positive GC/GEJ)*
2) Stop enrollment of the cohort of KN026 combined with XELOX (2 cycles) followed by KN026 combined with KN046 cohort for 1st line HER2 positive GC/GEJ. *(Considering that KN026 combined with KN046 had showed good efficacy for 1st line HER2 positive GC/GEJ)*
3) The end of the study is specified as "1 year after the last dose of all patients, or the last patient completes the last study-related telephone contact or visit, withdraws from the study or lost to follow-up (i.e., the investigator is unable to contact the patients), or all patients die or withdraw early from the study, or the investigator assesses that study expectations have been met (e.g., completion of the primary endpoint), whichever occurs first."
