# Supplementary material for: Efficacy and safety of KN026, a bispecific anti-HER2 antibody, in combination with KN046, an anti-CTLA4/PD-L1 antibody, in patients with advanced HER2-positive nonbreast cancer: a combined analysis of a phase Ib and a phase II study
Source: Signal Transduct Target Ther. 2025 Mar 19;10:104. doi: 10.1038/s41392-025-02195-x (PMC11923254; doi:10.1038/s41392-025-02195-x)
Supplement: Supplementary file 4 — The protocol of the Phase II study [file 41392_2025_2195_MOESM4_ESM.pdf]

CLINICAL STUDY PROTOCOL

KN026 / KN046

A Phase 2 Clinical Study to Evaluate the Efficacy, Safety, and Tolerability of KN026 in Combination with KN046 in Patients with HER2-positive Solid Tumor

Protocol No.: KN026-203

Version No.: 4.2

Version Date: June 20, 2022

STATEMENT

Alphamab Copyright Reserved. Confidentiality. Its contents shall not be disclosed, published or communicated without the authorization of Alphamab.

STUDY PROCEDURES

OVERALL STUDY DESIGN

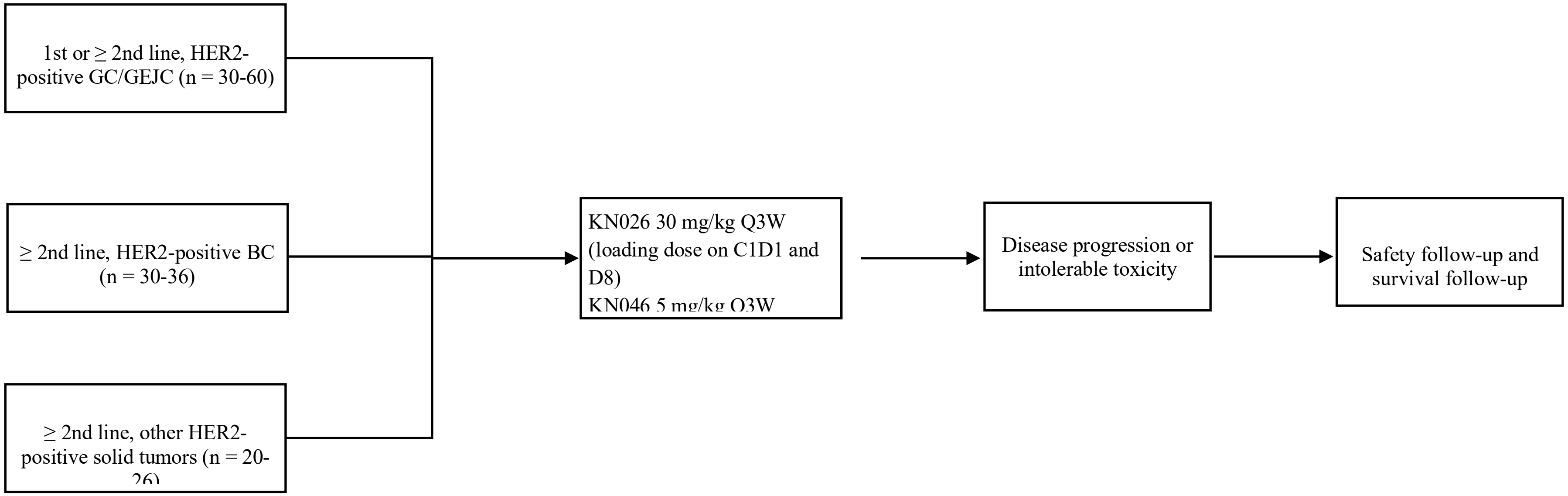

Study Flow Chart

Table 1 Study Flow Chart

**Evaluation**	**Screening Period**	**Treatment Period**							**End of Treatment**	**Safety Follow-up**	**Long-term Safety Follow-up**	**Survival Follow-up**
**Visit**	**Screening**	**Week 1**	**Week 2**	**Week 3**	**Week 4**	**Week 5**	**Week 6**	**Every 3 weeks from Week 7 onwards**	**EOT**	**30-day follow-up visit**	**90-day follow-up visit**	**Every 12 weeks**
**Day**	**(-28 to -1)**	**1**	**1 (± 3 days)**	**1 (± 3 days)**	**1 (± 3 days)**	**1 (± 3 days)**	**1 (± 3 days)**	**1 (± 3 days)**	**Within 7 days after the decision of discontinuation [1]**	**Within 30 days (± 3 days) after last dose**	**Within 90 days (± 7 days) after last dose [2]**	**± 14 days**
**General Procedures**												
Informed consent	X											
Inclusion/exclusion criteria	X											
Demographic data	X											
Medical history/complications	X											
Tumor history	X											
Prior medication history/concomitant medications/concomitant procedures [3]	X	X							X	X	（X）	（X）
Subsequent anti-tumor therapy									X	X	X	X
Survival status												X
**Clinical Examination/Evaluation**												
AEs [4]	X	X							X	X	X	X
Complete physical examination	X								X			
Symptom-directed physical examination		Prior to each study drug administration								X	X	
Height	X											
Weight	X	Prior to study drug administration at each visit							X	X	X	
Vital signs	X	Prior to each study drug administration							X	X	X	

12-Lead ECG	X	Every 6 weeks; 12-lead ECGs performed concurrently with PK blood collections are presented in Table 3							X			
ECOG PS score	X	Prior to each study drug administration							X	X	X	
**Local Laboratory Tests/Evaluations** [5]												
Blood count and differential [6]	X [8]	Prior to study drug administration at each visit							X	（X）[9]		
Coagulation function	X [8]	If clinically indicated										
Serum chemistry [7]	X [8]	Prior to study drug administration at each visit							X	（X）[9]		
Urinalysis [8]	X [8]	Every 12 weeks, or as clinically indicated							X	（X）[9]		
Thyroid function	X	Every 6 weeks, or as clinically indicated							X	（X）[9]		
LVEF	X	Every 9 weeks, or as clinically indicated							X		X	Every 3 months till 1 year
Troponin	X	If clinically indicated										
IL-6		If clinically indicated										
Serum pregnancy test (if applicable)	(X) [10]								(X)			
Urine pregnancy test (if applicable)		(Every 12 weeks)										
Follicle-stimulating hormone (only if confirmation of menopausal status is required)	(X)											
HBV, HCV, HIV [10]	X											
CRP	X											
**Central Laboratory Tests/Evaluations**												
HER2 status (HER2 IHC, HER2 amplification) (tumor tissue) [11]	X											
Biomarkers (tumor tissue) (non-mandatory) [11]	X											
Pharmacokinetics		See Table 3										
ADA		See Table 3										
**Tumor Imaging Assessments**												
Tumor imaging (thoracic/abdominal/pelvic) [12,13]	X	Every 6 weeks (± 7 days); from 48 weeks onwards: every 12 weeks (± 7 days)							(X)	(X)	(X)	(X)
Tumor imaging (brain, if applicable) [12,13]	(X)	(if clinically indicated)										
Bone scan (if applicable) [14]	(X)	(if clinically indicated)										

**Study Drug Administration**												
KN046 administration (Q3W) [15]		X			X			Administration on Day 1/21				
KN026 administration (Q3W) [15]		X	X		X			Administration on Day 1/21				

1 The EOT visit may be performed on the day of the decision to discontinue KN026 and KN046 treatment, and the same EOT test may not be repeated if it is performed within 7 days after the last pre-treatment evaluation;

2 The 30- and 90-day safety follow-up visits will be performed after the end of all study treatments. For subjects who discontinue KN046 treatment first and continue to receive KN026, the 90-day safety follow-up visit is not required if 30 days after the last dose of KN026 are later than 90 days after the last dose of KN046;

3 All concomitant medications/procedures from 28 days prior to dosing to 30 days after the last dose of KN046 and/or KN026 should be recorded. Concomitant medications/procedures after the 30-day safety follow-up period are also to be recorded as part of SAE recording, analysis, and reporting if they are performed for the purpose of treating SAEs related to KN046 and/or KN026;

4 After the subject signs the informed consent form, all adverse events need to be recorded until 30 days after the last dose of KN046 and/or KN026 or initiation of a new anti-tumor therapy, whichever occurs first. SAEs and TRAEs will be collected up to 90 days after the last dose of KN046 or 30 days after the last dose of KN026, whichever occurs later. The investigator is required to monitor adverse events until stable or until the outcome is known, unless the subject is recorded as "lost to follow-up". In the event of treatment-related serious adverse events (SAEs) of KN046 and/or KN026, they need to be recorded whenever they occur and regardless of the time from discontinuation of KN046 and/or KN026, and reported to regulatory authorities in accordance with local regulations; the symptomatic left ventricular systolic dysfunction and asymptomatic left ventricular systolic dysfunction ($\geq$ 10% and < 50% decrease in LVEF from baseline) within 12 months after the last dose of KN026 should be recorded and reported as AEs, and those meeting the criteria for SAEs should be reported to regulatory authorities in accordance with local regulations;

5 The safety laboratory evaluations should be obtained prior to dosing; the pre-dose safety laboratory evaluations on Cycle 1 Day 1 should be performed at screening. The safety laboratory evaluations should include: blood count and differential, coagulation tests (PT/INR/aPTT/TT), serum chemistry, urinalysis, thyroid function (TSH/total T3/free T3/free T4), troponin, HBV/HCV/HIV, and serum/urine pregnancy test. The blood count and differential, coagulation tests (PT/INR/aPTT/TT), serum chemistry, and urinalysis at screening are required to be obtained within 7 days (inclusive) prior to the first dose and used for evaluation of inclusion/exclusion criteria. Refer to Section 7.2.3 for detailed description;

6 Blood count and differential include: red blood cell count (RBC), hematocrit, hemoglobin, mean hemoglobin content (MCH), mean hemoglobin concentration (MCHC), mean corpuscular volume (MCV), platelet count, white blood cell count WBC, neutrophil count and percentage, lymphocyte count and percentage, monocyte count and percentage, basophil count and percentage, eosinophil count and percentage;

7 Serum chemistry tests include: alkaline phosphatase, gamma-glutamyltransferase, alanine aminotransferase (ALT), aspartate aminotransferase (AST), albumin, total protein, total bilirubin, direct bilirubin, indirect bilirubin, creatinine, serum total urea (or urea nitrogen), fasting blood glucose, blood calcium, blood potassium, blood sodium, chloride, phosphate, lactate dehydrogenase (LDH), triglycerides, total cholesterol, and uric acid;

8 Urinalysis includes: pH, specific gravity, glucose, protein, ketones, red blood cells and white blood cells; if urine protein is ≥ 2+ (dipstick), 24-hour urine protein and urine protein/creatinine ratio should be collected;

9 The items with abnormal EOT examination need to be re-determined at the 30-day safety follow-up visit;

10 The test includes hepatitis B five items, HCV antibody and HIV antibody. If HBsAg is positive, HBV DNA should be added; if HCV antibody is positive, HCV RNA should be added;

11 Formalin-fixed archival specimen (biopsy or surgery) tissue (block or slide) containing tumor tissue obtained recently (tumor tissue from a non-irradiated area within 2 years) is required for subjects at screening for HER2 IHC, HER2 amplification, PD-L1 expression, TIL, GEP, and HER2/CDK12 co-amplification analysis (breast cancer cohort), etc. Endoscopic biopsy, core needle biopsy, excisional biopsy, trephine biopsy, and surgical specimens are acceptable. Fine needle aspiration biopsy specimens are not acceptable. Samples may be provided as tumor-containing FFPE blocks or slides of blocks, and the slides should be freshly cut (within 6 months), 4-6 μm thick, and prepared on adhesion slides, preferably providing approximately 15-20 slides; if not possible, 10-15 slides are recommended. If the subject has received prior HER2-targeted therapy and has disease progression, except for the subject with breast cancer, the tumor tissue samples after failure of HER2-targeted therapy are required for confirmation of HER2 status (for HER2 IHC and HER2 expansion). If there are less than 10 slides, the investigator and the sponsor's medical monitor are required to decide whether to enroll the subject, and if there are less than 5 slides, the subject is not allowed to be enrolled (screening failure);

12 Subjects will have chest/abdomen/pelvis (specific tumor types need to include other specific areas) CT scan or MRI (chest CT is mandatory if MRI is used). If CT/MRI imaging is not sufficient to evaluate tumor burden, other established evaluation methods may be added. Contrast-enhanced CT scan is recommended, and contrast-enhanced MRI may be considered if the subject is allergic to contrast. The imaging method needs to be consistent with that used for lesion detection at baseline, and the same imaging device is preferred for subsequent tumor evaluation visits. Baseline tumor imaging assessments should be completed within 28 days prior to the first dose, including scans of at least the chest, abdomen, and pelvis. Subjects who have not undergone tumor imaging assessment of brain within 42 days prior to the first dose are required to undergo brain enhanced CT or MRI at screening. Tumor imaging follow-up visit will be performed every 6 weeks (± 7 days), every 12 weeks (± 7 days) after 48 weeks until disease progression (RECIST 1.1), initiation of new anti-tumor therapy, withdrawal of informed consent, lost to follow-up, death, or end of the study, whichever occurs first (Section 7.2.2.3). Brain imaging follow-up is required only if clinically indicated (e.g., new CNS symptoms or worsening of previous symptoms). The first occurrence of disease response (CR, PR) judged as per RECIST v1.1 criteria requires repeat imaging no earlier than 4 weeks and no later than 8 weeks to confirm disease response per RECIST v1.1 criteria (Section 7.2.2.3);

13 Subjects who discontinue treatment not due to imaging tumor progression (e.g., unacceptable toxicity (Section 6.1.4), clinical deterioration) must continue to be followed for tumor imaging after discontinuation until tumor progression judged as per RECIST v1.1 criteria, initiation of a new anti-tumor therapy, withdrawal of consent, or lost to follow-up, whichever occurs first (Section 7.2.2.3); if a subject experiences PD as judged by RECIST v.1.1 criteria, and continues to have clinical

benefit from continued treatment with KN026 and KN046 as judged by the investigator, the subject will continue to be followed for imaging and oncology evaluation with reference to iRECIST;

14 Subjects who do not have a bone scan within 90 days prior to the first dose will be required to perform bone scan at screening. Subsequently, bone scan should be performed only if clinically indicated. For new lesions suggested by the bone scan, CT/MRI examination is required to determine whether it is metastatic, and oncology evaluation will be performed according to RECIST 1.1 criteria;

15 The duration of each infusion for the first 6 doses of KN046 should be no less than 120 minutes; if no infusion-related adverse events occur, the subsequent infusion time may be adjusted to 90-120 minutes; after the end of each infusion of KN046, the subject should be observed at the study site for at least 2 hours. The first infusion time of KN026 should be 90 minutes (± 15 minutes). If no infusion-related AEs occurred during the first infusion, the subsequent infusion time could be adjusted to 60 minutes (45 to 75 minutes); after the end of each dose of KN026, the subject should be observed at the study site for at least 2 hours. If KN046 and KN026 are administered on the same day, KN026 should be administered first and then KN046 after 2 hours of the end of infusion.

Table 2 Study Flow Chart: Pharmacokinetic and Immunogenicity Sample Collection Form of KN046 and KN026, and ECGs Performed Concurrently with PK Blood Sampling

Week	Time	KN046 PK Sample	KN046 ADA Sample	KN026 PK Sample	KN026 ADA Sample	ECGs Performed Concurrently with PK Blood Sampling
1	0 h (pre-dose of KN046 or KN026) (-60 min)	3.5 mL	3.5 mL	3.5 mL	3.5 mL	X [1,2]
1	End of KN046 or KN026 infusion (+ 30 min)	3.5 mL		3.5 mL		X [1,2]
2	0 h (pre-dose of KN026) (-60 min)			3.5 mL	3.5 mL	
2	End of KN026 infusion (+ 30 minutes)			3.5 mL		
4	0 h (pre-dose of KN046 or KN026) (-60 min)	3.5 mL	3.5 mL	3.5 mL	3.5 mL	
4	End of KN046 or KN026 infusion (+ 30 min)	3.5 mL		3.5 mL		
7	0 h (pre-dose of KN046 or KN026) (-60 min)	3.5 mL	3.5 mL	3.5 mL	3.5 mL	
7	End of KN046 or KN026 infusion (+ 30 min)	3.5 mL		3.5 mL		
10	0 h (pre-dose of KN046 or KN026) (-60 min)	3.5 mL	3.5 mL	3.5 mL	3.5 mL	X [1,2]
16	0 h (pre-dose of KN046 or KN026) (-60 min)	3.5 mL	3.5 mL	3.5 mL	3.5 mL	
22	0 h (pre-dose of KN046 or KN026) (-60 min)	3.5 mL	3.5 mL	3.5 mL	3.5 mL	
31	0 h (pre-dose of KN046 or KN026) (-60 min)	3.5 mL	3.5 mL	3.5 mL	3.5 mL	
40	0 h (pre-dose of KN046 or KN026) (-60 min)	3.5 mL	3.5 mL	3.5 mL	3.5 mL	
49	0 h (pre-dose of KN046 or KN026) (-60 min)	3.5 mL	3.5 mL	3.5 mL	3.5 mL	

58	0 h (pre-dose of KN046 or KN026) (-60 min)	3.5 mL	3.5 mL	3.5 mL	3.5 mL	
67	0 h (pre-dose of KN046 or KN026) (-60 min)	3.5 mL	3.5 mL	3.5 mL	3.5 mL	
EOT		3.5 mL	3.5 mL	3.5 mL	3.5 mL	
30-Day Safety Follow-up Visit		3.5 mL	3.5 mL	3.5 mL	3.5 mL	
90-Day Safety Follow-up Visit		3.5 mL	3.5 mL			
Note: [1] ECGs performed concurrently with PK blood sampling need to be completed within 30-60 minutes after PK blood collection; each ECG will be repeated 3 times, at least 1 minute apart, and recorded<br>[2] Only before and after KN046 administration						

PROTOCOL TABLE OF CONTENTS

CLINICAL STUDY PROTOCOL **1**
STUDY PROCEDURES **2**
1.1 EGFR/HER 16
1.2 HER2-POSITIVE TUMORS AND IMMUNOTHERAPY 16
PROTOCOL TABLE OF CONTENTS **10**
ABBREVIATIONS **14**
1 STUDY BACKGROUND **16**
1.3 KN026 17
1.3.1 Preclinical Pharmacokinetic (PK) Study 17
1.3.2 Preclinical Toxicology Studies 18
1.3.3 Clinical Studies 18
1.4 KN046 20
1.4.1 Preclinical Pharmacokinetic (PK) Study 20
1.4.2 Preclinical Toxicology Studies 20
1.4.3 Clinical Studies 21
2 STUDY RATIONALE **25**
2.1 JUSTIFICATION FOR DOSE 25
2.1.1 Rationale for Dose Selection of KN026 25
2.1.2 Rationale for Dose Selection of KN046 26
2.2 RATIONALE FOR PRIMARY STUDY ENDPOINTS 27
2.3 RATIONALE FOR INDICATION SELECTION 27
2.4 BENEFIT-RISK ASSESSMENT 27
3 STUDY OBJECTIVES AND STUDY ENDPOINTS **29**
3.1 STUDY OBJECTIVES 29
3.1.1 Primary Objective 29
3.1.2 Secondary Objectives 29
3.1.3 Exploratory Objectives 29
3.2 STUDY ENDPOINTS 29
3.2.1 Primary Endpoints 29
3.2.2 Secondary Endpoints 29

3.2.3 Exploratory Endpoints 30
4 STUDY DESIGN 31
4.1 OVERALL STUDY DESIGN 31
4.1.1 End of Study Definition 32
4.1.2 Premature Study Termination 32
4.2 INTERIM ANALYSES 32
5 STUDY POPULATION 33
5.1 INCLUSION CRITERIA 33
5.2 EXCLUSION CRITERIA 35
5.3 CRITERIA FOR WITHDRAWAL OF SUBJECT 37
5.3.1 Discontinuation of Study Treatment 37
5.3.2 Subject Withdrawal 37
6 INVESTIGATIONAL PRODUCTS AND SUBJECT TREATMENT 39
6.1 INVESTIGATIONAL PRODUCTS 39
6.1.1 Dosage and Strength 39
6.1.2 Drug Preparation 39
6.1.3 Dosage and Method of Administration 40
6.1.4 Dose Modification of Investigational Products 41
6.1.5 Treatment Assignment 45
6.1.6 Packaging and Labeling of Drug 45
6.1.7 Storage, Transportation and Shelf Life of Drug 46
6.1.8 Quantity Management of Drug 46
6.1.9 Assessment of Compliance 46
6.1.10 Occupational Safety 46
6.2 CONCOMITANT MEDICATIONS AND NON-DRUG THERAPIES 46
6.2.1 Permitted Medications 46
6.2.2 Prohibited Medications and Procedures 47
6.2.3 Special Precautions 48
7 STUDY PROCEDURES AND EVALUATIONS 54
7.1 STUDY VISIT PLAN 54
7.1.1 Screening Period 54
7.1.2 Treatment Period 55

7.1.3 End of Treatment Visit (EOT) 55
7.1.4 30-Day and 90-Day Safety Visits.....56
7.1.5 Long-term Follow-up 56
7.1.6 End of Study.....57
7.2 STUDY EVALUATIONS 57
7.2.1 Demographics and Other Baseline Characteristics 57
7.2.2 Efficacy Evaluation 58
7.2.3 Safety Evaluation 61
7.2.4 Pharmacokinetic Evaluation.....64
7.2.5 Immunogenicity Evaluation 64
7.2.6 Biomarker Evaluation 64
8 EVALUATION AND RECORDING OF ADVERSE EVENTS 66
8.1 DEFINITION OF ADVERSE EVENT.....66
8.1.1 Adverse Event 66
8.1.2 Serious Adverse Event 66
8.2 DOCUMENTATION AND REPORTING OF ADVERSE EVENTS.....67
8.2.1 Serious Adverse Event Recording and Reporting.....68
8.2.2 Adverse Events of Special Interest.....69
8.2.3 Recording and Reporting of Laboratory Abnormalities and Other Abnormalities.....70
8.2.4 Pregnancy and Intrauterine Exposure 71
8.2.5 Overdose, Medication Error and Abuse 72
9 DATA ANALYSIS AND STATISTICS 73
9.1 STATISTICAL METHODS 73
9.2 SAMPLE SIZE CALCULATION 73
9.3 ANALYSIS SETS.....74
9.3.1 Safety Set.....74
9.3.2 Efficacy Analysis Set 74
9.3.3 PK Analysis Set.....74
9.3.4 Immunogenicity Analysis Set 74
9.4 DEMOGRAPHICS AND OTHER BASELINE CHARACTERISTICS 75
9.5 ANALYSIS OF THE PRIMARY ENDPOINT 75
9.6 ANALYSIS OF THE SECONDARY ENDPOINTS 75

9.6.1 Safety Endpoints 75
9.6.2 Efficacy Endpoints 77
9.6.3 Pharmacokinetic Endpoints 77
9.6.4 Correlation between Pharmacokinetic and Clinical Efficacy Variables 77
9.7 ANALYSES OF THE EXPLORATORY ENDPOINTS 78

ABBREVIATIONS

ADA：	Anti-drug antibody
AE:	Adverse Event
ALT：	Alanine aminotransferase
AIDS：	Acquired immunodeficiency syndrome
aPTT：	Activated partial thromboplastin time
AST：	Aspartate aminotransferase
CBR：	Clinical Benefit Rate
CR：	Complete response
CT：	Computed tomography
DDR：	DNA damage repair
DOR：	Duration of response
EAS：	Efficacy analysis set
ECG：	Electrocardiogram
ECOG：	Eastern Cooperative Oncology Group
HBV：	Hepatitis B virus
HCV：	Hepatitis C virus
HIV：	Human immunodeficiency virus
INR：	International normalized ratio
MRI：	Magnetic resonance imaging
NCI-CTCAE：	National Cancer Institute-Common Terminology Criteria for Adverse Events
NYHA：	New York Heart Association
ORR：	Objective response rate
OS：	Overall survival
PD-L1：	Programmed death ligand-1
PD：	Pharmacodynamics
PET：	Positron emission tomogram
PFS：	Progression-free survival
PK：	Pharmacokinetics
PT：	Prothrombin time
PR：	Partial response
RECIST：	Response Evaluation Criteria in Solid Tumors
SAE:	Serious Adverse Event
SS：	Safety analysis set
SD：	Stable disease
TEAE：	Treatment-Emergent Adverse Event
TESADR：	Treatment-Emergent Serious Adverse Reaction
TIL：	Tumor-infiltrating lymphocyte
TMB：	Tumor mutation burden
TRAE：	Treatment-Related Adverse Event
TRSADR：	Treatment-Related Serious Adverse Reaction

TT： Thrombin time
WOCBP: Women of childbearing potential

1 STUDY BACKGROUND

EGFR/HER

The human epidermal growth factor receptor (EGFR or HER) family consists of four distinct types: HER1 (EGFR or c-erbB-1), HER2 (neu, p185 or cerbB-2), HER3 (c-erbB-3), and HER4(c-erbB-4). These four molecules compile 20 families of receptor tyrosine kinases (RTKs) of type I [1] [2] [3]. The RTK families play an important role in cell proliferation, differentiation, adhesion, survival, and migration. The 20 RTK families share some structural relationships, including extracellular and intracellular segments linked by transmembrane segments, showing high functional and structural identity. In the HER family, similarity of these molecules ranges from 53% (EGFR and HER3) to 64% (EGFR and HER2). The most conserved sequence is located on the tyrosine kinase domain with 59 to 81% identity, and the C-terminal sequence shows a poor similarity between 12 to 30% [4].

HER proteins consist of highly conserved extracellular domains (ECDs) or extracellular binding regions in which ligand fragments interact and result in molecular structural changes that promote receptor dimerization and enhance the kinase activity. The ECD includes domains I, II, III, and IV. Domains I and III interact with corresponding ligands. II and IV are domains common to the HER group and consist of two highly reactive cysteine-rich domains that interact with each other and with the cell membrane. HER3 does not have intramembrane kinase activity whereas HER2 lacks ligand binding sites. However, these receptors can cooperate with each other to form active heterodimers and activate downstream pathways, leading to cell proliferation [5] [6] [7].

HER2 is a transmembrane 185 kDa protein, the gene of which is located on chromosome 17q2145. HER2 protein in mammary epithelial cells is between 20,000 and 50,000 per cell, but cancer cells have more than 2,000,000 receptors per cell [8] [9]. Overexpression of HER2 is also associated with poor prognosis in tumors such as ovarian cancer, gastric cancer, and uterine papillary serous carcinoma [10] [11] [12]. Mutations at codon 655 of the HER2 gene can alter the structure of the receptor transmembrane domain, resulting in an active form that promotes the formation of HER2 homodimers. The mutations of HER2 gene or overexpression of HER2 protein can promote the formation of HER2 homodimers, resulting in continuous uncontrolled growth and division, and apoptosis decrease. Amplification of HER2 results in overexpression of protein, formation of ligand-independent dimers, or formation of dimers with any other receptor in the same family. HER2 has no extracellular domain to which ligands can be ligated, but has high affinity for receptors in the same family, thereby forming heterodimers [1] [13]. Among type I RTKs, HER2 interacts most favorably with other molecules, prolonging ligand/heterodimer ligation, and prolonging activation of the mitogen-activated protein kinase (MAPK) pathway. In addition, HER2 does not interact with any ligand and its conformational arm is readily present in a competent form and linked to any other monomer of the same family [1] [14] [15].

HER2-POSITIVE TUMORS AND IMMUNOTHERAPY

Synergy of HER2-targeted agents with immunotherapy was observed in both HER2-positive breast and gastric cancers. Trastuzumab can promote immunogenic cell death, leading to uptake

of breast carcinoma-associated antigens (BCAAs) by dendritic cells (DCs), presenting and eliciting a CD8 + -specific immune response against breast cancer (BC) cells bearing BCAAs. In the NeoPHOEBE trial, neoadjuvant trastuzumab was combined with the pan-PI3K inhibitor Buparlisib or placebo for 6 weeks followed by the addition of paclitaxel. TIL infiltration was higher in tumor samples after 15 days of treatment compared with TIL score in baseline pretreatment biopsy, which was highly related to pCR [16]. Lapatinib blocks HER2 signaling by stabilizing HER2 protein expression on BC cell membranes, increasing the HER2 protein of FcR on mononuclear immune cells. When HER2 is blocked by lapatinib, the potential effect of IgG1 Fc on ADCC may explain the anti-tumor activity superimposed by trastuzumab. Trastuzumab in combination with lapatinib was superior to lapatinib in PFS (HR: 0.74; 95% CI: 0.58-0.94, p = 0.011) and OS (HR: 0.74; 95% CI: 0.57-0.97, p = 0.026) in the EGF104900 clinical trial [17]. These preclinical and clinical studies have suggested the potential of anti-HER2 therapy in combination with immunotherapy.

PD1 can strongly induce lymphocytes around BC cells. The combination of T-DM1 with checkpoint inhibitors (anti-PD-1 and anti-CTLA-4) has produced a curative effect in animal models, and combinations of PD-1 and CTLA4 inhibitors with anti-HER2+ therapies are currently being developed. In the PANACEA Phase 1b/2 study, pembrolizumab in combination with trastuzumab in patients with HER2+ BC had an objective response rate (ORR) of 15.2% in PD-L1-positive patients, 0% in PD-L1-negative patients, 39% in patients with more than 5% TIL in tumor samples, and 5% in patients with low TIL (< 5%) [18]. Margetuximab is an Fc-optimized mAb that targets HER2, which can enhance Fc ADCC function. The ORR of margetuximab in combination with pembrolizumab was 35.7% in patients with HER2 IHC3+ GC/GEJ and 63.6% in patients with PD-L1+/HER2 IHC3+ GC/GEJ, indicating synergistic efficacy of anti-HER2 therapy with immune checkpoint blockers [19].

Therefore, patients with HER2-positive tumors will be enrolled in this study to explore the efficacy of KN026 combined with KN046.

KN026

KN026 is a novel recombinant humanized bispecific antibody that binds two distinct extracellular domains II (pertuzumab recognition site) and IV (trastuzumab recognition site) of HER2 protein simultaneously. The intact KN026 antibody protein is a heterodimeric antibody molecule consisting of two different IgG1 isoform heavy chains (KN026 heavy chain I, KN026 heavy chain II) and two kappa isoform light chains with identical sequence (common light chain). Both ligand-dependent and ligand-independent signaling of HER2 can be blocked, and thus a better therapeutic effect is expected. The preclinical pharmacodynamic results showed that the anti-tumor effect of KN026 in multiple tumor models at the same dose is not only significantly superior to that of trastuzumab or pertuzumab alone, but also significantly superior to that of trastuzumab combined with pertuzumab in vivo for some tumor types.

Preclinical Pharmacokinetic (PK) Study

After a single intravenous injection of KN026 to cynomolgus monkeys in the range of 1-25 mg/kg, half-life ($T_{1/2}$), mean residence time (MRT) and volume of distribution (Vz) tended to be

prolonged, and clearance (CL) tended to be decreased with the increase of dose. Single doses of KN026 at 1, 5, and 25 mg/kg resulted in half-lives of 23.4, 49.6, and 91.4 h, respectively. There were no significant differences in the main pharmacokinetic parameters between male and female animals after dosing in each dose group. Maximum concentration (C_{max}) and serum exposure ($AUC_{(0-t)}$) increased with the increase of dose in cynomolgus monkeys.

After four consecutive subcutaneous injections of KN026 at 15, 40, and 120 mg/kg, the mean C_{max} and $AUC_{(0-t)}$ increased with the increase of dose in cynomolgus monkeys. There was no significant accumulation at the low, medium and high doses.

Preclinical Toxicology Studies

Single-dose and multiple-dose toxicity studies of KN026 were conducted in cynomolgus monkeys.

The maximum tolerated single dose was greater than 200 mg/kg in cynomolgus monkeys.

When KN026 was administered at 15, 40, and 120 mg/kg once weekly for 5 consecutive doses, no effects were observed on the cardiovascular system, respiratory system and central nervous system of cynomolgus monkeys.

The results of a 6-week study with a 4-week recovery period in cynomolgus monkeys showed that no KN026-related changes were observed in food consumption, local irritation reaction at the administration site, ophthalmological examination, blood pressure, respiratory rate, body temperature, ECG, neurobehavioral examination, clinical pathology parameters, organ weights, gross anatomy and relevant pathology. The no observed adverse effect level (NOAEL) was 120 mg/kg/dose. At this dose, mean C_{max} and $AUC_{(0-t)}$ values of KN026 on Day 22 of the dosing period were 3641.06 μg/mL and 268432.64 hr*μg/mL in males and 3939.65 μg/mL and 325175.98 hr*μg/mL in females, respectively.

Immunogenicity: In the single intravenous dose PK study in cynomolgus monkeys, all 3 dose groups (1 mg/kg, 5 mg/kg, or 25 mg/kg) of animals were positive for anti-drug antibodies (ADAs) 42 days post-dose, with the number of positive animals of 5/6, 3/6, and 4/6. In the multiple intravenous dose study in cynomolgus monkeys (15, 40, and 120 mg/kg weekly), the number of ADA-positive animals was 3/10, 4/10, and 3/10 on Day 29 post-dose, and was 3/4, 3/4, and 2/4 on Day 57 post-dose, respectively.

Hemolysis assay showed that KN026 (25 mg/mL) did not cause hemolytic reactions in red blood cells of rabbits.

In conclusion, the available preclinical toxicology studies support Phase 1 clinical studies in humans. Toxicology study results are detailed in the IB.

Clinical Studies

There are 2 clinical studies of KN026: KN026-CHN-I-001 (first-in-human study of KN026) and KN026-201.

1) The first-in-human study of KN026 (KN026-CHN-I-001) was conducted in Fudan University Shanghai Cancer Center and enrolled patients with HER2-positive advanced breast and gastric cancers who had failed prior standard of care using a traditional "3+3" dose escalation design. As of January 22, 2020, 63 patients with HER2-positive advanced breast cancer had been treated with KN026, including 5 mg/kg QW (n = 3), 10 mg/kg QW (n = 3), 20 mg/kg Q2W (n = 28), and 30 mg/kg Q3W (n = 29). All of these subjects had experienced prior first and later lines of anti-HER2 therapy (e.g., trastuzumab, lapatinib, T-DM1), and had completed DLT evaluation in all four dose groups, which showed the subjects tolerated it well and no DLT events occurred. Subjects received KN026 with a median exposure time of 12 weeks (range: 4 to 60 weeks). Of those, 33 (52.4%) subjects had an exposure time of more than 12 weeks, 8 (12.7%) subjects had more than 24 weeks, 2 (3.2%) subjects had more than 36 weeks, and 1 (1.6%) subject had more than 60 weeks. All 62 subjects had ≥ 1 postbaseline tumor assessment: for the 20 mg/kg Q2W group, 1 subject had complete disappearance of target lesions at two postbaseline evaluations, and the overall response was assessed as confirmed partial response (PR), 5 subjects were assessed as confirmed PRs, and 3 subjects were assessed as unconfirmed PRs; for the 30 mg/kg Q3W group, 2 subjects were assessed as confirmed PRs and 3 subjects were assessed as unconfirmed PRs. A total of 23 subjects had stable disease (SD), most of whom had varying degrees of shrinkage of target lesion. The overall objective response rate was 27.5%, and the disease control rate was 72.5%. At recommended Phase II doses (20 mg/kg Q2W and 30 mg/kg Q3W), the objective response rate was 31.1%, and the disease control rate was 75.6%. Forty-eight (76.2%) subjects had adverse events related to KN026 treatment, in which AEs with an incidence of ≥5% were: fever (n = 15, 23.8%), diarrhea (n = 12, 19.0%), aspartate aminotransferase increased (n = 9, 14.3%), hypokalemia (n = 6, 9.5%), infusion-related reaction (n = 6, 9.5%), ECG T wave abnormal (n = 6, 9.5%), white blood cell count decreased (n = 5, 7.9%), alanine aminotransferase increased (n = 5, 7.9%), blood creatinine increased (n = 5, 7.9%), proteinuria (n = 4, 6.3%), asthenia (n = 4, 6.3%), hypertriglyceridemia (n = 4, 6.3%), hyperglycemia (n = 4, 6.3%), rash (n = 4, 6.3%), anemia (n = 4, 6.3%), first degree atrioventricular block (n = 4, 6.3%), and neutrophil count decreased (n = 4, 6.3%). There were 4 ≥ Grade 3 treatment-related adverse events: in the 20 mg/kg Q2W group, 1 subject had a Grade 3 transaminase increased without bilirubin increased (leading to hospitalization; recovered after symptomatic treatment) and 1 subject had a Grade 3 ventricular arrhythmia (leading to hospitalization; discontinuation of KN026; recovered at the 30-day safety follow-up visit after symptomatic treatment); in the 30 mg/kg Q3W group, 1 subject had a Grade 3 infusion-related reaction (fever and chills on the first dose; recovered after treatment, but was hospitalized the next day due to fever) and 1 subject had a Grade 3 hypertension (the patient had a history of hypertension; the blood pressure transiently increased to Grade 3 2 hours after the first dose, and recovered on the same day). No KN026 treatment-related AEs led to death. No decrease in LVEF of ≥ 15% or below the lower limit of normal was observed. There were 5 KN026 treatment-related SAEs, including 1 Grade 3 transaminase increased, 1 Grade 3 ventricular arrhythmia, 1 Grade 2 interstitial pneumonia, 1 Grade 3 infusion-related reaction and 1 cardiac myxoma (the investigator could not determine the relationship with KN026, and the sponsor considered it unrelated to KN026, but the SAE was "related" according to the statistical analysis plan). The safety events were similar to those previously reported for anti-HER2-targeted agents, and no new safety signals occurred. As of September 2019, the pharmacokinetic data after the first dose have been available for 12 patients in the

Phase 1 KN026 study in China. In the dose range of 5-30 mg/kg, C_{max} and $AUC_{0-\infty}$ of KN026 increased with the increase of dose, showing a linear pharmacokinetic profile. Mean predose KN026 plasma concentrations in the 10 mg/kg QW group had reached steady-state trough levels (79 µg/mL) at the approved dose of trastuzumab.

2) Cohort 1 of KN026-201 included patients with HER2-positive metastatic breast cancer who had not received prior systemic therapy. As of January 18, 2021, 21 patients had been treated with KN026 in combination with docetaxel. The median treatment duration was 10 weeks (range: 3 to 51 weeks). Eleven subjects (45.8%) had a treatment duration of more than 12 weeks, 4 subjects (16.7%) had a treatment duration of more than 24 weeks, 2 subjects (8.2%) had a treatment duration of more than 36 weeks, and 1 subject (4.2%) had a treatment duration of more than 48 weeks. The events occurring in ≥ 10% of patients included neutrophil count decreased (n = 8, 22.9%), white blood cell count decreased (n = 8, 22.9%), diarrhea (n = 7, 20.0%), aspartate aminotransferase increased (n = 6, 17.1%), alanine aminotransferase increased (n = 5, 14.3%), and infusion-related reaction (n = 5, 14.3%). The combination of KN026 and taxane was demonstrated to be safe and tolerable.

KN046

KN046 is a novel recombinant humanized bispecific antibody that binds both PD-L1 and CTLA-4, thereby blocking the binding of PD-L1 to PD-1 and CTLA-4 to CD80/CD86. KN046 is expressed and produced by CHO cells and the wild-type IgG1 Fc in the molecule maintains ADCC and CDC functions. CTLA-4 inhibitors act on naive T cells in secondary lymphoid organs and also mediate the depletion of Tregs, resulting in anti-tumor effects. PD-1/PD-L1 inhibitors can relieve inhibitory conduction pathways in the tumor microenvironment, thereby activating tumor-infiltrating CTLs. The binding of KN046 molecule to PD-L1 is stronger than that of CTLA-4, and has a stronger inhibitory effect on tumors with high expression of PD-L1, so KN046 can strongly activate the immune system in the tumor microenvironment. In clinical toxicology studies in cynomolgus monkeys, KN046 also showed good tolerability.

Preclinical Pharmacokinetic (PK) Study

A preclinical PK study of KN046 was conducted in cynomolgus monkeys. After intravenous (IV) administration of 1-100 mg/kg KN046 in cynomolgus monkeys, C_{max} and AUC increased proportionally with the dose, with $T_{1/2}$ of 51-88.3 h and CL of 0.701-0.747 mL/hr/kg; the mean accumulation rate after multiple doses was less than 2.0 (0.72-1.38).

The PK information for KN046 is detailed in the Investigator's Brochure (IB).

Preclinical Toxicology Studies

Single-dose and multiple-dose toxicity studies of KN046 were conducted in non-human primates.

Single-dose toxicity: Single-dose toxicity was also observed in a 4-week multiple-dose toxicity study in cynomolgus monkeys. Its results showed the MTD of a single dose of KN046 was approximately greater than 100 mg/kg.

Multiple-dose toxicity: In the 4-week multiple-dose toxicology study in cynomolgus monkeys, the investigational product was administered intravenously at 0 mg/kg (control group), 10 mg/kg, 30 mg/kg, and 100 mg/kg weekly for a total of 5 doses, with a 6-week recovery period. In the 30 mg/kg group, 1 male monkey died during the 5th dosing period. Pathological observations were performed on Day 18, 20, and/or 29, presumably due to immunogenic challenge with KN046, which had the highest ADA titer on Day 29, rather than a direct effect of KN046. KN046-related major changes were noted in the ≥ 30 mg/kg group, including enlarged groin nodes and decreased body weight and food consumption in 1 animal (at 100 mg/kg); pathological changes were mainly indicative of inflammatory responses, including slightly increased large unstained cell counts, mild to moderate increases in fibrinogen concentrations, and slight to mild increases in globulin concentrations; histopathologically noted adverse reactions included multiorgan vascular inflammation, mesangioproliferative glomerulonephropathy and/or concomitant tubular degeneration/necrosis/regeneration, hypertrophy/hyperplasia of Kupffer cells, neutrophil/mononuclear cell infiltration in liver sinusoids and/or portal areas, and cardiomyocyte degeneration/necrosis (at ≥ 30 mg/kg). The no adverse effect level (NOAEL) for KN046 was 10 mg/kg.

Immunogenicity: In a single intravenous dose PK study in cynomolgus monkeys, all 3 groups of animals (1.0 mg/kg, 3.0 mg/kg, or 10.0 mg/kg) were positive for anti-drug antibodies (ADA) at 42 days post-dose, with the number of ADA-positive animals of 17/18. Only 1 animal in the 3.0 mg/kg group was ADA-negative. In the multiple IV dose study in cynomolgus monkeys (10, 30, and 100 mg/kg weekly), 4/10 animals were ADA-positive on Day 29 and 4/10, 0/10, and 1/10 animals were ADA-positive on Day 43 in three dose groups, respectively.

Hemolysis assay showed that KN046 (26.3 mg/mL) did not cause hemolytic reactions in red blood cells of rabbits.

In conclusion, the available preclinical toxicology studies support Phase 1 clinical studies in humans. Toxicology study results are detailed in the IB.

Clinical Studies

There are four KN046 studies: KN046-AUS-001 (first-in-human study of KN046), KN046-CHN-001 (first-in-China study of KN046), KN046-202, and KN046-IST-02.

1) The first-in-human study of KN046 (KN046-AUS-001) was conducted at 3 study sites in Australia using a traditional "3 + 3" dose escalation design. As of January 20, 2020, a total of 54 subjects were enrolled in this study, including 0.3 mg/kg Q2W (n = 1), 1 mg/kg Q2W (n = 3), 3 mg/kg Q2W (n = 17), 5 mg/kg Q2W (n = 30), and 10 mg/kg Q2W (n = 3). Subjects received KN046 for a median exposure time of 11 weeks (range: 2 to 67 weeks). Of these, 45 (51.1%) subjects had an exposure time of more than 12 weeks, 20 (22.7%) subjects had more than 24 weeks, 12 (13.6%) subjects had more than 36 weeks, and 2 (2.3%) subjects had more than 48 weeks. KN046 was generally well tolerated by the subjects. Four DLT events were observed in 3 subjects, including 1 subject in the 5 mg/kg group who experienced Grade 3 treatment-related hepatic function abnormal without increased bilirubin, 1 subject in the 10 mg/kg group who experienced Grade 3 pruritic erythematous rash, and 1 subject in the 10

mg/kg group who experienced Grade 3 aspartate transferase increased and Grade 3 arthritis. These events were resolved within 3 weeks. Therefore, the maximum tolerated dose was considered to be 5 mg/kg Q2W.

Treatment-related adverse events (TRAEs) of KN046 were reported in 41 (75.9%) subjects and KN046 TRAEs of Grade 3 or above were reported in 20 (37.0%) subjects. Treatment-related SAEs of KN046 were reported in 14 (25.9%) subjects and immune-related adverse events (irAEs) were reported in 26 (48.1%) subjects, of which 13 (24.1%) subjects experienced irAEs of Grade 3 or above. The most common TRAEs included arthralgia, fatigue, and infusion-related reactions. The most common irAEs included arthralgia (musculoskeletal and connective tissue disorders) and pruritus (skin and subcutaneous tissue disorders). No dose-related relationship was found for TRAEs and irAEs, and no increase in the number and severity of TRAEs or irAEs was found with escalation to the RP2D level as compared with the lower dose level.

As of January 20, 2020, 35 subjects received at least one postbaseline response evaluation and were included in the efficacy analysis. The results showed 1 confirmed CR, 2 confirmed PR, 4 unconfirmed PR, 15 SD, and 20 PD. The objective response rate (CR + PR) was 16.7% and the disease control rate (CR + PR + SD) was 52.4%. As of the cut-off date, 8 evaluable subjects were still receiving KN046 treatment.

After administration of 3 mg/kg (n = 3), KN046 showed linear elimination in humans with half-lives ranging from 4.4 to 7.3 days (mean 6.2 days) and trough concentrations ranging from 4.6 to 6.8 μg/L (mean 5.4 μg/L) on Day 15. After administration of 5 mg/kg (n = 6), KN046 showed linear elimination in humans with half-lives ranging from 5.2 to 11.3 days (mean 8.0 days) and trough concentrations ranging from 5.9 to 16.2 μg/L (mean 12.8 μg/L) on Day 15

2) KN046-CHN-001 was a Phase 1a/1b study in Chinese subjects conducted at the Sun Yat-sen University Cancer Center. As of January 20, 2020, 88 subjects were enrolled in the study, including 1 mg/kg Q2W (n = 1), 3 mg/kg Q2W (n = 30), 5 mg/kg Q2W (n = 45), 5 mg/kg Q3W (n = 6), and fixed dose of 300 mg Q3W (n = 6). Subjects received KN046 for a median exposure time of 12 weeks (range: 2 to 54 weeks). Of these, 45 (51.1%) subjects had an exposure time of more than 12 weeks, 20 (22.7%) subjects had more than 24 weeks, 12 (13.6%) subjects had more than 36 weeks, and 2 (2.3%) subjects had more than 48 weeks. Thirty-three subjects are still receiving study treatment. A total of 55 subjects have discontinued study treatment, including: 1) 36 subjects due to disease progression; 2) 5 subjects due to AEs; 3) 3 subjects due to death; 4) 2 subjects due to lost to follow-up; and 5) 9 subjects due to other reasons. The subjects enrolled in the Phase 1 study in China were mainly patients with advanced solid tumors. There were no DLT events during the dose escalation phase and the maximum tolerated dose was not reached at 5 mg/kg Q2W, therefore 5 mg/kg Q2W was considered as the RP2D. As of the cut-off date, 74 (84.1%) of 88 subjects experienced TRAEs, 9 (10.2%) experienced TRAEs of Grade 3 or above, and 4 (4.5%) experienced treatment-related SAEs. Forty (45.5%) subjects experienced irAEs, of which 4 (4.5%) were Grade 3. Similar to the results of the KN046-AUS-001 study, TRAEs and irAEs showed no dose dependence during the dose escalation phase. The most common TRAEs

included rash (34.1%), pruritus (31.8%), alanine aminotransferase increased (19.3%), infusion-related reaction (17.0%), asthenia (17.0%), aspartate aminotransferase increased (13.6%), pyrexia (12.5%), and arthralgia (10.2%). The most common irAEs included skin and subcutaneous tissue disorders and general disorders and administration site conditions. As of January 20, 2020, 75 subjects were evaluable for response, including 8 confirmed PR and 27 SD. Twenty-nine evaluable subjects are still receiving KN046. The objective response rate was 12.0% and the disease control rate was 48.0%.

As of June 24, 2019, *in vitro* releasing data of IL-2 were obtained from 16 subjects, and a PK/PD model was constructed using R, with the model equation as:

$$E(C) = E0 - \frac{Imax \cdot (E0 - 1) \times C}{IC50 + C}$$

The model showed adequate goodness-of-fit plots. Due to the high residual standard error (RSE) of 115.6% for the model parameter IC_{50}, the range of 95% CI (180-457 ng/mL) of the interval estimates was too large for a precise estimation of IC_{50}. Therefore, the corresponding Cmin targets were estimated from the point estimate of IC_{50} (138 ng/mL) and the upper limit of 95% CI (457 ng/mL), respectively. The corresponding Cmin targets were 2629 ng/mL and 8683 ng/mL, respectively. Based on the preliminary exposure-response relationship analysis of the efficacy data, 5 mg/kg Q2W was recommended for further exploration of KN046 monotherapy, and 5 mg/kg Q3W was recommended for further exploration of KN046 co-medication.

3) KN046-202 included patients with advanced NSCLC without EGFR-sensitive mutations who had not received prior systemic therapy. As of January 07, 2021, 36 patients with squamous cell carcinoma had been treated with KN046 in combination with paclitaxel + carboplatin. The median treatment duration was 21 weeks (range: 1.6 to 53.9 weeks). Twenty-eight subjects (77.8%) had a treatment duration of more than 12 weeks, 14 subjects (38.9%) had a treatment duration of more than 24 weeks, 8 subjects (22.2%) had a treatment duration of more than 36 weeks, and 2 subjects (5.6%) had a treatment duration of more than 48 weeks. The AEs with an incidence ≥10% included infusion-related reactions (52.9%), pruritus (40.2%), rash (34.5%), alanine aminotransferase increased (24.1%), aspartate aminotransferase increased (24.1%), pyrexia (17.2%), decreased appetite (13.8%), and diarrhea (10.3%). The combination of KN046 and paclitaxel was demonstrated to be safe and tolerated.

4) KN046-IST-02 was an investigator-initiated study in subjects with HER2-positive or low- and moderate-expressing solid tumors. The dose escalation phase designed three dose groups (KN026 20 mg/kg Q2W + KN046 3 mg/kg Q2W, KN026 20 mg/kg Q2W, C1D8 loading + KN046 5 mg/kg Q3W, and KN026 30 mg/kg Q3W + KN046 5 mg/kg Q3W), with 3 to 6 subjects planned to be included in each dose group for DLT observation. As of January 12, 2021, the study enrolled a total of 32 subjects with HER2-positive gastric cancer (7 for the first line treatment and 15 for the second line and above treatment) or other gastrointestinal tumors (10 subjects), including 20 subjects receiving KN026 20 mg/kg Q2W in combination with KN046 3 mg/kg Q2W, 2 subjects receiving KN026 20 mg/kg Q2W (C1D8 loading dose) in combination with KN046 5 mg/kg Q3W, and 10 subjects receiving KN026 20 mg/kg Q3W

(C1D8 loading dose) in combination with KN046 5 mg/kg Q3W. It was generally well tolerated in these 3 dose groups with no DLT events observed. Twenty-eight subjects had KN026-related AEs, including 5 subjects with ≥ Grade 3 KN026-related AEs; 27 subjects had KN046 treatment-related AEs, including 6 subjects with ≥ Grade 3 KN046-related AEs. The most common (incidence ≥ 15%) KN026- and KN046-related AEs included anemia (31%), diarrhea (28%), blood bilirubin increased (25%), AST increased (22%), platelet count increased (19%), white blood cell count decreased (19%), and ALT increased (16%). All were common AEs in previous clinical studies of KN026 and KN046, and no new AEs occurred with the combination of the two drugs. In the 7 evaluable subjects with gastric cancer receiving the first line treatment, the objective response rate and disease control rate were both 86% (6/7, 95% CI: 42% -100%); in the 11 evaluable subjects with gastric cancer receiving the second or above line treatment, the objective response rate was 46% (5/11, 95% CI: 42% -100%) and the disease control rate was 91% (10/11, 95% CI: 59% -100%); in the 7 evaluable subjects with other gastrointestinal tumors receiving the second or above line treatment, the objective response rate was 43% (3/7, 95% CI: 10% to 82%) and the disease control rate was 100% (7/7, 95% CI: 59% -100%).

2 STUDY RATIONALE

JUSTIFICATION FOR DOSE

Rationale for Dose Selection of KN026

The pre-specified dose of KN026 in this study is 30 mg/kg Q3W, which was mainly selected based on the safety and preliminary efficacy data from KN026-CHN-I-001 study (first-in-human study of KN026). The safety evaluation at 30 mg/kg dose level has been completed in KN026-CHN-I-001. KN026 was generally well tolerated by the subjects without DLT events. There were 3 subjects with confirmed PR and 5 subjects with unconfirmed PR, and the subjects with SD had varying degrees of reductions in the target lesions. The dose selection also referred to the approved doses of the same class of drugs trastuzumab and pertuzumab, as well as the clinical study data and recommended Phase 2 dose of the investigational drug ZW25 with the same action mechanism.

The action mechanism of KN026 was similar to trastuzumab in combination with pertuzumab. As described in the package inserts of trastuzumab [10], trastuzumab is used for metastatic breast cancer with an initial loading dose of 4 mg/kg and a weekly maintenance dose of 2 mg/kg, resulting in a steady-state trough level of 79 μg/mL; trastuzumab is used for adjuvant treatment of breast cancer with an initial loading dose of 4 mg/kg and a maintenance dose of 6 mg/kg every 3 weeks, resulting in a steady-state trough level of 63 μg/mL. As described in the package inserts for pertuzumab [10], pertuzumab is used for adjuvant treatment of breast cancer with an initial loading dose of 840 mg and a maintenance dose of 420 mg every 3 weeks, resulting in a steady-state trough level of 60-70 μg/mL. Therefore, we expected that the mean trough concentration levels of the effective dose of KN026 would also be within this range. In the Phase 1 dose escalation study of KN026, the dose levels with trough concentrations within the range of 60-80 μg/mL will be selected as the dose in Phase 2 clinical study. At present, we have obtained PK data of KN026 5 mg/kg and 10 mg/kg QW dose levels up to March 10, 2019, which showed that the pre-dose plasma concentration of KN026 in the 10 mg/kg QW dose group had reached the steady-state trough levels at the approved trastuzumab dose. The dose intensities of 30 mg/kg Q3W and 10 mg/kg QW were comparable.

Besides, the translational medicine researches of KN026 suggested that the effective concentration of KN026 was 20 μg/mL, and 60-80 μg/mL was the optimal tumor killing concentration range. A population PK model was constructed based on human PK data, and 1000 simulations of the target population were performed. The recommended Phase 2 dose was defined as that 90% or more of the simulated population reached the target concentration range of 60-80 μg/mL. Based on the clinical safety data, 20 mg/kg Q2W or 30 mg/kg Q3W was used as the recommended Phase 2 dose of KN026, and steady state would be reached at Week 8 (Q2W) or Week 9 (Q3W) after multiple doses (Figure 1 and 2).

A human PK-tumor growth model was constructed based on human pharmacokinetic and human efficacy data to analyze the correlation between PK and anti-tumor activity in humans. The analysis suggested that rapid maximal effective concentrations were associated with the optimal

antitumor activity, so a loading dose was designed. The steady-state concentrations would be reached in the first cycle of administration after the loading dose in Weeks 1 and 8 at 20 mg/kg Q2W (Figure 3), which was expected to further improve the efficacy.

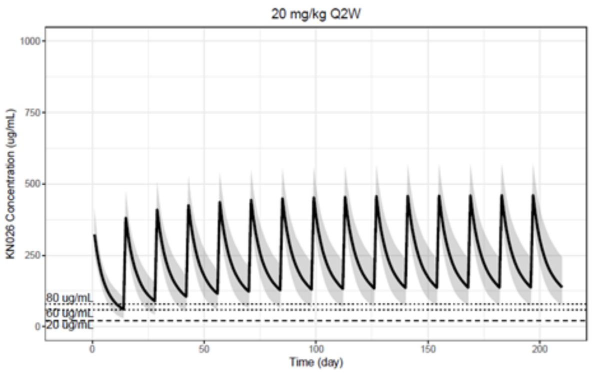

Figure 1

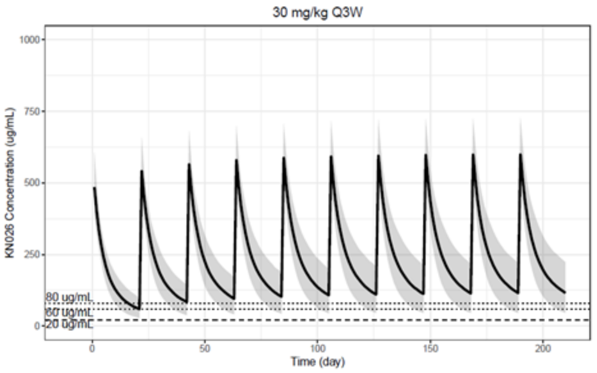

Figure 2

In the investigator-initiated study KN046-IST-02, the safety and tolerability of KN026 in combination with KN046 were investigated in patients with advanced HER2-positive or HER2-expressing gastrointestinal tumors after failure of standard of care. The study results showed that KN026 30 mg/kg Q3W (loading dose on C1D1 and D8) + KN046 5 mg/kg Q3W was a safe and tolerable dose.

Rationale for Dose Selection of KN046

The pre-specified dose of KN046 for this study is 5 mg/kg Q3W. The rationale for dose selection was mainly based on the safety and preliminary efficacy data from the KN046-AUS-001 study (first-in-human study of KN046) and KN046-CHN-001 study (first-in-China study of KN046). The safety evaluation at 5 mg/kg Q2W has been completed in KN046-AUS-001 and the safety evaluations at 5 mg/kg Q2W and Q3W have been completed in KN046-CHN-001. KN046 was generally well tolerated by the subjects. Based on the *ex vivo* release data of IL-2 from tumor patients in KN046-CHN-001 clinical study and corresponding KN046 drug concentration data, a PK (pharmacokinetic)/PD (pharmacodynamic) model was constructed to explore the correlation between drug concentration and *ex vivo* release of IL-2.

As of June 24, 2019, *in vitro* releasing data of IL-2 were obtained from 16 subjects, and a PK/PD model was constructed using R, with the model equation as:

$$E(C) = E0 - \frac{Imax \cdot (E0-1) \times C}{IC50 + C}$$

The corresponding Ctrough_targets (IC95) were estimated from the point estimate of IC_{50} (138 ng/mL) and the upper limit of 95% CI (457 ng/mL), respectively. The corresponding Ctrough_targets were 2,629 ng/mL and 8,683 ng/mL, respectively. Ctrough_target was placed into the 3 mg/kg Q2W, 5 mg/kg Q2W and the fitted 5 mg/kg Q3W concentration-time curve. Since more than 75% of the dosing interval of 5 mg/kg Q3W per cycle was above Ctrough_target, 5 mg/kg Q3W may be suitable for KN046 combination therapy.

KN046-IST-02 study has demonstrated that KN026 20 mg/kg Q2W + KN046 3 mg/kg Q2W is a safe and effective dose. Based on the data from Phase 1 studies of KN026 and KN046 and PK/PD study results, and considering that dosing every three weeks is more convenient in clinical practice, the dose of KN026 30 mg/kg Q3W + KN046 5 mg/kg Q3W is selected for this study. The safety data from the dose group KN026 30 mg/kg Q3W + KN046 5 mg/kg Q3W will be provided as further support before the subjects are enrolled in this study.

RATIONALE FOR PRIMARY STUDY ENDPOINTS

The primary endpoints of this study are ORR and DOR as assessed by the investigator per RECIST 1.1. For early exploratory efficacy study, objective response and duration of response are acceptable surrogate endpoints for measuring clinical benefits and can reflect the drug's anti-tumor activity, especially when persistent anti-tumor response can be observed.

RATIONALE FOR INDICATION SELECTION

The study will be conducted in designated tumor types, including HER2-positive gastric/gastroesophageal junction (GC/GEJ) cancer, HER2-positive breast cancer, HER2-positive non-small cell lung cancer, and other HER2-positive solid tumors. The rationale for selection of HER2-positive tumors is provided in Section 1.2.

BENEFIT-RISK ASSESSMENT

The occurrence of AEs will be closely observed in the clinical study, including vital signs, ECG, laboratory blood and urine tests. Based on the preclinical studies of KN026 and clinical results of currently marketed anti-HER2 monoclonal antibody drugs with the same target, the most common adverse reactions included pyrexia, nausea, vomiting, infusion-related reactions, diarrhea, infection, cough aggravated, headache, asthenia, dyspnea, rash, neutropenia, anemia, and myalgia. Most were mild to moderate in severity. Adverse reactions requiring interruption or discontinuation of anti-HER2 monoclonal antibody therapy included congestive heart failure, significant decrease in left ventricular function, severe infusion reactions, and pulmonary toxicity. Close monitoring and specific targeted treatment measures are necessary. Based on relevant ESMO and NCCN guidelines and the principles of toxicity management in expert consensus, this study will reduce the potential risks of subjects with measures such as exclusion criteria, safety monitoring, initial dose design, treatment interruption provisions, discontinuation criteria, and management of toxicity related to anti-HER2 monoclonal antibody drugs (Sections 6.1.4, 6.2.3.2 and 6.2.3.3). The management of potential infusion reactions and hypersensitivity reactions during use of KN026 as a biological product is described in Section 6.2.3.1.

Based on preclinical studies of KN046 and clinical results of marked anti-PD-L1 and anti-CTLA-4 monoclonal antibody drugs with the same target, the most common adverse reactions included tiredness, infusion reaction, diarrhea, arthralgia, rash, nausea, pruritus, and headache, most of which were mild to moderate in severity. Autoimmune-related toxicities may occur during drug treatment with anti-PD-L1 and anti-CTLA-4 monoclonal antibodies, therefore close monitoring and special therapeutic measures are required. These drug-related AEs included rash and pruritus

in the skin system, diarrhea and colitis in the gastrointestinal system, hypophysitis, hepatitis, endocrine disorders, pneumonia, and renal insufficiency. In this study, principles for the management of immune-related toxicities will be developed with reference to relevant ESMO and NCCN guidelines. Measures such as exclusion criteria, safety monitoring, initial dose design, treatment interruption, discontinuation criteria, and management of immune-related toxicities will be taken to reduce the potential risks of subjects (Section 6.1.4, Appendix 4). The management of potential infusion reactions and hypersensitivity reactions during use of KN046 as a biological product is described in Section 6.2.3.1. As ADCC function is retained in the structure of KN046, the management of potential tumor lysis syndrome is described in Section 6.2.3.4.

The study will be conducted in compliance with the protocol, GCP, the Declaration of Helsinki and other relevant regulations.

3 STUDY OBJECTIVES AND STUDY ENDPOINTS

STUDY OBJECTIVES

Primary Objective

- To evaluate the anti-tumor activity of KN026 in combination with KN046 in patients with HER2-positive solid tumors;

Secondary Objectives

- To evaluate the safety and tolerability of KN026 in combination with KN046;
- To evaluate the effect of HER2 amplification on the efficacy of KN026 in combination with KN046 (as supported by data);
- To evaluate the effect of HER2 expression levels (IHC1+ vs. IHC2+ vs. IHC3+) on the efficacy of KN026 in combination with KN046;
- To evaluate the effect of KN026 and KN046 drug exposure levels on anti-tumor activity;
- To evaluate immunogenicity of KN026;
- To evaluate immunogenicity of KN046.

Exploratory Objectives

- To evaluate the effect of PD-L1 expression, TIL, and TMB/GEP on the efficacy of KN026 in combination with KN046; to evaluate the correlation between HER2/CDK12 co-amplification and clinical efficacy variables in the breast cancer cohort.

STUDY ENDPOINTS

Primary Endpoints

- Objective response rate (ORR) and duration of response (DOR) as judged by the investigator per RECIST 1.1 criteria

Secondary Endpoints

- Progression free survival (PFS), progression-free survival rate (PFSR) at 6 and 12 months, clinical benefit rate (CBR; defined as CR, PR, or SD ≥ 24 weeks) as judged by the investigator according to RECIST 1.1 criteria;
- Overall survival (OS), overall survival rate (OS rate) at 6 and 12 months;
- Frequency and severity of adverse events (AEs) (NCI CTCAE 5.0); changes in vital signs, physical examinations, electrocardiograms, and safety laboratory measures;
- Correlation between biomarkers (HER2 IHC, HER2 amplification) and clinical efficacy variables (ORR, CBR, PFSR, etc.);

- Correlation of KN026 and KN046 exposure levels ($C_{max,ss}$, AUC_{ss}, $C_{trough,ss}$, $C_{avg,ss}$, etc.) with efficacy variables (ORR, DOR, CBR, etc.) and safety variables (QTc and LVEF);
- Incidence and titer of anti-KN026 antibody (ADA); incidence of KN026 neutralizing anti-drug antibody (NADA) in subjects with high ADA titer;
- Incidence and titer of anti-KN046 antibody (ADA); incidence of KN046 neutralizing anti-drug antibody (ADA) in subjects with high ADA titer.

Exploratory Endpoints

- The correlation between biomarkers (PD-L1 expression, TIL, and TMB) and clinical efficacy variables (ORR, CBR, PFSR, etc.); the correlation between HER2/CDK12 co-amplification and clinical efficacy variables in the breast cancer cohort.

4 STUDY DESIGN

OVERALL STUDY DESIGN

This study is an open-label, Phase 2, multicenter clinical study to evaluate the efficacy and safety of KN026 in combination with KN046 in subjects with HER2-positive solid tumors.

The study will include:

Three different cohorts of HER2-positive solid tumors treated with KN026 30 mg/kg Q3W (loading dose on C1D1 and D8) in combination with KN046 5 mg/kg Q3W:

- HER2-positive gastric/gastroesophageal junction cancer (GC/GEJ), n = 30 to 60;
- HER2-positive breast cancer (BC), n = 30 to 36;
- Other HER2-positive solid tumors, n = 20 to 26.

HER2-positive is defined as follows:

- HER2 IHC3+ (ASCO/CAP 2018; Wolff et al, 2018); **or**
- HER2 gene amplification (ISH+: HER2/CEP17 ratio > 2 or HER2 gene copy number > 6; or NGS: HER2 gene copy number > 6).

All subjects will be required to submit slides for central laboratory review of HER2 status at screening. Subjects who have received HER2-targeted therapies such as trastuzumab should provide tumor tissue samples collected after failure of HER2-targeted therapy for determination of HER2-positive status.

The study is divided into a screening period (Day -28 to -1), a treatment period, the end-of-treatment (EOT) follow-up visit, a 30-day safety follow-up visit, a 90-day safety follow-up visit, and a survival follow-up visit.

Subjects will undergo tumor imaging assessments at screening and every 6 weeks (± 7 days) after the dose of study drug. After 48 weeks, the frequency of imaging assessments will be adjusted to every 12 weeks (± 7 days) until disease progression per RECIST 1.1, initiation of a new anti-tumor therapy, or subject withdrawal of consent, whichever occurs first. When an objective response defined in RECIST 1.1 occurs, confirmation by imaging should be performed no earlier than 4 weeks and no later than 8 weeks.

Each subject will receive KN026 in combination with KN046 per protocol until radiographic progression per RECIST 1.1, intolerable toxicity, or withdrawal of consent, whichever occurs first. After the investigator first judges the subject condition as PD per RECIST v1.1 criteria, if the subject is clinically stable and does not experience intolerable toxicity during the treatment, the subject will be allowed to continue treatment with KN026 + KN046 until PD is confirmed. Clinical stability is defined as: stable ECOG PS score, no unacceptable toxicity associated with treatment of KN026 and/or KN046, no rapid PD requiring other salvage therapy, and no acute conditions requiring urgent medical intervention due to PD (e.g., central nervous system metastases, dyspnea due to tumor compression of the airway, spinal cord compression, etc.). The

clinical, oncology, and laboratory evaluation flow of each study period is detailed in Table 1 and 2 as well as Section 7.

End of Study Definition

The end of the study is defined as 1 year after the last dose of all subjects; the completion of the last study-related telephone contact or visit, withdrawal from the study or lost to follow-up (i.e., the investigator is unable to contact the subject) of the last subject; death or premature withdrawal from the study of all subjects; or the sponsor assesses that the study has met the study expectations (e.g., completion of the primary endpoint assessments), whichever occurs first.

Premature Study Termination

The study may be prematurely discontinued if:

- New information suggests an unacceptable risk-benefit ratio for the investigational product;
- The sponsor decides to terminate the study and/or a cohort based on the consideration that it is no longer appropriate to continue the study from the medical and ethical perspective;
- It is not possible to complete the study within an acceptable time period due to poor enrollment of subjects. The sponsor may decide to terminate the study of a cohort if the enrollment of a cohort is poor and the likelihood of study completed within an acceptable timeframe is low;
- The sponsor decides to discontinue the development of the investigational product.

If the study is terminated prematurely, the subject should receive a visit as soon as possible and complete the visit as required by Section 7.1.3. For the sake of protecting the subjects, the investigator may be asked to conduct additional visits.

After termination of the study, the regulatory authorities and the Independent Ethics Committee (IEC)/Institutional Review Board (IRB) will be notified in accordance with applicable regulations.

Regulatory authorities may also request suspension or termination of the whole study.

INTERIM ANALYSES

The primary efficacy analysis may be performed at the end of all cohorts (defined as "enrollment completed and at least 2 postbaseline oncology evaluations completed").

5 STUDY POPULATION

Subjects who meet all the inclusion criteria and none of the exclusion criteria are eligible to be enrolled in this study. Before any study evaluation beyond the subject's routine medical care, the investigators should ensure that the subject or subject's legal representative has provided the written informed consent.

INCLUSION CRITERIA

I01. Subjects are able to understand the Informed Consent Form (ICF), voluntarily participate in the study and sign the ICF;

I02. Subjects ≥ 18 and ≤ 75 years old on the day of signing the ICF, male or female;

I03. Patients with histologically or cytologically confirmed metastatic or locally advanced unresectable HER2-positive solid tumor. HER2-positive is defined as follows:

- HER2 IHC 3+; **or**
- HER2 gene amplification: HER2 gene amplification (HER2/CEP17 ratio ≥ 2.0, or HER2 copy number ≥ 6) confirmed by in situ molecular hybridization ISH method (fluorescence in situ hybridization, FISH; dual-color silver-enhanced in situ hybridization, DSISH), or NGS HER2 copy number ≥ 6;

Note: HER2 status at enrollment may be confirmed by a local laboratory or a central laboratory and used for eligibility evaluation, and if HER2 status is confirmed by a local laboratory, pathological slides must be provided to the sponsor-designated central laboratory for review; local and central laboratories should not only report HER2 gene amplification, but also indicate HER2/CEP17 values. If the patient has received HER2-targeted therapy in previous lines of treatment and disease has progressed, a post-progression tumor tissue sample is required to confirm HER2 status;

Note: urothelial carcinoma includes transitional cell carcinoma or mixed transitional/non-transitional cell carcinoma, and the latter should be mainly transitional cell carcinoma in histopathology;

Note: For colorectal cancer, **wild-type RAS** is required;

I04. Prior anti-tumor therapy requires the following:

- HER2-positive GC/GEJ: Have **not received** systemic therapy for metastatic or locally advanced unresectable GC/GEJ, or **have received** ≥ 1st line systemic therapy and have disease progression, and front-line systemic therapy includes at least platinum- or fluorouracil-based chemotherapy with or without trastuzumab; subjects who have relapsed within 6 months after the end of neoadjuvant/adjuvant chemotherapy are considered to have failed 1 line of therapy;
- HER2-positive BC: **Have received** ≥ 1st line HER2-targeted therapy for metastatic disease and have disease progression; subjects who have relapsed within 12 months after the end of neoadjuvant/adjuvant systemic therapy are considered to have failed 1 line of therapy;

- Other HER2-positive solid tumors: **Have received** ≥ 1st line systemic therapy for metastatic or locally advanced unresectable tumors and have disease progression, have received the standard of care without unequivocally prolonged survival, or have refused standard of care after the 1st line systemic therapy; front-line systemic therapy for ESCC and mCRC includes at least platinum in combination with fluorouracil or taxane-based chemotherapy; mCRC requires ≥ 2 lines of systemic therapy for metastatic or locally advanced unresectable tumor with disease progression; front-line systemic therapy for ovarian and cervical cancer includes at least platinum (cisplatin or carboplatin) -based chemotherapy; subjects who have relapsed within 6 months after the end of neoadjuvant/adjuvant platinum-containing chemotherapy are considered to have failed 1 line of therapy;

I05. At least 1 measurable lesion at baseline per RECIST 1.1 criteria. If a subject has only 1 measurable lesion at baseline, the lesion area must have not received prior radiotherapy, or there is evidence of significant progression of the lesion after the end of radiotherapy;

I06. ECOG score 0 or 1 (Appendix 5);

I07. LVEF ≥ 50%, determined by echocardiography (ECHO); multiple uptake gated acquisition (MUGA) scan will be used only in the absence of ECHO, and baseline and subsequent follow-up methods will be the same;

I08. Liver function meets the following criteria within 7 days prior to the first dose:

- Total bilirubin ≤ 1.0 × ULN (Gilbert's syndrome or total bilirubin of subjects with liver metastasis ≤ 1.5 × ULN);
- Transaminase (ALT/AST) ≤ 1.5 × ULN (≤ 3 × ULN for subjects with liver metastases);

I09. Renal function within 7 days prior to the first dose: Serum creatinine ≤ 1.5 × ULN and serum creatinine clearance ≥ 60 mL/min (calculated according to the Cockcroft-Gault formula);

I10. Bone marrow function meets the following criteria within 7 days prior to the first dose:

- Hemoglobin ≥90 g/L;
- Absolute neutrophil count ≥ 1.5 × 10^9 /L;
- Platelet count ≥ 100 × 10^9/L;
- INR or PT ≤ 1.5 × ULN and aPTT ≤ 1.5 × ULN;

I11. TSH is normal; if TSH is abnormal, total T3 or free T3, and free T4 should be within the normal range;

I12. Expected survival time ≥ 3 months;

I13. Female subjects of childbearing potential or male subjects with a partner of childbearing potential agree to use highly effective contraception (see Appendix 3) from 7 days before the first dose until 24 weeks after drug withdrawal. Female subjects of childbearing potential must have a negative serum pregnancy test within 7 days before the first dose;

I14. Subjects are able and willing to comply with protocol-scheduled visits, treatment regimens, laboratory tests, and other study-related procedures.

EXCLUSION CRITERIA

E01. Subjects with untreated active brain metastases or with meningeal metastases; if the subject's brain metastases are treated and the metastasis condition is stable (brain imaging at least 4 weeks prior to the first dose shows stable disease and there are no new neurological symptoms, or the neurological symptoms have returned to baseline), and there is no evidence of new metastasis or enlargement of original brain metastases, enrollment is allowed;

E02. Decrease in LVEF to <45% or absolute decrease in LVEF of >15% during prior HER2-targeted therapy;

E03. Prior cumulative doses of anthracyclines exceeding doxorubicin, liposomal doxorubicin or other anthracyclines by > 320 mg/m^2;

E04. Subjects who have participated in any other interventional clinical study or other anti-tumor therapy within 28 days or 5 half-lives (whichever is shorter, but at least 2 weeks) prior to the first dose;

E05. Having received major surgical treatment (such as major abdominal or transthoracic surgery; excluding diagnostic aspiration or peripheral vascular access replacement) within 28 days prior to the first dose;

E06. Radical radiotherapy within 3 months prior to the first dose; palliative radiation therapy within 2 weeks prior to the first dose is allowed, the radiation dose meets the diagnostic and treatment criteria for local palliative treatment, and the radiation coverage is less than 30% of the bone marrow area;

E07. Having received immune checkpoint blockers or T-cell costimulatory drugs, including but not limited to immune checkpoint blockers such as CTLA4 and LAG3, therapeutic vaccines, etc. (subjects previously treated with PD-1 or PD-L1 inhibitors are allowed);

E08. Requiring systemic corticosteroids (≥ 10 mg/day prednisone or equivalent dose of other corticosteroids) or immunosuppressant therapy within 14 days prior to the first dose; except inhaled or topical corticosteroids, or physiologic replacement doses of corticosteroids for adrenal insufficiency; short-term (≤ 7 days) corticosteroids are allowed for prophylaxis (e.g., contrast media allergy) or for the treatment of non-autoimmune disorders (e.g., delayed type hypersensitivity due to exposure to allergens);

E09. Subjects who have received live vaccines (including live attenuated vaccines) within 28 days prior to the first dose;

E10. Current interstitial lung disease; or history of pneumonitis requiring oral or intravenous corticosteroids;

E11. Previous or current autoimmune diseases, including, but not limited to, Crohn's disease, ulcerative colitis, systemic lupus erythematosus, sarcoidosis, Wegener's syndrome (granulomatosis with polyangiitis, Graves' disease, rheumatoid arthritis, hypophysitis, uveitis), autoimmune hepatitis, systemic sclerosis (scleroderma, etc.), Hashimoto's thyroiditis (exceptions are noted as below), autoimmune vasculitis, and autoimmune neuropathy (Guillain-Barre syndrome). With the following exceptions: type 1 diabetes mellitus, hypothyroidism with stable hormone replacement therapy (including hypothyroidism caused

by autoimmune thyroid disorder), psoriasis or vitiligo that does not require systemic treatment;

E12. Having other malignancies within 5 years prior to the first dose, except cured skin squamous cell carcinoma, basal cell carcinoma, non-muscle invasive bladder cancer, localized low-risk prostate cancer (defined as stage ≤ T2a, Gleason score ≤ 6, and PSA ≤ 10 ng/mL (as measured) at diagnosis of prostate cancer, the subjects had received curative treatment and no prostate-specific antigen (PSA) biochemical recurrence), and in-situ cervical/breast cancer;

E13. History of uncontrolled intercurrent illness including but not limited to:

- Active HBV or HCV infection;
- Subjects who are HBsAg positive and/or HCV antibody positive at screening must be tested for HBV DNA and/or HCV RNA. Subjects with HBV DNA ≤ 500 IU/mL (or ≤ 2000 copies/mL) and/or HCV RNA negative are allowed; HBV DNA will be monitored by the investigator during the study at the discretion of the subject;
- Known history of HIV infection or AIDS;
- Active tuberculosis infection;
- Active infection or systemic use of anti-infective drugs for more than 1 week within 28 days prior to the first dose of KN046; fever of unknown origin within 2 weeks prior to dosing.
- Uncontrolled hypertension (BP ≥ 150/95 mmHg at rest), symptomatic cardiac insufficiency (NYHA II-IV), unstable angina or myocardial infarction within 6 months, or risk of QTc prolongation or arrhythmia (baseline QTc > 470 msec <Fridericia correction>, refractory hypokalemia, long QT syndrome, atrial fibrillation with heart rate > 100 bpm at rest, or severe valvular disease, etc.);
- Active bleeding uncontrolled after medical treatment;

E14. Toxicities of prior anti-tumor therapy did not recover to ≤ CTCAE Grade 1 (NCI-CTCAE v5.0) or baseline, with the exception of alopecia and skin pigmentation (any grade);

E15. Prior history of allogeneic bone marrow or organ transplantation;

E16. Prior history of allergic reaction, hypersensitivity reaction, and intolerance to antibody drugs; history of significant allergy to drugs (e.g., severe allergic reactions, immune-mediated hepatotoxicity, immune-mediated thrombocytopenia or anemia);

E17. Pregnant and/or lactating women;

E18. Other conditions that, in the opinion of the investigator, would affect the safety or compliance with the study treatment, including but not limited to moderate to large pleural/ascites/pericardial effusion, refractory pleural/ascites/pericardial effusion, intestinal obstruction or subacute intestinal obstruction, psychiatric disorders, etc.;

E19. BMI < 18.5 kg/m^2 or weight loss ≥ 10% within 2 months prior to screening (and the effect of large pleural and ascites on body weight needs to be considered).

CRITERIA FOR WITHDRAWAL OF SUBJECT

Discontinuation of Study Treatment

Subjects will be required to discontinue treatment with KN046 and/or KN026 if:

- PD judged as per RECIST v1.1 (Note: If the subject's ECOG performance status remains stable and the investigator determines that the subject will benefit from continued treatment with KN046 and KN026, treatment with KN046 and KN026 will be allowed after the first PD judged per RECIST v1.1 criteria, as detailed in Section 6.1.3);
- Significant clinical deterioration (clinical progression), defined as new symptoms or worsening of existing symptoms that are considered clinically significant by the investigator (if PD is not met per RECIST 1.1, the subject needs to continue oncology evaluation);
- Treatment failure requiring urgent use of other anti-tumor drugs (if applicable);
- Unacceptable toxicity (Section 6.1.4) (if study treatment is discontinued due to unacceptable toxicity, the subject needs to continue oncology evaluation);
- Pregnancy;
- Concomitant use of prohibited concomitant medications and procedures as specified in Section 6.2.2, and its consequence is discontinuation of KN046 and KN026;
- Subjects refuse to receive treatment with KN046 and KN026 (if the subject refuses to receive treatment, and PD is not met per RECIST 1.1, the subject will be asked to continue the oncology evaluation);
- Noncompliance.

Subject Withdrawal

Subjects are free to discontinue the study at any time without giving reasons. If the subject withdraws from the study, the evaluation plan required at the last visit (EOT visit) should be implemented whenever possible (see Section 7.1.3), with emphasis on the most relevant evaluations. In all cases, the eCRF records for the EOT visit should be completed whenever possible. In case of withdrawal from the study, subjects will be asked whether to continue the safety and long-term follow-up visits. The long-term follow-up visit includes collection of survival and subsequent anti-tumor therapy data.

Subjects must be withdrawn from the study in case of any following condition:

- Withdrawal of consent and refusal to continue the study;
- Participation in other interventional clinical studies during the study; in this case, the subject will continue to be followed for survival;
- Lost to follow-up.

If the data are not further collected because the subject completely withdraws from the study or does not return to the visit, the investigator must determine the primary reason for the subject's withdrawal as completely and accurately as possible and record this information on the eCRF page. For subjects who are lost to follow-up, the investigator should record in the original document the measures taken to contact the subject to confirm that "he/she has performed his/her duty", such as telephone date and registered letter.

6 INVESTIGATIONAL PRODUCTS AND SUBJECT TREATMENT

INVESTIGATIONAL PRODUCTS

KN046 and KN026 are investigational products in this study.

Dosage and Strength

The dosage form of KN046 is IV injection for single use, with the strength of 40 mg/1.6 mL/vial or 300 mg/12 mL/vial.

The dosage form of KN026 is a single-use lyophilized powder for injection at the strength of 50 mg/vial or an intravenous infusion solution at the strength of 325 mg/13 mL/vial.

Drug Preparation

Preparation of KN046

KN046 is predetermined to be administered once every 3 weeks (Q3W) by intravenous infusion.

The dose volume of each group will be calculated based on body weight. For example, for a subject with body weight of 70 kg enrolled in the 5 mg/kg group, the dose of investigational product will be 350 mg, and the dose volume will be 350 mg/40 mg * 1.6 mL = 14 mL. Aseptically withdraw 14 mL from 9 vials of KN046 drug solution and then inject into a 200 mL sterile **5% Dextrose Injection** or 0.9% Sodium Chloride Injection bag that has previously withdrawn with a volume of 14 mL. The drug solution should be mixed gently and thoroughly before infusion, and an appropriate amount of sterile **5% Dextrose Injection** or 0.9% Sodium Chloride injection should be used for rinsing at the end of infusion.

Visible foreign matters and color should be examined macroscopically before drug infusion. KN046 cannot be administered by intravenous bolus or rapid infusion. After drug preparation, a maximum error of not more than ± 10% of the theoretical dose is allowed for the actual dose.

Preparation of KN026

KN026 will be administered every 3 weeks (Q3W) by intravenous infusion (1 additional loading dose on C1D8).

Dosing volume will be calculated based on body weight. The preparation of the drug solution must strictly follow the principle of sterility. For example, for a subject with a weight of 70 kg enrolled in the 30 mg/kg group, the dose will be 2100 mg. For lyophilized powder, 42 vials are required. Add 2 mL of water for injection to each vial of KN026 and allow to stand at room temperature until complete dissolution (reconstituted drug product must be diluted and used within 4 hours); then dilute it with **normal saline** as needed to prepare a 250 mL solution; finally, invert to mix the solution and then intravenously infuse through an infusion filter, and flush the

tube with normal saline after completion of infusion. Refer to the Pharmacy Manual for specific water for injection and drug ratio.

Visible foreign matters and color should be examined macroscopically before drug infusion. KN026 cannot be administered via intravenous bolus and short infusion. After drug preparation, a maximum error of not more than ± 10% of the theoretical dose is allowed for the actual dose.

Dosage and Method of Administration

It is predetermined that subjects in this study will receive KN046 by intravenous infusion every 3 weeks. The first 6 doses will be infused over 120 minutes. If no infusion-related adverse events occur, the subsequent infusion time can be adjusted to 90-120 minutes. At the end of each dose of KN046, subjects should stay at the study site for at least 2 hours for observation.

It is predetermined that subjects in this study will receive KN026 by intravenous infusion every 3 weeks (1 loading dose on C1D8), with the infusion time for the first dose of 90 (± 15) minutes. If no infusion-related AEs occur during the first infusion, the subsequent infusion time can be adjusted to 60 (± 15) minutes. At the end of each dose of KN026, subjects should stay at the study site for at least 2 hours for observation. If KN046 and KN026 are administered on the same day, KN026 should be administered first and then KN046 after 2 hours of the end of infusion.

Body weights of subjects will be measured prior to each KN026 and/or KN046 dose, and the administered doses of KN046 and KN026 will be calculated. Each subject will receive the planned doses of KN046 and KN026 according to the protocol until radiographic disease progression, significant clinical deterioration (clinical progression), intolerable toxicity (Section 6.1.4), end of study, or other conditions meeting discontinuation criteria of KN046 and KN026 or withdrawal from the study (Sections 5.3.1 and 5.3.2). Refer to Section 6.1.4 and Appendix 3 for situations requiring modifications to the mode of administration of KN046 and KN026 (e.g., change in infusion rate, dose delay, resumption of dosing). If the subject is judged by the investigator to be still benefiting at the end of the study, treatment is allowed to continue with the consent of the sponsor.

After the first radiographic disease progression, if the subject is clinically stable, the subject is allowed to continue treatment with KN026 in combination with KN046.

Clinical stability is defined as follows:

- Stable ECOG PS score;
- No KN026- and/or KN046-related, intolerable toxicities (Section 6.1.4);
- Absence of fast disease progression that needs an anti-tumor salvage therapy;
- No acute conditions requiring urgent medical intervention due to disease progression (e.g., metastases to central nervous system, dyspnea due to tumor compression of the airways, or spinal cord compression).

Dose Modification of Investigational Products

Dose Modification of KN046

Whenever a subject experiences a protocol-specified ≥ Grade 3 toxicities related to KN046 treatment (except for laboratory abnormalities that are not clinically significant or do not meet the criteria for AEs listed below), KN046 is required to be interrupted until treatment-related toxicity resolves to ≤ Grade 1. Guidelines for dose modification of KN046 are listed in Table 2.

Table2 Guidelines for Dose Modification of KN046

Toxicities	Grade	Dose Interrupted	Criteria for Resuming Treatment	Dose after Resuming Treatment	Discontinuation Criteria
**Hematologic Toxicities**	1, 2	No	N/A	N/A	N/A
	3<br>*Exclude Grade 3 agranulocytosis alone (Grade 3 agranulocytosis alone does not require dose interruption)*	Yes	Toxicity resolves to ≤ Grade 1 within 12 weeks of last dose	Original dose	Toxicity does not resolve to ≤ Grade 1 within 12 weeks of last dose<br>*Permanent discontinuation may be considered if toxicity meets the criteria for SAEs*
	4	Yes	N/A	N/A	Permanent discontinuation
**Non-hematologic Toxicities**<br>Note: The following toxicities will be treated as Grade 1 toxicities<br>• Alopecia of any grade<br>• Grade 2 asthenia<br>• Laboratory abnormalities that are not clinically significant or do not meet the criteria for AEs	1	No	N/A	N/A	N/A
	2	Consider dose interruption if toxicity persists	Toxicity resolves to ≤ Grade 1 within 12 weeks of last dose	Original dose	Toxicity does not resolve to ≤ Grade 1 within 12 weeks of last dose
	3	Yes	Toxicity resolves to ≤ Grade 1 within 12 weeks of last dose	Original dose	Toxicity does not resolve to ≤ Grade 1 within 12 weeks of last dose<br>*Permanent discontinuation may be considered if toxicity meets the criteria for SAEs*
	4	Yes	N/A	N/A	Permanent discontinuation

If KN046 treatment-related toxicity does not recover to Grade 0-1 within 12 weeks of the last dose, KN046 will be discontinued after discussion with the Sponsor's Medical Monitor.

When treatment-related toxicities maintain at Grade 2, KN046 treatment is allowed to be resumed in rare cases, e.g., treatment-related hypothyroidism, type I diabetes mellitus, and other medical conditions with appropriate alternative therapies. In these cases, the subject will restart the KN046

treatment after the symptoms are controlled with an alternative therapy and after the discussion between the investigator and the Sponsor's Medical Monitor.

For treatment-related toxicities requiring hormonal intervention therapy, KN046 treatment may be restarted when the required dose of hormone tapers to ≤ 10 mg/day prednisone (or equivalent other corticosteroids). If the dose of hormone cannot be reduced to ≤ 10 mg/day prednisone (or equivalent other corticosteroids) within 12 weeks after the last dose, the investigator and the Sponsor's Medical Monitor need to discuss and decide whether to restart the KN046 treatment.

After resumption of KN046 treatment, permanent discontinuation of KN046 treatment is required if the same type of treatment-related toxicity reappears. For infusion-related reactions that reappear and meet the criteria for dose interruption (excluding Grade 3/4 infusion reactions), the investigator and the Medical Monitor are allowed to discuss and decide whether permanent discontinuation of KN046 is required (Section 6.2.3.1).

Permanent discontinuation of KN046 treatment may also be considered for the safety of subjects in some cases of KN046 treatment-related toxicities, including but not limited to:

- Grade 3 immune-related pneumonia;
- Recurrence of Grade 2 immune-related pneumonia lasting more than 4 weeks after active treatment;
- Grade 2 immune-related central nervous system toxicity lasting more than 4 weeks after active treatment;
- Grade 3 immune-related colitis;
- Grade 3 immune-related uveitis;
- Grade 3 immune-related hepatitis with ALT/AST ≥ 5 × ULN for more than 2 weeks; or Grade 3 immune-related hepatitis with total bilirubin ≥ 3 × ULN;
- Grade 3 immune-related nephritis and renal insufficiency;
- ≥ Grade 2 immune-related myocarditis.

The rules for the management of KN046-related irAEs are provided in Appendix 4.

As a biological product, KN046 retains ADCC activity. Refer to Section 6.2.3.4 for the management of potential tumor lysis syndrome.

If KN046 is discontinued due to an AE, the investigator should decide whether to continue treatment with KN026 in accordance with Section 6.1.4.2.

Dose Modification of KN026

Whenever a subject experiences a protocol-specified ≥ Grade 3 toxicity related to KN026 treatment (except for laboratory abnormalities that are not clinically significant or do not meet the criteria for AEs listed below), KN026 is required to be interrupted until the treatment-related toxicity resolves to ≤ Grade 1. Guidelines for dose modification of KN026 are provided in Table 3.

To monitor asymptomatic pneumonitis (CTCAE Grade 1), a chest CT scan is required. Additional chest X-rays or CT scans are required clinically. Management and dose modification of pneumonitis suspected to be related to KN026 are provided in Section 6.2.3.2.

LVEF and cardiovascular-related clinical symptoms and signs need to be monitored during treatment with KN026. Management and dose modification of cardiotoxicity suspected to be related to KN026 are described in Section 6.2.3.3.

If KN026 is discontinued due to an AE, the investigator should decide whether to continue treatment with KN046 in accordance with Section 6.1.4.1.

Table 3 Guidelines for Dose Modification of KN026

Treatment-Related Toxicity	Severity	Management Recommendations
Cardiac Toxicity		See Section 6.2.3.3
Pulmonary Toxicity		See Section 6.2.3.2
Infusion-related Reactions		See Section 6.2.3.1
Drug-related Laboratory Abnormalities	Grade 3 leukopenia or neutropenia	Continue dosing after recovery to ≤ Grade 1
	Grade 2 ALT, AST, or TBIL increased for patients with normal baseline ALT, AST, or TBIL;<br>Grade 3 or 4 ALT, AST, or TBIL increased lasting < 7 days for patients with baseline ALT, AST, or TBIL > ULN	Dose Interrupted<br>Resume the treatment if ALT, AST, or TBIL decreased to ≤ Grade 1.
	Grade 3 ALT, AST, or TBIL increased lasting ≥ 7 days or Grade 4 ALT, AST, or TBIL increased	Permanent discontinuation
	Any other Grade 4 drug-related AE or laboratory abnormality	Permanently discontinue the treatment with the following exceptions:<br>1. Individual grade 4 amylase or lipase abnormalities, duration < 7 days and no clinical manifestations of pancreatitis;<br>2. Individual Grade 4 electrolyte imbalance/abnormality, which can be

		recovered by supplementation/or appropriate treatment within 72 hours;<br>3. Alkaline phosphatase increased to Grade 4 in patients with bone metastases, and no other relevant organ function changes judged by the investigator.
Other AEs	First occurrence of other Grade 3 AEs	Interrupt the treatment, and resume the treatment if recovered to ≤ Grade 1.
	Second occurrence of the same Grade 3 AE	Permanent discontinuation
	Grade 3 AEs that cannot recover to ≤ Grade 2 within 7 days or ≤ Grade 1 within 14 days	Permanent discontinuation
	Grade 4 AEs	Permanent discontinuation

Permanent discontinuation of KN026 treatment may also be considered for the safety of subjects in some cases of KN026 treatment-related toxicities, including but not limited to:

- Congestive cardiac failure;
- Left ventricular function is significantly decreased;
- Serious infusion reactions, including serious allergic reactions, angioedema, interstitial pneumonia or acute respiratory distress syndrome;
- Pulmonary toxicities, including dyspnea, pneumonia, pneumonitis, pleural effusion, acute pulmonary edema, and insufficiency;
- Cumulatively, medication delay for more than 9 weeks or interruption for more than 3 times.

Treatment Assignment

The subject will be given a subject number after signing the informed consent form, which will be prepared from small to large in order of dates. Each subject number cannot be reassigned to other subjects once it is determined.

Packaging and Labeling of Drug

KN046 is aseptically filled in a neutral borosilicate colorless glass vial, and sealed with a halobutyl rubber stopper and an aluminum-plastic cap. Each vial of KN046 liquid formulation contains 1.6 mL of drug solution.

KN026 is aseptically filled in a neutral borosilicate colorless glass vial, and sealed with a halobutyl rubber stopper and an aluminum-plastic cap. Each 10 mL vial contains 50 mg of KN026 lyophzed powder. Each vial is packed in a carton box. Each 20 mL vial of KN026 liquid formulation contains 13 mL of 325 mg of KN026. Each vial is packaged in a carton box.

The bottle label mainly contains the following information: protocol number, drug content and strength, batch number, expiry date, storage conditions, and dosage and administration. The contents of the label will meet the requirements of current regulations.

Storage, Transportation and Shelf Life of Drug

Study drugs should be dispensed to the study site only after receipt of the requisition form in accordance with relevant regulations and the provisions of the sponsor. The drugs should be used in accordance with the following procedures. KN046 and KN026 may not be given until subjects have been enrolled in this trial. Only authorized site personnel can supply or manage the study drugs. In order to ensure the safety of the subjects and to infuse the drugs according to the regimens, the subjects must return to the study site for each dose, and should not carry and use the drugs themselves. The study drugs must be stored in a secure area that is accessible only by the investigator and authorized study site personnel and meets the storage requirements of the study drugs at 2-8°C.

Quantity Management of Drug

The investigator or authorized drug administrator is responsible for the quantity verification, dispensing and record maintenance of the study drug. In accordance with relevant regulatory requirements, the investigator or site designee must maintain a drug accountability record throughout the study. It includes the quantity of study drugs received from the sponsor and the quantity supplied to the subjects.

Assessment of Compliance

Clinical research associates will verify compliance against body weight, group, and other medical records.

Occupational Safety

KN046 and KN026 do not pose an occupational safety risk to site staff under normal compounding and dosing conditions.

CONCOMITANT MEDICATIONS AND NON-DRUG THERAPIES

Permitted Medications

The investigator may administer concomitant medications or treatments according to medical standards from the perspective of the subject's disease treatment needs. All concomitant medications will be recorded on the eCRF form, including drug treatment (e.g., prescriptions, over-the-counter drugs, herbal supplements, IV injection medications, and liquid drugs), and important non-drug therapies (including physical and blood transfusion therapies). Concomitant medications from 28 days prior to the first dose to 30 days after the last dose of KN046 and/or KN026, medication modifications during the study, and treatments received after the 30-day

safety follow-up period for the treatment of KN046- and KN026-related SAEs must be recorded in the eCRF form, including the dose, regimen, route, indication, start and end time of concomitant medications.

Palliative radiotherapy to bone (e.g., local radiotherapy to relieve bone pain, or to prevent the risk of fracture due to lytic lesions) is allowed in this study, but it is required that lesions in the area where palliative radiotherapy to bone are not selected as target lesions and palliative radiotherapy is not intended to treat tumors. PD should be judged based on RECIST 1.1 criteria rather than the need for palliative radiotherapy to bone.

Prohibited Medications and Procedures

Medications that cannot be used concomitantly during the course of the study include:

- Other anti-tumor drugs (e.g., cytotoxic drugs, radical radiotherapy or radiotherapy for the treatment of tumors, immunotherapy, cytokine therapy [except erythropoietin, G-CSF] and endocrine therapy for breast cancer) ;
- Systemic corticosteroids (except for the treatment of immune-related adverse reactions, prophylaxis requiring prior to contrast-enhanced CT angiography due to contrast allergy, alternative therapy at physiological doses, topical hormones [topical, nasal, ocular, inhaled]);
- Immunosuppressants (except for treatment of immune-related adverse reactions);
- Traditional Chinese medicine with anti-tumor indications approved by China Food and Drug Administration (CFDA);
- Other investigational drugs;
- Major surgery (except for diagnostic puncture and peripheral venous catheterization, surgical treatment is the medical routine after the tumor is down-staged);
- RANKL inhibitors (e.g., denosumab) for treatment of bone metastases;
- Botanical drugs that stimulate the immune system (e.g., mistletoe extracts).

If prohibited concomitant medications or procedures are required during the course of the study, subjects should discontinue treatment with KN046 and KN026 (see Section 5.3.1). The investigator may also contact the sponsor to discuss whether the study treatment must be discontinued.

Drugs with clear vital organ toxicity (e.g., liver, kidney, heart) should be avoided whenever possible unless necessary, and alternative drugs should be considered.

Special Precautions

Subjects should receive KN046 and KN026 infusion in a medical institution equipped with resuscitation equipment and emergency medicine, and stay in the hospital for observation for at least 2 hours after the end of each infusion. Whenever a subject receives KN046 and KN026 infusion, it must be ensured that the subject's infusion-related reactions or severe hypersensitivity reactions can be urgently handled according to local treatment guidelines. In order to ensure timely treatment of possible allergic reactions, the study site must always have appropriate rescue medications, such as dexamethasone 10 mg, epinephrine (1:1000 dilution) or alternative drugs, and assisted ventilation equipment.

KN046 and KN026 infusion should be stopped immediately in the event of ≥ Grade 2 hypersensitivity, inflammatory, or allergic reactions.

NCI recommendations for the treatment of infusion-related reactions, severe hypersensitivity reactions, and oncolytic syndrome, are provided in Sections 6.2.3.1 and 6.2.3.4, respectively.

Infusion-Related Reactions and Hypersensitivity Reactions

Symptoms of infusion-related reactions may be fever, cold intolerance, chills, sweating, and headache. Infusion-related reactions can be handled as described in Table 4.

Potential risk factors for infusion-related reactions may include:

- Elderly
- Female
- History of anaphylaxis or history of drug allergy
- Atopic dermatitis or asthma
- Concomitant medications, including beta agonists, angiotensin-converting enzyme inhibitors (ACEIs), or nonsteroidal anti-inflammatory drugs (NSAIDs).

Table 4 Handling of Infusion-related Reactions Caused by KN046 and KN026

NCI-CTCAE Grade	Dose Modification of KN046 or KN026
**Grade 1 - Mild**<br>Mild transient reaction; infusion interruption not required; intervention not required.	• Decrease the infusion rate of KN046 or KN026 by 50% and closely monitor for any signs of worsening
**Grade 2 - Moderate**<br>Treatment interruption or infusion required, and prompt symptomatic treatment (e.g., antihistamines, NSAIDs, anesthetics, IV fluids)	• Discontinue infusion with KN046 or KN026<br>• Resume infusion after resolution of infusion-related reaction or decrease in severity to at least Grade 1, with the infusion rate of 50% of the original rate<br>• Closely monitor for signs of worsening
**Grade 3 or 4-Severe or life-threatening**<br>Grade 3: Prolonged (e.g., not rapidly responsive to symptomatic medication and/or interruption of infusion); recurrence of symptoms following improvement; hospitalization due to AEs<br>**Grade 4: Life-threatening, urgent intervention required**	• Immediately discontinue infusion with KN046 or KN026 and disconnect the subject's infusion line<br>• Subjects must immediately discontinue treatment with KN046 or KN026 and must not receive any KN046 or KN026 treatment again

Treatment with KN026 or KN046 is recommended to be discontinued if a ≥ Grade 2 infusion-related reaction reappears after administration of prophylaxis; if the investigator assesses that the subject can also benefit from KN026 or KN046 treatment, the drug may be continued after discussion with the sponsor.

For sterility reasons, the drug should be used immediately after preparation within 8 hours from the completion of the preparation to the completion of the infusion.

NCI-CTCAE = National Cancer Institute-Common Terminology Criteria for Adverse Events

If an infusion-related reaction occurs with the first dose of KN026, prophylaxis, including combinations of H1- and H2-blocker antihistamines (e.g. diphenhydramine and cimetidine) and NSAIDS (e.g., acetaminophen), will be administered over 90 minutes (± 15 minutes) at 30 to 60 minutes (± 15 minutes) prior to each subsequent dose. In case of infusion reactions, the details of drug preparation and infusion must be recorded. After the 5th dose, the duration of dosing may be shortened at the discretion of the investigator.

Slow infusion of KN046 is strongly recommended for the first 6 doses and prophylactic medications should be given 30 to 60 minutes prior to the first 4 KN046 infusions. Prophylactic medications include combination of H1- and H2-blocker antihistamines (e.g., diphenhydramine and cimetidine) and NSAIDs such as acetaminophen. The recommended prophylactic regimen includes diphenhydramine 25 to 50 mg IM or equivalent PO, cimetidine 400 mg IV infusion, and acetaminophen 500-1000 mg PO. Steroids are generally not recommended as part of routine prophylactic regimens, and are used only if necessary. Montelukast may be included in the prophylactic regimen if the patient experiences symptoms of spasm bronchial. If there is no infusion-related reaction after the 4th infusion, prophylactic medications for subsequent doses should be administered at the discretion of the investigator. In vitro studies showed that KN046 caused strong immunostimulatory effects. In combination with platinum-based doublet chemotherapy, a certain proportion of infusion-related reactions and increased inflammatory

factors (IL-6, IL-10) were observed after Dose 2 of KN046; the proportion of infusion reactions decreased rapidly with the increased number of doses. For subjects who experience ≥ Grade 2 treatment-related AEs of KN046 after Dose 1, it is recommended to determine IL-6 levels before the next dose. If IL-6 levels increase, pretreatment with tocilizumab 160 mg (intravenous infusion 1-2 hours prior to KN046 administration) is allowed after consultation with the sponsor's Medical Department; for subjects who experience ≥ Grade 2 IRRs during the administration of KN046, it is recommended to determine IL-6 levels immediately, and 160 mg tocilizumab treatment is allowed on the basis of routine clinical management. After IRR recovery, resuming slow KN046 administration (at least 50% reduction in infusion rate) under respiratory, ECG, and blood pressure monitoring conditions is allowed.

Severe hypersensitivity reactions could manifest as airway injury, decreased oxygen saturation (less than 90%), confusion, lethargy, hypotension, pale skin/clamminess, and cyanosis.

If severe hypersensitivity reactions occur, subjects should be monitored immediately and injected with epinephrine and dexamethasone, and the ICU should be notified and transferred if required. Specific management principles can be found in the full version of the Emergency Treatment of Anaphylactic Reactions: Guidelines for Healthcare Providers (UK) at https://www.resus.org.uk/pages/reaction.pdf (Reference: Emergency Treatment of Anaphylactic Reactions: Guidelines for Healthcare Providers, 2008).

If a subject experiences an infusion-related reaction or hypersensitivity reaction, the investigator is advised to collect plasma histamine, IL-6, and C-reactive protein (CRP) within 30 minutes of symptom onset, and collect urine methylhistamine again within 24 hours after symptom onset, as well as unplanned ADA and PK samples. For subjects with chest pain, ECG, myocardial enzymogram (CPK, CK, Tn1, TnT), and BNP should be done within 30 minutes of symptom onset. Serum complements (C3, C4, CH50) are required for subjects with typical or atypical manifestations of "triad", such as arthralgia, rash, and/or gastrointestinal symptoms.

Note: Patients with suspected anaphylaxis should not attempt additional premedication or re-dosing after slowing down the infusion rate. If, in the opinion of the investigator, the benefit of continuing KN046 treatment outweighs the risk to the patient, the investigator is encouraged to discuss with the sponsor and to reach consensus on the rechallenge of the desensitization regimen and/or an appropriate premedication regimen.

Pulmonary Toxicity

Patients receiving HER2-targeted therapy may develop pneumonitis, including asymptomatic pneumonitis, which is only radiologically detected (CTCAE Grade 1) and symptomatic pneumonitis (pneumonitis with CTCAE Grade 2 does not affect daily activities, or CTCAE Grade 3 pneumonitis affects daily activities and patients require oxygen). To monitor asymptomatic pneumonitis (CTCAE Grade 1), a chest CT scan is required. Additional chest X-rays or CT scans are required clinically. If pneumonitis progresses, consultation with a pulmonologist is required. If a patient develops Grade 3 pneumonitis, KN026 should be withheld and the patient should be treated accordingly by a physician (e.g., short course of hormonal therapy, oxygen inhalation, etc.). Management of pneumonitis is presented in Table 5.

Table 5 Management of Pneumonitis due to KN026

CTCAE Grade	Medical Examination Required	Management of Pneumonia	Dose Modification of KN026
Grade 1	• Lung CT scans, and required pulmonary function testing includes: spirometry, DLCO, and oxygen saturation at rest.<br>• Chest X-ray or CT scan will be repeated every 6 weeks until return to baseline.	• Exclude source of infection<br>• No special treatment is required	• Maintain the original dose
Grade 2	• Lung CT scans, and required pulmonary function testing includes: spirometry, DLCO, and oxygen saturation at rest.<br>• Repeat chest X-ray or CT scan every 3 to 4 weeks (in a treatment cycle) until return to baseline.	• Exclude source of infection<br>• Hormonal therapy if cough is evident	• Resume the original dose of KN026 after recovery to ≤ Grade 1. If cough persists, hold KN026 until recovery to ≤ Grade 1.<br>• Permanently discontinue KN026 if the subject fails to recover to ≤ Grade 1 within 3 weeks after appropriate medical intervention.
Grade 3	• Lung CT scans, and required pulmonary function testing includes: spirometry, DLCO, and oxygen saturation at rest.<br>• Repeat chest X-ray or CT scan every 3 to 4 weeks (in a treatment cycle) until return to baseline.<br>• Consultation with a pulmonologist for bronchoscopy (biopsy or bronchoalveolar lavage)	• Exclude source of infection<br>• Administer hormonal therapy and gradually reduce the dose as medically judged	• Hold KN026 until recovery to ≤ Grade 1.<br>• If a subject has clinical benefit, the original dose of KN026 can be maintained (recovered to ≤ Grade 1 within 3 weeks) after recovery to ≤ Grade 1.
Grade 4	• Lung CT scans, and required pulmonary function testing includes: spirometry, DLCO, and oxygen saturation at rest.<br>• Repeat chest X-ray or CT scan every 3 to 4 weeks (in a treatment cycle) until return to baseline.<br>• Consultation with a pulmonologist for bronchoscopy (biopsy or bronchoalveolar lavage)	• Exclude source of infection<br>• Administer hormonal therapy and gradually reduce the dose as medically judged	• Permanently discontinue KN026

Cardiac Toxicity

LVEF and clinical manifestations of cardiac function should be monitored during the administration of KN026. In the event of cardiotoxicity, treatment wil be performed according to Table 6.

Table6 Management of Cardiotoxicity of KN026

**Severity**	**Management**
Symptomatic left ventricular systolic dysfunction	Permanent discontinuation
Decrease in LVEF (LVEF < 45% and ≥ 10% absolute decrease from baseline; or ≥ 15% absolute decrease from baseline) for more than 9 weeks	Permanent discontinuation
≥ 15% absolute decrease in LVEF from baseline	Hold dose; repeat LVEF every 3 weeks:<br>• Resume KN026 if LVEF ≥ 50% or LVEF ≥ 45% and < 10% absolute decrease from baseline
LVEF < 45% and ≥ 10% absolute decrease from baseline	Hold dose; repeat LVEF every 3 weeks:<br>• Resume KN026 if LVEF ≥ 50% or LVEF ≥ 45% and < 10% absolute decrease from baseline

Tumor Lysis Syndrome

KN026 and KN046 have the potential to cause ADCC effects and therefore are at risk for tumor lysis syndrome. Once it occurs, management may be performed according to Figure 3.

Figure 3 Evaluation and Initial Treatment of Tumor Lysis Syndrome

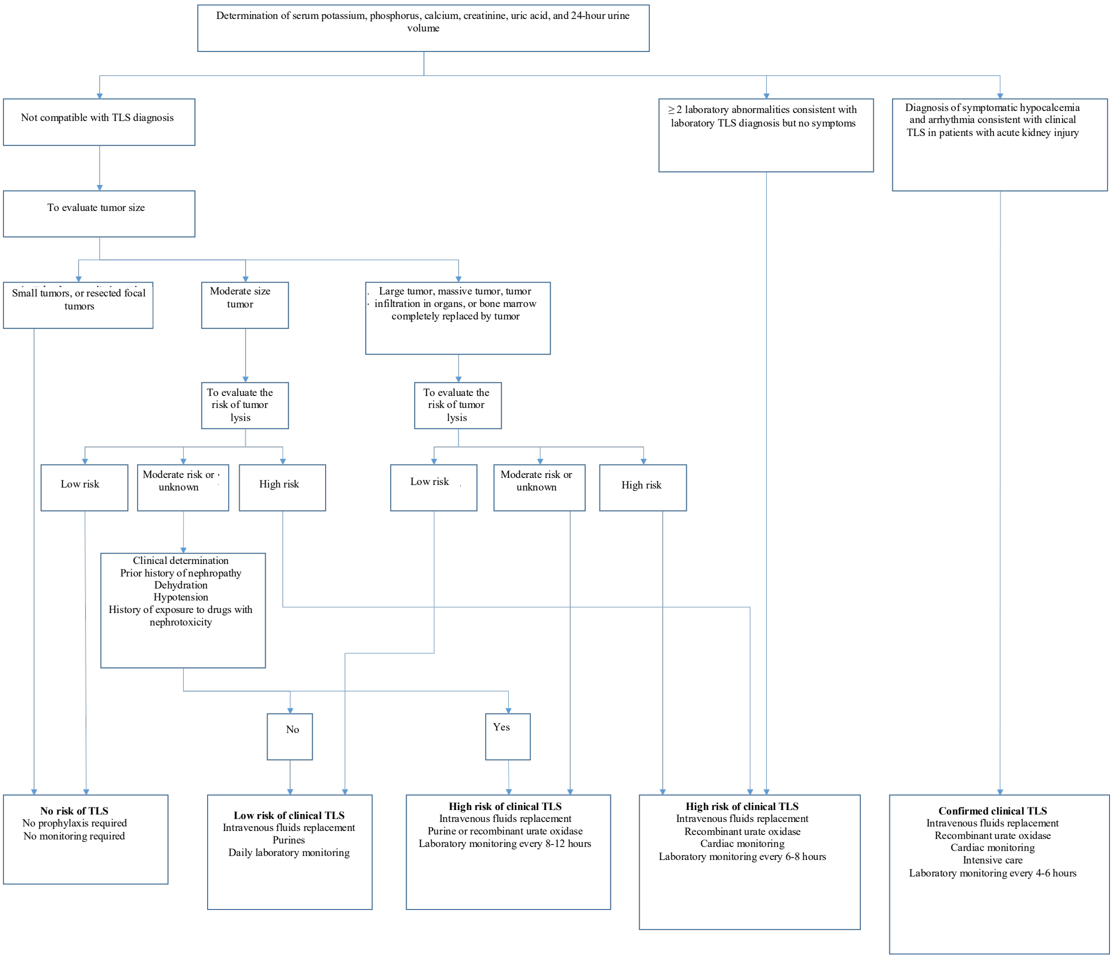

Note: Howard et al, 2011; TLS: tumor lysis syndrome

7 STUDY PROCEDURES AND EVALUATIONS

STUDY VISIT PLAN

The evaluation plan for this study is detailed in Table 1.

Screening Period

Subjects should sign the ICF before screening and the start of study evaluation. The screening period of this study is within 28 days prior to the administration of the study drug.

Subject information, including demographic information (date of birth, sex, and race) and complete medical history (including tumor history, prior and concomitant medications, prior surgery and radiotherapy, and baseline medical condition, etc.) will be recorded at screening.

Subjects will undergo a full physical examination at screening, including recording of weight, height, vital signs, 12-lead ECG, echocardiography/MUGA, and determination of ECOG performance status.

Safety laboratory tests, including hematology, coagulation, serum chemistry, and urinalysis, must be completed within 7 days pre-dose. Total triiodothyronine (TT3), free T3 (FT3), free thyroxine (FT4), and thyroid stimulating hormone (TSH) will also be evaluated at screening. All subjects should be tested for hepatitis B virus, hepatitis C virus, and human immunodeficiency virus (HIV).

Women of childbearing potential at screening should have a serum β-chorionic gonadotropin test within 7 days prior to study drug administration unless the age-related spontaneous menopause for ≥ 12 consecutive months and follicular estrogen (FSH) increase > 40 mIU/ml, surgical sterilization, or sexual abstinence. Women of non-childbearing potential can be waived from pregnancy tests. If confirmation of menopausal status is required, FSH may be measured at screening.

Oncology (Section 7.2.2), HER2 status, and biomarker evaluation (Section 7.2.6) will also be performed at screening.

In the event of screening failure due to abnormal laboratory tests, a repeat laboratory test is allowed upon agreement between the investigator and the Medical Monitor. Failure of a subject to reach the eligibility criteria at screening will result in screening failure and inability to receive study medication. For subjects who fail screening, the following information needs to be recorded on the eCRF:

- Informed Consent Page;
- Reason for screening failure;
- Demographic information;

- Adverse event;
- Inclusion/Exclusion Criteria Page.

Treatment Period

The treatment period begins from Cycle 1 Day 1 of KN046 and KN026 administration and continues until disease progression (see Sections 6.1.3 and 7.2.2.3), significant clinical deterioration (clinical progression), unacceptable toxicity, the subject withdrawal from the study or study treatment, or end of study (see Sections 5.3.1 and 5.3.2).

After disease progression per RECIST 1.1 (Solid Tumors) criteria, if the subject is clinically stable and can benefit from continuing KN026 and KN046 treatment as judged by the investigator, the subject is allowed to continue treatment with KN026 and KN046 (see Section 6.1.3). In this case, subjects may be treated with reference to the iRECIST (solid tumors) criteria (Appendix 2). If treatment with KN046 and KN026 is continued after the progression is confirmed per RECIST 1.1 criteria, KN046 and KN026 should be discontinued immediately once the subject is intolerant of the study drug or experiences treatment failure (see Sections 6.1.3, 5.3.1, and 5.3.2).

During the treatment period, subjects will have visits as specified in the protocol. The window for the visit period is up to 3 days (± 3 days) before or after the scheduled visit day (except for the PK and ADA sampling visits required in Table 2).

The evaluations performed during the treatment period are detailed in and should follow the provisions of Tables 1 and 2.

End of Treatment Visit (EOT)

Subjects who discontinue treatment with KN046 and KN026 for any reason must have an EOT visit. The EOT visit should be performed within 7 days of the decision to discontinue treatment, but before starting a new anti-tumor therapy, if applicable, whichever occurs first. The EOT visit may also be performed on the day of the decision to discontinue treatment with KN046 and KN026; if it is within 7 days after the last pre-treatment evaluation, the same tests as those of EOT visit need not be repeated.

The eCRF page for EOT visit must include the date of the decision to discontinue KN046 and KN026 treatment, the date of the last KN046 and KN026 treatment, and any of the following reasons:

- Adverse event;
- Abnormal laboratory value(s);
- Abnormal operation results;
- Protocol deviations;

- Withdrawal of informed consent;
- Lost to follow-up;
- Death (must be noted it is caused by "study indication" or "other" reasons);
- Disease progression as per RECIST 1.1 criteria;
- End of treatment according to the protocol requirements;
- Other causes, including clinical progression, administrative problems.

If no PD as defined by RECIST 1.1 occurs at the EOT visit, subjects will be asked to continue tumor evaluations. If a subject withdraws the ICF, it must be clearly stated that if he/she is willing to continue the follow-up after the end of treatment (see Sections 5.3.2, 7.1.4, 7.1.5).

See Table 1 for the specific assessments at the EOT visit.

30-Day and 90-Day Safety Visits

A safety follow-up visit is required for subjects 30 days (± 3 days) after the last dose of KN026 and/or KN046 and 90 days (± 7 days) after the last dose of KN046. For subjects who discontinue KN046 treatment first and continue to receive KN026, the 90-day safety follow-up visit is not required if 30 days after the last dose of KN026 are later than 90 days after the last dose of KN046.

After the EOT visit, AEs need to be recorded up to 30 days after the last dose of study drug. Thereafter, all SAEs and treatment-related non-serious AEs (including irAEs) will be recorded until 90 days after the last dose of KN046. SAEs that persist after the 30-day or 90-day safety follow-up visit are required to be monitored by the investigator until stabilization or until the outcome is known.

Subsequent anti-tumor therapies, tumor imaging evaluations (see Section 7.2.2, if applicable), etc. will also be collected during the safety follow-up period. Please refer to Table 1 for the contents of visits during the safety follow-up period.

Long-term Follow-up

Serious adverse events (SAEs) that continue beyond the 90-day safety follow-up visit will need to be monitored in the long-term follow-up until stabilization or until the outcome is known, unless the subject is documented as "lost to follow-up". The KN046 and/or KN026 treatment-related SAEs should be recorded, regardless of the time of occurrence or the time from discontinuation of KN046 or KN026, and reported to the regulatory authorities in accordance with local regulatory requirements.

The survival status information and subsequent anti-tumor therapy will be collected every 12 weeks (± 14 days) after the decision to discontinue treatment, regardless of whether or not the subject has an EOT visit. Survival follow-up will continue until 1 year after the last subject

completes KN026 and/or KN046 treatment; the completion of the last study-related telephone contact or visit, withdrawal from the study or lost to follow-up (i.e., the investigator is unable to contact the subject) of the last subject; death or premature withdrawal from the study of all subjects; or the sponsor assesses that the study has met the study expectations (e.g., completion of the primary endpoint assessments) (Section 4.1.1).

Subjects who have no PD as defined by RECIST 1.1 after the EOT visit will be asked to continue tumor evaluations (Section 7.2.2.3).

The evaluations to be completed during the long-term follow-up period are detailed in Table 1.

End of Study

The end of the study is defined as 1 year after the last dose of all subjects; the completion of the last study-related telephone contact or visit, withdrawal from the study or lost to follow-up (i.e., the investigator is unable to contact the subject) of the last subject; death or premature withdrawal from the study of all subjects; or the sponsor assesses that the study has met the study expectations (e.g., completion of the primary endpoint assessments), whichever occurs first.

Note: If the sponsor concludes that the study has met the expectations, but there are still patients on treatment, the sponsor will provide a subsequent protocol to continue providing KN046 and/or KN026 and certain safety follow-up visit for the patients.

STUDY EVALUATIONS

Demographics and Other Baseline Characteristics

The study evaluations described in this section must be completed at screening.

Demographic Data

The following data are collected during the screening period:

- Date of birth;
- Sex;
- Race;
- Ethnicity.

Tumor Diagnosis

Tumor disease information for each subject needs to be confirmed and documented at screening, including:

- The detailed tumor history, including histopathological type, grade and stage, determined according to the criteria for tumors, lymph nodes, and metastasis classification from the Union for International Cancer Control (UICC) at the time of diagnosis;
- All prior anti-tumor therapies (including surgery, radiotherapy and chemotherapy, immunotherapy);
- Any other disease receiving chemotherapy, radiotherapy, or immunotherapy;
- Current tumor symptoms and signs, and adverse reactions to current and/or prior anti-tumor therapies;
- Current tumor status.

Medical History

The complete medical history of each subject should be collected and documented at screening to confirm whether the subject is eligible for inclusion. The complete medical history should contain at least the following information:

- Prior non-tumor diseases and concomitant therapies;
- Prior tumor diseases and concomitant therapies;
- Medications (including botanicals) and procedures received within 28 days prior to screening;
- History of tobacco and alcohol use;
- Family history of tumor.

Other Baseline Evaluations

Additional baseline evaluations include oncology evaluations, vital signs, full physical examinations, ECOG score (Appendix 5), laboratory tests (Section 7.2.3.5), 12-lead ECG, ECHO/MUGA, biomarker tests, and evaluation of inclusion/exclusion criteria (Table 1).

Refer to Section 7.2.2.2 for baseline oncology evaluation; Section 7.2.3 for baseline safety evaluation; Section 7.2.6 for biomarker tests; and Sections 5.1 and 5.2 for evaluation of inclusion/exclusion criteria.

Efficacy Evaluation

In this study, the investigator will evaluate tumors according to RECIST 1.1 (Appendix 1). Results assessed by the investigator will be used to guide clinical decisions (e.g., discontinuation

of KN046 and KN026 treatment) and for the analysis of primary and secondary efficacy endpoints.

Computed Tomography (CT), MRI, FDG PET/CT, and Other Evaluations

All subjects will have chest/abdomen/pelvis (specific tumor types need to include other specific areas) CT scan or MRI (chest CT is mandatory if MRI is used). If CT/MRI imaging is not sufficient to evaluate tumor burden, other established evaluation methods may be added. Contrast-enhanced CT scan is recommended, and contrast-enhanced MRI may be considered if the subject is allergic to contrast.

Preferably, CT scan or MRI are performed using a 5 mm tomography thickness with a continuous reconstruction algorithm. The tomography thickness in the CT/MRI scan should not exceed 8 mm using a continuous reconstruction algorithm. All scans performed at baseline and other imaging performed as clinically required (other supportive imaging) need to be repeated at subsequent visits. In conclusion, the imaging method needs to be consistent with that used for lesion detection at baseline, and the same imaging device is preferred for subsequent tumor evaluation visits.

If no brain CT/MRI scan is performed within 42 days prior to screening, it should be performed at screening (either method can be used and it is recommended to use the contrast-enhanced method), and a brain CT/MRI scan may be added as clinically indicated at subsequent follow-up if there are new central nervous system symptoms. If no bone scan is performed within 3 months prior to screening, it should be performed at screening, and additional bone scan may be considered at subsequent follow-up if clinically indicated.

For each subject, the investigator will assign 1 or more of the following tumor evaluation methods to determine response to treatment or PD: CT or MRI, physical examination, and other evaluation results of primary and/or metastatic tumor lesions. During the study period, imaging methods that are most appropriate for the subject, as well as methods that are most appropriate for evaluating the subject's tumor status should be considered. The evaluation method of tumor status of subjects during the study should be consistent with that used at the time of enrollment.

Baseline Oncology Evaluation

For subjects with solid tumors, target and non-target lesions will be recorded for each subject at screening according to RECIST 1.1 criteria for baseline oncology evaluation.

Subjects must have measurable lesions as judged by the investigator per RECIST 1.1 criteria at baseline (Section 5.1). Subjects with only non-measurable lesions are not eligible for the study.

Measurable lesions is defined as at least 1 measurable non-nodal or nodal lesion by the RECIST 1.1:

- Measurable non-nodal lesions: lesions that can be accurately measured with at least 1 dimension and have the diameter not be less than 2 times the thickness of the tomography scan (e.g., if the thickness on spiral CT scan or MRI is 5 mm, the diameter of the lesion must be ≥ 10 mm);

- Measurable lymph node lesions: lymph nodes ≥ 15 mm in short axis;
- Bone lesions selected as target lesions must be osteolytic or mixed osteolytic-osteoblastic lesions that can be identified by CT/MRI and contain soft tissue components.

Non-measurable lesions refer to all other non-measurable lesions, including small lesions (e.g., if the tomography thickness is 5 mm, the long axis of the CT or MRI scan is < 10 mm, or the short axis of the pathological lymph node is ≥ 10 mm and < 15 mm). Examples of non-measurable lesions include osteoblastic lesions, leptomeningeal disease, ascites, pleural/pericardial effusion, inflammatory breast disease, cutaneous lymphangitis/pneumonia, abdominal masses not confirmed and followed by imaging techniques, and cystic lesions.

Lesions at sites that are treated with radiotherapy previously should be considered non-measurable unless PD is demonstrated by unequivocal imaging and the lesion is measurable.

Up to 5 measurable lesions (nodal and non-nodal; up to 2 lesions per organ) will be selected as target lesions and represent, whenever possible, all organs involved. Target lesions should be selected based on size (lesions with the maximum diameter) and suitability for accurate repeated measurements. Each target lesion must be measured at baseline and uniquely numbered sequentially on the eCRF (even if located in the same organ).

Non-target lesions include all other lesions, i.e., lesions that do not meet the criteria for target lesions at baseline. Multiple non-target lesions involved in the same organ may be evaluated as a group and recorded as a single item. Measurement of these lesions is not required. Any non-target lesion identified at baseline must be recorded on the eCRF.

Subsequent Oncology Evaluations

Subsequent oncology evaluations will be performed every 6 weeks (± 7 days) from the start of treatment, and every 12 weeks (± 7 days) after 48 weeks until radiographic disease progression, start of a new anti-tumor therapy, withdrawal of consent, lost to follow-up, and end of study, whichever occurs first. Subjects who discontinue KN046 treatment due to intolerable toxicity or clinical progression should continue to be followed up for imaging and oncological evaluation until radiographic disease progression occurs.

All target or non-target lesions identified at baseline need to be evaluated at subsequent follow-up visits and documented on the appropriate eCRF page, and the evaluation method needs to be consistent with that at baseline. Whenever possible, the same radiologist should perform all oncology evaluations of the subject at baseline and subsequent follow-up visits. New lesions, either measurable or non-measurable, present subsequently rather than identified at baseline should be evaluated and recorded on the corresponding eCRF page.

If a subject experiences a CR or PR as defined by RECIST 1.1, a CT or MRI scan should be performed 6 weeks later (but within 4 weeks) as planned to confirm the CR or PR (confirm response).

If a subject experiences PD as defined by RECIST 1.1 criteria for the first time and is clinically stable, the disease progression may be confirmed 6 weeks later (no earlier than 4 weeks and no later than 8 weeks) for clinical treatment decision making (Section 6.1.3).

Safety Evaluation

Each subject will be monitored for safety in this study, including hematology, blood chemistry and electrolytes, coagulation, urinalysis, 12-lead ECG, ECHO/MUGA, physical examination, vital signs, height, weight, ECOG score, endocrine test, and immunogenicity test. Detailed tests and frequencies are presented in Table 1.

Physical Examinations

General physical examination will include: general appearance, skin, neck (including thyroid), eyes, ears, nose, throat, lungs, heart, abdomen, back, lymph nodes, extremities, blood vessels, nervous system, and vital signs (Section 7.2.3.2).

Systemic physical examination will be performed at the following visits:

- Screening;
- EOT visit.

Symptom-directed physical examinations will include: general condition, vital signs (Section 7.2.3.2), and abnormal findings from physical examination.

Symptom-directed physical examinations will be performed at the following visits:

- Prior to each dose of KN026 and/or KN046;
- 30- and 90-day safety follow-up.

Physical examination needs to be documented in the source documents of the study sites. Physical examination abnormalities prior to signing of ICF should be recorded on the appropriate eCRF page for the past/current medical history. New or worsening physical examination abnormalities after signing ICF should be recorded as AEs on the appropriate eCRF page.

Vital Signs

Vital signs will include respiratory rate, pulse rate, blood pressure, and temperature, and will be performed at the following visits:

- Screening;
- Prior to each dose of KN026 and/or KN046;
- EOT visit;

- 30- and 90-day safety follow-up.

If vital sign measurements coincide with the safety laboratory or PK blood sampling, vital sign measurements should be performed first.

Height, weight

Height will be measured at screening.

Weight measurement will be performed at the following visits:

- Screening;
- Prior to each dose of KN026 and/or KN046;
- EOT visit;
- 30- and 90-day safety follow-up.

Body weights measured prior to each dose of KN026 and/or KN046 will be used to calculate the administered doses of KN046 and KN026 (Section 6.1.2).

ECOG PS score

ECOG scoring will be performed at the following visits:

- Screening;
- Prior to each dose of KN026 and/or KN046;
- EOT visit;
- 30- and 90-day safety follow-up.

ECOG PS scoring method is presented in Appendix 5.

Laboratory Tests

Laboratory tests for safety evaluation will be performed at the local laboratory, and the normal range of the local laboratory will be adopted, including: hematology, serum chemistry electrolytes, coagulation, urinalysis, endocrine tests, and urine pregnancy/serum pregnancy test (Table 8).

Prior to shipment of study drug, the study sites must provide the sponsor with a list of normal ranges for the laboratories of study sites. Changes in the normal ranges of the laboratory during the course of the study are required to be provided to the sponsor-designated CRO.

Subjects will be fasted for at least 8 hours prior to blood sampling, and samples will be collected prior to the dose of KN046 and KN026. All routine laboratory tests will be analyzed in the local laboratory of the study site, and test results that are clinically significant for the subject's treatment decision (e.g., hematology, blood chemistry and electrolytes, endocrine tests) must be known and evaluated prior to the dose of KN046 and KN026. Reporting of results must be maintained as part of the subject's medical records or source documents, and documented in the eCRF.

Table7 Laboratory Test Items

**Blood chemistry**	**Hematology**	**Coagulation function**	**Urinalysis**
Alkaline phosphatase	Red blood cell count	Prothrombin time (PT)	pH
Alanine aminotransferase （ATL）	Hematocrit	Activated partial thromboplastin time (aPTT)	Specific gravity
Aspartate aminotransferase (AST)	Haemoglobin	Thrombin time (TT)	Glucose
Albumin	Mean haemoglobin	International Normalized Ratio (INR)	Protein
Total bilirubin	Mean haemoglobin concentration	**Hormone levels**	Ketone bodies
Direct and indirect bilirubin	Mean corpuscular volume	Follicle-stimulating hormone (if applicable)	Red blood cells urine
Serum total urea (or urea nitrogen)	Platelet count	Thyrotropin (TSH)	Leucocytes
Blood calcium	WBC count with differential	Free thyroxine (FT4)	
Chloride	Neutrophil count and percentage	Total triiodothyronine (TT3)	
Creatinine	Lymphocyte count and percentage	Free T3 (FT3)	**Viral testing**
Glucose	Monocyte count and percentage	**Pregnancy test (if applicable)**	HbsAg, HBsAb, HBeAb, HBeAg, and HBcAb (if HBsAg is positive, additional HBV DNA test is required)
Lactate dehydrogenase	Basophil count and percentage	Serum β-human chorionic gonadotropin (HCG)	HCV antibody (if HCV antibody is positive, HCV RNA is required)
Phosphate	Eosinophil count and percentage	Urine HCG	HIV antibody

Total protein			
Blood potassium	Troponin (if applicable)		
Blood sodium	IL-6 (if applicable)		
Gamma-glutamyl transpeptidase (γ-GT)	C-reactive protein (if applicable)		

1. For urinalysis, if urine protein is ≥ 2+ (dipstick), a 24-hour urine sample will be collected for determination of total protein and a random urine sample will be collected for determination of protein/creatinine ratio

Pharmacokinetic Evaluation

The PK evaluation plan is presented in Tables 1 and 2.

The PK blood sample collection should be recorded on the appropriate eCRF page, including the exact date and clock time of administration and blood sample collection of KN046 and KN026.

Whole blood samples are drawn by direct venipuncture or by inserting a venous catheter or indwelling trocar into the forearm vein. Samples will be processed, labeled, stored, and shipped as detailed in the laboratory manual. PK blood samples are tested by a central laboratory.

If the PK samples and immunogenicity (anti-KN046 and anti-KN026 antibodies) samples are scheduled to be collected at the same time, all of these samples should be collected at the same time with the exact collection time of each sample recorded.

Immunogenicity Evaluation

The immunogenicity of KN046 and KN026 is evaluated by detecting anti-drug antibodies (ADAs) and neutralizing antibodies (NADAs). The detection time for ADA is presented in Table 2. Subjects who are positive for the ADAs will be further tested for antibody titers.

The immunogenicity samples should be collected, processed, standardized, stored, and shiped as specified in the laboratory manual. Immunogenicity testing will be performed at the central laboratory.

Biomarker Evaluation

Biomarker Evaluation of Tumor Tissue

All subjects will be required to submit slides for central laboratory review of HER2 status at screening. Subjects who have received HER2-targeted therapies such as trastuzumab should provide tumor tissue samples collected after failure of HER2-targeted therapy for determination of HER2-positive status (Section 4.1).

Subjects who agree to participate in the non-mandatory biomarker testing may submit recently obtained formalin-fixed, archival sample (biopsy or surgy) containing tumor tissue (blocks or

slides) (from non-irradiated areas within 2 years) for PD-L1 expression, TIL, GEP, and HER2/CDK12 co-amplification analysis (breast cancer cohort) at screening. The biomarker evaluation plan is presented in Table 1. Biomarker samples should be collected, processed, stored and shipped in accordance with the laboratory manual and materials provided by the sponsor or its designated central laboratory.

Tissue collection: Samples should be collected at screening, and tissues (blocks or slides) from archival specimens should be obtained within 2 years prior to screening. Samples from endoscopic biopsy, aspiration biopsy, excisional biopsy, trephine biopsy, and surgery are acceptable, but those from fine needle aspiration biopsy are not acceptable.

Provision of samples: Priority 1: tumor-containing FFPE tissue block; priority 2: if the entire tumor-containing FFPE tissue block cannot be provided, slides of this block should be provided: it should be freshly cut (within 6 months), be 4-6 μm thick, and prepared on adhesion slides.

Tissue processing: Tumor tissues should be fixed in 10% neutral formalin, embedded in paraffin, and routinely processed for histological evaluation. Formalin substitutes are not suitable as a fixative.

Samples and tissue bank: Biomarker samples may be stored until the end of the study and analyzed together with samples from other studies to analyze the relationship of biomarkers to the efficacy of the investigational product or the study disease, and to develop possible diagnostic kits.

8 EVALUATION AND RECORDING OF ADVERSE EVENTS

DEFINITION OF ADVERSE EVENT

Adverse Event

An adverse event (AE) is an untoward medical occurrence in a subject while receiving treatment with a medicinal product that does not necessarily have a causal relationship with the treatment. An AE may therefore be a discomfort or unexpected sign (including abnormal laboratory finding), symptom, or disease temporally related to the use of a medicinal product, regardless of the causality to the medicinal product.

For a surgical or diagnostic procedure, the condition/disease that led to the procedure, rather than the procedure itself, should be considered an AE. Conditions such as scheduled examinations and surgeries (including endoscopy, appendectomy), hospitalization for convenience or social reasons, and pre-existing symptoms or daily fluctuations rather than worsening of the disease prior to the start of the study are not considered AEs.

Serious Adverse Event

A Serious Adverse Event (SAE) is an untoward medical occurrence at any dose that results in any of the following outcomes:

- Results in death;
- Life-threatening;

Note: The term "life-threatening" in the definition refers to a subject who is at risk of death at the time of the event; it does not refer to an event that hypothetically might cause death if it is more severe.

- Requiring hospitalization or prolongation of existing hospitalization;
    - Hospitalization due to social reasons or convenience reasons (e.g., insurance, inconvenience of accommodation of out-of-town patients), rather than due to the AE itself, is not required to be reported as an SAE;
- Results in permanent or serious disability/incapacity;
- Results in congenital anomaly/birth defect;
- Other medically significant events.

Note: Significant medical events that do not result in death, are not life-threatening, or do not result in hospitalization may be assessed as SAEs if they may cause damages to the subjects or need medical or surgical intervention to prevent any of the above SAEs based on the medical

judgment by the investigator. Examples of such events include allergic bronchospasm requiring intensive treatment in an emergency room or at home, hematologic disease or convulsions that do not result in hospitalization, or development of drug dependence or drug abuse.

DOCUMENTATION AND REPORTING OF ADVERSE EVENTS

This study requires documentation of any untoward medical occurrence during the study after signing the ICF until 30 days after the last dose of study treatment or initiation of a new anti-tumor therapy, whichever occurs first. SAEs and TRAEs will be collected up to 90 days after the last dose of KN046 or 30 days after the last dose of KN026, whichever occurs later. SAEs suspected to be related to study drug should be recorded and reported at any time, regardless of the time from discontinuation of study drug. The symptomatic left ventricular systolic dysfunction and asymptomatic left ventricular systolic dysfunction (≥ 10% and < 50% decrease in LVEF from baseline) within 12 months after the last dose of KN026 should be recorded and reported as AEs, and those meeting the criteria for SAEs should be reported to regulatory authorities in accordance with local regulations.

The investigator will grade each AE according to NCI-CTCAE 5.0. If the severity/intensity of a particular AE is not specifically graded by the guideline, the investigator will grade in accordance with the following general definitions of Grade 1 through Grade 5, with his/her best medical judgment:

- Grade 1: Mild; asymptomatic or mild symptoms; clinical or diagnostic observations only; intervention not indicated;
- Grade 2: Moderate; minimal, local or noninvasive intervention indicated; limiting age-appropriate instrumental activities of daily living (ADL) (e.g., preparing meals, shopping for groceries, using the telephone, managing money, etc.);
- Grade 3: Severe; serious or clinically significant but not immediately life threatening; hospitalization or prolongation of hospitalization indicated; disabling; limiting self-care ADL (e.g., bathing, dressing and undressing, feeding self, using the toilet, taking medications), without bedridden;
- Grade 4: Life-threatening; urgent intervention indicated;
- Grade 5: Deaths related to AEs.

Each AE report should include a description of the event, duration (date/time of onset and resolution: if it is necessary to evaluate the time of onset of the AE relative to dosing, "Time of administration" should be also recorded), severity, relationship with the study drug, other potential causes leading to the AE, any treatment administered or other action taken (including delay or discontinuation of the study drug), and outcome. In addition, SAEs will be identified, with the corresponding serious criteria recorded.

In case of death, primary causes (the events leading to death) should be recorded and reported as SAEs. "Fatal" will be recorded as the outcome of the event; death itself could not be recorded as

an SAE separately. Only if the cause of death is unknown (e.g., sudden death, unknown cause of death), the death itself can be reported as an SAE.

Symptoms or signs of tumor progression will not be recorded as AEs, but if disease progression is more severe than expected or if tumor progression is considered by the investigator to be related to study drug administration or study procedures, an AE or SAE will be reported. The diagnosis should be recorded on the eCRF rather than the individual symptoms and signs (e.g., liver failure should be recorded, rather than jaundice, elevated transaminase with flapping tremor). However, if the symptoms and signs cannot be classified as an individual diagnosis at the time of the reporting, each individual event should be recorded on the eCRF as an AE or an SAE. And if a diagnosis is subsequently established, it should be reported as follow-up information.

The investigator should assess the causality of each AE or SAE to study drug based on clinical condition:

- **Not related:** no reasonable relationship to study drug is suspected. The occurrence of the event is more likely to be explained by other factors than the study drug;

- **Related:** a reasonable relationship to study drug is suspected. The AE could be medically (pharmaceutical/clinical) attributed to study drug.

AEs should be recorded completely, accurately and consistently, as detailed in the "eCRF Completion Guidelines".

Serious Adverse Event Recording and Reporting

In the event of any new SAE (any grade) during the reporting period, the investigator must immediately (within 24 hours of becoming aware of the event) complete the Serious Adverse Event Report and email the report to the sponsor's Pharmacovigilance Department: drugsafety@alphamabonc.com. In the event of any new conditions for previously reported SAEs occurring, the reporting procedures and time limits are the same as for the previously reported SAEs.

All written reports should be sent using the SAE Report Form, which must be completed by the investigator in accordance with the specific completion instructions. In addition, the AE section of the eCRF must be completed. Relevant pages of the eCRF (e.g., medical history, concomitant medications) may be provided in parallel. The information provided in the SAE Report Form must be consistent with the data of the events recorded in the corresponding sections of the eCRF all the time. If the sponsor or its designee requests follow-up information of SAEs (e.g., other information, outcome and final evaluation, specific records (if required)) or has questions about the SAE reports, the investigator should respond immediately. In this way, the sponsor or its designee can evaluate the events promptly, and the company can meet stringent time requirements of the regulatory authorities on the obligation of expedite reporting of safety events. Requests for follow up are usually made by the monitor responsible for the study site, but the sponsor may also contact the investigator directly to clarify or discuss specific key events in case of exceptions.

The investigator should promptly report all SAEs to the Ethics Committee in accordance with regulatory requirements, in addition to reporting them to the sponsor.

The sponsor has an obligation to report safety to regulatory authorities. The sponsor or its designee will submit safety reports to the regulatory authorities, investigators, or EC (if applicable), in accordance with regulatory requirements in China. The sponsor will prepare reports of suspected unexpected serious adverse reactions (SUSARs) or SAEs of the study drug and send them to the relevant investigators as required by regulations.

Follow-up of SAEs

If a subject experiences an SAE, it should be followed up by the investigator (for clinical symptoms/signs, laboratory test abnormalities, or other) until it returns to normal or baseline level, or the clinical outcomes are stable. Therefore, the SAE follow-up may continue beyond the end of the study, and the sponsor may also request the SAE follow-up results after the end of the study.

Adverse Events of Special Interest

"Adverse events of special interest" occurring during the study need to be reported promptly to the sponsor's medical department, safety department, or designee. The investigator should report the "adverse event of special interest" to the sponsor within 24 hours of becoming aware and record it on the Adverse Event Form of the eCRF, whether or not it is an SAE.

Adverse events of special interest include:

- ≥ Grade 3 infusion-related adverse reactions or hypersensitivity reactions;
- KN046
    - ≥ Grade 3 sensory and motor neuropathy (including Guillain-Barre syndrome, myasthenia syndrome) resulting in discontinuation of KN046;
    - Any suspected KN046 treatment-emergent serious adverse drug reactions (TESADRs) during non-hematologic treatment; uncommon TESADRs, including but not limited to, serum sickness, myocarditis, haemolytic anaemia, Lambert-Eaton syndrome, myasthenia gravis, rhabdomyolysis syndrome, partial epileptic seizure, vasculitis, and pemphigus; any treatment-related serious adverse drug reactions (TRSADRs) leading to discontinuation of KN046;
    - ≥ Grade 4 immune-related hematologic toxicity; ≥ Grade 4 skin toxicity;
    - ≥ Grade 3 immune-related pneumonia;

    - Recurrence of Grade 2 immune-related pneumonia lasting more than 4 weeks after active treatment;
    - Grade 2 immune-related central nervous system toxicity lasting more than 4 weeks after active treatment;
    - ≥ Grade 3 immune-related colitis;
    - ≥ Grade 3 immune-related uveitis or optic neuritis;
    - ≥ Grade 3 immune-related hepatitis with ALT/AST ≥ 10 × ULN; or ALT/AST ≥ 5 × ULN and lasting more than 2 weeks; or ALT/AST ≥ 5 × ULN with total bilirubin ≥ 3 × ULN; or ALT/AST ≥ 5 × ULN with gastrointestinal symptoms (e.g., nausea, vomiting, tenderness of right upper abdomen);
    - ≥ Grade 3 immune-related nephritis and renal insufficiency;
    - ≥ Grade 1 immune-related myocarditis.

- All cardiac adverse events (whether related to KN026 or not) occurring from the dose of KN026 through 1 year after the last dose need to be reported, including the following categories. In addition, all symptomatic left ventricular systolic dysfunction occurring within 3 years after the last dose of KN026 are required to be reported.
    - Symptomatic left ventricular systolic dysfunction;
    - Asymptomatic left ventricular systolic dysfunction with the following:
        - Decrease in LVEF ≥ 10% from baseline and absolute LVEF < 50%;
        - Decrease in LVEF ≥ 15% from baseline.

Recording and Reporting of Laboratory Abnormalities and Other Abnormalities

Laboratory test abnormalities and other abnormal study findings (e.g., ECGs) that are judged to be clinically significant, such as having related clinical symptoms and signs, leading to treatment interruption, requiring medical intervention or changing concomitant therapy, should be recorded as AEs; the laboratory test abnormalities that are judged to be not clinically significant are not required to be recorded as AEs. If a clinically significant laboratory abnormality or an abnormal vital sign is a sign of a disease or syndrome (e.g., ALP and bilirubin increased to > 5× ULN as a result of cholecystitis), only the diagnosis (i.e., cholecystitis) is recorded in the Adverse Event Form of the eCRF. Medical issues identified must be reported as AEs, with values above or below

the normal range indicated (e.g., "blood potassium increased" should be recorded rather than "blood potassium abnormal"); if there are corresponding standard clinical terms for the laboratory test abnormalities or vital sign abnormalities, clinical terms should be recorded in the eCRF, e.g., blood potassium increased to 5.5 mmol/L should be recorded as "hyperkalemia".

Hepatic function abnormal

The following events must be reported as AEs by the investigator to the sponsor within 24 hours of awareness with the most appropriate diagnoses or the laboratory abnormal values (when the diagnoses cannot be established) recorded on the AE Form of the eCRF, whether it is an SAE or not:

- ALT/AST≥10 × ULN；
- ALT/AST ≥ 5 × ULN and lasting more than 2 weeks;
- Or ALT/AST ≥ 5 × ULN with total bilirubin ≥ 3 × ULN; or
- ALT/AST ≥ 5 × ULN with gastrointestinal symptoms (e.g., nausea, vomiting, tenderness of right upper abdomen) and treatment-emergent ALT/AST > 10 × ULN.

Pregnancy and Intrauterine Exposure

To ensure the safety of the subject, pregnancy events occurring in the subject or the subjects' partner during treatment with the study drug or within 6 months after discontinuation of the study drug should be reported to the sponsor within 24 hours of awareness. Pregnant patients should be followed up to determine the pregnancy outcomes, including spontaneous abortion or voluntary termination of the pregnancy, details related to the birth of the infant, and the presence of birth defects, congenital abnormalities, or maternal genetic and/or neonatal complications.

Pregnancy events should be recorded on the clinical study pregnancy form, and reported by the investigator to the sponsor or its designee. Follow-up information of the pregnancy should be recorded on the same form, which should include an evaluation on the relationship between the study drug and the pregnancy outcome. All SAEs occurring during pregnancy must be recorded and reported on the Serious Adverse Events Report Form. Pregnancy outcomes of the female partners of the male subjects receiving study drug in this study must be collected. Informed consent regarding these pregnancy outcomes should be obtained from the mother when reporting.

Adverse pregnancy outcomes such as spontaneous abortion, fetal death, fetal malformation, stillbirth, birth defect, or congenital anomaly should be reported as SAEs.

Overdose, Medication Error and Abuse

An overdose is defined as more than 20% of the dose administered as specified in the protocol.

If a subject experiences an overdose, medication error, abuse, or misuse in the study, regardless of whether there is a relevant AE, the investigator should report it to the sponsor within 24 hours of awareness. If the above situations lead to SAEs, the investigator should report the SAEs.

9 DATA ANALYSIS AND STATISTICS

STATISTICAL METHODS

Study results will be summarized using descriptive statistics, i.e., statistics for continuous variables may include mean, median, range, and standard deviation/variability. Qualitative variables will be summarized by counts and percentages. The uncertainty of estimates will be assessed by confidence intervals (CIs). The specific analytical methods will be described in the SAP.

SAMPLE SIZE CALCULATION

In this study, 80-122 subjects are planned to be enrolled in the cohort treated with KN026 + KN046, including 30-36 subjects in the HER2-positive breast cancer cohort, 30-60 subjects in the first-line or ≥ 2nd-line HER2-positive gastric cancer cohort, and 20-26 subjects in other solid tumors. The sample size calculation is based on the estimate of the 95% CI for ORR using the Clopper Pearson method:

Sampling Volume	Number of Subjects Achieving Objective Response	ORR, %	ORR 95% CI
20	8	40%	(19.1%, 63.9%)
	9	45%	(23.1%, 68.5%)
	10	50%	(27.2%, 72.8%)
	11	55%	(31.5%, 76.9%)
	12	60%	(36.1%, 80.9%)
	13	65%	(40.8%, 84.6%)
	14	70%	(45.7%, 88.1%)
	15	75%	(50.9%, 91.3%)
30	12	40%	(22.7%, 59.4%)
	14	47%	(28.3%, 65.7%)
	16	53%	(34.3%, 71.7%)
	18	60%	(40.6%, 77.3%)
	20	67%	(47.2%, 82.7%)
	22	73%	(54.1%, 87.7%)
	24	80%	(61.4%, 92.3%)
40	15	37.5%	(22.7%, 54.2%)
	20	50%	(33.8%, 66.2%)
	25	50%	(45.8%, 77.3%)
	30	60%	(58.8%, 87.3%)
	35	70%	(73.2%, 95.8%)
50	20	40%	(26.4%, 54.8%)

	25	50%	(35.5%, 64.5%)
	30	60%	(45.2%, 73.6%)
	35	70%	(55.4%, 82.1%)
	40	80%	(66.3%, 90.0%)
60	25	41.7%	(29.1%, 55.2%)
	30	50%	(36.8%, 63.2%)
	35	58.3%	(44.9%, 70.9%)
	40	66.7%	(53.3%, 78.3%)
	45	75%	(62.1%, 85.3%)
	50	83.3%	(71.5%, 91.7%)

ANALYSIS SETS

The following analysis sets will be used for statistical analysis and data reporting.

Safety Set

The safety set (SS) includes all subjects who have received at least 1 full or partial dose of KN026 and/or KN046. Subjects will be categorized according to the treatment groups planned in the protocol. All raw data will be counted using SS, which will be the default analysis set used for all analyses unless otherwise noted.

Subjects who fail screening will be only listed, but not summarized for analysis.

Efficacy Analysis Set

The Efficacy Analysis Set (EAS) includes all subjects who have received at least 1 full or partial dose of KN026 and/or KN046 and have at least one postbaseline tumor imaging evaluation. This analysis set will be used for supportive analyses.

PK Analysis Set

The PK analysis set includes subjects who have received at least 1 full or partial dose of KN026 and/or KN046 and have at least 1 post-dose PK sample for analysis.

Immunogenicity Analysis Set

The immunogenicity analysis set includes subjects who have received at least 1 full dose or partial dose of KN026 and/or KN046 and have at least 1 post-dose immunogenicity sample for analysis.

DEMOGRAPHICS AND OTHER BASELINE CHARACTERISTICS

Demographic and other baseline data, including age, sex, body height, weight, ECOG score, etc. will be presented for each subject in the study report and summarized by dose level using descriptive statistics (continuous data) or frequency tables (categorical data).

ANALYSIS OF THE PRIMARY ENDPOINT

ORR and DOR: The 95% CIs calculated by Clopper Pearson method will be reported by cohort based on the SS; and the parameters of the time-related event (DOR) will be calculated using the Kaplan-Meier method by cohort.

ANALYSIS OF THE SECONDARY ENDPOINTS

Safety Endpoints

Actual cumulative dose and duration of exposure (days), dose intensity (calculated ratio of actual dose to actual treatment duration), relative dose intensity (calculated ratio of dose intensity to planned dose/planned treatment duration), number of dose delays, and treatment discontinuations will be listed and summarized for KN046 and KN026.

Safety analyses will be performed based on the Safety Analysis Set. Descriptive statistical analyses of safety endpoints will be performed by dose level. Safety analyses will be based on the incidence of treatment-emergent AEs (TEAEs), adverse events of special interests, immune-related AEs (irAEs), treatment-related AEs (TRAEs), Grade 3 or higher TEAEs, Grade 3 or higher irAEs and Grade 3 or higher TRAEs, and changes in vital signs, ECG, body weight, ECOG PS score, and laboratory values (hematology and serum chemistry). A treatment-emergent adverse event (TEAE) is defined as AEs from the first dose of KN026 and KN046 until 90 days after the last dose of KN046 (or 30 days after the last dose of KN026) or until 1 day before the initiation of a new anti-tumor therapy, whichever occurs first.

Treatment-emergent serious adverse events (TESAEs), immune-related adverse events (irAEs), and treatment-related adverse events (TRAEs) are defined as AEs from the first dose of KN026 and KN046 until 90 days after the last dose of KN046 (or 30 days after the last dose of KN026), or until 1 day before the initiation of a new anti-tumor therapy, whichever occurs first.

Adverse Event

All AEs will be coded using the Medical Dictionary for Regulatory Activities (MedDRA). The severity of AEs will be graded according to the NCI-CTCAE Version 5.0 Toxicity Rating Scale. AEs will be summarized by preferred term (PT) and system organ class (SOC) with their severities and relationship to the study drug described.

Analysis of AEs will include, but is not limited to:

- All adverse events (AEs);
- Serious adverse events (SAEs);
- Treatment-emergent adverse events (TEAEs);
- Treatment-related adverse events (TRAEs);
- Immune-related adverse events (irAEs);
- AEs leading to treatment discontinuation;
- AEs leading to death;
- AEs of special interest.

Tolerability

The tolerability of subjects to the investigational products will be evaluated by the number of subjects with dose interruption, delay, and discontinuation. Reasons for dose interruption, delay, and discontinuation will be listed, and descriptive statistical analysis will be performed on the frequency of dose interruption, delay and discontinuation by cohort.

Laboratory Abnormalities

Laboratory results will be graded according to NCI-CTCAE Version 5.0. Parameters that cannot be graded will be classified as decreased/normal/increased according to the normal range of laboratory tests.

Test values for each laboratory test (e.g., hematology, serum chemistry, etc.) will be listed by laboratory parameter, subject, and dose level. Then, the frequency of obvious laboratory abnormalities (Grade 3 or 4 laboratory test abnormalities judged per the NCI-CTCAE Version 5.0) will be described by parameters, number of treatment cycles and dose levels; and the frequency of all laboratory abnormalities will be described by parameter, worst grade (according to the NCI-CTCAE Version 5.0), and dose level.

The obvious laboratory abnormalities (Grade 3 or 4 laboratory test abnormalities judged by the NCI-CTCAE Version 5.0) will be listed. For the parameters that can be categorized using the CTCAE Version 5.0, laboratory data can be summarized by changes in grade as listed in the tables. The parameters that could not be categorized according to the CTCAE Version 5.0 are divided into 3 groups (decreased/normal/increased) according to the normal range, and the data will be listed to statistically analyze the frequency of occurrence.

Other Safety Data (Physical Examination, ECG and Vital Signs)

All ECG parameters, including the QT interval corrected according to the Fridericia method (QTc interval) for each subject, will be listed, and descriptive statistical analysis will be performed on the change from baseline for each parameter by dose level and evaluation time. Dose groups as well as correlations of changes in PK parameters (e.g., C_{max}, AUC_{0-t}) and QTc will be presented graphically.

Blood pressure, pulse, respiratory rate, body temperature, and body weight will be listed for all subjects, and changes from baseline for each value will be presented and statistically described.

All physical examination findings will be listed.

Efficacy Endpoints

The analysis of efficacy endpoints will be performed based on the EAS, and PFS and OS will be analyzed based on the SS. The 95% CIs calculated by Clopper Pearson method will be reported by dose group for individual proportion (CBR); the parameters (including median and 95% CIs) of time-related events (PFS and OS) calculated by the Kaplan-Meier method will be reported by dose group;

The specific analytical contents and analytical methods will be detailed in the SAP.

Pharmacokinetic Endpoints

The concentration-time curves will be plotted for subjects by measured plasma concentrations of KN046 and KN026 and the actual blood collection time points, and PK parameters will be calculated using a non-compartmental model; the mean (arithmetic mean and geometric mean) concentration-time curves will be plotted by measured plasma concentrations of KN046 and KN026 and the scheduled blood collection time points. Concentrations below the LLOQ will be recorded as zero and included in the calculation of PK parameters. Missing sample information will be recorded, but missing samples will not be included in the calculation of PK parameters.

Correlation between Pharmacokinetic and Clinical Efficacy Variables

Logistic regression analysis will be conducted on AUC (AUC_{0-t}, AUC_{inf}, and AUC_{last}), C_{trough} and C_{max} versus clinical efficacy variables (ORR, CBR, PFS rate, and OS rate).

Data for AUC can be derived from the results of population pharmacokinetic analysis.
Immunogenicity Endpoints

The frequency of anti-KN046 and -KN026 antibodies and neutralizing antibodies will be listed by cohort, and descriptive statistical analysis will be performed; for ADA-positive subjects, ADA titers will also be presented.

ANALYSES OF THE EXPLORATORY ENDPOINTS

Correlation between biomarkers and clinical efficacy variables (e.g., ORR, CBR, etc.). See the Statistical Analysis Plan for details.
