# Supplementary material for: Efficacy and safety of KN026, a bispecific anti-HER2 antibody, in combination with KN046, an anti-CTLA4/PD-L1 antibody, in patients with advanced HER2-positive nonbreast cancer: a combined analysis of a phase Ib and a phase II study
Source: Signal Transduct Target Ther. 2025 Mar 19;10:104. doi: 10.1038/s41392-025-02195-x (PMC11923254; doi:10.1038/s41392-025-02195-x)
Supplement: Supplementary file 5 — The table of sampling volume and ORR [file 41392_2025_2195_MOESM5_ESM.pdf]

Table Sampling Volume and ORR

Sampling Volume	Number of Subjects Achieving Objective Response	ORR,%	ORR 95% CI
15	6	40.0	(16.3, 67.7)
	7	46.7	(21.3, 73.4)
	8	53.3	(26.6, 78.7)
	9	60.0	(32.3, 83.7)
	10	66.7	(38.4, 88.2)
	11	73.3	(44.9, 92.2)
	12	80.0	(51.9, 95.7)
16	6	37.5	(15.2, 64.6)
	7	43.8	(19.8, 70.1)
	8	50.0	(24.7, 75.3)
	9	56.3	(29.9, 80.2)
	10	62.5	(35.4, 84.8)
	11	68.8	(41.3, 89.0)
	12	75.0	(47.6, 92.7)
17	6	35.3	(14.2, 61.7)
	7	41.2	(18.4, 67.1)
	8	47.1	(23.0, 72.2)
	9	52.9	(27.8, 77.0)
	10	58.8	(32.9, 81.6)
	11	64.7	(38.3, 85.8)
	12	70.6	(44.0, 89.7)
	13	76.5	(50.1, 93.2)
18	7	38.9	(17.3, 64.3)
	8	44.4	(21.5, 69.2)
	9	50.0	(26.0, 74.0)
	10	55.6	(30.8, 78.5)
	11	61.1	(35.7, 82.7)
	12	66.7	(41.0, 86.7)
	13	72.2	(46.5, 90.3)
	14	77.8	(52.4, 93.6)
19	7	36.8	(16.3, 61.6)
	8	42.1	(20.3, 66.5)
	9	47.4	(24.4, 71.1)
	10	52.6	(28.9, 75.6)
	11	57.9	(33.5, 79.7)
	12	63.2	(38.4, 83.7)
	13	68.4	(43.4, 87.4)
	14	73.7	(48.8, 90.9)
	15	78.9	(54.4, 93.9)
20	8	40.0	(19.1, 63.9)
	9	45.0	(23.1, 68.5)
	10	50.0	(27.2, 72.8)
	11	55.0	(31.5, 76.9)
	12	60.0	(36.1, 80.9)

Sampling Volume	Number of Subjects Achieving Objective Response	ORR,%	ORR 95% CI
20	13	65.0	(40.8, 84.6)
	14	70.0	(45.7, 88.1)
	15	75.0	(50.9, 91.3)
	16	80.0	(56.3, 94.3)
21	8	38.1	(18.1, 61.6)
	9	42.9	(21.8, 66.0)
	10	47.6	(25.7, 70.2)
	11	52.4	(29.8, 74.3)
	12	57.1	(34.0, 78.2)
	13	61.9	(38.4, 81.9)
	14	66.7	(43.0, 85.4)
	15	71.4	(47.8, 88.7)
	16	76.2	(52.8, 91.8)
22	8	36.4	(17.2, 59.3)
	9	40.9	(20.7, 63.6)
	10	45.5	(24.4, 67.8)
	11	50.0	(28.2, 71.8)
	12	54.5	(32.2, 75.6)
	13	59.1	(36.4, 79.3)
	14	63.6	(40.7, 82.8)
	15	68.2	(45.1, 86.1)
	16	72.7	(49.8, 89.3)
	17	77.3	(54.6, 92.2)
23	9	39.1	(19.7, 61.5)
	10	43.5	(23.2, 65.5)
	11	47.8	(26.8, 69.4)
	12	52.2	(30.6, 73.2)
	13	56.5	(34.5, 76.8)
	14	60.9	(38.5, 80.3)
	15	65.2	(42.7, 83.6)
	16	69.6	(47.1, 86.8)
	17	73.9	(51.6, 89.8)
	18	78.3	(56.3, 92.5)
24	9	37.5	(18.8, 59.4)
	10	41.7	(22.1, 63.4)
	11	45.8	(25.6, 67.2)
	12	50.0	(29.1, 70.9)
	13	54.2	(32.8, 74.4)
	14	58.3	(36.6, 77.9)
	15	62.5	(40.6, 81.2)
	16	66.7	(44.7, 84.4)
	17	70.8	(48.9, 87.4)
	18	75.0	(53.3, 90.2)
	19	79.2	(57.8, 92.9)
25	10	40.0	(21.1, 61.3)

Sampling Volume	Number of Subjects Achieving Objective Response	ORR,%	ORR 95% CI
25	11	44.0	(24.4, 65.1)
	12	48.0	(27.8, 68.7)
	13	52.0	(31.3, 72.2)
	14	56.0	(34.9, 75.6)
	15	60.0	(38.7, 78.9)
	16	64.0	(42.5, 82.0)
	17	68.0	(46.5, 85.1)
	18	72.0	(50.6, 87.9)
	19	76.0	(54.9, 90.6)
	20	80.0	(59.3, 93.2)
26	10	38.5	(20.2, 59.4)
	11	42.3	(23.4, 63.1)
	12	46.2	(26.6, 66.6)
	13	50.0	(29.9, 70.1)
	14	53.8	(33.4, 73.4)
	15	57.7	(36.9, 76.6)
	16	61.5	(40.6, 79.8)
	17	65.4	(44.3, 82.8)
	18	69.2	(48.2, 85.7)
	19	73.1	(52.2, 88.4)
	20	76.9	(56.4, 91.0)
27	10	37.0	(19.4, 57.6)
	11	40.7	(22.4, 61.2)
	12	44.4	(25.5, 64.7)
	13	48.1	(28.7, 68.1)
	14	51.9	(31.9, 71.3)
	15	55.6	(35.3, 74.5)
	16	59.3	(38.8, 77.6)
	17	63.0	(42.4, 80.6)
	18	66.7	(46.0, 83.5)
	19	70.4	(49.8, 86.2)
	20	74.1	(53.7, 88.9)
	21	77.8	(57.7, 91.4)
28	11	39.3	(21.5, 59.4)
	12	42.9	(24.5, 62.8)
	13	46.4	(27.5, 66.1)
	14	50.0	(30.6, 69.4)
	15	53.6	(33.9, 72.5)
	16	57.1	(37.2, 75.5)
	17	60.7	(40.6, 78.5)
	18	64.3	(44.1, 81.4)
	19	67.9	(47.6, 84.1)
	20	71.4	(51.3, 86.8)
	21	75.0	(55.1, 89.3)
	22	78.6	(59.0, 91.7)

Sampling Volume	Number of Subjects Achieving Objective Response	ORR,%	ORR 95% CI
29	11	37.9	(20.7, 57.7)
	12	41.4	(23.5, 61.1)
	13	44.8	(26.4, 64.3)
	14	48.3	(29.4, 67.5)
	15	51.7	(32.5, 70.6)
	16	55.2	(35.7, 73.6)
	17	58.6	(38.9, 76.5)
	18	62.1	(42.3, 79.3)
	19	65.5	(45.7, 82.1)
	20	69.0	(49.2, 84.7)
	21	72.4	(52.8, 87.3)
	22	75.9	(56.5, 89.7)
	23	79.3	(60.3, 92.0)
30	12	40.0	(22.7, 59.4)
	13	43.3	(25.5, 62.6)
	14	46.7	(28.3, 65.7)
	15	50.0	(31.3, 68.7)
	16	53.3	(34.3, 71.7)
	17	56.7	(37.4, 74.5)
	18	60.0	(40.6, 77.3)
	19	63.3	(43.9, 80.1)
	20	66.7	(47.2, 82.7)
	21	70.0	(50.6, 85.3)
	22	73.3	(54.1, 87.7)
	23	76.7	(57.7, 90.1)
	24	80.0	(61.4, 92.3)
31	12	38.7	(21.8, 57.8)
	13	41.9	(24.5, 60.9)
	14	45.2	(27.3, 64.0)
	15	48.4	(30.2, 66.9)
	16	51.6	(33.1, 69.8)
	17	54.8	(36.0, 72.7)
	18	58.1	(39.1, 75.5)
	19	61.3	(42.2, 78.2)
	20	64.5	(45.4, 80.8)
	21	67.7	(48.6, 83.3)
	22	71.0	(52.0, 85.8)
	23	74.2	(55.4, 88.1)
	24	77.4	(58.9, 90.4)
32	12	37.5	(21.1, 56.3)
	13	40.6	(23.7, 59.4)
	14	43.8	(26.4, 62.3)
	15	46.9	(29.1, 65.3)
	16	50.0	(31.9, 68.1)
	17	53.1	(34.7, 70.9)

Sampling Volume	Number of Subjects Achieving Objective Response	ORR,%	ORR 95% CI
32	18	56.3	(37.7, 73.6)
	19	59.4	(40.6, 76.3)
	20	62.5	(43.7, 78.9)
	21	65.6	(46.8, 81.4)
	22	68.8	(50.0, 83.9)
	23	71.9	(53.3, 86.3)
	24	75.0	(56.6, 88.5)
	25	78.1	(60.0, 90.7)
33	13	39.4	(22.9, 57.9)
	14	42.4	(25.5, 60.8)
	15	45.5	(28.1, 63.6)
	16	48.5	(30.8, 66.5)
	17	51.5	(33.5, 69.2)
	18	54.5	(36.4, 71.9)
	19	57.6	(39.2, 74.5)
	20	60.6	(42.1, 77.1)
	21	63.6	(45.1, 79.6)
	22	66.7	(48.2, 82.0)
	23	69.7	(51.3, 84.4)
	24	72.7	(54.5, 86.7)
	25	75.8	(57.7, 88.9)
	26	78.8	(61.1, 91.0)
34	13	38.2	(22.2, 56.4)
	14	41.2	(24.6, 59.3)
	15	44.1	(27.2, 62.1)
	16	47.1	(29.8, 64.9)
	17	50.0	(32.4, 67.6)
	18	52.9	(35.1, 70.2)
	19	55.9	(37.9, 72.8)
	20	58.8	(40.7, 75.4)
	21	61.8	(43.6, 77.8)
	22	64.7	(46.5, 80.3)
	23	67.6	(49.5, 82.6)
	24	70.6	(52.5, 84.9)
	25	73.5	(55.6, 87.1)
	26	76.5	(58.8, 89.3)
	27	79.4	(62.1, 91.3)
35	14	40.0	(23.9, 57.9)
	15	42.9	(26.3, 60.6)
	16	45.7	(28.8, 63.4)
	17	48.6	(31.4, 66.0)
	18	51.4	(34.0, 68.6)
	19	54.3	(36.6, 71.2)
	20	57.1	(39.4, 73.7)
	21	60.0	(42.1, 76.1)

Sampling Volume	Number of Subjects Achieving Objective Response	ORR,%	ORR 95% CI
35	22	62.9	(44.9, 78.5)
	23	65.7	(47.8, 80.9)
	24	68.6	(50.7, 83.1)
	25	71.4	(53.7, 85.4)
	26	74.3	(56.7, 87.5)
	27	77.1	(59.9, 89.6)
	28	80.0	(63.1, 91.6)
36	14	38.9	(23.1, 56.5)
	15	41.7	(25.5, 59.2)
	16	44.4	(27.9, 61.9)
	17	47.2	(30.4, 64.5)
	18	50.0	(32.9, 67.1)
	19	52.8	(35.5, 69.6)
	20	55.6	(38.1, 72.1)
	21	58.3	(40.8, 74.5)
	22	61.1	(43.5, 76.9)
	23	63.9	(46.2, 79.2)
	24	66.7	(49.0, 81.4)
	25	69.4	(51.9, 83.7)
	26	72.2	(54.8, 85.8)
	27	75.0	(57.8, 87.9)
	28	77.8	(60.8, 89.9)
37	14	37.8	(22.5, 55.2)
	15	40.5	(24.8, 57.9)
	16	43.2	(27.1, 60.5)
	17	45.9	(29.5, 63.1)
	18	48.6	(31.9, 65.6)
	19	51.4	(34.4, 68.1)
	20	54.1	(36.9, 70.5)
	21	56.8	(39.5, 72.9)
	22	59.5	(42.1, 75.2)
	23	62.2	(44.8, 77.5)
	24	64.9	(47.5, 79.8)
	25	67.6	(50.2, 82.0)
	26	70.3	(53.0, 84.1)
	27	73.0	(55.9, 86.2)
	28	75.7	(58.8, 88.2)
	29	78.4	(61.8, 90.2)
38	15	39.5	(24.0, 56.6)
	16	42.1	(26.3, 59.2)
	17	44.7	(28.6, 61.7)
	18	47.4	(31.0, 64.2)
	19	50.0	(33.4, 66.6)
	20	52.6	(35.8, 69.0)
	21	55.3	(38.3, 71.4)

Sampling Volume	Number of Subjects Achieving Objective Response	ORR,%	ORR 95% CI
38	22	57.9	(40.8, 73.7)
	23	60.5	(43.4, 76.0)
	24	63.2	(46.0, 78.2)
	25	65.8	(48.6, 80.4)
	26	68.4	(51.3, 82.5)
	27	71.1	(54.1, 84.6)
	28	73.7	(56.9, 86.6)
	29	76.3	(59.8, 88.6)
	30	78.9	(62.7, 90.4)
39	15	38.5	(23.4, 55.4)
	16	41.0	(25.6, 57.9)
	17	43.6	(27.8, 60.4)
	18	46.2	(30.1, 62.8)
	19	48.7	(32.4, 65.2)
	20	51.3	(34.8, 67.6)
	21	53.8	(37.2, 69.9)
	22	56.4	(39.6, 72.2)
	23	59.0	(42.1, 74.4)
	24	61.5	(44.6, 76.6)
	25	64.1	(47.2, 78.8)
	26	66.7	(49.8, 80.9)
	27	69.2	(52.4, 83.0)
	28	71.8	(55.1, 85.0)
	29	74.4	(57.9, 87.0)
	30	76.9	(60.7, 88.9)
	31	79.5	(63.5, 90.7)
40	16	40.0	(24.9, 56.7)
	17	42.5	(27.0, 59.1)
	18	45.0	(29.3, 61.5)
	19	47.5	(31.5, 63.9)
	20	50.0	(33.8, 66.2)
	21	52.5	(36.1, 68.5)
	22	55.0	(38.5, 70.7)
	23	57.5	(40.9, 73.0)
	24	60.0	(43.3, 75.1)
	25	62.5	(45.8, 77.3)
	26	65.0	(48.3, 79.4)
	27	67.5	(50.9, 81.4)
	28	70.0	(53.5, 83.4)
	29	72.5	(56.1, 85.4)
	30	75.0	(58.8, 87.3)
	31	77.5	(61.5, 89.2)
	32	80.0	(64.4, 90.9)
41	16	39.0	(24.2, 55.5)
	17	41.5	(26.3, 57.9)

Sampling Volume	Number of Subjects Achieving Objective Response	ORR,%	ORR 95% CI
41	18	43.9	(28.5, 60.3)
	19	46.3	(30.7, 62.6)
	20	48.8	(32.9, 64.9)
	21	51.2	(35.1, 67.1)
	22	53.7	(37.4, 69.3)
	23	56.1	(39.7, 71.5)
	24	58.5	(42.1, 73.7)
	25	61.0	(44.5, 75.8)
	26	63.4	(46.9, 77.9)
	27	65.9	(49.4, 79.9)
	28	68.3	(51.9, 81.9)
	29	70.7	(54.5, 83.9)
	30	73.2	(57.1, 85.8)
	31	75.6	(59.7, 87.6)
	32	78.0	(62.4, 89.4)
42	16	38.1	(23.6, 54.4)
	17	40.5	(25.6, 56.7)
	18	42.9	(27.7, 59.0)
	19	45.2	(29.8, 61.3)
	20	47.6	(32.0, 63.6)
	21	50.0	(34.2, 65.8)
	22	52.4	(36.4, 68.0)
	23	54.8	(38.7, 70.2)
	24	57.1	(41.0, 72.3)
	25	59.5	(43.3, 74.4)
	26	61.9	(45.6, 76.4)
	27	64.3	(48.0, 78.4)
	28	66.7	(50.5, 80.4)
	29	69.0	(52.9, 82.4)
	30	71.4	(55.4, 84.3)
	31	73.8	(58.0, 86.1)
	32	76.2	(60.5, 87.9)
	33	78.6	(63.2, 89.7)
43	17	39.5	(25.0, 55.6)
	18	41.9	(27.0, 57.9)
	19	44.2	(29.1, 60.1)
	20	46.5	(31.2, 62.3)
	21	48.8	(33.3, 64.5)
	22	51.2	(35.5, 66.7)
	23	53.5	(37.7, 68.8)
	24	55.8	(39.9, 70.9)
	25	58.1	(42.1, 73.0)
	26	60.5	(44.4, 75.0)
	27	62.8	(46.7, 77.0)
	28	65.1	(49.1, 79.0)

Sampling Volume	Number of Subjects Achieving Objective Response	ORR,%	ORR 95% CI
43	29	67.4	(51.5, 80.9)
	30	69.8	(53.9, 82.8)
	31	72.1	(56.3, 84.7)
	32	74.4	(58.8, 86.5)
	33	76.7	(61.4, 88.2)
	34	79.1	(64.0, 90.0)
44	17	38.6	(24.4, 54.5)
	18	40.9	(26.3, 56.8)
	19	43.2	(28.3, 59.0)
	20	45.5	(30.4, 61.2)
	21	47.7	(32.5, 63.3)
	22	50.0	(34.6, 65.4)
	23	52.3	(36.7, 67.5)
	24	54.5	(38.8, 69.6)
	25	56.8	(41.0, 71.7)
	26	59.1	(43.2, 73.7)
	27	61.4	(45.5, 75.6)
	28	63.6	(47.8, 77.6)
	29	65.9	(50.1, 79.5)
	30	68.2	(52.4, 81.4)
	31	70.5	(54.8, 83.2)
	32	72.7	(57.2, 85.0)
	33	75.0	(59.7, 86.8)
	34	77.3	(62.2, 88.5)
	35	79.5	(64.7, 90.2)
45	18	40.0	(25.7, 55.7)
	19	42.2	(27.7, 57.8)
	20	44.4	(29.6, 60.0)
	21	46.7	(31.7, 62.1)
	22	48.9	(33.7, 64.2)
	23	51.1	(35.8, 66.3)
	24	53.3	(37.9, 68.3)
	25	55.6	(40.0, 70.4)
	26	57.8	(42.2, 72.3)
	27	60.0	(44.3, 74.3)
	28	62.2	(46.5, 76.2)
	29	64.4	(48.8, 78.1)
	30	66.7	(51.0, 80.0)
	31	68.9	(53.4, 81.8)
	32	71.1	(55.7, 83.6)
	33	73.3	(58.1, 85.4)
	34	75.6	(60.5, 87.1)
	35	77.8	(62.9, 88.8)
	36	80.0	(65.4, 90.4)
46	18	39.1	(25.1, 54.6)

Sampling Volume	Number of Subjects Achieving Objective Response	ORR,%	ORR 95% CI
46	19	41.3	(27.0, 56.8)
	20	43.5	(28.9, 58.9)
	21	45.7	(30.9, 61.0)
	22	47.8	(32.9, 63.1)
	23	50.0	(34.9, 65.1)
	24	52.2	(36.9, 67.1)
	25	54.3	(39.0, 69.1)
	26	56.5	(41.1, 71.1)
	27	58.7	(43.2, 73.0)
	28	60.9	(45.4, 74.9)
	29	63.0	(47.5, 76.8)
	30	65.2	(49.8, 78.6)
	31	67.4	(52.0, 80.5)
	32	69.6	(54.2, 82.3)
	33	71.7	(56.5, 84.0)
	34	73.9	(58.9, 85.7)
	35	76.1	(61.2, 87.4)
	36	78.3	(63.6, 89.1)
47	18	38.3	(24.5, 53.6)
	19	40.4	(26.4, 55.7)
	20	42.6	(28.3, 57.8)
	21	44.7	(30.2, 59.9)
	22	46.8	(32.1, 61.9)
	23	48.9	(34.1, 63.9)
	24	51.1	(36.1, 65.9)
	25	53.2	(38.1, 67.9)
	26	55.3	(40.1, 69.8)
	27	57.4	(42.2, 71.7)
	28	59.6	(44.3, 73.6)
	29	61.7	(46.4, 75.5)
	30	63.8	(48.5, 77.3)
	31	66.0	(50.7, 79.1)
	32	68.1	(52.9, 80.9)
	33	70.2	(55.1, 82.7)
	34	72.3	(57.4, 84.4)
	35	74.5	(59.7, 86.1)
	36	76.6	(62.0, 87.7)
	37	78.7	(64.3, 89.3)
48	19	39.6	(25.8, 54.7)
	20	41.7	(27.6, 56.8)
	21	43.8	(29.5, 58.8)
	22	45.8	(31.4, 60.8)
	23	47.9	(33.3, 62.8)
	24	50.0	(35.2, 64.8)
	25	52.1	(37.2, 66.7)

Sampling Volume	Number of Subjects Achieving Objective Response	ORR,%	ORR 95% CI
48	26	54.2	(39.2, 68.6)
	27	56.3	(41.2, 70.5)
	28	58.3	(43.2, 72.4)
	29	60.4	(45.3, 74.2)
	30	62.5	(47.4, 76.0)
	31	64.6	(49.5, 77.8)
	32	66.7	(51.6, 79.6)
	33	68.8	(53.7, 81.3)
	34	70.8	(55.9, 83.0)
	35	72.9	(58.2, 84.7)
	36	75.0	(60.4, 86.4)
	37	77.1	(62.7, 88.0)
	38	79.2	(65.0, 89.5)
49	19	38.8	(25.2, 53.8)
	20	40.8	(27.0, 55.8)
	21	42.9	(28.8, 57.8)
	22	44.9	(30.7, 59.8)
	23	46.9	(32.5, 61.7)
	24	49.0	(34.4, 63.7)
	25	51.0	(36.3, 65.6)
	26	53.1	(38.3, 67.5)
	27	55.1	(40.2, 69.3)
	28	57.1	(42.2, 71.2)
	29	59.2	(44.2, 73.0)
	30	61.2	(46.2, 74.8)
	31	63.3	(48.3, 76.6)
	32	65.3	(50.4, 78.3)
	33	67.3	(52.5, 80.1)
	34	69.4	(54.6, 81.7)
	35	71.4	(56.7, 83.4)
	36	73.5	(58.9, 85.1)
	37	75.5	(61.1, 86.7)
	38	77.6	(63.4, 88.2)
	39	79.6	(65.7, 89.8)
50	20	40.0	(26.4, 54.8)
	21	42.0	(28.2, 56.8)
	22	44.0	(30.0, 58.7)
	23	46.0	(31.8, 60.7)
	24	48.0	(33.7, 62.6)
	25	50.0	(35.5, 64.5)
	26	52.0	(37.4, 66.3)
	27	54.0	(39.3, 68.2)
	28	56.0	(41.3, 70.0)
	29	58.0	(43.2, 71.8)
	30	60.0	(45.2, 73.6)

Sampling Volume	Number of Subjects Achieving Objective Response	ORR,%	ORR 95% CI
50	31	62.0	(47.2, 75.3)
	32	64.0	(49.2, 77.1)
	33	66.0	(51.2, 78.8)
	34	68.0	(53.3, 80.5)
	35	70.0	(55.4, 82.1)
	36	72.0	(57.5, 83.8)
	37	74.0	(59.7, 85.4)
	38	76.0	(61.8, 86.9)
	39	78.0	(64.0, 88.5)
	40	80.0	(66.3, 90.0)
51	20	39.2	(25.8, 53.9)
	21	41.2	(27.6, 55.8)
	22	43.1	(29.3, 57.8)
	23	45.1	(31.1, 59.7)
	24	47.1	(32.9, 61.5)
	25	49.0	(34.8, 63.4)
	26	51.0	(36.6, 65.2)
	27	52.9	(38.5, 67.1)
	28	54.9	(40.3, 68.9)
	29	56.9	(42.2, 70.7)
	30	58.8	(44.2, 72.4)
	31	60.8	(46.1, 74.2)
	32	62.7	(48.1, 75.9)
	33	64.7	(50.1, 77.6)
	34	66.7	(52.1, 79.2)
	35	68.6	(54.1, 80.9)
	36	70.6	(56.2, 82.5)
	37	72.5	(58.3, 84.1)
	38	74.5	(60.4, 85.7)
	39	76.5	(62.5, 87.2)
	40	78.4	(64.7, 88.7)
52	20	38.5	(25.3, 53.0)
	21	40.4	(27.0, 54.9)
	22	42.3	(28.7, 56.8)
	23	44.2	(30.5, 58.7)
	24	46.2	(32.2, 60.5)
	25	48.1	(34.0, 62.4)
	26	50.0	(35.8, 64.2)
	27	51.9	(37.6, 66.0)
	28	53.8	(39.5, 67.8)
	29	55.8	(41.3, 69.5)
	30	57.7	(43.2, 71.3)
	31	59.6	(45.1, 73.0)
	32	61.5	(47.0, 74.7)
	33	63.5	(49.0, 76.4)

Sampling Volume	Number of Subjects Achieving Objective Response	ORR,%	ORR 95% CI
52	34	65.4	(50.9, 78.0)
	35	67.3	(52.9, 79.7)
	36	69.2	(54.9, 81.3)
	37	71.2	(56.9, 82.9)
	38	73.1	(59.0, 84.4)
	39	75.0	(61.1, 86.0)
	40	76.9	(63.2, 87.5)
	41	78.8	(65.3, 88.9)
53	21	39.6	(26.5, 54.0)
	22	41.5	(28.1, 55.9)
	23	43.4	(29.8, 57.7)
	24	45.3	(31.6, 59.6)
	25	47.2	(33.3, 61.4)
	26	49.1	(35.1, 63.2)
	27	50.9	(36.8, 64.9)
	28	52.8	(38.6, 66.7)
	29	54.7	(40.4, 68.4)
	30	56.6	(42.3, 70.2)
	31	58.5	(44.1, 71.9)
	32	60.4	(46.0, 73.5)
	33	62.3	(47.9, 75.2)
	34	64.2	(49.8, 76.9)
	35	66.0	(51.7, 78.5)
	36	67.9	(53.7, 80.1)
	37	69.8	(55.7, 81.7)
	38	71.7	(57.7, 83.2)
	39	73.6	(59.7, 84.7)
	40	75.5	(61.7, 86.2)
	41	77.4	(63.8, 87.7)
	42	79.2	(65.9, 89.2)
54	21	38.9	(25.9, 53.1)
	22	40.7	(27.6, 55.0)
	23	42.6	(29.2, 56.8)
	24	44.4	(30.9, 58.6)
	25	46.3	(32.6, 60.4)
	26	48.1	(34.3, 62.2)
	27	50.0	(36.1, 63.9)
	28	51.9	(37.8, 65.7)
	29	53.7	(39.6, 67.4)
	30	55.6	(41.4, 69.1)
	31	57.4	(43.2, 70.8)
	32	59.3	(45.0, 72.4)
	33	61.1	(46.9, 74.1)
	34	63.0	(48.7, 75.7)
	35	64.8	(50.6, 77.3)

Sampling Volume	Number of Subjects Achieving Objective Response	ORR,%	ORR 95% CI
54	36	66.7	(52.5, 78.9)
	37	68.5	(54.4, 80.5)
	38	70.4	(56.4, 82.0)
	39	72.2	(58.4, 83.5)
	40	74.1	(60.3, 85.0)
	41	75.9	(62.4, 86.5)
	42	77.8	(64.4, 88.0)
	43	79.6	(66.5, 89.4)
55	22	40.0	(27.0, 54.1)
	23	41.8	(28.7, 55.9)
	24	43.6	(30.3, 57.7)
	25	45.5	(32.0, 59.4)
	26	47.3	(33.7, 61.2)
	27	49.1	(35.4, 62.9)
	28	50.9	(37.1, 64.6)
	29	52.7	(38.8, 66.3)
	30	54.5	(40.6, 68.0)
	31	56.4	(42.3, 69.7)
	32	58.2	(44.1, 71.3)
	33	60.0	(45.9, 73.0)
	34	61.8	(47.7, 74.6)
	35	63.6	(49.6, 76.2)
	36	65.5	(51.4, 77.8)
	37	67.3	(53.3, 79.3)
	38	69.1	(55.2, 80.9)
	39	70.9	(57.1, 82.4)
	40	72.7	(59.0, 83.9)
	41	74.5	(61.0, 85.3)
	42	76.4	(63.0, 86.8)
	43	78.2	(65.0, 88.2)
	44	80.0	(67.0, 89.6)
56	22	39.3	(26.5, 53.2)
	23	41.1	(28.1, 55.0)
	24	42.9	(29.7, 56.8)
	25	44.6	(31.3, 58.5)
	26	46.4	(33.0, 60.3)
	27	48.2	(34.7, 62.0)
	28	50.0	(36.3, 63.7)
	29	51.8	(38.0, 65.3)
	30	53.6	(39.7, 67.0)
	31	55.4	(41.5, 68.7)
	32	57.1	(43.2, 70.3)
	33	58.9	(45.0, 71.9)
	34	60.7	(46.8, 73.5)
	35	62.5	(48.5, 75.1)

Sampling Volume	Number of Subjects Achieving Objective Response	ORR,%	ORR 95% CI
56	36	64.3	(50.4, 76.6)
	37	66.1	(52.2, 78.2)
	38	67.9	(54.0, 79.7)
	39	69.6	(55.9, 81.2)
	40	71.4	(57.8, 82.7)
	41	73.2	(59.7, 84.2)
	42	75.0	(61.6, 85.6)
	43	76.8	(63.6, 87.0)
	44	78.6	(65.6, 88.4)
57	22	38.6	(26.0, 52.4)
	23	40.4	(27.6, 54.2)
	24	42.1	(29.1, 55.9)
	25	43.9	(30.7, 57.6)
	26	45.6	(32.4, 59.3)
	27	47.4	(34.0, 61.0)
	28	49.1	(35.6, 62.7)
	29	50.9	(37.3, 64.4)
	30	52.6	(39.0, 66.0)
	31	54.4	(40.7, 67.6)
	32	56.1	(42.4, 69.3)
	33	57.9	(44.1, 70.9)
	34	59.6	(45.8, 72.4)
	35	61.4	(47.6, 74.0)
	36	63.2	(49.3, 75.6)
	37	64.9	(51.1, 77.1)
	38	66.7	(52.9, 78.6)
	39	68.4	(54.8, 80.1)
	40	70.2	(56.6, 81.6)
	41	71.9	(58.5, 83.0)
	42	73.7	(60.3, 84.5)
	43	75.4	(62.2, 85.9)
	44	77.2	(64.2, 87.3)
	45	78.9	(66.1, 88.6)
58	23	39.7	(27.0, 53.4)
	24	41.4	(28.6, 55.1)
	25	43.1	(30.2, 56.8)
	26	44.8	(31.7, 58.5)
	27	46.6	(33.3, 60.1)
	28	48.3	(35.0, 61.8)
	29	50.0	(36.6, 63.4)
	30	51.7	(38.2, 65.0)
	31	53.4	(39.9, 66.7)
	32	55.2	(41.5, 68.3)
	33	56.9	(43.2, 69.8)
	34	58.6	(44.9, 71.4)

Sampling Volume	Number of Subjects Achieving Objective Response	ORR,%	ORR 95% CI
58	35	60.3	(46.6, 73.0)
	36	62.1	(48.4, 74.5)
	37	63.8	(50.1, 76.0)
	38	65.5	(51.9, 77.5)
	39	67.2	(53.7, 79.0)
	40	69.0	(55.5, 80.5)
	41	70.7	(57.3, 81.9)
	42	72.4	(59.1, 83.3)
	43	74.1	(61.0, 84.7)
	44	75.9	(62.8, 86.1)
	45	77.6	(64.7, 87.5)
	46	79.3	(66.6, 88.8)
59	23	39.0	(26.5, 52.6)
	24	40.7	(28.1, 54.3)
	25	42.4	(29.6, 55.9)
	26	44.1	(31.2, 57.6)
	27	45.8	(32.7, 59.2)
	28	47.5	(34.3, 60.9)
	29	49.2	(35.9, 62.5)
	30	50.8	(37.5, 64.1)
	31	52.5	(39.1, 65.7)
	32	54.2	(40.8, 67.3)
	33	55.9	(42.4, 68.8)
	34	57.6	(44.1, 70.4)
	35	59.3	(45.7, 71.9)
	36	61.0	(47.4, 73.5)
	37	62.7	(49.1, 75.0)
	38	64.4	(50.9, 76.4)
	39	66.1	(52.6, 77.9)
	40	67.8	(54.4, 79.4)
	41	69.5	(56.1, 80.8)
	42	71.2	(57.9, 82.2)
	43	72.9	(59.7, 83.6)
	44	74.6	(61.6, 85.0)
	45	76.3	(63.4, 86.4)
	46	78.0	(65.3, 87.7)
	47	79.7	(67.2, 89.0)
60	24	40.0	(27.6, 53.5)
	25	41.7	(29.1, 55.1)
	26	43.3	(30.6, 56.8)
	27	45.0	(32.1, 58.4)
	28	46.7	(33.7, 60.0)
	29	48.3	(35.2, 61.6)
	30	50.0	(36.8, 63.2)
	31	51.7	(38.4, 64.8)

Sampling Volume	Number of Subjects Achieving Objective Response	ORR,%	ORR 95% CI
60	32	53.3	(40.0, 66.3)
	33	55.0	(41.6, 67.9)
	34	56.7	(43.2, 69.4)
	35	58.3	(44.9, 70.9)
	36	60.0	(46.5, 72.4)
	37	61.7	(48.2, 73.9)
	38	63.3	(49.9, 75.4)
	39	65.0	(51.6, 76.9)
	40	66.7	(53.3, 78.3)
	41	68.3	(55.0, 79.7)
	42	70.0	(56.8, 81.2)
	43	71.7	(58.6, 82.5)
	44	73.3	(60.3, 83.9)
	45	75.0	(62.1, 85.3)
	46	76.7	(64.0, 86.6)
	47	78.3	(65.8, 87.9)
	48	80.0	(67.7, 89.2)
